# Relatively Small Contribution of Methylation and Genomic Copy Number Aberration to the Aberrant Expression of Inflammation-Related Genes in HBV-Related Hepatocellular Carcinoma

**DOI:** 10.1371/journal.pone.0126836

**Published:** 2015-05-12

**Authors:** Dianke Yu, Guosheng Zhang, Xudong Huang, Chen Wu, Wen Tan, Yan Qiao, Jiang Chang, Hong Zhao, Xinyu Bi, Jianqiang Cai, Yun Li, Dongxin Lin

**Affiliations:** 1 State Key Laboratory of Molecular Oncology and Department of Etiology & Carcinogenesis, Cancer Institute and Hospital, Chinese Academy of Medical Sciences and Peking Union Medical College, Beijing, China; 2 Department of Genetics, University of North Carolina, Chapel Hill, North Carolina, United States of America; 3 Department of Biostatistics, University of North Carolina, Chapel Hill, North Carolina, United States of America; 4 Department of Abdominal Surgery, Cancer Institute and Hospital, Chinese Academy of Medical Sciences and Peking Union Medical College, Beijing, China; The University of Hong Kong, CHINA

## Abstract

**Background:**

It is well known that chronic inflammation plays a pivotal role in the development of hepatitis B virus (HBV) related hepatocellular carcinoma (HCC). However, the causes behind aberrant expression of inflammation-related genes occurred in HCC remain unclear.

**Methods:**

We performed array-based analyses to comprehensively investigate the contributions of DNA methylation and somatic copy number aberration (SCNA) to the aberrant expression of 1,027 inflammation-related genes in 30 HCCs and paired non-tumor tissues. The results were validated in public datasets and an additional sample set of 47 paired HCCs and non-tumor tissues.

**Results:**

We identified 252 differentially expressed, 125 aberrantly methylated and 287 copy number changed inflammation-related genes. Despite reasonable statistical power, among them, only 11 genes and 56 genes whose aberrant expression was associated with DNA methylation or SCNA, respectively. DNA methylation and SCNA together contributed to less than 30% aberrant expression of inflammation-related genes.

**Conclusion:**

These results suggest that molecular mechanisms other than DNA methylation and SCNA might play major role in the regulation of aberrant expression of inflammation-related gene in HBV-related HCCs.

## Introduction

Hepatocellular carcinoma (HCC) is one of the most common cancers in the world. The major risk factors for the development of HCC include infection with hepatitis B virus (HBV) or hepatitis C virus (HCV), aflatoxin exposure, and chronic alcohol abuse [1]. It has been reported that about 55% of HCC occurs in China, where the major etiological factor is chronic HBV infection [2]. HCC is one clear example of inflammation-related cancers, with chronic inflammation being indispensable in its development. It has been shown that chronic liver inflammation due to persistent HBV or HCV infection may lead to cirrhosis, which can eventually progress to HCC at an incidence rate that is 4–5 times higher than that among asymptomatic HBV carriers [3].

During the past decades, evidence has been accumulated to show that dysregulation of inflammation-related genes plays important roles in the development of HCCs [4, 5]. It was reported that both chromosomal aberrations such as copy number loss or gain and epigenome deregulation by DNA methylation, histone modification and non-coding RNAs may contribute to the aberrant expression of inflammation-related genes [6–9]. However, current knowledge is mainly derived from incomplete studies focusing on only single or a few such genes, using either a candidate gene or genome-wide approach. To the best of our knowledge, there are few, if any, comprehensive studies employing integrative analysis to simultaneously interrogate both genetic and epigenetic events contributing to the aberrant expression of genes in the inflammation pathway in HBV-related HCCs.

In this current study, we used high throughput array-based technology to comprehensively analyze the relationship between DNA methylation or somatic copy number aberration (SCNA) and aberrant expression of 1,027 genes in the inflammation pathway [10] in HBV-related HCCs. We validated our array-based results in public datasets and in an additional sample set of HCCs and paired non-tumor tissues. Our data indicated that DNA methylation and SCNA indeed cause some inflammation-related genes to be aberrantly expressed, but they only contribute about 30% aberrant expression of these genes in HBV-related HCCs.

## Materials and Methods

### HCC samples

In this study, we performed a two-stage analysis in total up to 77 HCCs and their paired non-tumor tissues (more than 2 cm from tumor). In brief, 30 HCCs and their paired non-tumor specimens were randomly selected for the discovery phase and the rest 47 HCCs and their paired non-tumor tissues were used for the validation phase. All tumor and their paired non-tumor tissues were obtained from hepatectomy of patients with HCC between 2010 and 2013 at Cancer Hospital, Chinese Academy of Medical Sciences (Beijing). The samples were immediately frozen in liquid nitrogen upon surgically resected. The diagnosis of HCC was all confirmed by histopathology. We selected the samples for HBV positive but HCV negative according to serology tests and infection history. Patients who had received chemotherapy or radiotherapy were excluded from this study. We also collected clinical characteristics of each subject in this study, which are shown in S1 Table. All patients signed an informed consent and this study was approved by the Institutional Review Board of the Chinese Academy of Medical Sciences Cancer Institute.

### Arrays used in the discovery phase

In the discovery phase, high-throughput screening was performed using Affymetrix Human Gene 1.0 ST Array (Affymetrix, Santa Clara, CA), Nimblegen 3×720 K CpG Island Plus RefSeq Promoter Array (Roche NimbleGen, Madison, WI) and Affymetrix GeneChip Human Mapping 6.0 array (Affymetrix) to measure mRNA expression, DNA methylation and copy number changes in HCCs and paired non-tumor specimens, respectively. Referring to the corresponding array design database, we confirmed that the Affymetrix Human Gene 1.0 ST Array interrogates all 1,027 inflammation-related genes described by Loza et al. [10] with 1,108 transcripts; and the Nimblegen 3×720 K CpG Island Plus RefSeq Promoter Array and Affymetrix GeneChip Human Mapping 6.0 array interrogate 38,179 probes covering 1,024 genes and 33,855 probes covering 938 genes, respectively.

### Array-based data production

Total RNA samples were extracted from 30 fresh HCCs and paired non-tumor tissues using the Trizol reagent (Life Technologies, Carlsbad, CA). Genome-wide transcriptional profiling was produced using Affymetrix Human Gene 1.0 ST Array according to the manufacturer's protocol. Arrays were processed in two batches; one included 10 arrays, and the other included 20 arrays. Raw data were first processed using Robust Multiarray Averaging [11] and batch effect was adjusted using ComBat [12]. Adjusted expression values were used in subsequent downstream analysis.

Genomic DNA samples were isolated from the same 30 fresh HCCs and paired non-tumor specimens using a commercial DNeasy Blood & Tissue Kit (QIAGEN, Valencia, CA). Each DNA sample was then divided into two portions. One portion was bisulfite-converted using the EZ DNA Methylation kit (Zymo Research, Irvine, CA), and the DNA methylation profiles was obtained with the MeDIP-chip platform based on the Nimblegen 3×720 K CpG Island Plus RefSeq Promoter Array according to the manufacturer's protocol. Arrays were also processed in two batches in the same manner as afore-described for gene expression quantification. The pre-processing of DNA methylation array data was similar to that used for gene expression array data. We first normalized the raw data using control probes designed in the methylation array, and then adjusted batch effect with ComBat.

The other portion of genomic DNA sample was used to detect DNA copy number aberrations using the Affymetrix GeneChip Human Mapping 6.0 set according to the manufacturer's protocol. Affymetrix Power Tools was used on the raw data to generate signal intensities, which were further analyzed by PennCNV [13] to call probe-based copy numbers. Log R ratios (LRR) estimated by PennCNV were carried forward for further analysis.

### Array-based data analysis

Paired student's *t*-test was used to determine whether the difference in gene expression, DNA methylation or copy numbers of inflammation-related genes between HCC tissues and matched non-tumor liver tissues is significant. Bonferroni adjustment was used to correct for multiple comparisons in view of 1,108 transcripts, 38,179 methylation probes, and 33,855 SNP/CN probes covering inflammation-related genes; therefore, *P* values <4.5×10^–5^, <1.3×10^–6^ and <1.48×10^–6^ were considered to be statistically significant, respectively, for transcripts, methylation and copy number. In this study, significant methylation probes covering the same gene locus all showed effect in the same direction, except for the 2 significant probes covering the *FYN* gene. Therefore, we calculated the mean methylation value of all significant probes across the same gene for further gene-level DNA methylation analysis. As to the *FYN* gene, the 2 significant probes were both taken forward for separate further analysis.

Because of tumor heterogeneity, the abundance and size of SCNA often vary across different patients with HCC or even across different tumor cells from the same tissue. Besides, although several analytical programs have been established to detect SCNAs based on the intensity of SNP array probes, the results obtained by using these programs are not all consistent. Therefore, in this study, we used a simple spanning strategy similar to that described by Abecasis [14] to detect structure variant using PCR. Under this strategy, only DNA regions that contain at least 2 adjacent SNP/CN probes showing significant difference between tumor and paired normal tissue were considered as SCNA markers. For significant probes exhibiting changes in the same direction at one gene locus, gene level mean LRR value across the significant probes was used for further analysis. For probes exhibiting changes in opposite directions at the same gene locus, mean LRR values across the significant probes were separately calculated for each direction and carried forward for further analysis. Spearman correlation coefficient was used to test the correlation between gene expression and DNA methylation or copy numbers for each gene.

### Validation using public dataset

Public datasets, GSE14520 and GSE25097, or GSE37988 and GSE54503 datasets, together with our data, were used to identify the overlapping genes reported by array-based gene expression or DNA methylation. The processed datasets of GSE14520, GSE25097, GSE37988 and GSE54503 were extracted from GEO database (http://www.ncbi.nlm.nih.gov/geo/), and analyzed by GEO2R software. Student's *t*-test was used to examine the difference in aberrant expression or DNA methylation changes of inflammation-related genes in HCCs with Bonferroni corrected significance threshold. In addition, we validated the SCNA results in the GSE38323 dataset, and validated the correlation between gene expression and SCNA in the published GSE28127 dataset [15,16].

### Validation in the independent sample

An independent sample, consisting of 47 surgically removed HCC and paired non-tumor specimens, was used to further validate our findings from the discovery stage.

Total RNA isolated from each tissue specimen was converted to cDNA using oligo(dT)15 primer and SuperScriptII (Invitrogen, Grand Island, NY). mRNA levels were measured by quantitative real-time PCR (RT-PCR) on an ABI Prism 7900 sequence detection system (Applied Biosystems, Foster City, CA) using the SYBR Green method. mRNA levels of the candidate genes were calculated relative to expression of *GAPDH*.

Methylation profile was determined in genomic DNA isolated from each tissue specimen. Primers targeting the promoter regions or CpG islands of the candidate inflammation-related genes were designed as described by Wojdacz et al. [17]. Tissue DNA samples, commercial fully methylated DNA and unmethylated DNA (Zymo Research) were simultaneously converted by bisulfite. A series of methylation dilution standards of 100%, 75%, 50%, 25%, 10%, 5% and 0% were prepared to depict methylation standard curve by mixing methylated DNA and unmethylated DNA. RT-PCR and methylation-sensitive high resolution melting (MS-HRM) analysis were carried out on an ABI Prism 7900 sequence detection system.

RT- PCR based on SYBR Green method was used to examine copy numbers of the candidate genes in each genomic DNA sample, with the *LINE-1* gene, the most abundant retrotransposon in the human genome as the reference [18]. Copy numbers of the candidate genes were calculated relative to those of *LINE-1* in each sample.

Paired student's *t*-test was used to examine the differences in gene expression, DNA methylation and copy numbers, at a false discovery rate (FDR) of 0.05. All the primers used in the validation are shown in S2 Table.

### Gene network construction

Inflammation-related genes with expression changes tallying with DNA methylation changes or copy number changes were selected to construct gene networks using the MetaCore database and software (GeneGo, Inc.; http://thomsonreuters.com/metacore/).

## Results

### Identification of aberrant expression, methylation and SCNA in inflammation-related genes

**Fig 1:**
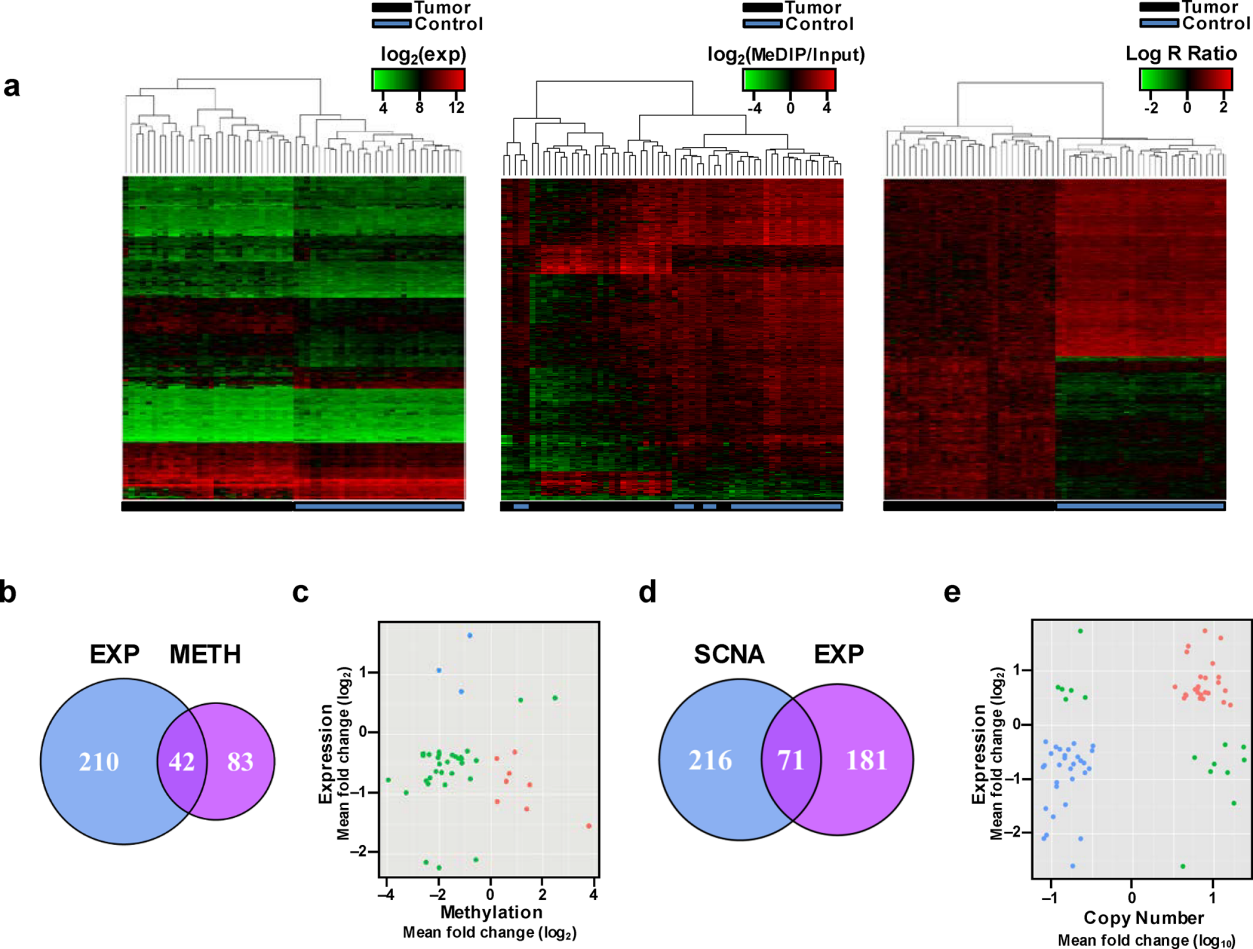
Identification of inflammation-related genes exhibiting coordinative changes between mRNA expression and DNA methylation or SCNA in HBV-related HCCs. (a) Hierarchical clustering with 260 significant transcripts corresponding 252 genes (*left*), 464 significant methylation probes covering 125 genes (*middle*), or 1,246 significant SNP/CN probes covering 287 genes (*right*) across HCCs (Tumor) and paired non-tumor tissues (Control). (b) Venn diagram showing 42 overlapping genes identified by the analysis of both mRNA expression and DNA methylation. EXP, genes with aberrant expression; METH, genes with aberrant DNA methylation. (c) Starburst plot of 42 overlapping genes identified by the analysis of both gene expression and DNA methylation. Red, blue and green dots indicate the genes hypermethylated and down-regulated, the genes hypomethylated and up-regulated and the genes having their expression not associated with DNA methylation, respectively. (d) Venn diagram showing 71 overlapping genes identified by the analysis of both mRNA expression and SCNA. EXP, genes with aberrant expression; SCNA, genes with copy number aberration. (e) Starburst plot of 71 overlapping genes identified by the analysis of both mRNA expression and SCNA. Red, blue and green dots indicate the genes with copy number gain and up-regulated expression, the genes with copy number deletion and down-regulated expression and the genes having inverse relationship between SCNA and mRNA expression, respectively.

We first obtained the profiles of mRNA expression, DNA methylation and copy number changes of all inflammation-related genes utilizing the array-based techniques. Overall, we identified 260 transcripts covering 252 inflammation-related genes that exhibited substantially aberrant expression in HCCs. Among them, 121 transcripts (114 genes) were up-regulated while 139 (138 genes) were down-regulated (S3 Table). We then performed exploratory hierarchical clustering of the 260 transcripts and found that the expression profiles in tumors and adjacent non-tumor tissues created unequivocally separate clusters (Fig 1A, ***left***). We found 71 probes residing in 39 inflammation-related gene loci were hypermethylated in HCCs, 391 probes in 85 genes hypomethylated. In addition, there were two differentially methylated probes in the *FYN* gene with one hypermethylated and the other hypomethylated (S4 Table). Hierarchical clustering resulted in overall clear distinction between tumor and adjacent non-tumor tissues, except for 3 samples (Fig 1A, ***middle***). As to copy number profiles, we identified 131 inflammation-related genes (consistent evidence from 512 probes) showing significant copy number gain and 141 genes (consistent evidence from 617 probes) showing significant copy number loss in HCC tumor tissues. In addition, 117 probes showed significant copy number difference between tumor and non-tumor tissues, exhibiting effects in both directions across 15 genes (S5 Table). Hierarchical clustering based on copy number resulted in clear distinction between tumor and non-tumor tissues (Fig 1A, ***right*)**.

### Contribution of methylation and SCNA to aberrant expression of inflammation-related genes

**Fig 2:**
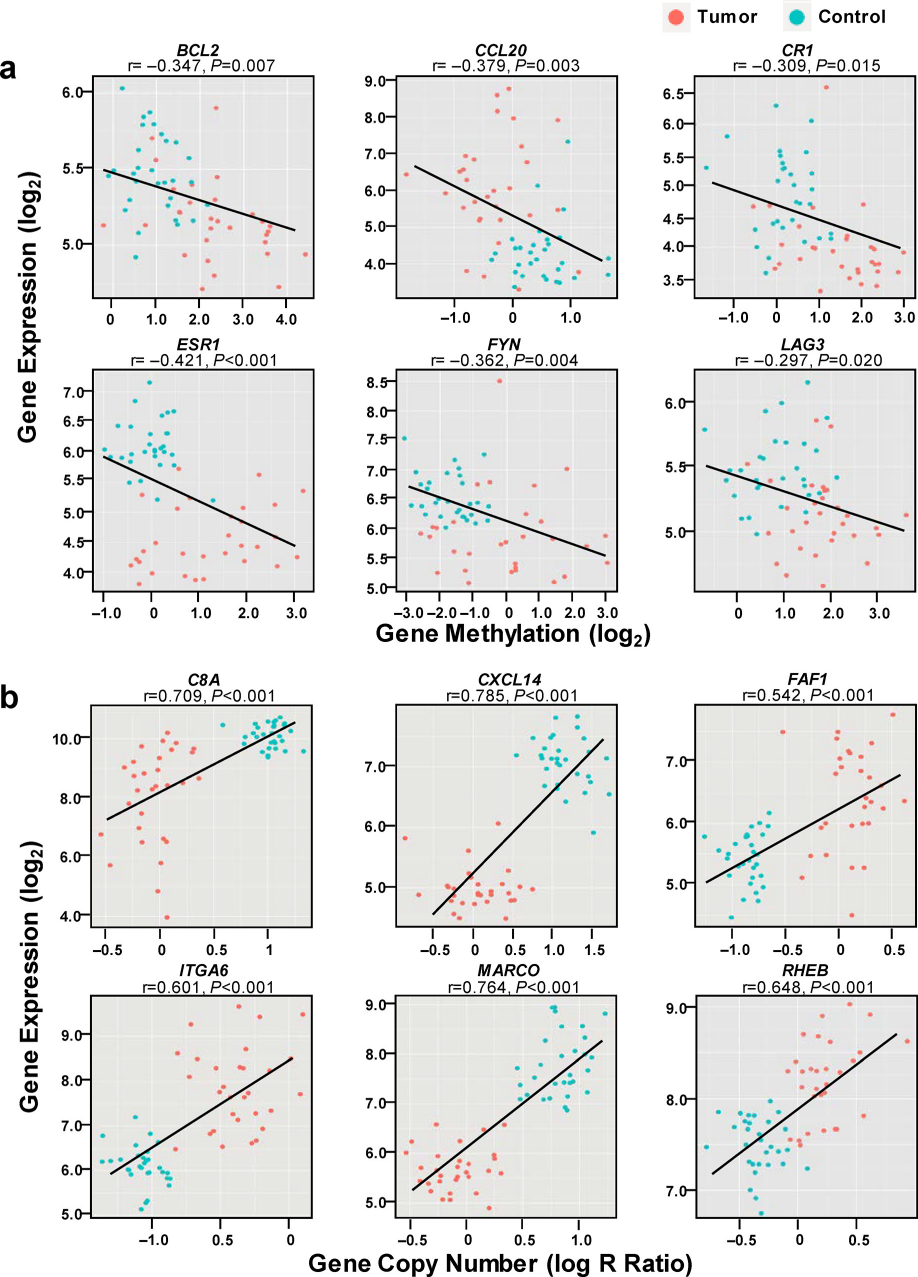
Relationship between mRNA expression and DNA methylation (a) or SCNA (b) of the randomly selected inflammation-related genes. Tumor, HCCs; Control, paired no-tumor tissues.

To investigate the contributions of DNA methylation and SCNA to the aberrant expression of inflammation-related genes, we integrated the expression profiles and DNA methylation profiles or copy number profiles obtained from tumors and non-tumor tissues. We found that 42 genes with aberrant expression had aberrant DNA methylation in HCCs compared with paired non-tumor tissues. Among them, only 8 genes with substantial down-regulation had DNA hypermethylation and 3 genes with substantial over-expression had DNA hypomethylation (Fig 1B and 1C), indicating a possible minor role (<5% of total) of DNA methylation in the expression regulation of these inflammation-related genes. We observed essential segregation of the expression levels and DNA methylation values in tumor and non-tumor tissues (Fig 1C). For the correlation between gene expression and SCNA, we found 56 genes with aberrant expression had concomitant SCNA in HCCs. Of these 56 genes, 26 with over-expression had copy number gain, while 30 with down-regulation had DNA deletion; SCNA can explain only one-fifth of aberrant expression of inflammation-related genes in HCCs (Fig 1D and 1E). The essential segregation of the gene expression levels and DNA copy number values is shown in Fig 1E. We found a negative correlation between the expression levels and DNA methylation for 11 genes (Fig 2A and S6 Table) and a positive correlation between the expression levels and copy numbers for 56 genes (Fig 2B and S7 Table). Interestingly, not only aberrant DNA methylation but also SCNA contribute to the aberrant expression of *BCL2*, *ESR1*, *FYN*, *PRKCB*, and *PTPN13* in HCCs.

### Validation of methylation and SCNA relevant to aberrant inflammation-related gene expression

**Fig 3:**
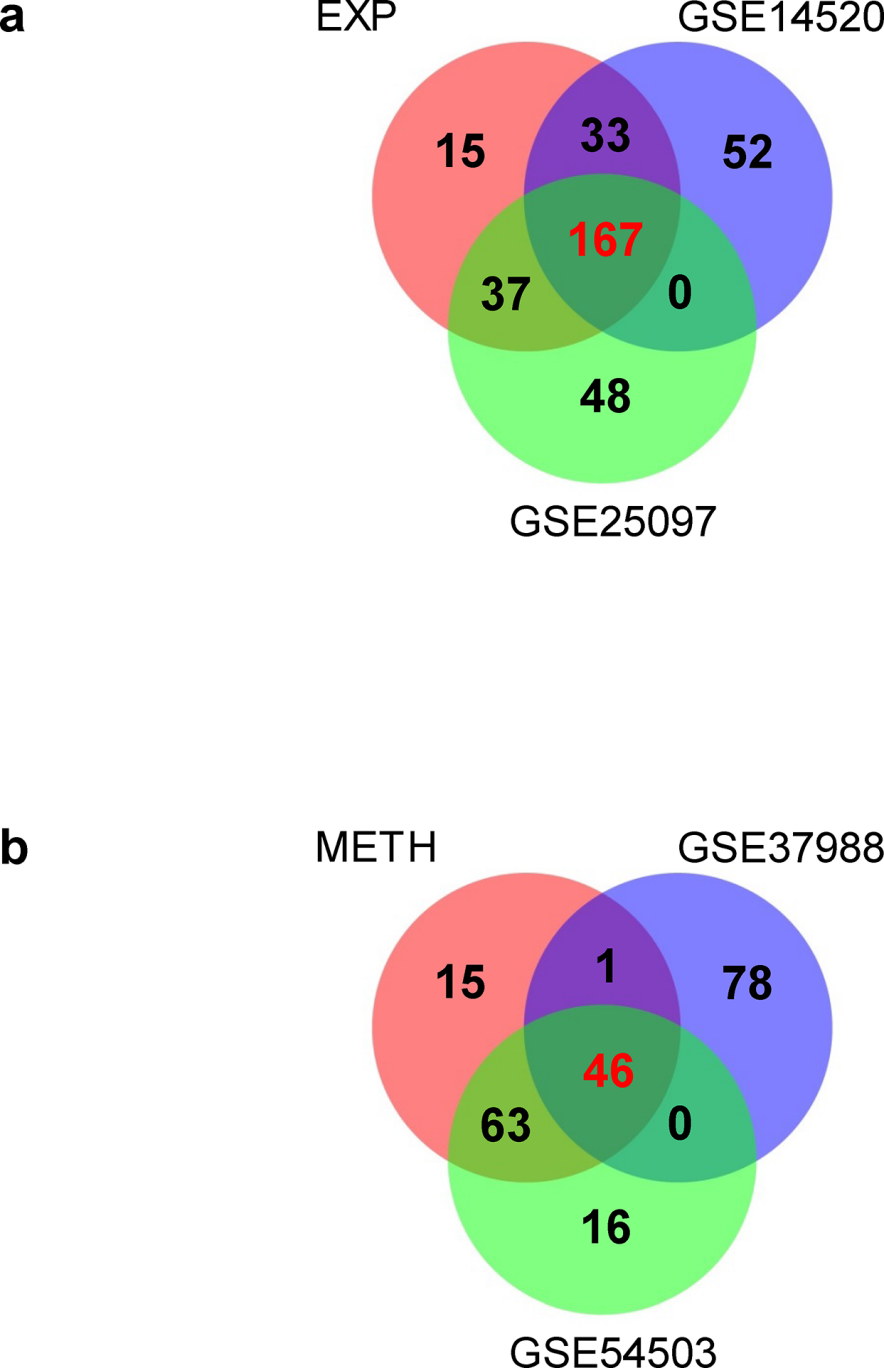
Venn diagram of the overlapping inflammation-related genes having significant mRNA expression (a), or DNA methylation (b) in HBV-related HCCs in our study with public datasets. EXP, genes with aberrant expression; METH, genes with aberrant methylation.

There are currently no published integrative analyses on the relationship between aberrant expression of inflammation-related genes and DNA methylation or SCNA in HCCs. To fill in this gap, we investigated the overlapping genes with aberrant expression and aberrant DNA methylation or SCNA in our dataset and the public datasets. Of the 252 aberrantly expressed genes found in our study, 200 (79.4%) and 204 (81.0%) were verified in the GSE14520 (S8 Table) and GSE25097 (S9 Table) dataset, which totaled up to 237 (94.1%) in at least one dataset (Fig 3A). As to the 125 aberrantly methylated genes identified in our study, 110 (88.0%) and 47 (37.6%) were also found to be aberrantly methylated in the GSE54503 (S10 Table) and GSE37988 (S11 Table) dataset, respectively, totaling up to 88.8% in at least one dataset (Fig 3B). We compared SCNA identified in our study with that in the GSE38323 dataset, and found that among the 287 genes with SCNA, 68 (23.7%) were also reported in the public dataset. In addition, as to the 56 genes with aberrant expression and concomitant SCNA identified in our study, 17 (30.4%) were found in GSE28127.

**Fig 4:**
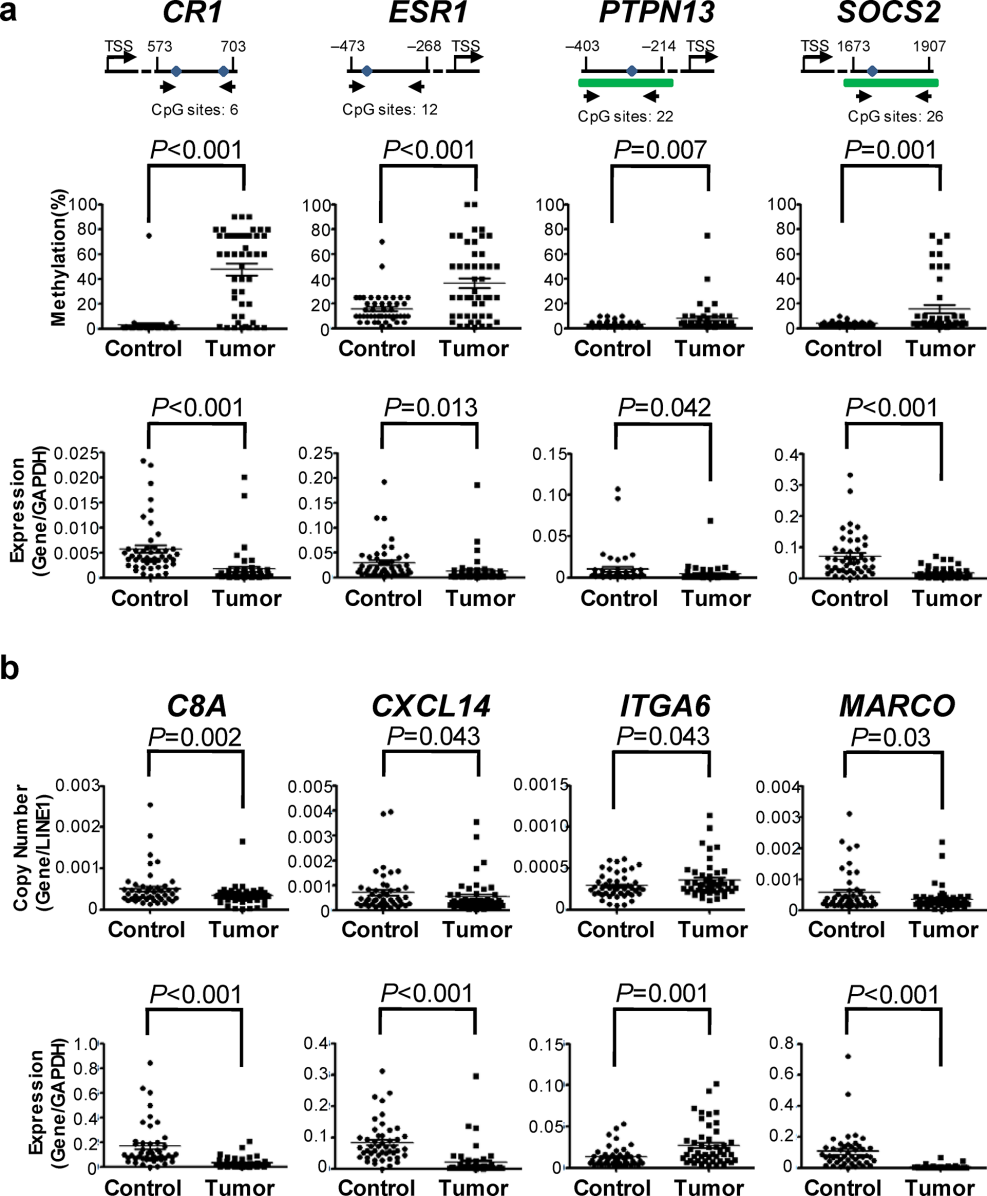
Validation of mRNA expression, DNA methylation and copy number variation of the randomly selected inflammation-related genes in 47 samples. (a) Validations of mRNA expression and DNA methylation of *CR1*, *ESR1*, *PTPN13* and *SOCS2*. *TSS*, transcription start site; Tumor, HCCs; Control, paired non-tumor tissues. Arrows represent the validation primers. Blue dots represent the significant methylation probes designed in array. Green bars represent the CpG island. (b) Validation of gene expression and copy number variation of *C8A*, *CXCL14*, *ITGA6* and *MARCO*.

We next randomly selected 8 genes aberrantly expressed in HCCs that had aberrant DNA methylation (*CR1*, *ESR1*, *PTPN13*, and *SOCS2*) or SCNA (*C8A*, *CXCL14*, *ITGA6* and *MARCO*) to validate the array-based results in an independent sample set consisting 47 HCCs and paired non-tumor specimens. The array-based analysis in our discovery stage showed that for *PTPN13* and *SOCS2*, the methylation sites are located in the CpG islands but for *CR1* and *ESR1* the methylation sites are located in the intronic and promoter regions. MS-HRM was used to measure methylation at these 4 genes in the validation sample set and standard curves are shown in S1 Fig. We found substantial changes of both DNA methylation and mRNA expression of *CR1*, *ESR1*, *PTPN13*, and *SOCS2* in HCCs compared with paired non-tumor tissues (Fig 4A), which is consistent with the results obtained by analyzing the array data in our discovery sample. Similarly, we were able to validate the association between SCNA and gene expression of *C8A*, *CXCL14*, *ITGA6* and *MARCO* (Fig 4B).These validation experiments indicate that the results produced by array-based analysis are reliable.

### Functional network construction

**Fig 5:**
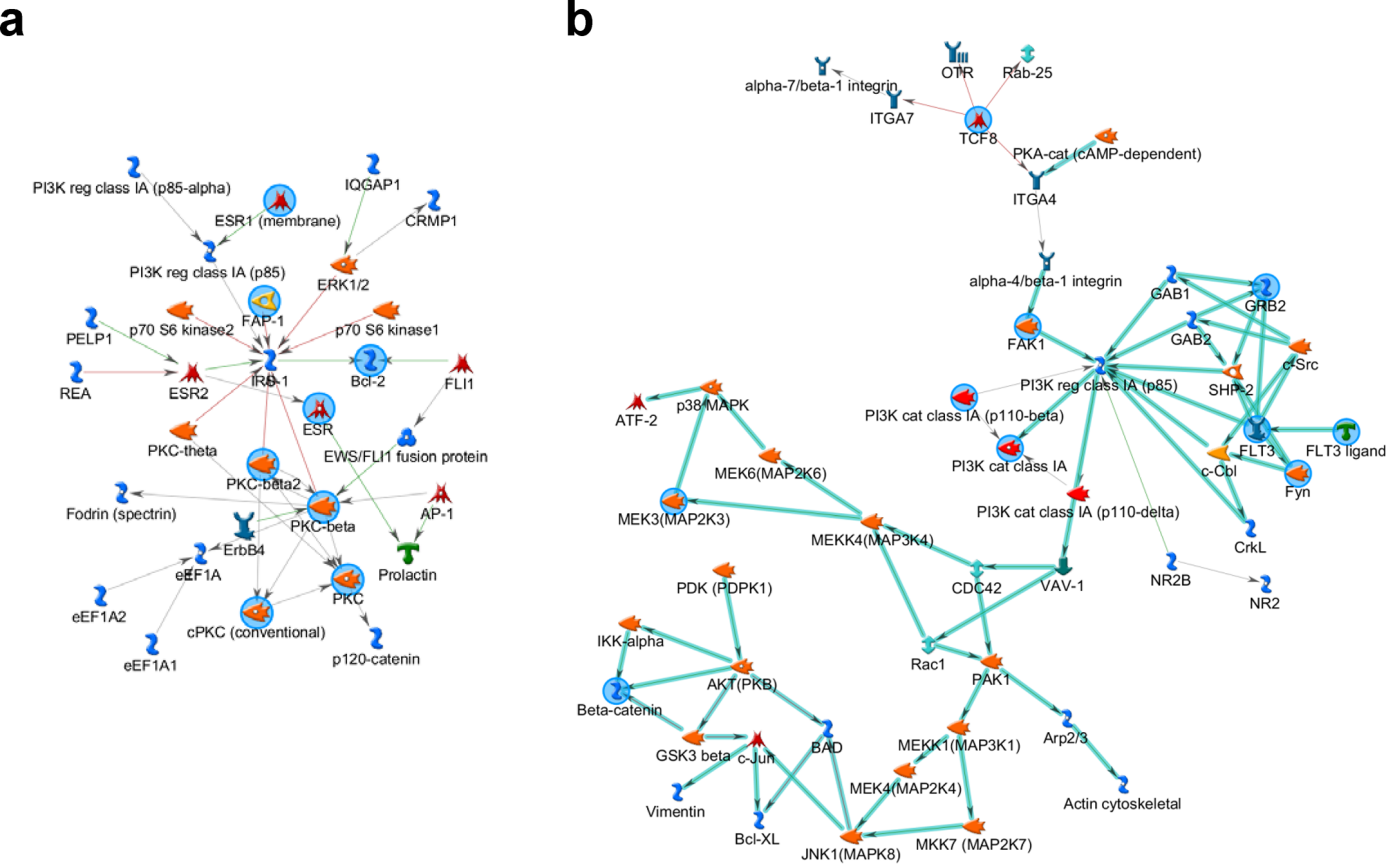
The functional network of top inflammation-related genes created by integrative analysis of mRNA expression associated with aberrant DNA methylation (a) and SCNA (b) in HBV-related HCCs. Blue circles represent the key nodes of the network.

We used MetaCore database and software to investigate the possible functional networks of inflammation-related genes suffering from aberrant DNA methylation (11 genes, S6 Table) or SCNA (56 genes, S7 Table) in HCCs. For the genes suffering from aberrant methylation, the top functional network (gScore = 76.97) involved in the following GO processes: response to alcohol, response to organic cyclic compound, immune response-regulating cell surface receptor signaling pathway, response to steroid hormone stimulus and immune response-regulating signaling pathway, using PKC-β, Bcl-2, FAP-1, ESR and PKC as key nodes (Fig 5A and S12 Table). For the genes suffering from SCNA, the top functional network (gScore = 271.81) involved in the GO processes of immune response-regulating signaling pathway, Fc receptor signaling pathway, innate immune response, regulation of immune system process and regulation of immune response, using FLT3, GRB2, TCF8, β-catenin, and Fyn as key nodes (Fig 5B and S13 Table). Noticeably, the functional network of the genes suffering from aberrant methylation is distinct from that of the genes suffering from SCNA, suggesting that methylation and SCNA contribute to different inflammation processes which may have the different regulatory mechanism (S2 Fig).

## Discussion

It is well known that chronic inflammation induced by HBV plays a pivotal role in the development of HCC [19, 20]. Many efforts have been made to investigate the aberrant expression and the regulatory mechanisms of inflammation-related genes in HBV-related HCC, but most have focused on a single or few genes. In this study, we performed a two-stage analysis to systematically investigate the contributions of aberrant DNA methylation and SCNA to the aberrant expression of genes in the whole inflammation system.

We identified 252 differentially expressed, 125 aberrantly methylated and 287 copy number changed inflammation-related genes in HBV-related HCC, which were validated in several published datasets. Except for the GSE54503 dataset, all the other five referred datasets were obtained from Chinese patients with HCC, which may efficiently reduce ethnic bias for comparison analysis. Most of the differentially expressed genes (94.1%) and aberrantly methylated genes (88.8%) were found in at least one referred dataset, indicating consistent results among these studies and ours. However, only 23.7% of copy number-changed genes identified in our study were also identified in the published report of GSE38323, probably due to different array platforms and methods used to detect SCNA across studies.

The most significant result in the current study is that we integrated the expression profile with DNA methylation or SCNA profiles to investigate to what extent the aberrant expression of these inflammation genes is attributable to methylation or SCNA. We found that among the 252 aberrantly expressed inflammation genes in HCC, only 11 genes whose aberrant expression can be explained by change in methylation; and only 56 genes whose aberrant expression can be explained by SCNA. It has been well known that aberrant DNA methylation is one of the most crucial hallmarks of carcinogenesis. However, our results revealed that aberrant DNA methylation might play a minor role (<5%) in the transcriptional regulation of inflammation-related genes in HBV-related HCCs. Previous studies have shown that aberrant DNA methylation of inflammation-related genes comes into existence in liver cirrhosis and some are maintained in HCCs [21, 22]. These findings are in agreement with the notion that both aberrant DNA methylation and inflammatory response occur as early events in HCC carcinogenesis. It has been reported that structure aberrations are persistently accumulated from early to late stage of HCCs [23]. Our results indicated that SCNA might explain one-fifth aberrant expression of inflammation genes in HBV-related HCCs. Taken together, aberrant DNA methylation and SCNAs in HBV-related HCCs contributed to less than 30% aberrant expression of inflammation-related genes. Furthermore, analysis of functional networks of aberrantly expressed genes showed that aberrant DNA methylation and SCNA brought about different inflammatory response pathways. All these findings suggested that the regulatory system of inflammation-related genes is complicated and meticulous. Other molecular mechanisms such as transcription factors, non-coding RNAs and mutations might be more relevant in terms of aberrant expression of inflammation-related genes in HCCs, which warrants future investigation.

Four genes (*CR1*, *ESR1*, *PTPN13*, and *SOCS2*) that showed aberrant expression associated with DNA methylation were randomly selected to verify in an additional sample set and proved to be consistent with array-based results. *CR1*, a negative regulator of the complement cascade, has been reported to be hypermethylated in acute lymphoblastic leukemia [24]. *ESR1*, a ligand-activated transcription factor, has been shown to be hypermethylated in the promoter in 83.3% of HCC samples [25]. *PTPN13* is a protein tyrosine phosphatase gene that is frequently down-regulated and hypermethylated in HCC cell lines [26]. *SOCS2*, encoding the suppressor of cytokine signaling protein, is frequently hypermethylated in primary ovarian cancer [27]. Similarly, the association between SCNA and aberrant expression of *C8A*, *CXCL14*, *ITGA6* and *MARCO* was also verified in the validation sample set. The *CXCL14* gene, whose copy number was significantly lower in HCCs compared with paired non-tumor tissues, encodes a chemokine that has been shown to play a pivotal role as a tumor suppressor in HCC [28]. However, the roles that *C8A*, *ITGA6* and *MARCO* genes play in the development of HCC remain unclear and further studies on these genes are under way.

We acknowledge some limitations of this study. Although tissue heterogeneity of the liver is much less than other tissues such as the lung and the breast, which provides good opportunity for analysis of gene expression in cancer tissue, the stromal content in clinical samples might also have potential to affect mRNA analysis of HCC. Besides, this study analyzed only the mRNA levels and it would be profited to analyze the protein level of some genes to confirm their mRNA levels.

In summary, our two-stage comprehensive study achieved at least two progresses: identification of HCC-specific DNA methylation, SCNA and mRNA expression profiles of inflammation-related genes and elucidation of the contribution of DNA methylation and SCNA to the aberrant expression of inflammation-related genes in HBV-related HCCs. These results partially answered the long-standing question, i.e., what mechanism contributes to the aberrant expression of inflammation-related genes that play important roles in the development of HBV-related HCCs.

## Supporting Information

S1 Fig Normalized HRM stand curves for *CR1*, *ESR1*, *PTPN13* and *SOCS2* genes. Curves with different colors represent standard temples with DNA different methylation ratios (as indicated).
(PDF)

S2 Fig Inflammation-related gene network. Network constructed by merging the top network of significant gene expression changes associated with inverse DNA methylation changes (blue), and that of significant gene expression changes associated with coordinative SCNAs (green).
(PDF)

S1 Table Clinical Characteristics of 30 HCC Patients with Array Data and 47 Patients with Validation Data. (DOC)

S2 Table Primers used in the Validation for Gene Expression, DNA Methylation, and SCNAs in 47 HCC Patients. (DOC)

S3 Table Inflammation-related Genes with Dysregulated Expression in HCC. (DOC)

S4 Table Inflammation-related Genes with Aberrant Methylation in HCC. (DOC)

S5 Table Inflammation-related genes with SCNAs in HCC. (DOC)

S6 Table Inflammation-related Genes with DNA Methylation Changes Associated with Inverse Expression Changes in HCC. (DOC)

S7 Table Inflammation-related Genes with SCNAs Associated with Expression Changes in HCC. (DOC)

S8 Table 200 Aberrantly Expressed Inflammation-related Genes Validated in GSE14520 Dataset. (DOC)

S9 Table 204 Aberrantly Expressed Inflammation-related Genes Validated in GSE25097 Dataset. (DOC)

S10 Table 110 Aberrantly Methylated Inflammation-related Genes Validated in GSE54503 Dataset. (DOC)

S11 Table 47 Aberrantly Methylated Inflammation-related Genes Validated in GSE37988 Dataset. (DOC)

S12 Table Networks Constructed by the Inflammation-related Genes with DNA Methylation Changes Associated with Inverse Expression Changes in HCC. (DOC)

S13 Table Networks Constructed by the Inflammation-related Genes with SCNAs Associated with Expression Changes in HCC. (DOC)
