# Supplementary figures and images for: Relatively Small Contribution of Methylation and Genomic Copy Number Aberration to the Aberrant Expression of Inflammation-Related Genes in HBV-Related Hepatocellular Carcinoma
Source: PLoS One. 2015 May 12;10(5):e0126836. doi: 10.1371/journal.pone.0126836 (PMC4429029; doi:10.1371/journal.pone.0126836)

S1 Fig.

*CR1*

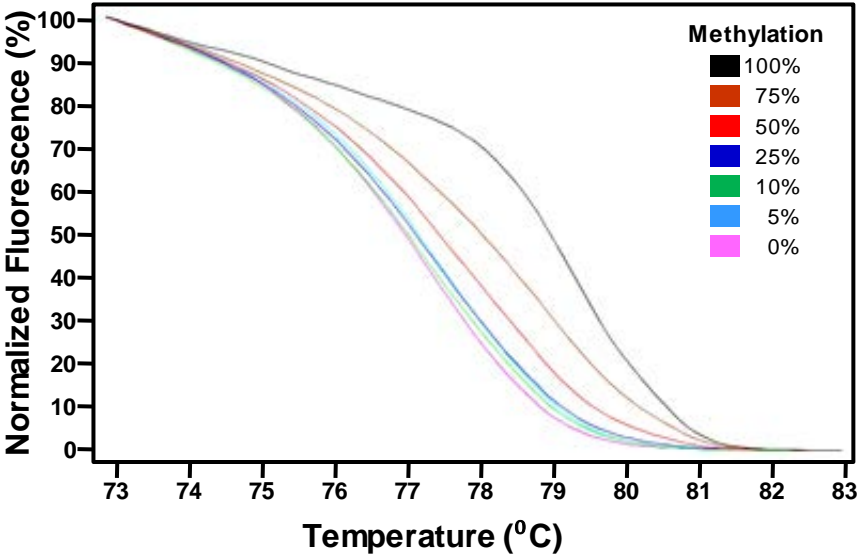

*ESR1*

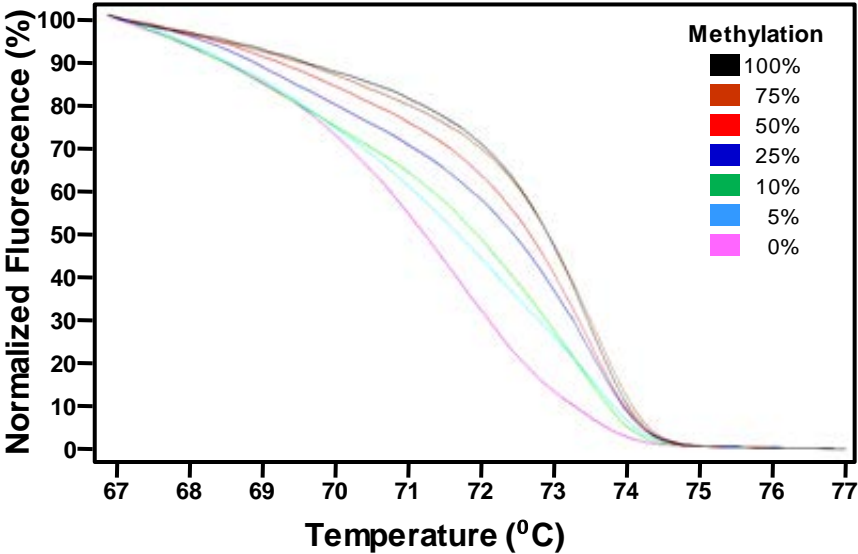

*PTPN13*

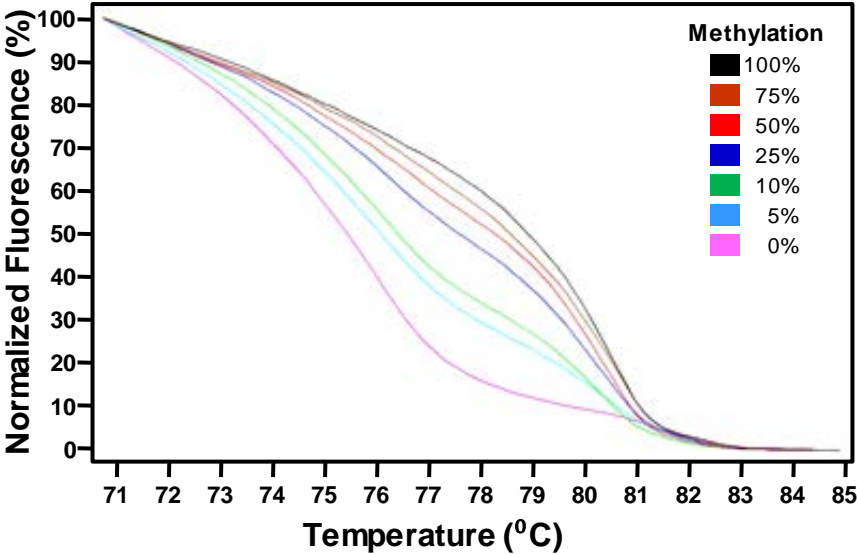

*SOCS2*

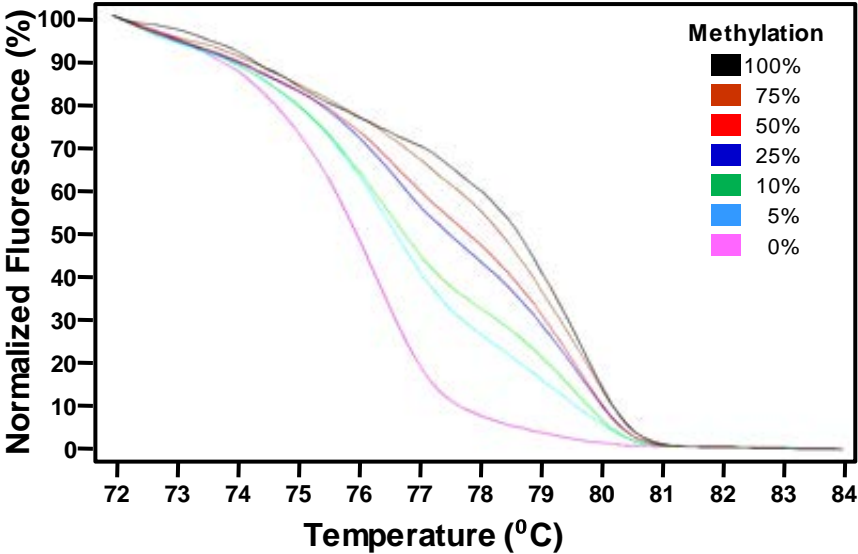

Supplement: S1 Fig — Curves with different colors represent standard temples with DNA different methylation ratios (as indicated). (PDF) [file pone.0126836.s001.pdf]

## S2 Fig.

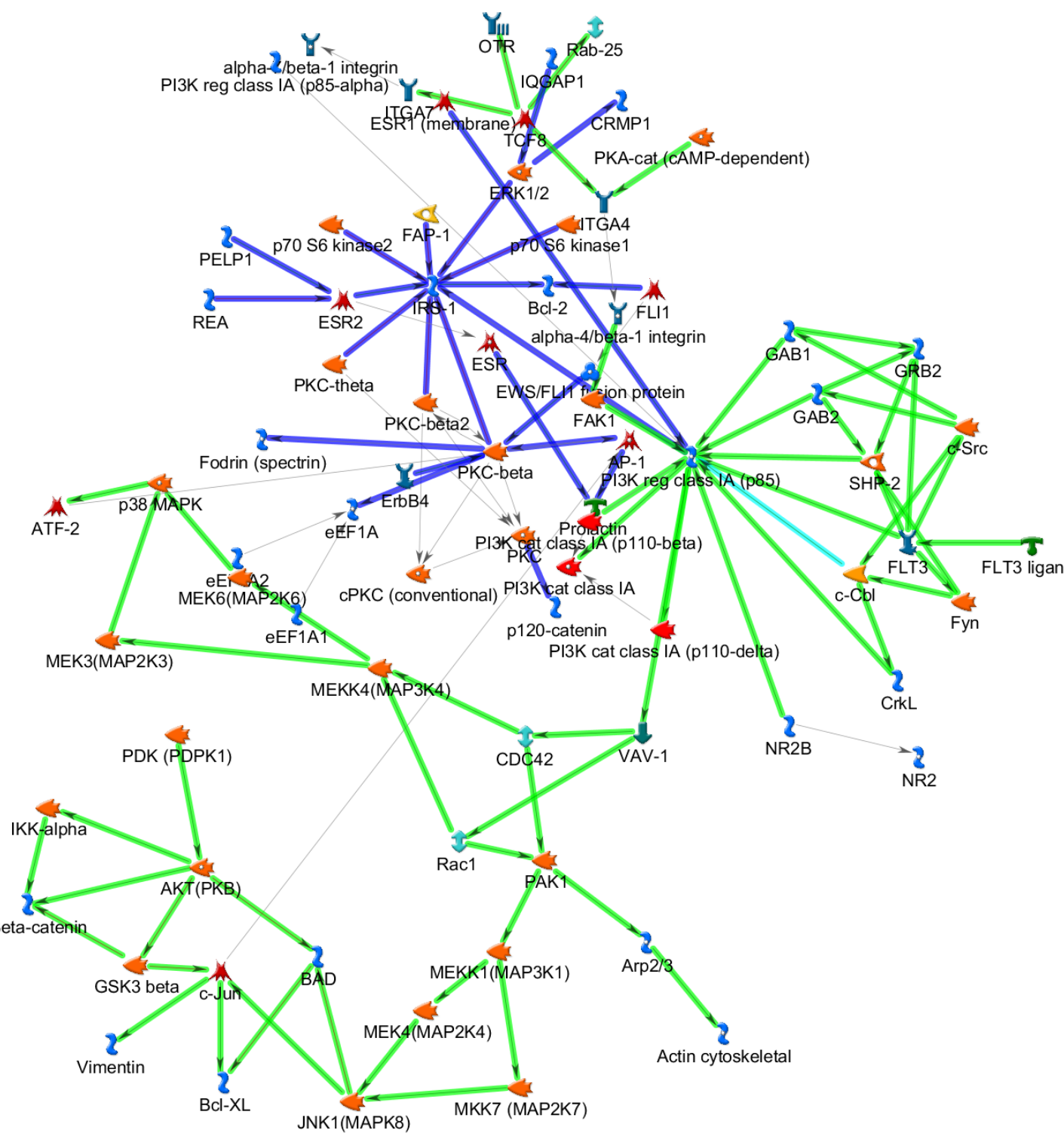

Supplement: S2 Fig — Network constructed by merging the top network of significant gene expression changes associated with inverse DNA methylation changes (blue), and that of significant gene expression changes associated with coordinative SCNAs (green). (PDF) [file pone.0126836.s002.pdf]
