# Supplementary material for: Relatively Small Contribution of Methylation and Genomic Copy Number Aberration to the Aberrant Expression of Inflammation-Related Genes in HBV-Related Hepatocellular Carcinoma
Source: PLoS One. 2015 May 12;10(5):e0126836. doi: 10.1371/journal.pone.0126836 (PMC4429029; doi:10.1371/journal.pone.0126836)
Supplement: S1 Table — (DOC) [file pone.0126836.s003.doc]

| **Characteristics** | **Patients with Array Data** |  | **Patients with Validation Data** |
| --- | --- | --- | --- |
|  | **No. (%)** |  | **No. (%)** |
| Age* | 54.7±11.0 |  | 54.2±11.1 |
| Sex |  |  |  |
| Male | 28 (93.3) |  | 35 (74.5) |
| Female | 2 (6.7) |  | 12 (25.5) |
| Smoking status |  |  |  |
| Smoker | 18 (60.0) |  | 17 (36.2) |
| Nonsmoker | 12 (40.0) |  | 29 (61.7) |
| N/A | 0 |  | 1 (2.1) |
| Alcohol drinking |  |  |  |
| Drinker | 12 (40.0) |  | 9 (19.1) |
| Nondrinker | 18 (60.0) |  | 37 (78.8) |
| N/A | 0 |  | 1 (2.1) |
| HBV infection | 30 (100.0) |  | 47(100.0) |
| Cirrhosis |  |  |  |
| Present | 26 (86.7) |  | 43 (91.5) |
| Absent | 3 (10.0) |  | 3 (6.4) |
| N/A | 1 (3.3) |  | 1(2.1) |
| BCLC stage |  |  |  |
| A | 13 (43.3) |  | 31 (66.0) |
| B | 14 (46.7) |  | 11 (23.4) |
| C | 3 (10.0) |  | 5 (10.6) |

**S1 Table. Clinical Characteristics of 30 HCC Patients with Array Data and 47 Patients with Validation Data**

*Mean±SD.

Abbreviations: N/A, Not available; BCLC, Barcelona Clinic Liver cancer classification.
