# Supplementary material for: Relatively Small Contribution of Methylation and Genomic Copy Number Aberration to the Aberrant Expression of Inflammation-Related Genes in HBV-Related Hepatocellular Carcinoma
Source: PLoS One. 2015 May 12;10(5):e0126836. doi: 10.1371/journal.pone.0126836 (PMC4429029; doi:10.1371/journal.pone.0126836)
Supplement: S2 Table — (DOC) [file pone.0126836.s004.doc]

**S2 Table. Primers used in the Validation for Gene Expression, DNA Methylation, and SCNAs in 47 HCC Patients**

| **Primers** | **Sequence (5’ to 3’)** | **Tm (⁰C)** | **Validation for** |
| --- | --- | --- | --- |
| CR1-EXP-F | CAAGTGTTGAAGACAACTGTAGACGAA | 60.0 | CR1 Expression |
| CR1-EXP-R | ACTGTGTATCTGTGTTTATATGCACCAT | 60.0 | CR1 Expression |
| ESR1-EXP-F | CTGCTGGCTACATCATCTCGG | 60.0 | ESR1 Expression |
| ESR1-EXP-R | GACTCGGTGGATATGGTCCTTCT | 60.0 | ESR1 Expression |
| PTPN13-EXP-F | TGGCTCTCCAGGCTGAGTATG | 60.0 | PTPN13 Expression |
| PTPN13-EXP-R | CGGGCAAATAGTGCTCCATT | 60.0 | PTPN13 Expression |
| SOCS2-EXP-F | TTAAAAGAGGCACCAGAAGGAACT | 60.0 | SOCS2 Expression |
| SOCS2-EXP-R | AGTCGATCAGATGAACCACACTGT | 60.0 | SOCS2 Expression |
| C8A-EXP-F | AATGTTGGTGGAGGTTTATCAGG | 60.0 | C8A Expression |
| C8A-EXP-R | CAGGAACGGTATGTAATGGTGC | 60.0 | C8A Expression |
| CXCL14-EXP-F | ACGGGTCCAAATGCAAGTG | 60.0 | CXCL14Expression |
| CXCL14-EXP-R | GGCTTCATTTCCAGCTTCTTCA | 60.0 | CXCL14 Expression |
| GAPDH-EXP-F | TTGGCCAGGGGTGCTAAG | 60.0 | Reference for Gene Expression |
| GAPDH-EXP-R | AGCCAAAAGGGTCATCATCTC | 60.0 | Reference for Gene Expression |
| ITGA6-EXP-F | TTTCCCTGAGAAACAGTTGAGTTGT | 60.0 | ITGA6 Expression |
| ITGA6-EXP-R | GGATTTCCGAGCTCACAGTCA | 60.0 | ITGA6 Expression |
| MARCO-EXP-F | GTGCATCGAGGCTGCAAGTC | 60.0 | MARCO Expression |
| MARCO-EXP-R | CGCCTTGTTCACCTTTGATTC | 60.0 | MARCO Expression |
| CR1-M-F | GGATAGAGAGCGAGGTTAGGGTTT | 63.5 | CR1 Methylation |
| CR1-M-R | CACAACACGAACTCTAACAACAATCC | 63.5 | CR1 Methylation |
| ESR1-M-F | ATTCGTTTTTCGCGTTTATTTTAAGTTT | 60.8 | ESR1Methylation |
| ESR1-M-R | TCCCGAACTCATATACATTACAAAAATAC | 60.8 | ESR1 Methylation |
| PTPN13-M-F | TTACGTTGAGGCGAGGGTGAT | 60.5 | PTPN13 Methylation |
| PTPN13-M-R | CCCCGCCGCCCTACTATAC | 60.5 | PTPN13 Methylation |
| SOCS2-M-F | CGGGGTCGAGGTGGGAAGT | 60.8 | SOCS2 Methylation |
| SOCS2-M-R | CGCGACACCGACTTACTAATTACAC | 60.8 | SOCS2 Methylation |
| C8A-CN-F | GCTAAACAGCCTACAATACAAGGACAG | 62.0 | C8A SCNA |
| C8A-CN-R | GGTGGGAGTTATTTACAACTGATGC | 62.0 | C8A SCNA |
| CXCL14-CN-F | TGGTGGTGATGCTGAAACGGA | 62.0 | CXCL14 SCNA |
| CXCL14-CN-R | GGGCTGCTCACTACATACAGGGTTA | 62.0 | CXCL14 SCNA |
| ITGA6-CN-F | CATACAAATGAAACGATCCACACAC | 62.0 | ITGA6 SCNA |
| ITGA6-CN-F | CTACTTTACCCAAGGCATTCTCAAC | 62.0 | ITGA6 SCNA |
| LINE1-CN-F | AAAGCCGCTCAACTACATGG | 62.0 | Reference for SCNAs |
| LINE1-CN-R | TGCTTTGAATGCGTCCCAGAG | 62.0 | Reference for SCNAs |
| MARCO-CN-F | GAGCAGCCACCCTTCAAGTATTCAG | 62.0 | MARCO SCNA |
| MARCO-CN-R | AGAGCCCTGGGTCTTAGCATTGAGT | 62.0 | MARCO SCNA |
