# Supplementary material for: Relatively Small Contribution of Methylation and Genomic Copy Number Aberration to the Aberrant Expression of Inflammation-Related Genes in HBV-Related Hepatocellular Carcinoma
Source: PLoS One. 2015 May 12;10(5):e0126836. doi: 10.1371/journal.pone.0126836 (PMC4429029; doi:10.1371/journal.pone.0126836)
Supplement: S3 Table — (DOC) [file pone.0126836.s005.doc]

**S3 Table. Inflammation-related Genes with Dysregulated Expression in HCC**

| **Symbol** | **Transcript ID*** | **Chr.** | **Start**† | **Stop**† | **FC** | **P value**‡ |
| --- | --- | --- | --- | --- | --- | --- |
| *ADAM17* | 8050190 | chr2 | 9546863 | 9613368 | 0.532023 | 3.84634E–05 |
| *ADCY1* | 8132667 | chr7 | 45580645 | 45729237 | –0.44142 | 1.6273E–05 |
| *ADCY5* | 8090070 | chr3 | 124486088 | 124650082 | –0.52072 | 5.27753E–08 |
| *ADCY6* | 7962842 | chr12 | 47446247 | 47469087 | 0.314944 | 4.3461E–05 |
| *AKR1C3* | 7925929 | chr10 | 5126567 | 5139878 | 1.461112 | 4.75361E–10 |
| *ALCAM* | 8081431 | chr3 | 106568879 | 106776709 | 0.639063 | 1.93342E–05 |
| *APAF1* | 7957759 | chr12 | 97563208 | 97653341 | 0.489051 | 3.02253E–07 |
| *ATF1* | 7955425 | chr12 | 49444127 | 49500327 | 0.605543 | 4.63152E–07 |
| *ATF2* | 8056909 | chr2 | 175647253 | 175741143 | 0.504905 | 3.94674E–08 |
| *B3GAT1* | 7952884 | chr11 | 133753611 | 133787022 | –0.39935 | 8.08981E–08 |
| *BAK1* | 8125766 | chr6 | 33648306 | 33655959 | 0.624969 | 1.71749E–05 |
| *BBC3* | 8037872 | chr19 | 52415923 | 52426291 | –0.35705 | 2.30584E–05 |
| *BCL2* | 8023646 | chr18 | 59136114 | 59137025 | –0.30302 | 4.40409E–05 |
| *BMPR1A* | 7952305 | chr10 | 88506375 | 88674924 | 0.67351 | 4.73056E–06 |
| *BMPR1A* | 7928855 | chr10 | 88506375 | 88674924 | 0.644845 | 1.18491E–05 |
| *C1QA* | 7898793 | chr1 | 22836686 | 22838686 | –0.70374 | 3.00313E–05 |
| *C1QC* | 7898799 | chr1 | 22842733 | 22847189 | –0.79173 | 3.34869E–05 |
| *C1R* | 7960744 | chr12 | 7057770 | 7136184 | –1.24337 | 9.99485E–08 |
| *C6* | 8111864 | chr5 | 41178092 | 41249369 | –2.18531 | 1.4086E–08 |
| *C7* | 8105084 | chr5 | 40945355 | 41018796 | –3.82467 | 5.35579E–14 |
| *C8A* | 7901732 | chr1 | 57093066 | 57156482 | –2.0264 | 2.04834E–08 |
| *C8B* | 7916526 | chr1 | 57167472 | 57204276 | –1.46448 | 2.16786E–06 |
| *C9* | 8111757 | chr5 | 39320764 | 39400412 | –3.71283 | 5.12929E–09 |
| *CAMK4* | 8107307 | chr5 | 110587980 | 110848645 | –0.73093 | 2.10383E–08 |
| *CAPN1* | 7941179 | chr11 | 64705918 | 64736052 | 0.42467 | 2.15479E–05 |
| *CAPN2* | 7909967 | chr1 | 221966823 | 222030341 | 0.761698 | 9.9506E–07 |
| *CAPNS1* | 8028172 | chr19 | 41322757 | 41333094 | 0.584584 | 4.87417E–07 |
| *CASP2* | 8136869 | chr7 | 142695523 | 142714906 | 0.676722 | 1.05957E–09 |
| *CASP8AP2* | 8121118 | chr6 | 90596378 | 90640875 | 0.82531 | 6.92934E–08 |
| *CAT* | 7939298 | chr11 | 34417053 | 34450179 | –1.08783 | 9.62177E–07 |
| *CCL15* | 8014349 | chr17 | 31348776 | 31353125 | –0.94894 | 2.6567E–07 |
| *CCL19* | 8160879 | chr9 | 34679566 | 34681274 | –1.53527 | 1.45227E–08 |
| *CCL20* | 8048864 | chr2 | 228386813 | 228390494 | 1.730508 | 2.67385E–06 |
| *CCL21* | 8160889 | chr9 | 34699001 | 34700147 | –1.37975 | 5.98483E–10 |
| *CCR1* | 8086600 | chr3 | 46218203 | 46224836 | –0.59383 | 1.06131E–05 |
| *CD14* | 8114612 | chr5 | 139991504 | 139992956 | –1.40883 | 2.7156E–07 |
| *CD160* | 7919243 | chr1 | 144407154 | 144426922 | –0.43316 | 2.8294E–06 |
| *CD1C* | 7906348 | chr1 | 156526427 | 156530028 | –0.47736 | 4.56353E–06 |
| *CD1D* | 7906330 | chr1 | 156416360 | 156421309 | –0.58783 | 4.04607E–08 |
| *CD1E* | 7906355 | chr1 | 156590402 | 156593009 | –0.48127 | 3.64512E–05 |
| *CD207* | 8052916 | chr2 | 70910855 | 70916461 | –0.34925 | 3.18167E–06 |
| *CD226* | 8023757 | chr18 | 65681174 | 65775140 | –0.90743 | 4.91781E–08 |
| *CD244* | 7921677 | chr1 | 159066574 | 159099269 | –0.87138 | 1.68491E–08 |
| *CD247* | 7922040 | chr1 | 165666509 | 165754450 | –0.36911 | 5.95205E–06 |
| *CD300A* | 8009653 | chr17 | 69974116 | 69992526 | –0.52722 | 4.01548E–07 |
| *CD33* | 8030804 | chr19 | 56420174 | 56435084 | –0.30141 | 2.22421E–06 |
| *CD34* | 7923978 | chr1 | 206126506 | 206151138 | 1.286975 | 1.35716E–11 |
| *CD4* | 7953428 | chr12 | 6769004 | 6800235 | –0.81143 | 9.71427E–08 |
| *CD46* | 7909400 | chr1 | 205992024 | 206035481 | 0.599898 | 6.87296E–08 |
| *CDK1* | 7927710 | chr10 | 62208218 | 62224616 | 1.740277 | 1.66441E–11 |
| *CFD* | 8024062 | chr19 | 810664 | 814608 | –0.55007 | 1.48822E–07 |
| *CFI* | 8102328 | chr4 | 110881300 | 110942590 | –1.20406 | 2.07103E–07 |
| *CFP* | 8172333 | chrX | 47368569 | 47374648 | –1.55777 | 3.68506E–16 |
| *CHUK* | 7935707 | chr10 | 101938114 | 101979334 | 0.665382 | 5.39298E–07 |
| *CR1* | 7909371 | chr1 | 205736124 | 205880609 | –0.85364 | 3.17032E–06 |
| *CREB1* | 8047839 | chr2 | 208102930 | 208171813 | 0.485513 | 1.02096E–08 |
| *CSF1R* | 8115076 | chr5 | 149413050 | 149473128 | –0.71365 | 4.12015E–07 |
| *CSF3R* | 7914950 | chr1 | 36704230 | 36721096 | –0.58213 | 8.9002E–06 |
| *CSNK2A1* | 8064351 | chr20 | 411337 | 472482 | 0.643747 | 3.19448E–09 |
| *CSNK2B* | 8118218 | chr6 | 31741635 | 31745822 | 0.882453 | 1.30735E–09 |
| *CSNK2B* | 8178050 | chr6 | 31741635 | 31745822 | 0.929641 | 1.37556E–09 |
| *CSNK2B* | 8179298 | chr6 | 31741635 | 31745822 | 0.87526 | 1.60571E–09 |
| *CTNNB1* | 8079021 | chr3 | 41216015 | 41256938 | 0.66611 | 1.16354E–06 |
| *CTTN* | 7942204 | chr11 | 69922291 | 69960337 | 0.66253 | 2.25842E–06 |
| *CXCL1* | 8095697 | chr4 | 74953972 | 74955817 | –0.729 | 2.03272E–06 |
| *CXCL12* | 7933194 | chr10 | 44185612 | 44200551 | –2.59707 | 7.96015E–13 |
| *CXCL14* | 8114249 | chr5 | 134934273 | 134942868 | –2.08826 | 2.09839E–19 |
| *CXCL2* | 8100994 | chr4 | 75181619 | 75183756 | –1.83776 | 8.31064E–12 |
| *CXCR1* | 8058905 | chr2 | 218735813 | 218739961 | –0.42176 | 2.06981E–05 |
| *CXCR2* | 8048227 | chr2 | 218698981 | 218710221 | –0.36856 | 2.06654E–05 |
| *DAP3* | 7906041 | chr1 | 153925497 | 153974941 | 0.895661 | 6.25869E–12 |
| *DAXX* | 8180179 | chr6 | 33394380 | 33398682 | 0.381272 | 5.41773E–06 |
| *DAXX* | 8125734 | chr6 | 33394380 | 33398682 | 0.374045 | 6.3263E–06 |
| *DAXX* | 8178991 | chr6 | 33394380 | 33398682 | 0.348587 | 2.3439E–05 |
| *DFFA* | 7912303 | chr1 | 10443191 | 10455200 | 0.514885 | 1.56998E–06 |
| *DIABLO* | 7967230 | chr12 | 121258162 | 121276552 | 0.389098 | 2.75042E–05 |
| *DPEP2* | 8002194 | chr16 | 66578800 | 66590857 | –0.32633 | 7.36436E–06 |
| *DUSP1* | 8115831 | chr5 | 172127706 | 172130809 | –0.96459 | 7.86834E–06 |
| *EDNRB* | 7972157 | chr13 | 77368543 | 77447665 | –0.99109 | 4.67411E–08 |
| *EIF2AK2* | 8051501 | chr2 | 37187202 | 37237572 | 0.695925 | 1.2817E–08 |
| *ELANE* | 8024056 | chr19 | 803290 | 807244 | –0.34676 | 9.99493E–06 |
| *ENAH* | 7924619 | chr1 | 225501045 | 225667356 | 1.610688 | 3.95318E–12 |
| *ENG* | 8164269 | chr9 | 129617116 | 129656805 | –0.71982 | 3.39931E–07 |
| *ESR1* | 8122843 | chr6 | 152170378 | 152466099 | –1.53614 | 1.46569E–12 |
| *FAF1* | 7915995 | chr1 | 50679522 | 51198524 | 0.497572 | 7.7233E–07 |
| *FAS* | 7929032 | chr10 | 90740267 | 90765521 | –0.63887 | 1.37187E–06 |
| *FASLG* | 7907430 | chr1 | 170894807 | 170902633 | –0.32497 | 6.30063E–07 |
| *FCGR2B* | 7906777 | chr1 | 159899563 | 159914575 | –1.43776 | 2.46083E–09 |
| *FCGR3A* | 7921873 | chr1 | 159778176 | 159786442 | –0.89681 | 1.1475E–07 |
| *FCGR3A* | 7921868 | chr1 | 159778176 | 159786442 | –0.64478 | 9.73453E–06 |
| *FLT3* | 7970737 | chr13 | 27475752 | 27572703 | –0.3362 | 3.76737E–07 |
| *FOS* | 7975779 | chr14 | 74815283 | 74818666 | –2.41109 | 1.37724E–10 |
| *FOSL1* | 7949532 | chr11 | 65416267 | 65424573 | –0.30243 | 3.9612E–05 |
| *FOXO1* | 7971177 | chr13 | 40027816 | 40138734 | –0.46756 | 4.80903E–06 |
| *FPR2* | 8030860 | chr19 | 56958453 | 56965589 | –0.47772 | 2.03638E–05 |
| *FYN* | 8128956 | chr6 | 112089179 | 112301320 | –0.65269 | 3.95565E–05 |
| *GMEB1* | 7899504 | chr1 | 28867830 | 28913972 | 0.593293 | 1.50896E–07 |
| *GRB2* | 8018364 | chr17 | 70825752 | 70913384 | 0.688746 | 1.61982E–08 |
| *HDAC1* | 7899774 | chr1 | 32530294 | 32571811 | 0.845587 | 1.0211E–09 |
| *HMGN1* | 8176191 | chr21 | 39636111 | 39642917 | 0.542229 | 2.4771E–08 |
| *HMGN1* | 8070389 | chr21 | 39636111 | 39642917 | 0.357286 | 1.52402E–05 |
| *HMMR* | 8109712 | chr5 | 162820240 | 162851523 | 1.476708 | 4.63491E–10 |
| *HRH2* | 8110106 | chr5 | 175042317 | 175044162 | –0.34154 | 4.36464E–06 |
| *HSP90AA1* | 7981335 | chr14 | 101617138 | 101675776 | 0.795694 | 8.80286E–08 |
| *HSP90AB1* | 8119993 | chr6 | 44322826 | 44329592 | 1.32464 | 8.31882E–12 |
| *HSP90B1* | 7958130 | chr12 | 102848318 | 102865833 | 0.570376 | 3.44038E–06 |
| *HSPB1* | 8133721 | chr7 | 75769858 | 75771548 | 1.209542 | 3.15659E–11 |
| *HSPB2* | 7943787 | chr11 | 111288708 | 111290027 | –0.25678 | 4.39438E–05 |
| *HSPD1* | 8058052 | chr2 | 198059554 | 198072885 | 0.439166 | 2.52874E–05 |
| *IFI30* | 8026971 | chr19 | 18145578 | 18149927 | 0.71235 | 1.42987E–06 |
| *IGSF3* | 7918913 | chr1 | 116918553 | 117011837 | 0.70307 | 4.63142E–07 |
| *IL10RA* | 7944152 | chr11 | 117362318 | 117377404 | –0.71887 | 2.05094E–05 |
| *IL11* | 8039484 | chr19 | 60567568 | 60573626 | –0.29326 | 2.05077E–05 |
| *IL13RA2* | 8174598 | chrX | 114144795 | 114158463 | –0.82068 | 1.73109E–07 |
| *IL16* | 7985364 | chr15 | 79262254 | 79392156 | –0.3804 | 1.36538E–08 |
| *IL17D* | 7967969 | chr13 | 20175481 | 20195236 | –0.33104 | 4.96556E–06 |
| *IL18R1* | 8044035 | chr2 | 102345528 | 102381650 | –0.71507 | 3.18753E–08 |
| *IL18RAP* | 8044049 | chr2 | 102401685 | 102435457 | –0.62057 | 1.76407E–07 |
| *IL1B* | 8054722 | chr2 | 113303807 | 113310827 | –0.74679 | 5.88285E–09 |
| *IL1RL1* | 8044021 | chr2 | 102294393 | 102334929 | –0.80002 | 3.39305E–06 |
| *IL6* | 8138542 | chr7 | 22733344 | 22738141 | –0.261 | 5.37608E–06 |
| *ILF2* | 7920317 | chr1 | 151901137 | 151910103 | 1.4019 | 1.60593E–12 |
| *ILK* | 7938154 | chr11 | 6581782 | 6588673 | 0.352566 | 1.84227E–05 |
| *INPPL1* | 7942342 | chr11 | 71613472 | 71627796 | 0.391089 | 2.65932E–05 |
| *INS* | 7945688 | chr11 | 2137584 | 2139000 | –2.22155 | 2.18823E–13 |
| *IRF7* | 7945462 | chr11 | 602590 | 605921 | –0.28581 | 1.43358E–05 |
| *IRF8* | 7997712 | chr16 | 84490274 | 84513710 | –0.87501 | 1.87382E–07 |
| *ITGA2* | 8105267 | chr5 | 52321013 | 52423947 | 1.302186 | 2.74129E–05 |
| *ITGA5* | 7963786 | chr12 | 53075313 | 53099317 | 0.561493 | 2.40077E–05 |
| *ITGA6* | 8046380 | chr2 | 173000615 | 173079248 | 1.744526 | 1.08229E–09 |
| *ITGAD* | 7995161 | chr16 | 31312133 | 31345327 | –0.36128 | 5.9565E–08 |
| *KIR2DS4* | 8031358 | chr19 | 60035985 | 60051836 | –0.33292 | 3.01634E–06 |
| *KIR3DL3* | 8031260 | chr19 | 59927795 | 59939815 | –0.32776 | 2.37908E–06 |
| *KLRB1* | 7961059 | chr12 | 9639137 | 9651764 | –0.92873 | 2.66477E–09 |
| *KLRC1* | 7961187 | chr12 | 10489900 | 10497196 | –0.33829 | 1.49932E–05 |
| *KLRK1* | 7961151 | chr12 | 10416219 | 10451632 | –1.258 | 6.36059E–06 |
| *LAG3* | 7953418 | chr12 | 6751930 | 6757880 | –0.36339 | 3.61683E–05 |
| *LCP2* | 8115734 | chr5 | 169607666 | 169657400 | –0.73508 | 1.31829E–05 |
| *LECT2* | 8114263 | chr5 | 135310498 | 135318622 | –1.9022 | 3.83063E–06 |
| *LIFR* | 8111677 | chr5 | 38515661 | 38631253 | –2.60298 | 1.352E–12 |
| *LILRA1* | 8031213 | chr19 | 59796924 | 59804352 | –0.63621 | 6.51614E–08 |
| *LILRA5* | 8039236 | chr19 | 59510165 | 59516180 | –0.32526 | 3.66624E–06 |
| *LILRB1* | 8031223 | chr19 | 59820440 | 59840814 | –0.47349 | 5.40648E–06 |
| *LILRB2* | 8039212 | chr19 | 59470131 | 59476762 | –0.6168 | 1.07361E–07 |
| *LILRB5* | 8039180 | chr19 | 59446217 | 59452937 | –0.82856 | 7.82999E–12 |
| *LILRP2* | 8031253 | chr19 | 59911512 | 59916654 | –0.29397 | 2.68514E–05 |
| *LMNA* | 7906085 | chr1 | 154351121 | 154376495 | 0.48717 | 1.48856E–05 |
| *LTA4H* | 7965627 | chr12 | 94918741 | 94953496 | 0.738441 | 3.60696E–07 |
| *MAL* | 8043504 | chr2 | 95055205 | 95083462 | –0.30678 | 2.57677E–06 |
| *MAP2K3* | 8005707 | chr17 | 21128580 | 21158870 | –0.44694 | 1.65945E–05 |
| *MAP3K7* | 8128260 | chr6 | 91282073 | 91353628 | 0.542324 | 3.6236E–07 |
| *MAPK1* | 8074791 | chr22 | 20453331 | 20551970 | 0.704083 | 1.89215E–08 |
| *MAPK14* | 8119000 | chr6 | 36103550 | 36186513 | 0.516351 | 1.68781E–05 |
| *MAPK3* | 8000811 | chr16 | 30032927 | 30042039 | 0.409158 | 5.89631E–06 |
| *MAPK9* | 8116402 | chr5 | 179595389 | 179640216 | 0.82237 | 5.78801E–10 |
| *MAPKAPK5* | 7958800 | chr12 | 110764661 | 110815610 | 0.519019 | 1.08098E–07 |
| *MARCO* | 8044773 | chr2 | 119416214 | 119468707 | –2.09308 | 1.64588E–14 |
| *MASP1* | 8092661 | chr3 | 188434567 | 188492164 | –1.05427 | 8.93537E–07 |
| *MASP2* | 7912361 | chr1 | 11027442 | 11029872 | –1.47321 | 3.83481E–05 |
| *MAVS* | 8060705 | chr20 | 3775484 | 3795972 | 0.446409 | 1.30127E–05 |
| *MBL2* | 7933665 | chr10 | 54195146 | 54201466 | –1.77581 | 5.34271E–08 |
| *MED1* | 8014841 | chr17 | 34816379 | 34861030 | 0.86319 | 7.7767E–10 |
| *MEF2D* | 7921014 | chr1 | 154700142 | 154737153 | 0.487307 | 2.52079E–06 |
| *MEFV* | 7998940 | chr16 | 3232028 | 3246628 | –0.32914 | 9.28035E–06 |
| *MICB* | 8118116 | chr6 | 31573944 | 31586879 | 0.466434 | 1.35288E–05 |
| *NCOA2* | 8151254 | chr8 | 71186820 | 71478574 | 0.593684 | 1.22059E–05 |
| *NCR1* | 8031387 | chr19 | 60109319 | 60119197 | –0.38808 | 1.33969E–06 |
| *NFIL3* | 8162276 | chr9 | 93211149 | 93225965 | –0.74064 | 1.3039E–07 |
| *NFRKB* | 7952677 | chr11 | 129239574 | 129268114 | 0.466128 | 3.49745E–07 |
| *NLRC4* | 8051396 | chr2 | 32244877 | 32286158 | –0.38365 | 4.40207E–06 |
| *NLRP3* | 7911178 | chr1 | 245647974 | 245679027 | –0.40049 | 5.6043E–06 |
| *NMI* | 8055702 | chr2 | 151835230 | 151854620 | 0.497824 | 1.33462E–05 |
| *NR4A1* | 7955589 | chr12 | 50723762 | 50739552 | –0.76518 | 8.8936E–07 |
| *NRAS* | 7918813 | chr1 | 115051107 | 115061038 | 1.041889 | 2.49433E–11 |
| *PAK1* | 7950578 | chr11 | 76710708 | 76862581 | 0.614331 | 7.40274E–06 |
| *PARP1* | 7924733 | chr1 | 224615128 | 224662414 | 1.141952 | 4.8989E–10 |
| *PDE1A* | 8057486 | chr2 | 182740906 | 183095710 | –0.69376 | 2.92189E–08 |
| *PDE2A* | 7950162 | chr11 | 71964833 | 72063060 | –0.73977 | 2.34991E–08 |
| *PDGFRA* | 8095080 | chr4 | 54790203 | 54859168 | –1.6876 | 9.11621E–09 |
| *PDPK1* | 7998825 | chr16 | 2527970 | 2593189 | 0.630973 | 8.35986E–07 |
| *PGLYRP2* | 8035069 | chr19 | 15440462 | 15451312 | –1.80668 | 1.18491E–09 |
| *PIAS1* | 7984453 | chr15 | 66133625 | 66267456 | 0.551172 | 2.54169E–07 |
| *PIAS3* | 7904812 | chr1 | 144287388 | 144297903 | 0.833483 | 5.86158E–09 |
| *PIK3CA* | 8084016 | chr3 | 180349004 | 180435189 | 0.480379 | 1.27153E–05 |
| *PIK3CB* | 8091009 | chr3 | 139856920 | 139960875 | 0.875247 | 9.36864E–10 |
| *PLCB1* | 8060854 | chr20 | 8061295 | 8813547 | 1.355186 | 2.35944E–09 |
| *PLCB2* | 7987475 | chr15 | 38367401 | 38387330 | –0.71347 | 4.99375E–08 |
| *PPP1CA* | 7949765 | chr11 | 66922227 | 66925952 | 0.47833 | 1.02963E–06 |
| *PPP1CB* | 8041122 | chr2 | 28828129 | 28879309 | 0.561329 | 8.79973E–07 |
| *PPP1CC* | 7966368 | chr12 | 109642126 | 109665050 | 1.132466 | 5.27316E–13 |
| *PPP2CA* | 8114158 | chr5 | 133560046 | 133589849 | 0.565186 | 4.91021E–07 |
| *PPP2R1A* | 8030881 | chr19 | 57385045 | 57421482 | 0.628173 | 2.70975E–07 |
| *PRDX1* | 7915733 | chr1 | 45749294 | 45760196 | 0.650387 | 7.49743E–07 |
| *PRKAR2A* | 8087210 | chr3 | 48763096 | 48860274 | 0.652825 | 7.08268E–09 |
| *PRKAR2B* | 8135378 | chr7 | 106472413 | 106589491 | –0.95084 | 2.25932E–09 |
| *PRKCB* | 7994131 | chr16 | 23754822 | 24139063 | –0.76886 | 5.20237E–07 |
| *PSMA1* | 7946728 | chr11 | 14482998 | 14621739 | 0.644303 | 3.73559E–08 |
| *PSMB5* | 7977879 | chr14 | 22564899 | 22573949 | 0.573125 | 1.00877E–06 |
| *PTGDR* | 7974363 | chr14 | 51804180 | 51813191 | –0.58416 | 4.22525E–07 |
| *PTGES3* | 7964250 | chr12 | 55343648 | 55368156 | 0.944371 | 2.03572E–09 |
| *PTGIR* | 8037775 | chr19 | 51815565 | 51820194 | –0.38177 | 2.52029E–06 |
| *PTGIS* | 8066925 | chr20 | 47553819 | 47618114 | –0.91227 | 6.38956E–07 |
| *PTK2* | 8153223 | chr8 | 141737682 | 142080514 | 0.79025 | 1.48567E–07 |
| *PTPN11* | 7958846 | chr12 | 111340918 | 111432099 | 0.405446 | 5.70904E–06 |
| *PTPN12* | 8133788 | chr7 | 77198056 | 77300607 | 0.638902 | 2.56027E–08 |
| *PTPN13* | 8096176 | chr4 | 87734908 | 87955326 | –1.12886 | 1.59928E–14 |
| *RAF1* | 8085374 | chr3 | 12600107 | 12680678 | 0.607837 | 5.47809E–09 |
| *RASA1* | 8106784 | chr5 | 86600521 | 86723488 | 0.493553 | 2.09906E–06 |
| *RHEB* | 8143957 | chr7 | 150794728 | 150847942 | 1.008556 | 2.20562E–10 |
| *RHEB* | 7927285 | chr7 | 150794728 | 150847942 | 0.710082 | 1.16564E–09 |
| *RHOA* | 8087409 | chr3 | 49371584 | 49424530 | 0.710399 | 8.69847E–10 |
| *RIPK2* | 8147206 | chr8 | 90839172 | 90872432 | 0.677975 | 7.59082E–06 |
| *RIPK3* | 7978312 | chr14 | 23875071 | 23879013 | –0.32601 | 2.50793E–08 |
| *ROCK1* | 8022441 | chr18 | 16787532 | 16944869 | 0.490034 | 1.63335E–06 |
| *ROCK2* | 8050302 | chr2 | 11239228 | 11402162 | 0.664662 | 6.0707E–06 |
| *RPS6KB1* | 8008887 | chr17 | 55325224 | 55382566 | 0.447614 | 6.11154E–06 |
| *S100A12* | 7920238 | chr1 | 151612807 | 151614699 | –0.58723 | 2.63463E–05 |
| *S100A8* | 7920244 | chr1 | 151629132 | 151630173 | –1.05891 | 4.46576E–05 |
| *SCARF1* | 8011114 | chr17 | 1483902 | 1495791 | –0.32206 | 5.97413E–06 |
| *SDPR* | 8057797 | chr2 | 192407985 | 192420035 | –0.61535 | 1.85187E–06 |
| *SELP* | 7922200 | chr1 | 167824713 | 167866031 | –0.5746 | 5.58612E–07 |
| *SEMA4D* | 8162231 | chr9 | 91181971 | 91284431 | –0.36035 | 6.26573E–08 |
| *SERPING1* | 7940028 | chr11 | 57121733 | 57138895 | –0.77765 | 1.56692E–05 |
| *SH2D1B* | 7921900 | chr1 | 160631679 | 160648552 | –0.31342 | 8.14792E–08 |
| *SHC1* | 7920600 | chr1 | 153201398 | 153213464 | 0.553254 | 6.10935E–06 |
| *SIGLEC1* | 8064716 | chr20 | 3617206 | 3635775 | –0.61316 | 1.50884E–07 |
| *SIGLEC7* | 8030789 | chr19 | 56337385 | 56348595 | –0.46521 | 6.47019E–08 |
| *SOCS2* | 7957551 | chr12 | 92487728 | 92494109 | –1.256 | 1.17793E–08 |
| *SOCS3* | 8018864 | chr17 | 73864458 | 73867753 | –0.73909 | 1.63087E–05 |
| *SOCS4* | 7974447 | chr14 | 54563593 | 54585957 | 0.514559 | 4.44972E–07 |
| *SOCS5* | 8041820 | chr2 | 46779852 | 46843431 | 0.424168 | 1.16175E–06 |
| *SOS1* | 8051670 | chr2 | 39066468 | 39201067 | 0.549599 | 4.31355E–06 |
| *SPTAN1* | 8158317 | chr9 | 130354706 | 130435693 | 0.675932 | 1.90434E–08 |
| *STAT4* | 8057771 | chr2 | 191602550 | 191724170 | –0.80184 | 8.63998E–06 |
| *TBK1* | 7956795 | chr12 | 63132203 | 63182158 | 0.501684 | 1.57409E–06 |
| *TBX21* | 8008029 | chr17 | 43165608 | 43178484 | –0.42043 | 4.61806E–09 |
| *TBXA2R* | 8032623 | chr19 | 3546517 | 3557658 | –0.58887 | 1.78452E–09 |
| *TBXAS1* | 8136557 | chr7 | 139175420 | 139366471 | –0.59495 | 3.82336E–06 |
| *TDP2* | 8124262 | chr6 | 24758184 | 24775094 | 0.888612 | 1.17271E–09 |
| *TGFBR1* | 8156826 | chr9 | 100907232 | 100952128 | 0.56227 | 1.19143E–06 |
| *THEM4* | 7920114 | chr1 | 150113156 | 150148603 | 0.561657 | 2.21741E–08 |
| *THY1* | 7952268 | chr11 | 118794097 | 118799064 | 1.123458 | 2.14335E–10 |
| *TICAM2* | 8113623 | chr5 | 114942246 | 114989610 | 0.582742 | 6.02533E–08 |
| *TLR4* | 8157524 | chr9 | 119506404 | 119517874 | –0.86364 | 9.57509E–07 |
| *TMEM189* | 8066964 | chr20 | 48173681 | 48203742 | 0.419593 | 1.12252E–06 |
| *TNFRSF10C* | 8145244 | chr8 | 23016378 | 23030893 | –0.2951 | 2.20297E–06 |
| *TNFRSF10D* | 8149749 | chr8 | 23049050 | 23077485 | –0.67002 | 1.3773E–07 |
| *TNFRSF1A* | 7960518 | chr12 | 6308184 | 6321522 | –0.45787 | 1.85105E–06 |
| *TNFSF12* | 8004464 | chr17 | 7393098 | 7401930 | –0.32464 | 2.12932E–06 |
| *TNFSF8* | 8163629 | chr9 | 116704944 | 116732591 | –0.55551 | 1.4915E–05 |
| *TRAF2* | 8159476 | chr9 | 138900785 | 138940887 | 0.358853 | 3.04403E–05 |
| *TSC1* | 8164781 | chr9 | 134756556 | 134809841 | 0.501045 | 3.85438E–05 |
| *TXK* | 8100210 | chr4 | 47763517 | 47831030 | –0.59175 | 2.12166E–08 |
| *VTCN1* | 7918936 | chr1 | 117487732 | 117555072 | –0.60395 | 6.12512E–06 |
| *YWHAB* | 8062880 | chr20 | 42947757 | 42970574 | 0.603357 | 4.74609E–08 |
| *YWHAG* | 8140398 | chr7 | 75794052 | 75826252 | 0.488931 | 1.13296E–06 |
| *YWHAH* | 8072577 | chr22 | 30670478 | 30683589 | 0.738881 | 1.14407E–07 |
| *YWHAQ* | 8050215 | chr2 | 9641556 | 9688557 | 0.713685 | 1.00991E–08 |
| *YWHAZ* | 8152096 | chr8 | 102000089 | 102033447 | 0.81156 | 1.66128E–06 |
| *ZEB1* | 7926916 | chr10 | 31648147 | 31856740 | 0.374503 | 5.26484E–06 |

*Transcript ID in the Affymetrix Human Gene 1.0 ST Array.

† NCBI36/hg18.

‡ Paired Student's *t*-test.

Abbreviations: Chr., chromosome; FC, log_2_(Fold changes), HCCs vs. adjacent non-tumor tissues.
