# Supplementary material for: Relatively Small Contribution of Methylation and Genomic Copy Number Aberration to the Aberrant Expression of Inflammation-Related Genes in HBV-Related Hepatocellular Carcinoma
Source: PLoS One. 2015 May 12;10(5):e0126836. doi: 10.1371/journal.pone.0126836 (PMC4429029; doi:10.1371/journal.pone.0126836)
Supplement: S4 Table — (DOC) [file pone.0126836.s006.doc]

**S4 Table. Inflammation-related Genes with Aberrant Methylation in HCC**

| **Symbol** | **Probe ID*** | **Chr.** | **Start**† | **Stop**† | **FC** | **P Value**‡ |
| --- | --- | --- | --- | --- | --- | --- |
| *ADORA1* | CHR01FS201361942 | chr1 | 201361942 | 201361991 | –1.13017 | 2.44E–07 |
| *ADORA1* | CHR01FS201361118 | chr1 | 201361118 | 201361167 | –1.68554 | 3.55E–07 |
| *ADORA1* | CHR01FS201361634 | chr1 | 201361634 | 201361683 | –1.26435 | 8.39E–07 |
| *ADRA2A* | CHR10FS112825059 | chr10 | 112825059 | 112825121 | –0.66561 | 4.82E–07 |
| *BCL2* | CHR18FS059136853 | chr18 | 59136853 | 59136902 | 1.377769 | 7.99E–08 |
| *BMP4* | CHR14FS053492340 | chr14 | 53492340 | 53492389 | 2.700758 | 1.18E–08 |
| *BMP4* | CHR14FS053492656 | chr14 | 53492656 | 53492705 | 1.78738 | 1.68E–07 |
| *BMP4* | CHR14FS053492542 | chr14 | 53492542 | 53492591 | 1.028192 | 6.15E–07 |
| *BPI* | CHR20FS036364945 | chr20 | 36364945 | 36364994 | –1.04742 | 1.2E–08 |
| *BPI* | CHR20FS036365027 | chr20 | 36365027 | 36365088 | –1.18813 | 4.76E–08 |
| *BPI* | CHR20FS036365129 | chr20 | 36365129 | 36365184 | –0.74312 | 3.25E–07 |
| *BTK* | CHRXFS100490580 | chrX | 100490580 | 100490629 | –0.99431 | 4.58E–08 |
| *C1QA* | CHR01FS022833364 | chr1 | 22833364 | 22833413 | –0.67611 | 7E–07 |
| *C4BPB* | CHR01FS205328299 | chr1 | 205328299 | 205328355 | 0.745012 | 3.51E–08 |
| *CCL1* | CHR17FS029714758 | chr17 | 29714758 | 29714817 | –1.07603 | 2.8E–08 |
| *CCL1* | CHR17FS029716241 | chr17 | 29716241 | 29716290 | –0.58073 | 7.42E–07 |
| *CCL13* | CHR17FS029714456 | chr17 | 29714456 | 29714505 | –1.53251 | 1.93E–09 |
| *CCL13* | CHR17FS029714662 | chr17 | 29714662 | 29714711 | –1.40296 | 2.32E–09 |
| *CCL13* | CHR17FS029714370 | chr17 | 29714370 | 29714419 | –1.42347 | 2.8E–09 |
| *CCL13* | CHR17FS029714180 | chr17 | 29714180 | 29714229 | –1.45339 | 5.73E–09 |
| *CCL13* | CHR17FS029714566 | chr17 | 29714566 | 29714615 | –1.33705 | 9.59E–09 |
| *CCL13* | CHR17FS029714076 | chr17 | 29714076 | 29714125 | –1.10506 | 9.67E–08 |
| *CCL13* | CHR17FS029714262 | chr17 | 29714262 | 29714311 | –0.89678 | 3.44E–07 |
| *CCL13* | CHR17FS029713980 | chr17 | 29713980 | 29714029 | –0.91281 | 4.71E–07 |
| *CCL20* | CHR02FS228386768 | chr2 | 228386768 | 228386817 | –0.80547 | 6.32E–08 |
| *CCL20* | CHR02FS228387164 | chr2 | 228387164 | 228387213 | –0.7978 | 1.2E–07 |
| *CCL20* | CHR02FS228387064 | chr2 | 228387064 | 228387113 | –1.2774 | 1.23E–07 |
| *CCL20* | CHR02FS228386962 | chr2 | 228386962 | 228387019 | –1.26765 | 2.09E–07 |
| *CCL20* | CHR02FS228386864 | chr2 | 228386864 | 228386913 | –1.06092 | 3.45E–07 |
| *CCL7* | CHR17FS029619842 | chr17 | 29619842 | 29619897 | –1.43284 | 1.24E–08 |
| *CCL7* | CHR17FS029621254 | chr17 | 29621254 | 29621317 | –0.93566 | 4.46E–08 |
| *CCL7* | CHR17FS029619946 | chr17 | 29619946 | 29620002 | –1.36437 | 1.64E–07 |
| *CCL7* | CHR17FS029621154 | chr17 | 29621154 | 29621203 | –0.99814 | 7.58E–07 |
| *CCND1* | CHR11FS069160332 | chr11 | 69160332 | 69160381 | 1.313773 | 1.25E–06 |
| *CCR2* | CHR03FS046368510 | chr3 | 46368510 | 46368510 | –0.76639 | 2.41E–07 |
| *CCR6* | CHR06FS167456124 | chr6 | 167456124 | 167456173 | –0.67275 | 2.12E–07 |
| *CD1A* | CHR01FS156488650 | chr1 | 156488650 | 156488700 | –1.45135 | 3.65E–09 |
| *CD1A* | CHR01FS156488210 | chr1 | 156488210 | 156488259 | –1.14015 | 2.31E–08 |
| *CD1A* | CHR01FS156488740 | chr1 | 156488740 | 156488796 | –1.39012 | 3.16E–08 |
| *CD1A* | CHR01FS156488132 | chr1 | 156488132 | 156488182 | –1.51696 | 3.83E–08 |
| *CD1A* | CHR01FS156488556 | chr1 | 156488556 | 156488606 | –1.08588 | 4.39E–08 |
| *CD1A* | CHR01FS156488842 | chr1 | 156488842 | 156488892 | –1.08638 | 6.5E–07 |
| *CD207* | CHR02FS070918458 | chr2 | 70918458 | 70918507 | –1.08738 | 4.13E–08 |
| *CD207* | CHR02FS070916356 | chr2 | 70916356 | 70916405 | –1.49097 | 7.78E–08 |
| *CD207* | CHR02FS070916260 | chr2 | 70916260 | 70916309 | –1.42532 | 8.21E–08 |
| *CD207* | CHR02FS070915974 | chr2 | 70915974 | 70916023 | –1.27724 | 1.49E–07 |
| *CD207* | CHR02FS070915870 | chr2 | 70915870 | 70915926 | –1.45251 | 1.97E–07 |
| *CD207* | CHR02FS070918854 | chr2 | 70918854 | 70918903 | –1.4139 | 7.93E–07 |
| *CD207* | CHR02FS070917652 | chr2 | 70917652 | 70917706 | –0.61455 | 1.11E–06 |
| *CD247* | CHR01FS165755862 | chr1 | 165755862 | 165755921 | –1.17898 | 2.37E–07 |
| *CD247* | CHR01FS165755786 | chr1 | 165755786 | 165755835 | –0.9087 | 9.23E–07 |
| *CD27* | CHR12FS006432801 | chr12 | 6432801 | 6432801 | 1.145342 | 9E–07 |
| *CD27* | CHR12FS006432701 | chr12 | 6432701 | 6432701 | 1.586543 | 1.1E–06 |
| *CD33* | CHR19FS056420673 | chr19 | 56420673 | 56420722 | –2.61666 | 2.81E–12 |
| *CD33* | CHR19FS056419667 | chr19 | 56419667 | 56419717 | –1.3449 | 3.3E–12 |
| *CD33* | CHR19FS056420361 | chr19 | 56420361 | 56420410 | –1.8482 | 4.09E–12 |
| *CD33* | CHR19FS056420561 | chr19 | 56420561 | 56420610 | –1.91383 | 1.62E–11 |
| *CD33* | CHR19FS056420281 | chr19 | 56420281 | 56420330 | –1.5933 | 7.74E–11 |
| *CD33* | CHR19FS056419779 | chr19 | 56419779 | 56419828 | –1.45004 | 2.26E–10 |
| *CD33* | CHR19FS056420459 | chr19 | 56420459 | 56420508 | –1.93149 | 2.68E–10 |
| *CD33* | CHR19FS056420157 | chr19 | 56420157 | 56420206 | –1.43097 | 1.01E–09 |
| *CD33* | CHR19FS056419971 | chr19 | 56419971 | 56420020 | –1.62186 | 3.21E–09 |
| *CD33* | CHR19FS056420057 | chr19 | 56420057 | 56420106 | –1.66874 | 4.21E–09 |
| *CD33* | CHR19FS056419881 | chr19 | 56419881 | 56419930 | –1.59869 | 2.4E–08 |
| *CD40LG* | CHRXFS135556769 | chrX | 135556769 | 135556818 | –0.56363 | 9.01E–07 |
| *CD40LG* | CHRXFS135556567 | chrX | 135556567 | 135556616 | –0.68484 | 1.19E–06 |
| *CD46* | CHR01FS205991508 | chr1 | 205991508 | 205991559 | 1.087041 | 4.98E–07 |
| *CD55* | CHR01FS205561076 | chr1 | 205561076 | 205561125 | 1.443126 | 4.32E–07 |
| *CD55* | CHR01FS205561274 | chr1 | 205561274 | 205561323 | 1.803881 | 5.65E–07 |
| *CD8A* | CHR02FS086870000 | chr2 | 86870000 | 86870049 | 2.016771 | 2.17E–07 |
| *CD8A* | CHR02FS086870787 | chr2 | 86870787 | 86870836 | 1.193949 | 3.32E–07 |
| *CEACAM3* | CHR19FS046992947 | chr19 | 46992947 | 46993003 | –0.74009 | 1.12E–07 |
| *CEACAM8* | CHR19FS047790417 | chr19 | 47790417 | 47790466 | –0.6272 | 6.5E–09 |
| *CEACAM8* | CHR19FS047790337 | chr19 | 47790337 | 47790386 | –0.72305 | 1.34E–08 |
| *CR1* | CHR01FS205884797 | chr1 | 205884797 | 205884847 | 1.224578 | 9.46E–08 |
| *CR1* | CHR01FS205884715 | chr1 | 205884715 | 205884764 | 1.238795 | 2.8E–07 |
| *CXCL14* | CHR05FS134945153 | chr5 | 134945153 | 134945202 | –0.79149 | 6.14E–07 |
| *CXCR1* | CHR02FS218740210 | chr2 | 218740210 | 218740266 | –0.63012 | 7.18E–07 |
| *CXCR2* | CHR02FS218699350 | chr2 | 218699350 | 218699399 | –0.988 | 2.57E–07 |
| *CXCR2* | CHR02FS218699274 | chr2 | 218699274 | 218699323 | –1.00687 | 5.85E–07 |
| *CXCR2* | CHR02FS218699174 | chr2 | 218699174 | 218699223 | –0.83996 | 8.87E–07 |
| *CYSLTR2* | CHR13FS048179301 | chr13 | 48179301 | 48179358 | –1.1537 | 1.09E–06 |
| *DAP* | CHR05FS010816065 | chr5 | 10816065 | 10816114 | 1.006225 | 5.56E–07 |
| *DAP* | CHR05FS010815859 | chr5 | 10815859 | 10815908 | 1.001971 | 7.77E–07 |
| *DARC* | CHR01FS157438812 | chr1 | 157438812 | 157438812 | –0.91092 | 4.74E–08 |
| *DUSP4* | CHR08FS029262532 | chr8 | 29262532 | 29262581 | 1.254216 | 1.07E–06 |
| *ESR1* | CHR06FS152170090 | chr6 | 152170090 | 152170139 | 1.242305 | 3.54E–07 |
| *ESR1* | CHR06FS152169902 | chr6 | 152169902 | 152169951 | 2.183071 | 8.2E–07 |
| *FKBP4* | CHR12FS002773569 | chr12 | 2773569 | 2773620 | 1.503166 | 3.76E–08 |
| *FKBP4* | CHR12FS002773653 | chr12 | 2773653 | 2773702 | 1.169293 | 8.48E–08 |
| *FPR2* | CHR19FS056956074 | chr19 | 56956074 | 56956123 | –0.7955 | 9.78E–07 |
| *FYN* | CHR06FS112301735 | chr6 | 112301735 | 112301784 | 1.622644 | 6.66E–07 |
| *FYN* | CHR06FS112303553 | chr6 | 112303553 | 112303604 | –0.76535 | 8.62E–07 |
| *HLA-DMA* | CHR06FS033029296 | chr6 | 33029296 | 33029296 | 1.04158 | 2.81E–07 |
| *HLA-DQB2* | CHR06FS032841343 | chr6 | 32841343 | 32841343 | –0.74646 | 1.42E–07 |
| *HLA-DQB2* | CHR06FS032841733 | chr6 | 32841733 | 32841733 | –1.24176 | 3.19E–07 |
| *HLA-DQB2* | CHR06FS032840217 | chr6 | 32840217 | 32840217 | –0.88106 | 8.33E–07 |
| *HRH1* | CHR03FS011153489 | chr3 | 11153489 | 11153538 | –1.58764 | 6.13E–07 |
| *HRH2* | CHR05FS175040354 | chr5 | 175040354 | 175040403 | –1.74092 | 2.51E–09 |
| *HRH2* | CHR05FS175040232 | chr5 | 175040232 | 175040281 | –1.71488 | 3.65E–09 |
| *HRH2* | CHR05FS175040154 | chr5 | 175040154 | 175040211 | –1.94308 | 1.91E–08 |
| *HRH2* | CHR05FS175040430 | chr5 | 175040430 | 175040479 | –1.59648 | 2.13E–07 |
| *HRH2* | CHR05FS175039842 | chr5 | 175039842 | 175039891 | –1.47775 | 4.23E–07 |
| *HRH2* | CHR05FS175039752 | chr5 | 175039752 | 175039801 | –1.12299 | 4.42E–07 |
| *HRH2* | CHR05FS175041243 | chr5 | 175041243 | 175041299 | –0.78436 | 4.54E–07 |
| *HRH2* | CHR05FS175041654 | chr5 | 175041654 | 175041712 | –0.49246 | 7.95E–07 |
| *IFIH1* | CHR02FS162884682 | chr2 | 162884682 | 162884740 | 0.687038 | 8.43E–07 |
| *IFNA5* | CHR09FS021296094 | chr9 | 21296094 | 21296150 | –0.93551 | 7.32E–07 |
| *IFNG* | CHR12FS066839800 | chr12 | 66839800 | 66839857 | –1.13737 | 4.43E–09 |
| *IFNK* | CHR09FS027518692 | chr9 | 27518692 | 27518692 | 1.163301 | 7.18E–07 |
| *IFNK* | CHR09FS027518792 | chr9 | 27518792 | 27518792 | 1.262653 | 7.54E–07 |
| *IFNK* | CHR09FS027518312 | chr9 | 27518312 | 27518312 | 1.480219 | 1.02E–06 |
| *IL12RB2* | CHR01FS067545800 | chr1 | 67545800 | 67545850 | 2.666653 | 3.5E–08 |
| *IL12RB2* | CHR01FS067545704 | chr1 | 67545704 | 67545753 | 2.776246 | 2.56E–07 |
| *IL13RA2* | CHRXFS114158455 | chrX | 114158455 | 114158511 | –1.14726 | 3.58E–07 |
| *IL17A* | CHR06FS052158224 | chr6 | 52158224 | 52158273 | –1.24059 | 6.36E–07 |
| *IL18RAP* | CHR02FS102401495 | chr2 | 102401495 | 102401554 | –1.21469 | 1.23E–06 |
| *IL1RL1* | CHR02FS102292628 | chr2 | 102292628 | 102292692 | –1.10435 | 1.11E–07 |
| *IL1RL1* | CHR02FS102292816 | chr2 | 102292816 | 102292866 | –0.59367 | 1.22E–07 |
| *IL1RL1* | CHR02FS102293004 | chr2 | 102293004 | 102293062 | –0.71807 | 2.01E–07 |
| *IL1RL1* | CHR02FS102292728 | chr2 | 102292728 | 102292786 | –0.87609 | 3.36E–07 |
| *IL21R* | CHR16FS027345465 | chr16 | 27345465 | 27345514 | –1.17819 | 1E–06 |
| *IL22* | CHR12FS066933460 | chr12 | 66933460 | 66933509 | –1.24274 | 9.5E–07 |
| *IL25* | CHR14FS022909918 | chr14 | 22909918 | 22909967 | –1.05656 | 4.63E–08 |
| *IL25* | CHR14FS022910240 | chr14 | 22910240 | 22910292 | –1.10808 | 7.85E–08 |
| *IL25* | CHR14FS022909736 | chr14 | 22909736 | 22909785 | –1.17223 | 9.01E–08 |
| *IL25* | CHR14FS022909842 | chr14 | 22909842 | 22909891 | –1.13388 | 9.44E–08 |
| *IL25* | CHR14FS022910018 | chr14 | 22910018 | 22910067 | –1.34328 | 1.59E–07 |
| *IL25* | CHR14FS022910422 | chr14 | 22910422 | 22910471 | –1.20482 | 2.7E–07 |
| *IL25* | CHR14FS022910322 | chr14 | 22910322 | 22910372 | –0.98444 | 8.66E–07 |
| *IL6* | CHR07FS022732295 | chr7 | 22732295 | 22732351 | –1.04123 | 6.51E–07 |
| *INS* | CHR11FS002138620 | chr11 | 2138620 | 2138620 | –1.3895 | 3.85E–07 |
| *IRAK3* | CHR12FS064867829 | chr12 | 64867829 | 64867887 | –0.93528 | 4.53E–08 |
| *IRAK3* | CHR12FS064867943 | chr12 | 64867943 | 64867996 | –1.04603 | 1.15E–07 |
| *IRF2* | CHR04FS185634856 | chr4 | 185634856 | 185634905 | 1.06433 | 2.53E–08 |
| *IRF2* | CHR04FS185634758 | chr4 | 185634758 | 185634807 | 0.703933 | 5.49E–07 |
| *IRF4* | CHR06FS000336270 | chr6 | 336270 | 336319 | 2.303203 | 3.42E–08 |
| *IRF4* | CHR06FS000338766 | chr6 | 338766 | 338815 | 1.519009 | 3.7E–07 |
| *ITGA4* | CHR02FS182030530 | chr2 | 182030530 | 182030579 | 1.765921 | 6.29E–07 |
| *ITGA4* | CHR02FS182030646 | chr2 | 182030646 | 182030695 | 1.841813 | 7.42E–07 |
| *ITGA4* | CHR02FS182030726 | chr2 | 182030726 | 182030775 | 2.516052 | 1.11E–06 |
| *JUN* | CHR01FS059024699 | chr1 | 59024699 | 59024699 | 0.48997 | 3.91E–07 |
| *KCNH8* | CHR03FS019165126 | chr3 | 19165126 | 19165175 | 2.463461 | 9.48E–07 |
| *KIR2DS4* | CHR19FS060051902 | chr19 | 60051902 | 60051951 | –1.47015 | 1.12E–07 |
| *KIR2DS4* | CHR19FS060051278 | chr19 | 60051278 | 60051327 | –1.71622 | 1.34E–07 |
| *KIR2DS4* | CHR19FS060051796 | chr19 | 60051796 | 60051845 | –1.42118 | 2.31E–07 |
| *KIR2DS4* | CHR19FS060051668 | chr19 | 60051668 | 60051717 | –1.3444 | 4.7E–07 |
| *KIR2DS4* | CHR19FS060052350 | chr19 | 60052350 | 60052413 | –1.38907 | 9.68E–07 |
| *KIR2DS4* | CHR19FS060052268 | chr19 | 60052268 | 60052325 | –1.48865 | 1.03E–06 |
| *KIR2DS4* | CHR19FS060052148 | chr19 | 60052148 | 60052209 | –1.44185 | 1.26E–06 |
| *KIR3DL2* | CHR19FS060017369 | chr19 | 60017369 | 60017369 | –1.71592 | 7.22E–09 |
| *KIR3DL2* | CHR19FS060033867 | chr19 | 60033867 | 60033867 | –1.66315 | 1.74E–08 |
| *KIR3DL2* | CHR19FS060033651 | chr19 | 60033651 | 60033651 | –1.65337 | 1.94E–08 |
| *KIR3DL2* | CHR19FS060006758 | chr19 | 60006758 | 60006758 | –1.5212 | 2.2E–08 |
| *KIR3DL2* | CHR19FS060017877 | chr19 | 60017877 | 60017877 | –1.27136 | 3.48E–08 |
| *KIR3DL2* | CHR19FS060017265 | chr19 | 60017265 | 60017265 | –1.66738 | 3.64E–08 |
| *KIR3DL2* | CHR19FS060033559 | chr19 | 60033559 | 60033559 | –1.71466 | 4.11E–08 |
| *KIR3DL2* | CHR19FS060017471 | chr19 | 60017471 | 60017471 | –1.83468 | 4.15E–08 |
| *KIR3DL2* | CHR19FS060017589 | chr19 | 60017589 | 60017589 | –1.7404 | 5.84E–08 |
| *KIR3DL2* | CHR19FS060033959 | chr19 | 60033959 | 60033959 | –1.49364 | 6.13E–08 |
| *KIR3DL2* | CHR19FS060006660 | chr19 | 60006660 | 60006660 | –1.52547 | 9.35E–08 |
| *KIR3DL2* | CHR19FS060017673 | chr19 | 60017673 | 60017673 | –1.21423 | 1.98E–07 |
| *KIR3DL2* | CHR19FS060017773 | chr19 | 60017773 | 60017773 | –1.06344 | 3.94E–07 |
| *KIR3DL2* | CHR19FS060034295 | chr19 | 60034295 | 60034295 | –1.70771 | 4.12E–07 |
| *KIR3DL2* | CHR19FS060034047 | chr19 | 60034047 | 60034047 | –1.47701 | 5.17E–07 |
| *KIR3DL2* | CHR19FS060034435 | chr19 | 60034435 | 60034435 | –1.04509 | 1.2E–06 |
| *LAG3* | CHR12FS006750613 | chr12 | 6750613 | 6750662 | 0.955802 | 1.84E–07 |
| *LAG3* | CHR12FS006751201 | chr12 | 6751201 | 6751250 | 1.02188 | 6.07E–07 |
| *LAG3* | CHR12FS006750407 | chr12 | 6750407 | 6750456 | 0.688983 | 1.24E–06 |
| *LAG3* | CHR12FS006751005 | chr12 | 6751005 | 6751061 | 0.956234 | 1.26E–06 |
| *LAIR1* | CHR19FS059568433 | chr19 | 59568433 | 59568484 | –1.73568 | 1.64E–11 |
| *LAIR1* | CHR19FS059568247 | chr19 | 59568247 | 59568296 | –0.68124 | 1.81E–07 |
| *LCP2* | CHR05FS169657311 | chr5 | 169657311 | 169657360 | –1.46804 | 2.1E–09 |
| *LCP2* | CHR05FS169656995 | chr5 | 169656995 | 169657044 | –2.66885 | 4.11E–09 |
| *LCP2* | CHR05FS169657391 | chr5 | 169657391 | 169657440 | –1.5719 | 1.09E–08 |
| *LCP2* | CHR05FS169656803 | chr5 | 169656803 | 169656852 | –2.14276 | 2.01E–08 |
| *LCP2* | CHR05FS169656915 | chr5 | 169656915 | 169656964 | –1.99211 | 5.95E–08 |
| *LCP2* | CHR05FS169657093 | chr5 | 169657093 | 169657142 | –1.7355 | 1.42E–07 |
| *LILRA1* | CHR19FS059797315 | chr19 | 59797315 | 59797364 | –1.75043 | 8.06E–13 |
| *LILRA1* | CHR19FS059797515 | chr19 | 59797515 | 59797564 | –2.05441 | 9.06E–13 |
| *LILRA1* | CHR19FS059796302 | chr19 | 59796302 | 59796358 | –1.42893 | 2.1E–11 |
| *LILRA1* | CHR19FS059796506 | chr19 | 59796506 | 59796555 | –1.58204 | 5.59E–11 |
| *LILRA1* | CHR19FS059797133 | chr19 | 59797133 | 59797182 | –1.60205 | 6.13E–11 |
| *LILRA1* | CHR19FS059797209 | chr19 | 59797209 | 59797258 | –1.21055 | 5.92E–10 |
| *LILRA1* | CHR19FS059796184 | chr19 | 59796184 | 59796233 | –1.12678 | 6.35E–10 |
| *LILRA1* | CHR19FS059796588 | chr19 | 59796588 | 59796644 | –1.86448 | 7.52E–10 |
| *LILRA1* | CHR19FS059797033 | chr19 | 59797033 | 59797082 | –1.42017 | 1.3E–09 |
| *LILRA1* | CHR19FS059797409 | chr19 | 59797409 | 59797458 | –1.01239 | 1.02E–08 |
| *LILRA1* | CHR19FS059796684 | chr19 | 59796684 | 59796733 | –1.25782 | 1.31E–08 |
| *LILRA1* | CHR19FS059796406 | chr19 | 59796406 | 59796455 | –1.18482 | 3.06E–08 |
| *LILRA1* | CHR19FS059796823 | chr19 | 59796823 | 59796872 | –1.47438 | 1.46E–07 |
| *LILRA2* | CHR19FS059777053 | chr19 | 59777053 | 59777102 | –1.54493 | 2.18E–11 |
| *LILRA2* | CHR19FS059776841 | chr19 | 59776841 | 59776890 | –1.81921 | 2.63E–11 |
| *LILRA2* | CHR19FS059777243 | chr19 | 59777243 | 59777292 | –1.73728 | 2.82E–11 |
| *LILRA2* | CHR19FS059777677 | chr19 | 59777677 | 59777728 | –1.69995 | 1.38E–10 |
| *LILRA2* | CHR19FS059775553 | chr19 | 59775553 | 59775602 | –1.1572 | 1.84E–10 |
| *LILRA2* | CHR19FS059775353 | chr19 | 59775353 | 59775402 | –1.69417 | 3.42E–10 |
| *LILRA2* | CHR19FS059777553 | chr19 | 59777553 | 59777602 | –1.52713 | 4.01E–10 |
| *LILRA2* | CHR19FS059776635 | chr19 | 59776635 | 59776684 | –1.26312 | 9.18E–10 |
| *LILRA2* | CHR19FS059777473 | chr19 | 59777473 | 59777522 | –1.39685 | 2.35E–09 |
| *LILRA2* | CHR19FS059776543 | chr19 | 59776543 | 59776592 | –0.87042 | 2.75E–09 |
| *LILRA2* | CHR19FS059776943 | chr19 | 59776943 | 59776992 | –1.10072 | 3.04E–09 |
| *LILRA2* | CHR19FS059775675 | chr19 | 59775675 | 59775724 | –1.38852 | 3.84E–09 |
| *LILRA2* | CHR19FS059775273 | chr19 | 59775273 | 59775322 | –1.57154 | 1.1E–08 |
| *LILRA2* | CHR19FS059777363 | chr19 | 59777363 | 59777412 | –1.10581 | 1.99E–08 |
| *LILRA2* | CHR19FS059775175 | chr19 | 59775175 | 59775224 | –1.33164 | 2.1E–08 |
| *LILRA2* | CHR19FS059795608 | chr19 | 59795608 | 59795657 | –1.34368 | 2.33E–08 |
| *LILRA2* | CHR19FS059777151 | chr19 | 59777151 | 59777200 | –1.35437 | 8.28E–08 |
| *LILRA2* | CHR19FS059795306 | chr19 | 59795306 | 59795355 | –0.73688 | 1.66E–07 |
| *LILRA2* | CHR19FS059775963 | chr19 | 59775963 | 59776020 | –0.82873 | 1.95E–07 |
| *LILRA2* | CHR19FS059775775 | chr19 | 59775775 | 59775824 | –1.57958 | 4.27E–07 |
| *LILRA2* | CHR19FS059795486 | chr19 | 59795486 | 59795535 | –1.11783 | 1.24E–06 |
| *LILRA3* | CHR19FS059495940 | chr19 | 59495940 | 59495989 | –1.76461 | 1.17E–11 |
| *LILRA3* | CHR19FS059495564 | chr19 | 59495564 | 59495613 | –1.79431 | 5.98E–11 |
| *LILRA3* | CHR19FS059495852 | chr19 | 59495852 | 59495901 | –1.49234 | 3.4E–10 |
| *LILRA3* | CHR19FS059495652 | chr19 | 59495652 | 59495701 | –1.47462 | 2.25E–09 |
| *LILRA3* | CHR19FS059496374 | chr19 | 59496374 | 59496423 | –1.44784 | 3.08E–09 |
| *LILRA3* | CHR19FS059496765 | chr19 | 59496765 | 59496820 | –1.47272 | 1.09E–08 |
| *LILRA3* | CHR19FS059496867 | chr19 | 59496867 | 59496916 | –1.11263 | 3.19E–08 |
| *LILRA3* | CHR19FS059495760 | chr19 | 59495760 | 59495809 | –0.80509 | 3.62E–08 |
| *LILRA3* | CHR19FS059496468 | chr19 | 59496468 | 59496517 | –0.88654 | 9.07E–08 |
| *LILRA4* | CHR19FS059542948 | chr19 | 59542948 | 59542997 | –1.41894 | 8.5E–10 |
| *LILRA4* | CHR19FS059542824 | chr19 | 59542824 | 59542873 | –1.26793 | 2.95E–09 |
| *LILRA4* | CHR19FS059543428 | chr19 | 59543428 | 59543490 | –1.3252 | 3.33E–08 |
| *LILRA4* | CHR19FS059541624 | chr19 | 59541624 | 59541673 | –1.20265 | 4.09E–08 |
| *LILRA4* | CHR19FS059542746 | chr19 | 59542746 | 59542795 | –0.71774 | 7.04E–08 |
| *LILRA4* | CHR19FS059543348 | chr19 | 59543348 | 59543397 | –1.04859 | 7.5E–08 |
| *LILRA4* | CHR19FS059541836 | chr19 | 59541836 | 59541885 | –1.185 | 7.75E–08 |
| *LILRA4* | CHR19FS059541748 | chr19 | 59541748 | 59541797 | –1.04351 | 1.19E–07 |
| *LILRA4* | CHR19FS059543044 | chr19 | 59543044 | 59543093 | –1.28706 | 3.77E–07 |
| *LILRA5* | CHR19FS059515672 | chr19 | 59515672 | 59515721 | –0.82169 | 1.15E–06 |
| *LILRA6* | CHR19FS059439808 | chr19 | 59439808 | 59439857 | –1.33102 | 5.11E–09 |
| *LILRA6* | CHR19FS059440010 | chr19 | 59440010 | 59440059 | –1.4411 | 1.21E–08 |
| *LILRA6* | CHR19FS059440126 | chr19 | 59440126 | 59440175 | –1.71545 | 2.87E–08 |
| *LILRA6* | CHR19FS059439924 | chr19 | 59439924 | 59439973 | –1.38909 | 4.47E–08 |
| *LILRA6* | CHR19FS059438832 | chr19 | 59438832 | 59438881 | –1.17798 | 4.69E–08 |
| *LILRA6* | CHR19FS059438950 | chr19 | 59438950 | 59438999 | –0.83946 | 4.97E–08 |
| *LILRA6* | CHR19FS059439618 | chr19 | 59439618 | 59439670 | –1.5216 | 6.59E–08 |
| *LILRA6* | CHR19FS059439704 | chr19 | 59439704 | 59439753 | –1.69006 | 9E–08 |
| *LILRA6* | CHR19FS059439404 | chr19 | 59439404 | 59439453 | –0.87555 | 1.06E–07 |
| *LILRA6* | CHR19FS059437948 | chr19 | 59437948 | 59437997 | –1.14381 | 2.28E–07 |
| *LILRA6* | CHR19FS059438244 | chr19 | 59438244 | 59438293 | –1.10363 | 3.09E–07 |
| *LILRA6* | CHR19FS059438332 | chr19 | 59438332 | 59438381 | –1.1673 | 4.37E–07 |
| *LILRA6* | CHR19FS059439502 | chr19 | 59439502 | 59439551 | –1.40519 | 5.9E–07 |
| *LILRA6* | CHR19FS059438538 | chr19 | 59438538 | 59438587 | –0.945 | 6.66E–07 |
| *LILRA6* | CHR19FS059440208 | chr19 | 59440208 | 59440257 | –1.53594 | 7.33E–07 |
| *LILRA6* | CHR19FS059438436 | chr19 | 59438436 | 59438485 | –1.08406 | 8.88E–07 |
| *LILRA6* | CHR19FS059438136 | chr19 | 59438136 | 59438185 | –0.99304 | 9.13E–07 |
| *LILRB1* | CHR19FS059833071 | chr19 | 59833071 | 59833120 | –1.85912 | 6.7E–11 |
| *LILRB1* | CHR19FS059834368 | chr19 | 59834368 | 59834417 | –1.64689 | 7.96E–11 |
| *LILRB1* | CHR19FS059833355 | chr19 | 59833355 | 59833404 | –1.54258 | 1.71E–10 |
| *LILRB1* | CHR19FS059833259 | chr19 | 59833259 | 59833308 | –1.97168 | 3.34E–10 |
| *LILRB1* | CHR19FS059832957 | chr19 | 59832957 | 59833006 | –1.47094 | 3.94E–10 |
| *LILRB1* | CHR19FS059833968 | chr19 | 59833968 | 59834017 | –1.97408 | 3.97E–10 |
| *LILRB1* | CHR19FS059833455 | chr19 | 59833455 | 59833508 | –1.92403 | 8.77E–10 |
| *LILRB1* | CHR19FS059832755 | chr19 | 59832755 | 59832812 | –1.64871 | 1.12E–09 |
| *LILRB1* | CHR19FS059834158 | chr19 | 59834158 | 59834207 | –1.61187 | 2.55E–09 |
| *LILRB1* | CHR19FS059833870 | chr19 | 59833870 | 59833919 | –1.362 | 8.98E–09 |
| *LILRB1* | CHR19FS059833674 | chr19 | 59833674 | 59833727 | –1.81641 | 1.21E–08 |
| *LILRB1* | CHR19FS059833569 | chr19 | 59833569 | 59833618 | –1.7309 | 1.31E–08 |
| *LILRB1* | CHR19FS059833778 | chr19 | 59833778 | 59833827 | –0.76514 | 4.79E–08 |
| *LILRB1* | CHR19FS059833163 | chr19 | 59833163 | 59833213 | –1.01679 | 5.9E–08 |
| *LILRB1* | CHR19FS059834074 | chr19 | 59834074 | 59834123 | –1.23569 | 1.52E–07 |
| *LILRB1* | CHR19FS059832255 | chr19 | 59832255 | 59832304 | –1.36999 | 2.07E–07 |
| *LILRB1* | CHR19FS059834266 | chr19 | 59834266 | 59834315 | –1.16111 | 5.77E–07 |
| *LILRB1* | CHR19FS059832461 | chr19 | 59832461 | 59832510 | –1.431 | 6.17E–07 |
| *LILRB1* | CHR19FS059832555 | chr19 | 59832555 | 59832612 | –1.25851 | 7.01E–07 |
| *LILRB2* | CHR19FS059477384 | chr19 | 59477384 | 59477436 | –2.06027 | 9.51E–13 |
| *LILRB2* | CHR19FS059476449 | chr19 | 59476449 | 59476498 | –1.80899 | 2.28E–12 |
| *LILRB2* | CHR19FS059477272 | chr19 | 59477272 | 59477321 | –1.764 | 4.56E–12 |
| *LILRB2* | CHR19FS059476249 | chr19 | 59476249 | 59476298 | –1.34007 | 5.3E–11 |
| *LILRB2* | CHR19FS059476351 | chr19 | 59476351 | 59476400 | –1.09868 | 5.96E–11 |
| *LILRB2* | CHR19FS059477180 | chr19 | 59477180 | 59477229 | –1.60843 | 8.88E–11 |
| *LILRB2* | CHR19FS059477060 | chr19 | 59477060 | 59477109 | –1.89007 | 2.19E–10 |
| *LILRB2* | CHR19FS059477478 | chr19 | 59477478 | 59477527 | –1.45527 | 2.22E–10 |
| *LILRB2* | CHR19FS059476966 | chr19 | 59476966 | 59477022 | –1.82998 | 2.91E–10 |
| *LILRB2* | CHR19FS059476555 | chr19 | 59476555 | 59476604 | –1.6169 | 4.15E–10 |
| *LILRB2* | CHR19FS059476781 | chr19 | 59476781 | 59476833 | –1.68171 | 6.16E–09 |
| *LILRB2* | CHR19FS059478074 | chr19 | 59478074 | 59478128 | –1.41781 | 1.36E–08 |
| *LILRB2* | CHR19FS059476685 | chr19 | 59476685 | 59476734 | –1.16044 | 1.48E–08 |
| *LILRB2* | CHR19FS059477660 | chr19 | 59477660 | 59477709 | –1.04336 | 2.03E–08 |
| *LILRB2* | CHR19FS059477778 | chr19 | 59477778 | 59477827 | –0.71457 | 2.44E–08 |
| *LILRB2* | CHR19FS059478384 | chr19 | 59478384 | 59478434 | –0.85725 | 6.27E–08 |
| *LILRB2* | CHR19FS059478260 | chr19 | 59478260 | 59478309 | –1.18131 | 8.97E–08 |
| *LILRB2* | CHR19FS059478176 | chr19 | 59478176 | 59478226 | –1.42857 | 5.03E–07 |
| *LILRB2* | CHR19FS059477970 | chr19 | 59477970 | 59478019 | –1.20975 | 9.7E–07 |
| *LILRB3* | CHR19FS059418761 | chr19 | 59418761 | 59418810 | –1.14014 | 1.06E–09 |
| *LILRB3* | CHR19FS059420385 | chr19 | 59420385 | 59420434 | –1.48304 | 6.04E–09 |
| *LILRB3* | CHR19FS059419983 | chr19 | 59419983 | 59420032 | –1.70933 | 9E–09 |
| *LILRB3* | CHR19FS059418467 | chr19 | 59418467 | 59418516 | –1.44395 | 1.73E–08 |
| *LILRB3* | CHR19FS059420169 | chr19 | 59420169 | 59420218 | –1.34372 | 2.32E–08 |
| *LILRB3* | CHR19FS059418581 | chr19 | 59418581 | 59418630 | –1.33588 | 3.04E–08 |
| *LILRB3* | CHR19FS059419881 | chr19 | 59419881 | 59419937 | –1.66485 | 4.4E–08 |
| *LILRB3* | CHR19FS059418873 | chr19 | 59418873 | 59418922 | –1.10853 | 4.62E–08 |
| *LILRB3* | CHR19FS059418683 | chr19 | 59418683 | 59418732 | –1.04266 | 7.11E–08 |
| *LILRB3* | CHR19FS059418175 | chr19 | 59418175 | 59418224 | –1.07967 | 7.99E–08 |
| *LILRB3* | CHR19FS059420061 | chr19 | 59420061 | 59420110 | –1.3896 | 9.03E–08 |
| *LILRB3* | CHR19FS059419079 | chr19 | 59419079 | 59419128 | –1.28597 | 2.27E–07 |
| *LILRB3* | CHR19FS059419765 | chr19 | 59419765 | 59419814 | –1.34972 | 7.01E–07 |
| *LILRB3* | CHR19FS059418269 | chr19 | 59418269 | 59418318 | –1.17179 | 9.68E–07 |
| *LILRB3* | CHR19FS059419663 | chr19 | 59419663 | 59419712 | –0.95181 | 1.15E–06 |
| *LILRB4* | CHR19FS059864136 | chr19 | 59864136 | 59864185 | –1.64928 | 9.9E–11 |
| *LILRB4* | CHR19FS059864220 | chr19 | 59864220 | 59864270 | –1.83037 | 6.15E–10 |
| *LILRB4* | CHR19FS059864042 | chr19 | 59864042 | 59864091 | –1.38777 | 1.71E–08 |
| *LILRB4* | CHR19FS059864320 | chr19 | 59864320 | 59864369 | –1.20345 | 2.95E–08 |
| *LILRB4* | CHR19FS059864428 | chr19 | 59864428 | 59864480 | –1.01991 | 3.24E–08 |
| *LILRB4* | CHR19FS059863942 | chr19 | 59863942 | 59863991 | –1.22416 | 4.49E–08 |
| *LILRB4* | CHR19FS059863836 | chr19 | 59863836 | 59863885 | –0.94962 | 5.24E–08 |
| *LILRB4* | CHR19FS059866045 | chr19 | 59866045 | 59866094 | –0.87411 | 7.89E–08 |
| *LILRB4* | CHR19FS059863744 | chr19 | 59863744 | 59863793 | –1.01443 | 1.26E–06 |
| *LILRB5* | CHR19FS059452676 | chr19 | 59452676 | 59452725 | –1.57436 | 2.15E–08 |
| *LILRB5* | CHR19FS059453596 | chr19 | 59453596 | 59453645 | –0.9192 | 3.85E–08 |
| *LILRB5* | CHR19FS059452886 | chr19 | 59452886 | 59452935 | –1.45881 | 4.22E–08 |
| *LILRB5* | CHR19FS059452770 | chr19 | 59452770 | 59452819 | –1.25356 | 9.13E–08 |
| *LILRB5* | CHR19FS059452470 | chr19 | 59452470 | 59452519 | –1.31245 | 1.96E–07 |
| *LILRB5* | CHR19FS059453394 | chr19 | 59453394 | 59453443 | –1.09365 | 2.49E–07 |
| *LILRB5* | CHR19FS059452592 | chr19 | 59452592 | 59452641 | –1.0737 | 3.7E–07 |
| *LILRB5* | CHR19FS059453218 | chr19 | 59453218 | 59453267 | –1.41729 | 5.02E–07 |
| *LILRB5* | CHR19FS059453002 | chr19 | 59453002 | 59453051 | –0.7685 | 7.55E–07 |
| *LILRP2* | CHR19FS059909194 | chr19 | 59909194 | 59909243 | –1.30976 | 1.19E–07 |
| *LILRP2* | CHR19FS059909386 | chr19 | 59909386 | 59909435 | –1.4248 | 3.07E–07 |
| *MADD* | CHR11FS047247437 | chr11 | 47247437 | 47247486 | 1.958156 | 4.23E–08 |
| *MADD* | CHR11FS047247313 | chr11 | 47247313 | 47247362 | 1.322657 | 1.06E–07 |
| *MADD* | CHR11FS047247215 | chr11 | 47247215 | 47247272 | 1.824406 | 5.43E–07 |
| *MADD* | CHR11FS047247137 | chr11 | 47247137 | 47247186 | 1.768924 | 9.66E–07 |
| *MAP3K14* | CHR17FS040751115 | chr17 | 40751115 | 40751171 | 0.695659 | 6.51E–07 |
| *MARCO* | CHR02FS119416398 | chr2 | 119416398 | 119416455 | –1.5438 | 2.22E–08 |
| *MARCO* | CHR02FS119416486 | chr2 | 119416486 | 119416543 | –1.28424 | 2.46E–07 |
| *MARCO* | CHR02FS119414792 | chr2 | 119414792 | 119414841 | –1.4402 | 3.13E–07 |
| *MARCO* | CHR02FS119416298 | chr2 | 119416298 | 119416347 | –1.02548 | 7.14E–07 |
| *MEF2C* | CHR05FS088215998 | chr5 | 88215998 | 88216052 | 1.708617 | 1.08E–06 |
| *MMP9* | CHR20FS044069834 | chr20 | 44069834 | 44069884 | –1.07634 | 2.34E–07 |
| *MSR1* | CHR08FS016094494 | chr8 | 16094494 | 16094553 | –0.73182 | 1.45E–07 |
| *MUC1* | CHR01FS153430833 | chr1 | 153430833 | 153430882 | 1.661523 | 9.08E–07 |
| *NCR1* | CHR19FS060109473 | chr19 | 60109473 | 60109522 | –0.8051 | 1.71E–07 |
| *NCR1* | CHR19FS060109057 | chr19 | 60109057 | 60109106 | –0.92689 | 3.58E–07 |
| *NCR1* | CHR19FS060109157 | chr19 | 60109157 | 60109206 | –1.11721 | 6.96E–07 |
| *NFATC1* | CHR18FS075320640 | chr18 | 75320640 | 75320689 | –0.4299 | 6.33E–07 |
| *NLRP3* | CHR01FS245681393 | chr1 | 245681393 | 245681442 | –1.81313 | 1.08E–08 |
| *NLRP3* | CHR01FS245681207 | chr1 | 245681207 | 245681256 | –1.93368 | 2.43E–08 |
| *NLRP3* | CHR01FS245681299 | chr1 | 245681299 | 245681348 | –1.96462 | 5.45E–08 |
| *NLRP3* | CHR01FS245648065 | chr1 | 245648065 | 245648115 | –1.1745 | 1.42E–07 |
| *NLRP3* | CHR01FS245682613 | chr1 | 245682613 | 245682663 | –1.31899 | 2.3E–07 |
| *NLRP3* | CHR01FS245681117 | chr1 | 245681117 | 245681166 | –1.75703 | 2.66E–07 |
| *NLRP3* | CHR01FS245648181 | chr1 | 245648181 | 245648230 | –1.01237 | 3.02E–07 |
| *NLRP3* | CHR01FS245645540 | chr1 | 245645540 | 245645589 | –0.61888 | 3.6E–07 |
| *NLRP3* | CHR01FS245647979 | chr1 | 245647979 | 245648030 | –1.00337 | 4.66E–07 |
| *NLRP3* | CHR01FS245681711 | chr1 | 245681711 | 245681760 | –1.51807 | 6.06E–07 |
| *NLRP3* | CHR01FS245681611 | chr1 | 245681611 | 245681660 | –1.44731 | 6.77E–07 |
| *NLRP3* | CHR01FS245683917 | chr1 | 245683917 | 245683966 | –1.47271 | 1.1E–06 |
| *NLRP3* | CHR01FS245647789 | chr1 | 245647789 | 245647838 | –1.14503 | 1.19E–06 |
| *NR2F1* | CHR05FS092949667 | chr5 | 92949667 | 92949716 | 0.595724 | 9.56E–07 |
| *NR4A2* | CHR02FS156885421 | chr2 | 156885421 | 156885470 | 1.130123 | 1.65E–08 |
| *NRAS* | CHR01FS115062862 | chr1 | 115062862 | 115062862 | –1.22279 | 3.76E–08 |
| *PAG1* | CHR08FS082189148 | chr8 | 82189148 | 82189202 | –0.81561 | 2.54E–07 |
| *PAK1* | CHR11FS076800830 | chr11 | 76800830 | 76800879 | 1.541341 | 6.81E–07 |
| *PAX5* | CHR09FS037028168 | chr9 | 37028168 | 37028217 | 1.534409 | 3.22E–09 |
| *PAX5* | CHR09FS037027870 | chr9 | 37027870 | 37027919 | 1.763854 | 3.67E–09 |
| *PAX5* | CHR09FS037028286 | chr9 | 37028286 | 37028335 | 2.111363 | 1.31E–08 |
| *PAX5* | CHR09FS037027984 | chr9 | 37027984 | 37028033 | 1.800044 | 2.38E–08 |
| *PAX5* | CHR09FS037028088 | chr9 | 37028088 | 37028137 | 2.233444 | 2.86E–08 |
| *PAX5* | CHR09FS037027766 | chr9 | 37027766 | 37027815 | 0.588759 | 1.11E–06 |
| *PDE2A* | CHR11FS072058648 | chr11 | 72058648 | 72058697 | –1.2049 | 1.24E–06 |
| *PDE2A* | CHR11FS072058748 | chr11 | 72058748 | 72058798 | –1.11141 | 1.26E–06 |
| *PDE4C* | CHR19FS018197218 | chr19 | 18197218 | 18197267 | 1.437373 | 9.37E–07 |
| *PDPK1* | CHR16FS002555247 | chr16 | 2555247 | 2555304 | –0.95576 | 7.08E–10 |
| *PGLYRP3* | CHR01FS151552214 | chr1 | 151552214 | 151552263 | –1.05113 | 2.7E–07 |
| *PGLYRP4* | CHR01FS151588454 | chr1 | 151588454 | 151588503 | –2.26948 | 3.63E–11 |
| *PGLYRP4* | CHR01FS151588360 | chr1 | 151588360 | 151588409 | –2.38496 | 6.21E–10 |
| *PGLYRP4* | CHR01FS151588148 | chr1 | 151588148 | 151588197 | –2.38631 | 8.25E–10 |
| *PGLYRP4* | CHR01FS151588552 | chr1 | 151588552 | 151588601 | –1.55524 | 8.89E–10 |
| *PGLYRP4* | CHR01FS151588236 | chr1 | 151588236 | 151588285 | –2.46232 | 1.45E–09 |
| *PGLYRP4* | CHR01FS151588058 | chr1 | 151588058 | 151588107 | –1.90076 | 1.73E–09 |
| *PGLYRP4* | CHR01FS151587940 | chr1 | 151587940 | 151587989 | –1.89583 | 2.03E–09 |
| *PGLYRP4* | CHR01FS151588654 | chr1 | 151588654 | 151588703 | –0.89635 | 6.76E–09 |
| *PGLYRP4* | CHR01FS151588860 | chr1 | 151588860 | 151588909 | –1.84807 | 8.75E–09 |
| *PGLYRP4* | CHR01FS151587740 | chr1 | 151587740 | 151587789 | –1.35251 | 3.37E–08 |
| *PGLYRP4* | CHR01FS151588950 | chr1 | 151588950 | 151588999 | –1.68278 | 6.05E–08 |
| *PGLYRP4* | CHR01FS151587838 | chr1 | 151587838 | 151587887 | –1.1627 | 2.38E–07 |
| *PGLYRP4* | CHR01FS151589160 | chr1 | 151589160 | 151589209 | –1.00239 | 3.92E–07 |
| *PGLYRP4* | CHR01FS151589048 | chr1 | 151589048 | 151589097 | –1.58134 | 4.71E–07 |
| *PIK3R5* | CHR17FS008756674 | chr17 | 8756674 | 8756723 | –0.7434 | 1.88E–08 |
| *PIK3R5* | CHR17FS008758872 | chr17 | 8758872 | 8758921 | –0.71925 | 1.04E–07 |
| *PIK3R5* | CHR17FS008811394 | chr17 | 8811394 | 8811445 | –1.16793 | 1.36E–07 |
| *PIK3R5* | CHR17FS008758452 | chr17 | 8758452 | 8758501 | –1.19877 | 1.43E–07 |
| *PIK3R5* | CHR17FS008758558 | chr17 | 8758558 | 8758607 | –1.15862 | 5.87E–07 |
| *PIK3R5* | CHR17FS008811700 | chr17 | 8811700 | 8811749 | –1.2198 | 6.89E–07 |
| *PIK3R5* | CHR17FS008756774 | chr17 | 8756774 | 8756823 | –1.10128 | 7.26E–07 |
| *PIK3R5* | CHR17FS008811798 | chr17 | 8811798 | 8811847 | –0.93672 | 8.62E–07 |
| *PIK3R5* | CHR17FS008811514 | chr17 | 8811514 | 8811563 | –1.26901 | 1.06E–06 |
| *PIK3R5* | CHR17FS008811602 | chr17 | 8811602 | 8811651 | –1.08551 | 1.07E–06 |
| *PLCB4* | CHR20FS009025302 | chr20 | 9025302 | 9025351 | –1.41388 | 4.06E–07 |
| *PPP2R3A* | CHR03FS137166887 | chr3 | 137166887 | 137166948 | 1.811771 | 6.43E–08 |
| *PRKAR1A* | CHR17FS064019220 | chr17 | 64019220 | 64019269 | 0.82587 | 1.9E–08 |
| *PRKAR1A* | CHR17FS064019138 | chr17 | 64019138 | 64019187 | 0.979657 | 2.2E–07 |
| *PRKAR1A* | CHR17FS064019037 | chr17 | 64019037 | 64019094 | 0.878994 | 3.34E–07 |
| *PRKCB* | CHR16FS023754104 | chr16 | 23754104 | 23754160 | 1.747457 | 7.18E–08 |
| *PRKCB* | CHR16FS023754330 | chr16 | 23754330 | 23754379 | 1.639834 | 3.47E–07 |
| *PRKCB* | CHR16FS023753988 | chr16 | 23753988 | 23754039 | 0.712345 | 5.56E–07 |
| *PRKCB* | CHR16FS023754424 | chr16 | 23754424 | 23754473 | 1.955496 | 1.2E–06 |
| *PTGER4* | CHR05FS040716473 | chr5 | 40716473 | 40716525 | 1.475918 | 6.09E–08 |
| *PTGER4* | CHR05FS040716649 | chr5 | 40716649 | 40716698 | 1.639984 | 8.73E–07 |
| *PTPN13* | CHR04FS087733870 | chr4 | 87733870 | 87733919 | 1.672533 | 5.5E–07 |
| *RASSF5* | CHR01FS204796198 | chr1 | 204796198 | 204796247 | –1.31774 | 2.97E–07 |
| *RFX4* | CHR12FS105499183 | chr12 | 105499183 | 105499232 | 1.365513 | 2.79E–08 |
| *S100A8* | CHR01FS151630474 | chr1 | 151630474 | 151630523 | –2.39857 | 4.74E–13 |
| *S100A8* | CHR01FS151630582 | chr1 | 151630582 | 151630631 | –2.27525 | 1.89E–12 |
| *S100A8* | CHR01FS151629888 | chr1 | 151629888 | 151629937 | –2.62589 | 2.48E–12 |
| *S100A8* | CHR01FS151629566 | chr1 | 151629566 | 151629615 | –2.22878 | 3.19E–12 |
| *S100A8* | CHR01FS151630368 | chr1 | 151630368 | 151630417 | –1.83167 | 3.75E–12 |
| *S100A8* | CHR01FS151629964 | chr1 | 151629964 | 151630013 | –2.58911 | 5.52E–12 |
| *S100A8* | CHR01FS151630064 | chr1 | 151630064 | 151630113 | –2.15252 | 6.41E–12 |
| *S100A8* | CHR01FS151629686 | chr1 | 151629686 | 151629735 | –2.24183 | 7.34E–12 |
| *S100A8* | CHR01FS151630166 | chr1 | 151630166 | 151630215 | –1.74501 | 8.31E–12 |
| *S100A8* | CHR01FS151629764 | chr1 | 151629764 | 151629813 | –2.33275 | 8.31E–12 |
| *S100A8* | CHR01FS151630274 | chr1 | 151630274 | 151630330 | –1.49773 | 3.91E–11 |
| *S100A8* | CHR01FS151630682 | chr1 | 151630682 | 151630733 | –1.36812 | 3.74E–10 |
| *S100A8* | CHR01FS151630968 | chr1 | 151630968 | 151631017 | –1.14132 | 4.67E–10 |
| *S100A8* | CHR01FS151630888 | chr1 | 151630888 | 151630937 | –1.07236 | 8.97E–07 |
| *SIGLEC10* | CHR19FS056614956 | chr19 | 56614956 | 56614956 | –1.0891 | 4.26E–08 |
| *SIGLEC10* | CHR19FS056613979 | chr19 | 56613979 | 56613979 | –0.97624 | 4.44E–08 |
| *SIGLEC10* | CHR19FS056615078 | chr19 | 56615078 | 56615078 | –1.28622 | 4.55E–08 |
| *SIGLEC10* | CHR19FS056613883 | chr19 | 56613883 | 56613883 | –1.18894 | 1.31E–07 |
| *SIGLEC10* | CHR19FS056612767 | chr19 | 56612767 | 56612767 | –0.98942 | 2.01E–07 |
| *SIGLEC10* | CHR19FS056614085 | chr19 | 56614085 | 56614085 | –1.11319 | 2.06E–07 |
| *SIGLEC10* | CHR19FS056615158 | chr19 | 56615158 | 56615158 | –1.14189 | 2.24E–07 |
| *SIGLEC10* | CHR19FS056614203 | chr19 | 56614203 | 56614203 | –0.8793 | 2.38E–07 |
| *SIGLEC10* | CHR19FS056613779 | chr19 | 56613779 | 56613779 | –1.02531 | 2.93E–07 |
| *SIGLEC5* | CHR19FS056825048 | chr19 | 56825048 | 56825097 | –1.03686 | 5.36E–08 |
| *SIGLEC5* | CHR19FS056825230 | chr19 | 56825230 | 56825279 | –0.81789 | 7.48E–07 |
| *SIGLEC7* | CHR19FS056337335 | chr19 | 56337335 | 56337384 | –1.57902 | 2E–08 |
| *SIGLEC7* | CHR19FS056336429 | chr19 | 56336429 | 56336478 | –0.98681 | 2.13E–08 |
| *SIGLEC7* | CHR19FS056337253 | chr19 | 56337253 | 56337302 | –1.45533 | 2.54E–08 |
| *SIGLEC7* | CHR19FS056337741 | chr19 | 56337741 | 56337790 | –1.60772 | 5.07E–08 |
| *SIGLEC7* | CHR19FS056337835 | chr19 | 56337835 | 56337884 | –1.57757 | 6.29E–08 |
| *SIGLEC7* | CHR19FS056337631 | chr19 | 56337631 | 56337680 | –1.39896 | 1.78E–07 |
| *SIGLEC7* | CHR19FS056336831 | chr19 | 56336831 | 56336880 | –0.83669 | 1.91E–07 |
| *SIGLEC7* | CHR19FS056336553 | chr19 | 56336553 | 56336602 | –1.62194 | 5.61E–07 |
| *SIGLEC7* | CHR19FS056337943 | chr19 | 56337943 | 56337992 | –0.93107 | 8.91E–07 |
| *SIRPB1* | CHR20FS001548601 | chr20 | 1548601 | 1548650 | –0.90595 | 7.06E–08 |
| *SIRPB1* | CHR20FS001548803 | chr20 | 1548803 | 1548852 | –1.03761 | 2.67E–07 |
| *SIRPG* | CHR20FS001586824 | chr20 | 1586824 | 1586873 | –1.16382 | 1.58E–08 |
| *SIRPG* | CHR20FS001586734 | chr20 | 1586734 | 1586783 | –0.71979 | 3.1E–08 |
| *SLA2* | CHR20FS034708563 | chr20 | 34708563 | 34708612 | –1.07588 | 5.73E–08 |
| *SLC3A2* | CHR11FS062403446 | chr11 | 62403446 | 62403495 | –0.9005 | 1.23E–10 |
| *SOCS2* | CHR12FS092490556 | chr12 | 92490556 | 92490605 | 1.091051 | 1.78E–07 |
| *SPTAN1* | CHR09FS130354146 | chr9 | 130354146 | 130354195 | –0.68917 | 4.02E–07 |
| *SYK* | CHR09FS092601614 | chr9 | 92601614 | 92601668 | –0.87745 | 5.37E–08 |
| *SYK* | CHR09FS092601508 | chr9 | 92601508 | 92601564 | –0.79267 | 1.28E–07 |
| *SYK* | CHR09FS092601792 | chr9 | 92601792 | 92601841 | –1.29288 | 2.12E–07 |
| *TBX21* | CHR17FS043165617 | chr17 | 43165617 | 43165666 | –0.66041 | 4.73E–07 |
| *TBX21* | CHR17FS043163584 | chr17 | 43163584 | 43163633 | –0.70227 | 5.02E–07 |
| *TBX21* | CHR17FS043163368 | chr17 | 43163368 | 43163417 | –0.78869 | 1.15E–06 |
| *TRAF5* | CHR01FS209583903 | chr1 | 209583903 | 209583952 | –0.97156 | 9.88E–07 |
| *TREM1* | CHR06FS041362272 | chr6 | 41362272 | 41362321 | –1.2224 | 1.31E–09 |
| *TREM1* | CHR06FS041362450 | chr6 | 41362450 | 41362499 | –1.00661 | 1.36E–07 |
| *TREM2* | CHR06FS041238391 | chr6 | 41238391 | 41238440 | –2.07437 | 7.03E–10 |
| *TREM2* | CHR06FS041238701 | chr6 | 41238701 | 41238750 | –1.56547 | 4.29E–09 |
| *TREM2* | CHR06FS041238493 | chr6 | 41238493 | 41238542 | –1.78712 | 1.52E–08 |
| *TREM2* | CHR06FS041238315 | chr6 | 41238315 | 41238364 | –1.55155 | 1.71E–08 |
| *TREM2* | CHR06FS041240191 | chr6 | 41240191 | 41240242 | –0.86754 | 5.28E–08 |
| *TREM2* | CHR06FS041239915 | chr6 | 41239915 | 41239964 | –0.84053 | 3.75E–07 |
| *TREM2* | CHR06FS041240009 | chr6 | 41240009 | 41240058 | –1.12843 | 6.64E–07 |
| *TRGV9* | CHR07FS038317301 | chr7 | 38317301 | 38317301 | –0.6697 | 8.96E–07 |

*Probe ID in the Nimblegen 3×720 K CpG Island Plus RefSeq Promoter Array.

† NCBI36/hg18.

‡ Paired Student's *t*-test.

Abbreviations: Chr., chromosome; FC, log_2_(Fold changes), HCCs vs. adjacent non-tumor tissues.
