# Supplementary material for: Relatively Small Contribution of Methylation and Genomic Copy Number Aberration to the Aberrant Expression of Inflammation-Related Genes in HBV-Related Hepatocellular Carcinoma
Source: PLoS One. 2015 May 12;10(5):e0126836. doi: 10.1371/journal.pone.0126836 (PMC4429029; doi:10.1371/journal.pone.0126836)
Supplement: S5 Table — (DOC) [file pone.0126836.s007.doc]

**S5 Table. Inflammation-related genes with SCNAs in HCC**

| **Symbol** | **Probe ID*** | **Chr.** | **Position**† | **FC** | **P Value**‡ |
| --- | --- | --- | --- | --- | --- |
| *ABL1* | CN_1303851 | Chr9 | 132713149 | –0.50995 | 4.90794E–09 |
| *ABL1* | CN_1303764 | Chr9 | 132632921 | –0.50188 | 1.62809E–08 |
| *ABL1* | CN_1303850 | Chr9 | 132713318 | –0.44696 | 1.9246E–08 |
| *ABL1* | CN_1303762 | Chr9 | 132632870 | –1.09964 | 1.33859E–07 |
| *ABL1* | CN_1303763 | Chr9 | 132632886 | –0.42828 | 1.84415E–07 |
| *ABL1* | CN_382930 | Chr9 | 132713198 | –0.39741 | 6.56043E–07 |
| *ABL1* | CN_1303849 | Chr9 | 132713295 | –0.39783 | 1.33E–06 |
| *ACE* | SNP_A-8557538 | Chr17 | 58916903 | 0.516217 | 7.44637E–11 |
| *ACE* | SNP_A-8560002 | Chr17 | 58916932 | 0.59167 | 2.24323E–09 |
| *ACE* | SNP_A-2257238 | Chr17 | 58909398 | 0.663523 | 7.47333E–07 |
| *ACIN1* | CN_640282 | Chr14 | 22632346 | –0.72678 | 1.66102E–12 |
| *ACIN1* | CN_640283 | Chr14 | 22633588 | –0.51084 | 3.47318E–07 |
| *ADCY1* | CN_1240570 | Chr7 | 45596658 | –0.90094 | 8.64741E–16 |
| *ADCY1* | CN_1240572 | Chr7 | 45599855 | –0.40324 | 2.18327E–07 |
| *ADCY2* | CN_1128613 | Chr5 | 7494686 | –0.79346 | 2.46067E–09 |
| *ADCY2* | SNP_A-4299019 | Chr5 | 7720153 | –0.5078 | 1.28953E–08 |
| *ADCY2* | CN_304882 | Chr5 | 7450750 | –0.61208 | 1.77426E–08 |
| *ADCY2* | SNP_A-1869624 | Chr5 | 7495910 | –0.70306 | 6.63323E–08 |
| *ADCY2* | SNP_A-2222753 | Chr5 | 7452053 | –0.45718 | 2.3136E–07 |
| *ADCY2* | SNP_A-2159648 | Chr5 | 7719786 | –0.3797 | 7.72119E–07 |
| *ADCY4* | CN_646695 | Chr14 | 23869621 | –0.57416 | 6.23628E–08 |
| *ADCY4* | SNP_A-8611039 | Chr14 | 23869198 | –0.31947 | 1.33E–06 |
| *ADCY5* | CN_1010596 | Chr3 | 124617294 | –1.00939 | 7.095E–15 |
| *ADCY5* | SNP_A-2167657 | Chr3 | 124617500 | –0.65569 | 1.61277E–13 |
| *ADCY5* | CN_1010595 | Chr3 | 124616400 | –1.0054 | 3.91838E–12 |
| *ADCY5* | SNP_A-2263519 | Chr3 | 124611103 | –0.64857 | 1.38397E–10 |
| *ADCY5* | CN_1010594 | Chr3 | 124612682 | –0.48711 | 2.70225E–08 |
| *ADRBK1* | CN_579095 | Chr11 | 66795277 | –0.4275 | 1.46427E–09 |
| *ADRBK1* | CN_579094 | Chr11 | 66792577 | –0.58329 | 2.86846E–08 |
| *ADRBK1* | SNP_A-8318334 | Chr11 | 66795188 | –0.35315 | 4.03938E–07 |
| *AKR1C3* | SNP_A-8385807 | Chr10 | 5139144 | 0.682423 | 5.12622E–10 |
| *AKR1C3* | SNP_A-8684879 | Chr10 | 5138453 | 0.62171 | 2.13537E–07 |
| *AKT2* | CN_796163 | Chr19 | 45473212 | –0.64673 | 1.15527E–11 |
| *AKT2* | CN_796145 | Chr19 | 45455639 | 0.536583 | 2.38388E–09 |
| *AKT2* | CN_796151 | Chr19 | 45468000 | –0.54876 | 3.26184E–09 |
| *AKT2* | CN_796185 | Chr19 | 45480968 | –0.75123 | 1.92663E–08 |
| *AKT2* | CN_796144 | Chr19 | 45455691 | 0.723967 | 2.05964E–08 |
| *AKT2* | CN_796162 | Chr19 | 45473195 | –0.72369 | 3.65115E–08 |
| *AKT2* | CN_796186 | Chr19 | 45480902 | –0.5249 | 1.16395E–07 |
| *AKT2* | SNP_A-8538112 | Chr19 | 45480481 | –0.5905 | 1.27718E–07 |
| *AKT2* | CN_796159 | Chr19 | 45472935 | –0.51752 | 2.8366E–07 |
| *AKT2* | CN_796150 | Chr19 | 45467992 | –0.49354 | 9.3554E–07 |
| *AKT3* | CN_484189 | Chr1 | 241832554 | 1.656867 | 1.01434E–15 |
| *AKT3* | CN_484190 | Chr1 | 241832598 | 1.228803 | 2.41542E–15 |
| *AKT3* | CN_484235 | Chr1 | 241909555 | 1.005573 | 1.59185E–13 |
| *AKT3* | CN_484263 | Chr1 | 241947568 | 1.087253 | 6.23254E–12 |
| *AKT3* | CN_484230 | Chr1 | 241898499 | 0.685177 | 1.08004E–11 |
| *AKT3* | CN_484302 | Chr1 | 242008275 | 1.078847 | 1.94425E–11 |
| *AKT3* | CN_484255 | Chr1 | 241932870 | 1.19065 | 4.84081E–11 |
| *AKT3* | CN_484231 | Chr1 | 241900711 | 1.035317 | 8.94238E–11 |
| *AKT3* | CN_486419 | Chr1 | 242064203 | 0.8493 | 4.85942E–09 |
| *AKT3* | CN_482104 | Chr1 | 241818523 | 0.61062 | 1.14897E–08 |
| *AKT3* | SNP_A-4266586 | Chr1 | 241952471 | 0.686943 | 1.57427E–08 |
| *AKT3* | CN_484256 | Chr1 | 241936491 | 0.636093 | 1.8028E–08 |
| *AKT3* | CN_484234 | Chr1 | 241900816 | 0.527257 | 2.22754E–08 |
| *AKT3* | CN_484262 | Chr1 | 241947551 | 0.91157 | 3.65436E–08 |
| *AKT3* | SNP_A-8314212 | Chr1 | 242009707 | 0.58237 | 9.24677E–08 |
| *AKT3* | CN_019644 | Chr1 | 241822426 | 0.70891 | 1.04452E–07 |
| *AKT3* | CN_486420 | Chr1 | 242064210 | 0.597367 | 1.14258E–07 |
| *AKT3* | CN_486421 | Chr1 | 242064877 | 0.825303 | 2.45206E–07 |
| *AKT3* | CN_486418 | Chr1 | 242064170 | 0.696297 | 7.37665E–07 |
| *AKT3* | CN_484268 | Chr1 | 241950752 | 0.4719 | 8.42206E–07 |
| *ALCAM* | SNP_A-2308694 | Chr3 | 106745798 | 1.13826 | 4.67127E–14 |
| *ALCAM* | SNP_A-4252112 | Chr3 | 106746314 | 0.692553 | 3.04823E–10 |
| *ALOX5* | SNP_A-8502850 | Chr10 | 45237382 | –0.70059 | 1.49356E–13 |
| *ALOX5* | SNP_A-8453895 | Chr10 | 45237098 | –0.44856 | 4.95494E–08 |
| *ALOX5AP* | CN_660312 | Chr13 | 30216199 | –1.06657 | 3.84883E–12 |
| *ALOX5AP* | SNP_A-2296939 | Chr13 | 30216308 | –0.71648 | 1.08534E–11 |
| *ALOX5AP* | SNP_A-8307140 | Chr13 | 30217133 | –0.9278 | 5.64783E–10 |
| *ALS2* | SNP_A-8464802 | Chr2 | 202312920 | 0.43829 | 6.46067E–11 |
| *ALS2* | SNP_A-2131409 | Chr2 | 202305680 | 0.48628 | 4.13239E–09 |
| *ALS2* | CN_816395 | Chr2 | 202311658 | 0.452177 | 5.72085E–08 |
| *ALS2* | CN_816394 | Chr2 | 202301380 | 0.581277 | 4.13414E–07 |
| *ARAF* | CN_966722 | ChrX | 47313352 | 0.92838 | 1.79435E–12 |
| *ARAF* | SNP_A-8391560 | ChrX | 47312538 | 0.632563 | 3.43653E–10 |
| *ARAF* | CN_966721 | ChrX | 47311750 | 0.75933 | 2.08269E–08 |
| *ATF1* | CN_594362 | Chr12 | 49479428 | 0.799443 | 5.0368E–10 |
| *ATF1* | CN_594363 | Chr12 | 49481553 | 0.596687 | 2.80503E–07 |
| *ATF3* | CN_483602 | Chr1 | 210823122 | 1.382923 | 1.16332E–14 |
| *ATF3* | SNP_A-8707068 | Chr1 | 210825104 | 0.834207 | 9.45352E–10 |
| *B3GAT1* | SNP_A-8371326 | Chr11 | 133756462 | –0.94914 | 4.02576E–17 |
| *B3GAT1* | SNP_A-2174155 | Chr11 | 133760600 | –0.45467 | 1.16E–06 |
| *BAD* | SNP_A-8692850 | Chr11 | 63803448 | 0.679703 | 9.5393E–12 |
| *BAD* | SNP_A-4276342 | Chr11 | 63803461 | 0.687153 | 3.57817E–09 |
| *BBC3* | CN_798488 | Chr19 | 52427494 | 1.1366 | 1.61794E–09 |
| *BBC3* | CN_798485 | Chr19 | 52423922 | 0.849087 | 1.06483E–08 |
| *BBC3* | CN_798486 | Chr19 | 52424862 | 0.430857 | 1.47E–06 |
| *BCAR1* | SNP_A-2291033 | Chr16 | 73821162 | –0.87511 | 6.33758E–11 |
| *BCAR1* | SNP_A-8451682 | Chr16 | 73823220 | –0.80913 | 6.35415E–11 |
| *BCAR1* | SNP_A-8467141 | Chr16 | 73824108 | –0.74305 | 8.94695E–09 |
| *BCL2* | CN_771138 | Chr18 | 59131352 | –1.07584 | 2.63218E–16 |
| *BCL2* | SNP_A-1919985 | Chr18 | 59129566 | –0.45732 | 9.19856E–11 |
| *BCL2* | SNP_A-8557928 | Chr18 | 59111959 | –0.69806 | 6.60232E–10 |
| *BCL2* | CN_768935 | Chr18 | 58983622 | –0.50326 | 1.61238E–09 |
| *BCL2* | CN_768934 | Chr18 | 58983396 | –0.56896 | 4.35221E–07 |
| *BCL2* | CN_769062 | Chr18 | 59112327 | –0.49574 | 8.95035E–07 |
| *BCL2L1* | SNP_A-1803760 | Chr20 | 29751357 | –0.81507 | 3.07206E–11 |
| *BCL2L1* | CN_894219 | Chr20 | 29751068 | –0.88616 | 2.02608E–10 |
| *BIRC2* | SNP_A-4205329 | Chr11 | 101742930 | –0.49419 | 7.54148E–11 |
| *BIRC2* | CN_557747 | Chr11 | 101726789 | –0.45222 | 4.9651E–09 |
| *BLNK* | SNP_A-4229992 | Chr10 | 97992856 | 0.90453 | 5.41236E–13 |
| *BLNK* | SNP_A-1848118 | Chr10 | 97992875 | 0.987137 | 1.43101E–11 |
| *BLNK* | SNP_A-8580163 | Chr10 | 97992222 | 0.852597 | 1.37743E–09 |
| *BLNK* | CN_534091 | Chr10 | 97992590 | 0.385643 | 1.40603E–06 |
| *BMPR1B* | CN_1107915 | Chr4 | 96133396 | –0.85172 | 1.41145E–12 |
| *BMPR1B* | SNP_A-4210372 | Chr4 | 96110096 | –0.68723 | 1.13236E–11 |
| *BMPR1B* | CN_1107904 | Chr4 | 96089289 | –0.8804 | 3.30206E–11 |
| *BMPR1B* | CN_1107927 | Chr4 | 96167299 | –0.75181 | 9.03457E–11 |
| *BMPR1B* | CN_282569 | Chr4 | 96115426 | –0.60787 | 7.21177E–10 |
| *BMPR1B* | CN_1107936 | Chr4 | 96189080 | –0.72418 | 7.63269E–10 |
| *BMPR1B* | SNP_A-8459676 | Chr4 | 96115367 | –0.99061 | 1.00487E–09 |
| *BMPR1B* | SNP_A-8502118 | Chr4 | 96165529 | –0.58257 | 7.63712E–09 |
| *BMPR1B* | CN_1107903 | Chr4 | 96088419 | –0.82488 | 4.24148E–08 |
| *BMPR1B* | SNP_A-4278630 | Chr4 | 96133421 | –0.61984 | 2.70905E–07 |
| *BMPR1B* | CN_1107935 | Chr4 | 96188191 | –0.85811 | 3.35016E–07 |
| *BMPR1B* | CN_1107913 | Chr4 | 96116552 | –0.46899 | 3.80531E–07 |
| *BMPR2* | CN_820701 | Chr2 | 202998278 | –0.8431 | 1.78505E–15 |
| *BMPR2* | SNP_A-2131436 | Chr2 | 202998258 | –0.54981 | 1.31711E–08 |
| *BRAF* | CN_340348 | Chr7 | 140240554 | 0.95053 | 1.26237E–15 |
| *BRAF* | CN_1212565 | Chr7 | 140157648 | 1.008957 | 2.82327E–13 |
| *BRAF* | CN_1214732 | Chr7 | 140260522 | 1.032953 | 3.94868E–13 |
| *BRAF* | CN_1212569 | Chr7 | 140157602 | 0.881897 | 2.56249E–12 |
| *BRAF* | CN_1212566 | Chr7 | 140157677 | 0.879277 | 5.66663E–12 |
| *BRAF* | CN_1212654 | Chr7 | 140240502 | 0.76836 | 6.33941E–09 |
| *BRAF* | CN_1212655 | Chr7 | 140240542 | 0.563553 | 1.21679E–08 |
| *BRAF* | CN_1212646 | Chr7 | 140233091 | 0.921733 | 2.03866E–08 |
| *BRAF* | CN_1212588 | Chr7 | 140178158 | 0.577973 | 6.70398E–08 |
| *BRAF* | CN_1212587 | Chr7 | 140178127 | 0.53434 | 1.17754E–07 |
| *BRAF* | CN_1212653 | Chr7 | 140240486 | 0.65235 | 3.95798E–07 |
| *BRAF* | CN_1214731 | Chr7 | 140260261 | 0.6717 | 5.58781E–07 |
| *BRAF* | CN_1212647 | Chr7 | 140233109 | 0.686943 | 7.32544E–07 |
| *BTRC* | CN_511970 | Chr10 | 103210604 | –1.11437 | 3.4138E–13 |
| *BTRC* | CN_032888 | Chr10 | 103205111 | –0.66852 | 7.32501E–11 |
| *BTRC* | CN_511967 | Chr10 | 103205507 | –0.52438 | 5.8399E–08 |
| *C1QB* | SNP_A-1936572 | Chr1 | 22854463 | –0.67928 | 7.75763E–15 |
| *C1QB* | CN_479526 | Chr1 | 22854434 | –0.62334 | 3.74438E–09 |
| *C5* | CN_1319333 | Chr9 | 122809550 | 0.63433 | 1.85393E–08 |
| *C5* | SNP_A-2035799 | Chr9 | 122809663 | 0.405447 | 1.23931E–07 |
| *C8A* | SNP_A-1910012 | Chr1 | 57152280 | –1.06579 | 1.05329E–18 |
| *C8A* | SNP_A-1888688 | Chr1 | 57152620 | –0.54431 | 4.55368E–10 |
| *C8A* | CN_484686 | Chr1 | 57115915 | –0.59518 | 2.07704E–07 |
| *C8A* | SNP_A-1854202 | Chr1 | 57115844 | –0.58674 | 2.92978E–07 |
| *C8B* | CN_484718 | Chr1 | 57190592 | –0.83199 | 3.73043E–14 |
| *C8B* | CN_484717 | Chr1 | 57187389 | –0.58894 | 1.26169E–09 |
| *CABIN1* | SNP_A-4292461 | Chr22 | 22883535 | –0.97188 | 1.01854E–12 |
| *CABIN1* | CN_915345 | Chr22 | 22832919 | –0.94726 | 2.0938E–10 |
| *CABIN1* | CN_917430 | Chr22 | 22880382 | –0.49933 | 1.37375E–08 |
| *CABIN1* | CN_915346 | Chr22 | 22837381 | –0.53494 | 2.13814E–07 |
| *CABIN1* | CN_915300 | Chr22 | 22765294 | –0.56112 | 2.25689E–07 |
| *CABIN1* | CN_915299 | Chr22 | 22763384 | –0.49837 | 7.1934E–07 |
| *CAMK1D* | SNP_A-8534321 | Chr10 | 12622719 | 0.718723 | 7.90538E–10 |
| *CAMK1D* | SNP_A-2048907 | Chr10 | 12508143 | 0.543563 | 8.47738E–10 |
| *CAMK1D* | SNP_A-8607420 | Chr10 | 12622695 | 0.818943 | 1.60637E–09 |
| *CAMK1D* | SNP_A-8471273 | Chr10 | 12504979 | 0.7042 | 5.75569E–09 |
| *CAMK1D* | SNP_A-8478972 | Chr10 | 12577095 | 0.699553 | 3.23624E–08 |
| *CAMK1D* | SNP_A-8652923 | Chr10 | 12622525 | 0.671577 | 4.12303E–08 |
| *CAMK1D* | SNP_A-8633091 | Chr10 | 12574304 | 0.62919 | 5.39457E–08 |
| *CAMK1D* | CN_508123 | Chr10 | 12575045 | 0.75768 | 6.42966E–08 |
| *CAMK1D* | SNP_A-8534490 | Chr10 | 12873618 | 0.539797 | 1.26399E–07 |
| *CAMK1D* | CN_490564 | Chr10 | 12873111 | 0.665463 | 1.27254E–07 |
| *CAMK1D* | CN_503753 | Chr10 | 12508207 | 0.521363 | 3.93858E–07 |
| *CAMK1D* | SNP_A-8394021 | Chr10 | 12505710 | 0.660633 | 6.5324E–07 |
| *CAMK1D* | SNP_A-8543459 | Chr10 | 12577433 | 0.742693 | 7.28127E–07 |
| *CAMK4* | CN_1088471 | Chr5 | 110709308 | –0.90696 | 4.63845E–13 |
| *CAMK4* | SNP_A-1982919 | Chr5 | 110707772 | –0.56786 | 2.50421E–11 |
| *CAPN1* | SNP_A-8680521 | Chr11 | 64719534 | 1.119087 | 2.63183E–10 |
| *CAPN1* | CN_570414 | Chr11 | 64729754 | 0.59105 | 3.07284E–07 |
| *CAPN10* | SNP_A-8475458 | Chr2 | 241181703 | –0.8403 | 1.33157E–13 |
| *CAPN10* | CN_852525 | Chr2 | 241192759 | –0.55902 | 1.56831E–13 |
| *CAPN10* | CN_852523 | Chr2 | 241192678 | –0.80031 | 6.76241E–10 |
| *CAPN10* | SNP_A-8647694 | Chr2 | 241181631 | –0.6285 | 1.78707E–09 |
| *CAPN10* | CN_852524 | Chr2 | 241192694 | –0.34659 | 1.99581E–07 |
| *CARD14* | SNP_A-8288743 | Chr17 | 75790078 | 0.917483 | 4.9117E–09 |
| *CARD14* | CN_753115 | Chr17 | 75788521 | 0.511097 | 1.15899E–07 |
| *CASP1* | CN_538605 | Chr11 | 104402072 | –0.86564 | 2.41345E–10 |
| *CASP1* | CN_538607 | Chr11 | 104405479 | –0.66982 | 1.65157E–09 |
| *CASP1* | CN_540632 | Chr11 | 104411076 | –0.7177 | 1.69646E–09 |
| *CASP1* | CN_538606 | Chr11 | 104401936 | –0.72605 | 1.2764E–07 |
| *CASP1* | SNP_A-8339325 | Chr11 | 104416380 | –0.42477 | 2.65229E–07 |
| *CASP7* | SNP_A-8350851 | Chr10 | 115458798 | –0.86821 | 7.02094E–16 |
| *CASP7* | SNP_A-8630771 | Chr10 | 115480050 | –0.94016 | 2.07212E–13 |
| *CASP7* | SNP_A-2258969 | Chr10 | 115479640 | –0.64531 | 6.88296E–13 |
| *CASP7* | SNP_A-8289641 | Chr10 | 115461551 | –0.78992 | 4.58619E–12 |
| *CASP7* | CN_516542 | Chr10 | 115459028 | –0.79763 | 8.44591E–11 |
| *CASP7* | SNP_A-2005532 | Chr10 | 115461763 | –0.7678 | 6.33524E–10 |
| *CASP7* | SNP_A-8359721 | Chr10 | 115447879 | –0.61002 | 4.15298E–07 |
| *CASP7* | CN_516541 | Chr10 | 115445057 | –0.46824 | 7.38088E–07 |
| *CCBP2* | SNP_A-8646553 | Chr3 | 42849114 | 0.605377 | 3.23789E–07 |
| *CCBP2* | SNP_A-8694952 | Chr3 | 42858469 | 0.398333 | 1.39E–06 |
| *CD101* | SNP_A-2243834 | Chr1 | 117369049 | –0.86948 | 9.96699E–15 |
| *CD101* | CN_437420 | Chr1 | 117376625 | –0.7617 | 2.53099E–10 |
| *CD101* | CN_437417 | Chr1 | 117363764 | –0.68215 | 2.84507E–10 |
| *CD180* | SNP_A-2312753 | Chr5 | 66519502 | –0.59808 | 1.68333E–08 |
| *CD180* | CN_1126314 | Chr5 | 66517292 | –0.54209 | 6.03249E–08 |
| *CD2* | SNP_A-8403711 | Chr1 | 117106599 | –1.41554 | 1.41448E–19 |
| *CD2* | CN_436242 | Chr1 | 117107015 | –0.59297 | 1.31139E–06 |
| *CD244* | SNP_A-8446667 | Chr1 | 159070359 | 1.16652 | 5.77649E–12 |
| *CD244* | SNP_A-8319977 | Chr1 | 159070342 | 0.900527 | 2.91362E–10 |
| *CD244* | SNP_A-1795937 | Chr1 | 159070426 | 0.39352 | 2.18341E–07 |
| *CD40LG* | SNP_A-8455615 | ChrX | 135566928 | 1.41898 | 1.41961E–18 |
| *CD40LG* | SNP_A-8640601 | ChrX | 135567338 | 0.614847 | 5.03811E–10 |
| *CD44* | SNP_A-8333643 | Chr11 | 35118246 | 0.46103 | 1.72871E–09 |
| *CD44* | SNP_A-2009785 | Chr11 | 35120062 | 0.503047 | 1.93685E–07 |
| *CD48* | SNP_A-8579347 | Chr1 | 158920690 | 1.069847 | 4.84405E–14 |
| *CD48* | CN_436688 | Chr1 | 158946330 | 1.337403 | 8.0392E–12 |
| *CD48* | CN_436658 | Chr1 | 158920390 | 1.117367 | 3.33063E–10 |
| *CD48* | CN_436659 | Chr1 | 158920458 | 0.716447 | 3.02786E–09 |
| *CD48* | CN_436689 | Chr1 | 158946370 | 0.957607 | 3.6341E–09 |
| *CD48* | SNP_A-2209370 | Chr1 | 158919354 | 0.488803 | 2.66534E–08 |
| *CD48* | CN_436655 | Chr1 | 158920233 | 0.441607 | 5.94263E–07 |
| *CD53* | SNP_A-1961816 | Chr1 | 111235145 | 0.6529 | 3.49945E–12 |
| *CD53* | CN_437344 | Chr1 | 111234982 | 0.708017 | 1.00503E–06 |
| *CD55* | CN_454780 | Chr1 | 205599580 | 0.755927 | 1.12909E–10 |
| *CD55* | SNP_A-1791550 | Chr1 | 205575988 | 0.591683 | 1.59732E–08 |
| *CD55* | CN_454771 | Chr1 | 205574165 | 0.57122 | 3.30513E–08 |
| *CD55* | SNP_A-8550725 | Chr1 | 205593908 | 0.480557 | 1.06783E–06 |
| *CD9* | SNP_A-2200897 | Chr12 | 6210649 | –0.79573 | 8.90245E–10 |
| *CD9* | CN_594706 | Chr12 | 6210410 | –0.53261 | 8.03981E–07 |
| *CD96* | SNP_A-1781345 | Chr3 | 112846428 | 0.64777 | 3.40933E–12 |
| *CD96* | CN_984269 | Chr3 | 112848511 | 0.79199 | 1.514E–10 |
| *CD96* | SNP_A-8685944 | Chr3 | 112767824 | 0.769973 | 5.64891E–09 |
| *CD96* | CN_984234 | Chr3 | 112766357 | 0.51689 | 6.49115E–08 |
| *CD99* | SNP_A-8572264 | ChrX | 2655366 | 0.822777 | 2.58399E–09 |
| *CD99* | SNP_A-8573942 | ChrX | 2663552 | 0.85365 | 1.07949E–08 |
| *CDK1* | CN_539712 | Chr10 | 62209928 | –0.64757 | 1.1853E–09 |
| *CDK1* | SNP_A-2246005 | Chr10 | 62210069 | –0.59483 | 4.67296E–08 |
| *CKLF* | SNP_A-4240547 | Chr16 | 65150380 | 0.649547 | 9.08111E–10 |
| *CKLF* | SNP_A-2084653 | Chr16 | 65148073 | 0.798387 | 3.86011E–09 |
| *CKLF* | CN_702974 | Chr16 | 65150413 | 0.523067 | 4.41635E–07 |
| *CR1* | SNP_A-8461276 | Chr1 | 205825167 | 0.705677 | 1.58421E–13 |
| *CR1* | SNP_A-2146758 | Chr1 | 205737892 | 0.472847 | 1.39998E–09 |
| *CR1* | CN_456950 | Chr1 | 205834284 | 0.956617 | 1.70759E–09 |
| *CR1* | CN_456949 | Chr1 | 205829194 | 0.809997 | 2.14447E–08 |
| *CR1* | SNP_A-4238870 | Chr1 | 205737551 | 0.759093 | 2.66717E–07 |
| *CR2* | CN_454819 | Chr1 | 205706754 | 0.60834 | 5.1881E–08 |
| *CR2* | CN_454821 | Chr1 | 205708194 | 1.054743 | 8.36446E–08 |
| *CR2* | CN_454820 | Chr1 | 205707770 | 0.399733 | 1.15E–06 |
| *CRADD* | SNP_A-8500983 | Chr12 | 92726783 | 0.799327 | 1.12242E–11 |
| *CRADD* | SNP_A-2057103 | Chr12 | 92704882 | 0.77233 | 7.58918E–11 |
| *CRADD* | CN_620919 | Chr12 | 92705184 | 0.746677 | 6.21483E–08 |
| *CRADD* | SNP_A-2212038 | Chr12 | 92731325 | 0.728307 | 8.6884E–08 |
| *CREB1* | SNP_A-1865785 | Chr2 | 208132792 | 0.864253 | 1.13294E–15 |
| *CREB1* | CN_189036 | Chr2 | 208130572 | 0.7778 | 2.18092E–08 |
| *CREBBP* | SNP_A-8530964 | Chr16 | 3756551 | 0.89717 | 3.63785E–13 |
| *CREBBP* | SNP_A-8304899 | Chr16 | 3767554 | 0.733867 | 7.02E–10 |
| *CREBBP* | CN_715535 | Chr16 | 3757679 | 0.63875 | 2.03894E–07 |
| *CSF1R* | SNP_A-1784305 | Chr5 | 149467961 | 0.944843 | 1.25007E–13 |
| *CSF1R* | SNP_A-2296455 | Chr5 | 149469303 | 1.00272 | 4.44823E–12 |
| *CSF1R* | SNP_A-8509028 | Chr5 | 149468103 | 0.853807 | 1.32822E–11 |
| *CSF1R* | SNP_A-1789093 | Chr5 | 149468134 | 0.73828 | 9.40021E–09 |
| *CSF2RB* | SNP_A-4279148 | Chr22 | 35642119 | 1.10616 | 8.55804E–13 |
| *CSF2RB* | SNP_A-2044385 | Chr22 | 35641823 | 1.05866 | 4.75569E–11 |
| *CSF3R* | SNP_A-8547982 | Chr1 | 36714338 | –0.67998 | 8.9372E–11 |
| *CSF3R* | SNP_A-8671956 | Chr1 | 36710001 | –0.53701 | 1.92987E–07 |
| *CSF3R* | CN_456116 | Chr1 | 36711517 | –0.53022 | 1.29647E–06 |
| *CSK* | SNP_A-2303951 | Chr15 | 72864420 | –0.59552 | 1.33916E–08 |
| *CSK* | CN_697098 | Chr15 | 72865261 | –0.48091 | 1.19255E–07 |
| *CTNNB1* | CN_1037308 | Chr3 | 41230105 | –0.87323 | 8.17432E–11 |
| *CTNNB1* | CN_1039488 | Chr3 | 41254863 | –0.77308 | 1.4978E–10 |
| *CTNNB1* | CN_1039486 | Chr3 | 41254825 | –0.5603 | 2.68209E–09 |
| *CTNNB1* | CN_1037303 | Chr3 | 41230227 | –0.59019 | 4.76918E–09 |
| *CTNNB1* | CN_1037324 | Chr3 | 41236724 | –0.74426 | 1.15218E–07 |
| *CTNNB1* | CN_1037322 | Chr3 | 41236542 | –0.7123 | 6.28754E–07 |
| *CTSS* | CN_446683 | Chr1 | 148987890 | 0.785603 | 7.9102E–08 |
| *CTSS* | CN_446684 | Chr1 | 148989419 | 0.573937 | 5.67298E–07 |
| *CTTN* | CN_592162 | Chr11 | 69951246 | 0.67754 | 1.26912E–08 |
| *CTTN* | CN_592163 | Chr11 | 69951574 | 0.63498 | 1.62431E–08 |
| *CTTN* | SNP_A-8319439 | Chr11 | 69951839 | 0.76741 | 1.68774E–08 |
| *CX3CL1* | SNP_A-8369253 | Chr16 | 55975689 | –1.02829 | 1.63883E–14 |
| *CX3CL1* | CN_715877 | Chr16 | 55975840 | –0.53978 | 1.08733E–07 |
| *CXCL11* | SNP_A-1977658 | Chr4 | 77178648 | 1.040013 | 3.62807E–15 |
| *CXCL11* | SNP_A-2136438 | Chr4 | 77177358 | 0.900187 | 1.65698E–12 |
| *CXCL12* | SNP_A-8601697 | Chr10 | 44188263 | –0.50836 | 7.45878E–11 |
| *CXCL12* | SNP_A-2046007 | Chr10 | 44190021 | –0.69345 | 1.23079E–10 |
| *CXCL12* | CN_550071 | Chr10 | 44187841 | –0.74165 | 2.9461E–10 |
| *CXCL12* | CN_550070 | Chr10 | 44187830 | –0.51083 | 2.39788E–08 |
| *CXCL12* | CN_550072 | Chr10 | 44190048 | –0.50297 | 1.64678E–07 |
| *CXCL13* | CN_1087730 | Chr4 | 78679849 | –0.8006 | 3.14439E–12 |
| *CXCL13* | CN_1087749 | Chr4 | 78747958 | –0.6826 | 2.33659E–09 |
| *CXCL13* | CN_1087729 | Chr4 | 78679651 | –0.75645 | 4.62148E–09 |
| *CXCL13* | SNP_A-1854960 | Chr4 | 78675526 | –0.80793 | 2.42994E–08 |
| *CXCL13* | CN_1087751 | Chr4 | 78750520 | –0.55753 | 1.38817E–07 |
| *CXCL13* | SNP_A-2293412 | Chr4 | 78740549 | –0.57622 | 2.65665E–07 |
| *CXCL13* | CN_1087731 | Chr4 | 78684197 | –0.4738 | 2.87248E–07 |
| *CXCL13* | SNP_A-8680283 | Chr4 | 78746688 | –0.42361 | 3.84355E–07 |
| *CXCL13* | CN_1087745 | Chr4 | 78739710 | –0.42143 | 9.83755E–07 |
| *CXCL13* | SNP_A-8555523 | Chr4 | 78683722 | –0.54085 | 1.34E–06 |
| *CXCL14* | SNP_A-2186937 | Chr5 | 134938478 | –1.09759 | 3.66319E–11 |
| *CXCL14* | CN_1084655 | Chr5 | 134938502 | –0.59247 | 1.86308E–08 |
| *CYBB* | SNP_A-1816426 | ChrX | 37526470 | 1.133737 | 2.49477E–14 |
| *CYBB* | CN_946319 | ChrX | 37528050 | 1.36921 | 2.83826E–12 |
| *CYBB* | SNP_A-2055135 | ChrX | 37543996 | 0.778213 | 6.09097E–12 |
| *CYBB* | CN_946326 | ChrX | 37544779 | 0.61857 | 1.01076E–07 |
| *CYBB* | CN_946318 | ChrX | 37527724 | 0.44322 | 7.08281E–07 |
| *CYSLTR1* | SNP_A-8281019 | ChrX | 77418759 | 1.120533 | 4.62032E–17 |
| *CYSLTR1* | CN_965091 | ChrX | 77424698 | 1.369873 | 5.69465E–15 |
| *CYSLTR1* | SNP_A-8281020 | ChrX | 77428125 | 0.899247 | 2.20062E–13 |
| *CYSLTR1* | CN_965094 | ChrX | 77429297 | 1.28234 | 9.77554E–13 |
| *CYSLTR1* | SNP_A-2173651 | ChrX | 77422652 | 0.918587 | 2.37589E–12 |
| *CYSLTR1* | SNP_A-4281363 | ChrX | 77420158 | 0.716873 | 9.95912E–12 |
| *CYSLTR1* | CN_965089 | ChrX | 77421943 | 0.7345 | 1.20116E–10 |
| *CYSLTR1* | CN_965092 | ChrX | 77425773 | 0.87474 | 5.67095E–10 |
| *CYSLTR1* | SNP_A-8497374 | ChrX | 77421618 | 0.404247 | 5.65699E–08 |
| *CYSLTR1* | CN_965090 | ChrX | 77422187 | 0.68075 | 1.52728E–07 |
| *CYSLTR1* | CN_965095 | ChrX | 77430456 | 0.736437 | 2.63126E–07 |
| *CYSLTR1* | CN_965088 | ChrX | 77416100 | 0.787987 | 5.45701E–07 |
| *DAP3* | CN_441480 | Chr1 | 153956723 | 0.833373 | 2.32764E–07 |
| *DAP3* | CN_441481 | Chr1 | 153965405 | 0.369367 | 1.30291E–06 |
| *DAPK1* | SNP_A-1997155 | Chr9 | 89361860 | –0.92542 | 2.13765E–17 |
| *DAPK1* | SNP_A-1936841 | Chr9 | 89362099 | –0.49367 | 5.47198E–11 |
| *DAPK1* | SNP_A-8599782 | Chr9 | 89453843 | –0.53035 | 5.92253E–09 |
| *DAPK1* | SNP_A-2258140 | Chr9 | 89452483 | –0.5736 | 3.94672E–08 |
| *DAPK1* | SNP_A-4207741 | Chr9 | 89380485 | –0.49264 | 6.85952E–07 |
| *DAPP1* | AFFX-SNP_6513477 | Chr4 | 100966995 | –0.53332 | 1.5783E–11 |
| *DAPP1* | SNP_A-1978249 | Chr4 | 100970716 | 0.797137 | 1.8275E–10 |
| *DAPP1* | SNP_A-2089646 | Chr4 | 100966995 | –0.56746 | 1.98233E–09 |
| *DAPP1* | CN_1027406 | Chr4 | 100970558 | 0.99881 | 1.5128E–08 |
| *DAPP1* | CN_1027405 | Chr4 | 100967008 | –0.53217 | 9.7034E–08 |
| *DDX58* | SNP_A-8311832 | Chr9 | 32480675 | 1.06381 | 3.3314E–12 |
| *DDX58* | SNP_A-8456315 | Chr9 | 32451331 | 0.830717 | 4.49414E–12 |
| *DDX58* | SNP_A-8691258 | Chr9 | 32516146 | 0.638537 | 6.83239E–11 |
| *DDX58* | SNP_A-8345065 | Chr9 | 32480551 | 1.089623 | 4.09599E–09 |
| *DDX58* | CN_1317801 | Chr9 | 32508727 | 0.91331 | 5.75656E–08 |
| *DDX58* | SNP_A-8388061 | Chr9 | 32449450 | 0.612267 | 5.95977E–08 |
| *DDX58* | CN_1317802 | Chr9 | 32512044 | 0.736557 | 6.05738E–08 |
| *DDX58* | SNP_A-2293598 | Chr9 | 32515915 | 0.40924 | 1.17734E–07 |
| *DFFB* | SNP_A-2022696 | Chr1 | 3782661 | –0.55424 | 1.44127E–09 |
| *DFFB* | SNP_A-8381206 | Chr1 | 3784133 | –0.55576 | 9.46515E–07 |
| *DFFB* | CN_487345 | Chr1 | 3782826 | –0.36997 | 1.45E–06 |
| *DPP4* | CN_835016 | Chr2 | 162594367 | –0.48692 | 1.47999E–09 |
| *DPP4* | SNP_A-2297627 | Chr2 | 162594135 | –0.41361 | 1.75391E–08 |
| *DPP4* | CN_835010 | Chr2 | 162580798 | –0.61415 | 1.76004E–08 |
| *DPP4* | SNP_A-1946356 | Chr2 | 162598463 | –0.46475 | 5.62929E–07 |
| *DPP4* | SNP_A-2217544 | Chr2 | 162582416 | –0.51358 | 7.13573E–07 |
| *EDNRA* | SNP_A-2086040 | Chr4 | 148622999 | –1.0152 | 1.02838E–14 |
| *EDNRA* | CN_1061305 | Chr4 | 148660329 | –0.74051 | 2.24278E–12 |
| *EDNRA* | CN_1061304 | Chr4 | 148657294 | –0.501 | 5.32339E–10 |
| *EDNRA* | SNP_A-2110850 | Chr4 | 148622935 | –0.75541 | 3.0098E–09 |
| *EDNRA* | SNP_A-8663065 | Chr4 | 148659051 | –0.69348 | 4.4922E–08 |
| *EDNRB* | SNP_A-2271251 | Chr13 | 77368351 | –0.73612 | 1.47533E–10 |
| *EDNRB* | CN_652580 | Chr13 | 77369631 | –0.74744 | 2.77596E–09 |
| *EGF* | SNP_A-1922903 | Chr4 | 111144941 | –1.13179 | 6.87258E–14 |
| *EGF* | SNP_A-4275410 | Chr4 | 111144091 | –0.61481 | 2.29049E–12 |
| *EGF* | CN_1014301 | Chr4 | 111073844 | –0.61219 | 7.86966E–08 |
| *EGF* | CN_1014292 | Chr4 | 111058565 | –0.36243 | 5.71378E–07 |
| *EGF* | SNP_A-2098377 | Chr4 | 111056013 | –0.42548 | 8.80005E–07 |
| *EGF* | SNP_A-2010063 | Chr4 | 111073993 | –0.51043 | 9.50857E–07 |
| *EGFR* | CN_1227280 | Chr7 | 55181510 | –1.46982 | 2.28825E–18 |
| *EGFR* | SNP_A-2099331 | Chr7 | 55196509 | –0.95058 | 3.56592E–13 |
| *EGFR* | SNP_A-1895819 | Chr7 | 55259815 | –0.46913 | 1.41653E–12 |
| *EGFR* | SNP_A-8551620 | Chr7 | 55195667 | –0.978 | 6.50889E–11 |
| *EGFR* | CN_1225016 | Chr7 | 55073335 | –0.88671 | 1.55259E–10 |
| *EGFR* | CN_1225004 | Chr7 | 55065293 | –0.83032 | 1.46805E–09 |
| *EGFR* | CN_1227278 | Chr7 | 55181612 | –0.74337 | 1.5301E–09 |
| *EGFR* | SNP_A-8709043 | Chr7 | 55057873 | –0.4183 | 2.26907E–09 |
| *EGFR* | CN_1225005 | Chr7 | 55065159 | –0.61657 | 3.14689E–09 |
| *EGFR* | SNP_A-2252883 | Chr7 | 55072814 | –0.45717 | 5.68879E–09 |
| *EGFR* | SNP_A-1862898 | Chr7 | 55259220 | –0.47762 | 4.60733E–08 |
| *EGFR* | SNP_A-8455465 | Chr7 | 55181842 | –0.33708 | 8.63756E–08 |
| *EGFR* | CN_1225054 | Chr7 | 55098494 | –0.44416 | 3.99033E–07 |
| *EGFR* | SNP_A-8641374 | Chr7 | 55055909 | –0.42095 | 4.4027E–07 |
| *EGFR* | CN_1227281 | Chr7 | 55181696 | –0.48327 | 5.43712E–07 |
| *EGFR* | CN_1227279 | Chr7 | 55181632 | –0.81861 | 6.12099E–07 |
| *EGFR* | CN_1227282 | Chr7 | 55181522 | –0.51933 | 1.22447E–06 |
| *ELK1* | CN_966773 | ChrX | 47390772 | 0.999343 | 1.06274E–13 |
| *ELK1* | SNP_A-8536291 | ChrX | 47392685 | 0.848367 | 8.72944E–10 |
| *ELK1* | CN_966772 | ChrX | 47388989 | 0.56932 | 1.33961E–07 |
| *ELK1* | CN_966771 | ChrX | 47388531 | 0.594167 | 4.32711E–07 |
| *ENAH* | CN_488223 | Chr1 | 223821120 | 1.083103 | 5.78164E–14 |
| *ENAH* | CN_488227 | Chr1 | 223830839 | 1.040967 | 3.42276E–13 |
| *ENAH* | CN_486129 | Chr1 | 223782782 | 0.915503 | 2.65092E–12 |
| *ENAH* | SNP_A-8669552 | Chr1 | 223741626 | 0.550893 | 1.42534E–10 |
| *ENAH* | CN_486103 | Chr1 | 223743937 | 0.768353 | 8.96844E–10 |
| *ENAH* | SNP_A-2155437 | Chr1 | 223842585 | 0.58332 | 3.16772E–09 |
| *ENAH* | CN_016924 | Chr1 | 223830215 | 0.62973 | 4.07678E–09 |
| *ENAH* | CN_486108 | Chr1 | 223750598 | 0.530967 | 3.10946E–08 |
| *ENAH* | SNP_A-2229325 | Chr1 | 223821831 | 0.30977 | 6.55416E–08 |
| *ENAH* | SNP_A-1986639 | Chr1 | 223770108 | 0.510903 | 1.19787E–07 |
| *ENAH* | CN_488229 | Chr1 | 223842773 | 0.504493 | 1.97444E–07 |
| *ENAH* | CN_486095 | Chr1 | 223741354 | 0.47805 | 2.80545E–07 |
| *ENAH* | CN_486128 | Chr1 | 223768349 | 0.662157 | 3.47171E–07 |
| *ESR1* | CN_1176946 | Chr6 | 152272420 | –1.07435 | 6.26557E–16 |
| *ESR1* | CN_1176969 | Chr6 | 152297458 | –0.59317 | 7.85397E–12 |
| *ESR1* | CN_1174823 | Chr6 | 152203077 | –0.59805 | 1.07365E–10 |
| *ESR1* | CN_1176971 | Chr6 | 152297728 | –0.6842 | 1.36545E–10 |
| *ESR1* | CN_317926 | Chr6 | 152203259 | –0.53937 | 4.94798E–08 |
| *ESR1* | CN_1176970 | Chr6 | 152297692 | –0.65519 | 8.71159E–08 |
| *ESR1* | CN_1176949 | Chr6 | 152272367 | –0.48531 | 5.44199E–07 |
| *ETS1* | CN_541156 | Chr11 | 127869835 | –0.99537 | 4.0738E–14 |
| *ETS1* | SNP_A-2043806 | Chr11 | 127885735 | –0.7149 | 6.08338E–14 |
| *ETS1* | CN_541189 | Chr11 | 127885807 | –0.77336 | 2.49228E–11 |
| *ETS1* | CN_539113 | Chr11 | 127860104 | –0.6361 | 3.27247E–11 |
| *ETS1* | CN_541192 | Chr11 | 127886092 | –0.65731 | 1.82695E–10 |
| *ETS1* | CN_541159 | Chr11 | 127869783 | –0.68368 | 3.91183E–10 |
| *ETS1* | CN_541191 | Chr11 | 127885976 | –0.59844 | 5.47814E–10 |
| *ETS1* | CN_539114 | Chr11 | 127859974 | –0.69314 | 6.81443E–10 |
| *ETS1* | CN_541157 | Chr11 | 127869875 | –0.42071 | 2.52588E–09 |
| *ETS1* | CN_541187 | Chr11 | 127885623 | –0.79957 | 6.56293E–09 |
| *ETS1* | CN_541197 | Chr11 | 127886251 | –0.43161 | 1.00671E–08 |
| *ETS1* | CN_056151 | Chr11 | 127840087 | –0.43784 | 2.85678E–08 |
| *ETS1* | CN_541190 | Chr11 | 127885883 | –0.71855 | 4.87589E–08 |
| *ETS1* | CN_541158 | Chr11 | 127869921 | –0.4666 | 6.71789E–08 |
| *ETS1* | CN_539033 | Chr11 | 127840260 | –0.42477 | 1.01474E–07 |
| *ETS1* | CN_541185 | Chr11 | 127881332 | –0.43539 | 1.05276E–07 |
| *ETS1* | CN_539115 | Chr11 | 127860005 | –0.49723 | 1.21941E–07 |
| *ETS1* | CN_541230 | Chr11 | 127900543 | –0.47695 | 4.04202E–07 |
| *ETS1* | CN_541229 | Chr11 | 127898055 | –0.67395 | 4.09778E–07 |
| *ETS1* | CN_541198 | Chr11 | 127886269 | –0.44842 | 1.06929E–06 |
| *ETS1* | CN_539032 | Chr11 | 127840102 | –0.30516 | 1.4061E–06 |
| *ETS1* | CN_541155 | Chr11 | 127869798 | –0.33294 | 1.4133E–06 |
| *EZR* | SNP_A-1988326 | Chr6 | 159142532 | 0.790493 | 5.9958E–10 |
| *EZR* | SNP_A-1988325 | Chr6 | 159142435 | 0.55569 | 7.91679E–07 |
| *FAF1* | SNP_A-2063364 | Chr1 | 50849410 | 0.70091 | 5.26276E–10 |
| *FAF1* | CN_473828 | Chr1 | 50967736 | 0.827527 | 6.01506E–10 |
| *FAF1* | CN_473847 | Chr1 | 51044199 | 0.493863 | 7.43754E–08 |
| *FAF1* | SNP_A-2281703 | Chr1 | 51025206 | 0.440383 | 7.53218E–08 |
| *FAF1* | CN_473848 | Chr1 | 51044651 | 0.802543 | 8.01321E–08 |
| *FAF1* | SNP_A-8427962 | Chr1 | 50850677 | 0.519137 | 1.7159E–07 |
| *FAF1* | CN_473827 | Chr1 | 50962461 | 0.514833 | 2.07015E–07 |
| *FAS* | SNP_A-8373090 | Chr10 | 90743224 | 1.371263 | 2.38243E–13 |
| *FAS* | SNP_A-2218638 | Chr10 | 90743240 | 0.726587 | 1.41465E–08 |
| *FCGR2B* | CN_439125 | Chr1 | 159884179 | 1.245283 | 1.20441E–12 |
| *FCGR2B* | CN_439126 | Chr1 | 159884263 | 1.163177 | 1.22234E–11 |
| *FCGR2B* | CN_439137 | Chr1 | 159885636 | 1.20031 | 6.69181E–09 |
| *FCGR2B* | CN_439136 | Chr1 | 159885592 | 0.99517 | 2.93668E–08 |
| *FCGR2B* | CN_439115 | Chr1 | 159878870 | 0.80239 | 1.13553E–07 |
| *FCGR2B* | CN_439124 | Chr1 | 159884071 | 0.701593 | 1.39773E–07 |
| *FCGR2B* | CN_439114 | Chr1 | 159878821 | 0.67809 | 3.85535E–07 |
| *FCGR2B* | CN_439135 | Chr1 | 159885573 | 0.83164 | 1.46763E–06 |
| *FLT3* | CN_649389 | Chr13 | 27542422 | –0.7045 | 2.71502E–10 |
| *FLT3* | SNP_A-2240138 | Chr13 | 27509186 | –0.682 | 2.84761E–09 |
| *FLT3* | CN_649391 | Chr13 | 27542551 | –0.72713 | 2.56681E–08 |
| *FLT3* | CN_649388 | Chr13 | 27542407 | –0.6441 | 4.54657E–08 |
| *FLT3* | CN_649390 | Chr13 | 27542454 | –0.67004 | 9.44602E–08 |
| *FLT3* | SNP_A-8454420 | Chr13 | 27510246 | –0.52839 | 1.57791E–07 |
| *FLT3* | SNP_A-8635120 | Chr13 | 27510351 | –0.52563 | 5.17078E–07 |
| *FOXO1* | CN_094957 | Chr13 | 40068000 | –0.40104 | 7.12974E–08 |
| *FOXO1* | CN_643185 | Chr13 | 40096885 | –0.48308 | 8.05613E–08 |
| *FOXO1* | CN_643173 | Chr13 | 40064757 | –0.5037 | 3.2264E–07 |
| *FOXO1* | CN_643184 | Chr13 | 40092575 | –0.35181 | 5.38073E–07 |
| *FOXP3* | CN_975788 | ChrX | 48999231 | 1.248103 | 1.16241E–11 |
| *FOXP3* | CN_975787 | ChrX | 48999074 | 0.958523 | 1.60841E–08 |
| *FOXP3* | CN_975790 | ChrX | 48999720 | 0.74769 | 3.47104E–07 |
| *FOXP3* | CN_975793 | ChrX | 49005311 | 0.49122 | 1.40328E–06 |
| *FYN* | CN_1156304 | Chr6 | 112233085 | –0.56124 | 8.31131E–09 |
| *FYN* | SNP_A-1813267 | Chr6 | 112226674 | –0.64426 | 1.29509E–08 |
| *FYN* | SNP_A-8659107 | Chr6 | 112233032 | –0.62548 | 1.60432E–08 |
| *FYN* | SNP_A-2217758 | Chr6 | 112228919 | –0.55364 | 4.2535E–08 |
| *FYN* | SNP_A-8644912 | Chr6 | 112136297 | –0.45847 | 9.97487E–08 |
| *FYN* | CN_1156270 | Chr6 | 112133845 | –0.45477 | 2.15667E–07 |
| *GAB1* | CN_1072227 | Chr4 | 144511609 | 0.94598 | 2.93097E–09 |
| *GAB1* | CN_1072228 | Chr4 | 144511610 | 0.99053 | 3.9635E–09 |
| *GAB1* | CN_1072226 | Chr4 | 144511608 | 0.90069 | 4.11393E–09 |
| *GAS2* | CN_560939 | Chr11 | 22747913 | –0.61843 | 1.64319E–09 |
| *GAS2* | CN_560934 | Chr11 | 22736030 | –0.85946 | 8.40512E–09 |
| *GAS2* | SNP_A-8564187 | Chr11 | 22734230 | –0.69303 | 4.0343E–08 |
| *GLCCI1* | SNP_A-2149357 | Chr7 | 8013431 | 0.59295 | 1.83133E–09 |
| *GLCCI1* | SNP_A-4292255 | Chr7 | 8013694 | 0.636857 | 2.30675E–07 |
| *GRB2* | SNP_A-8496437 | Chr17 | 70826963 | 0.79969 | 2.71551E–09 |
| *GRB2* | CN_150911 | Chr17 | 70827185 | 0.65694 | 3.07829E–09 |
| *HLA-DOA* | SNP_A-2175319 | Chr6 | 33081721 | –0.6525 | 1.00259E–10 |
| *HLA-DOA* | SNP_A-1793236 | Chr6 | 33081856 | –0.46274 | 1.89973E–07 |
| *HLA-DQA1* | SNP_A-2214036 | Chr6 | 32710247 | 0.752997 | 2.15411E–12 |
| *HLA-DQA1* | SNP_A-1842324 | Chr6 | 32712350 | 0.50333 | 1.94151E–07 |
| *HLA-DQB2* | SNP_A-4265591 | Chr6 | 32837990 | 1.235207 | 9.41166E–13 |
| *HLA-DQB2* | SNP_A-1985638 | Chr6 | 32837799 | 0.87891 | 1.13139E–07 |
| *HRH1* | CN_979898 | Chr3 | 11195177 | –0.58349 | 1.17313E–10 |
| *HRH1* | CN_979905 | Chr3 | 11197054 | –0.69847 | 1.17236E–09 |
| *ICAM2* | CN_735255 | Chr17 | 59448849 | 1.05905 | 1.41356E–09 |
| *ICAM2* | CN_735254 | Chr17 | 59443946 | 0.49786 | 1.04437E–08 |
| *ICOSLG* | CN_895517 | Chr21 | 44481040 | 1.549543 | 1.19548E–09 |
| *ICOSLG* | SNP_A-2020423 | Chr21 | 44478042 | 0.536297 | 3.60412E–07 |
| *IFNAR2* | CN_880203 | Chr21 | 33540110 | –0.85297 | 7.97305E–11 |
| *IFNAR2* | SNP_A-1799142 | Chr21 | 33551045 | –0.81788 | 1.08086E–09 |
| *IFNAR2* | SNP_A-2018270 | Chr21 | 33540155 | –0.46029 | 7.98781E–07 |
| *IFNAR2* | CN_880207 | Chr21 | 33551123 | –0.42237 | 9.07907E–07 |
| *IGSF3* | CN_436164 | Chr1 | 116944744 | –0.92383 | 1.27436E–15 |
| *IGSF3* | CN_436163 | Chr1 | 116943111 | –0.76313 | 2.73528E–11 |
| *IL10RB* | SNP_A-1970077 | Chr21 | 33584581 | 0.497523 | 6.02975E–07 |
| *IL10RB* | SNP_A-2018287 | Chr21 | 33583043 | 0.406817 | 1.12706E–06 |
| *IL12RB1* | CN_782660 | Chr19 | 17800553 | 0.878573 | 5.35384E–09 |
| *IL12RB1* | CN_782664 | Chr19 | 17807304 | 0.66974 | 1.11926E–06 |
| *IL13RA1* | CN_920603 | ChrX | 117809160 | 0.92931 | 1.78189E–12 |
| *IL13RA1* | SNP_A-8547951 | ChrX | 117809926 | 0.91974 | 1.88489E–12 |
| *IL13RA1* | CN_918520 | ChrX | 117791875 | 1.15329 | 5.41658E–12 |
| *IL13RA1* | SNP_A-1926559 | ChrX | 117791585 | 0.900277 | 4.23338E–11 |
| *IL13RA1* | CN_918504 | ChrX | 117748617 | 0.94824 | 8.59565E–11 |
| *IL13RA1* | SNP_A-8426265 | ChrX | 117810809 | 1.070037 | 9.42264E–11 |
| *IL13RA1* | SNP_A-8683112 | ChrX | 117749921 | 0.983243 | 1.20021E–09 |
| *IL13RA1* | SNP_A-2115761 | ChrX | 117747776 | 0.70584 | 3.8272E–09 |
| *IL13RA1* | CN_920602 | ChrX | 117808386 | 0.68905 | 2.70084E–07 |
| *IL13RA1* | CN_918505 | ChrX | 117750349 | 0.566653 | 3.23174E–07 |
| *IL13RA1* | CN_918503 | ChrX | 117747166 | 0.6745 | 4.92681E–07 |
| *IL13RA1* | CN_918502 | ChrX | 117746286 | 0.72769 | 1.08014E–06 |
| *IL15* | SNP_A-2241527 | Chr4 | 142843742 | –0.78235 | 3.08155E–14 |
| *IL15* | SNP_A-8662883 | Chr4 | 142855763 | –0.66774 | 4.2906E–10 |
| *IL15* | CN_1063352 | Chr4 | 142848423 | –1.00269 | 1.01785E–09 |
| *IL15* | SNP_A-1979194 | Chr4 | 142856551 | –0.54811 | 3.90694E–09 |
| *IL15* | CN_1063358 | Chr4 | 142866334 | –0.57456 | 1.24279E–07 |
| *IL15* | CN_1063339 | Chr4 | 142799137 | –0.53795 | 4.86599E–07 |
| *IL15* | SNP_A-8371807 | Chr4 | 142799266 | –0.62296 | 9.20426E–07 |
| *IL15* | SNP_A-8309509 | Chr4 | 142856755 | –0.41741 | 9.31643E–07 |
| *IL16* | CN_727786 | Chr15 | 79359318 | –0.49294 | 4.13331E–08 |
| *IL16* | SNP_A-2208704 | Chr15 | 79359358 | –0.43995 | 9.02236E–07 |
| *IL17RA* | SNP_A-2192904 | Chr22 | 15971089 | –0.6331 | 1.02189E–08 |
| *IL17RA* | CN_897870 | Chr22 | 15970970 | –0.48394 | 7.29787E–07 |
| *IL17RB* | SNP_A-2035822 | Chr3 | 53864028 | 0.65259 | 8.60178E–10 |
| *IL17RB* | CN_1033076 | Chr3 | 53859083 | 0.674243 | 1.00454E–06 |
| *IL18R1* | SNP_A-1964291 | Chr2 | 102360765 | –0.60176 | 3.58653E–11 |
| *IL18R1* | SNP_A-2284768 | Chr2 | 102357801 | –0.52077 | 1.84557E–09 |
| *IL18R1* | CN_787961 | Chr2 | 102365316 | –0.59499 | 4.20663E–09 |
| *IL18R1* | CN_787960 | Chr2 | 102358384 | –0.73313 | 9.8048E–09 |
| *IL19* | SNP_A-2117477 | Chr1 | 205069942 | 0.933447 | 7.85462E–16 |
| *IL19* | SNP_A-1795090 | Chr1 | 205069997 | 0.834437 | 6.81018E–15 |
| *IL19* | SNP_A-1837995 | Chr1 | 205039862 | 0.766913 | 3.45501E–08 |
| *IL19* | SNP_A-2270537 | Chr1 | 205039659 | 0.441137 | 1.44127E–06 |
| *IL1A* | SNP_A-1929337 | Chr2 | 113249356 | 0.75008 | 1.69269E–13 |
| *IL1A* | CN_803555 | Chr2 | 113249404 | 0.567367 | 1.19594E–07 |
| *IL1R1* | SNP_A-8525742 | Chr2 | 102161035 | –0.59967 | 3.0224E–10 |
| *IL1R1* | CN_785841 | Chr2 | 102161250 | –0.28053 | 4.70992E–07 |
| *IL1RL1* | SNP_A-8450759 | Chr2 | 102304398 | –0.53928 | 5.86954E–14 |
| *IL1RL1* | SNP_A-2240346 | Chr2 | 102303413 | –0.38512 | 9.02441E–08 |
| *IL22RA1* | CN_457886 | Chr1 | 24328360 | –0.82381 | 1.34188E–10 |
| *IL22RA1* | CN_457883 | Chr1 | 24327352 | –0.55458 | 5.27555E–07 |
| *IL23R* | SNP_A-8485927 | Chr1 | 67426698 | 0.586307 | 2.79792E–09 |
| *IL23R* | SNP_A-8515023 | Chr1 | 67426677 | 0.55672 | 1.20915E–06 |
| *IL2RA* | CN_533323 | Chr10 | 6129122 | –0.88204 | 3.39432E–10 |
| *IL2RA* | SNP_A-2230517 | Chr10 | 6128705 | –0.49392 | 3.80317E–07 |
| *IL2RB* | SNP_A-8673480 | Chr22 | 35848578 | 1.029787 | 3.99952E–09 |
| *IL2RB* | SNP_A-8701067 | Chr22 | 35846316 | 0.780963 | 3.93829E–08 |
| *IL31RA* | CN_1134916 | Chr5 | 55198469 | –0.74449 | 2.60398E–11 |
| *IL31RA* | CN_1134915 | Chr5 | 55196610 | –0.75612 | 3.67007E–08 |
| *IL31RA* | SNP_A-8547238 | Chr5 | 55196859 | –0.43251 | 1.3216E–07 |
| *IL5RA* | SNP_A-8618211 | Chr3 | 3104632 | –0.50576 | 4.76094E–09 |
| *IL5RA* | CN_1014877 | Chr3 | 3104210 | –0.52686 | 2.6058E–07 |
| *INPP5D* | CN_847827 | Chr2 | 233747745 | 0.71714 | 2.0101E–08 |
| *INPP5D* | SNP_A-8540052 | Chr2 | 233746895 | 0.739163 | 1.32213E–07 |
| *INPP5D* | CN_847828 | Chr2 | 233748193 | 0.669703 | 1.83677E–07 |
| *IRAK2* | SNP_A-2214370 | Chr3 | 10233192 | 0.89833 | 8.58762E–11 |
| *IRAK2* | SNP_A-2080736 | Chr3 | 10234114 | 1.026757 | 2.71961E–09 |
| *IRAK2* | CN_957251 | Chr3 | 10232620 | 0.678583 | 2.17588E–07 |
| *IRAK4* | SNP_A-4221093 | Chr12 | 42450708 | –0.46221 | 1.71969E–09 |
| *IRAK4* | SNP_A-4232504 | Chr12 | 42450956 | –0.43789 | 2.6362E–07 |
| *IRF2* | SNP_A-1903862 | Chr4 | 185566046 | –0.8943 | 1.24406E–10 |
| *IRF2* | SNP_A-8476891 | Chr4 | 185565811 | –0.61745 | 5.06217E–10 |
| *IRF2* | SNP_A-1980221 | Chr4 | 185591421 | –0.38681 | 4.39359E–08 |
| *IRF2* | CN_1064236 | Chr4 | 185591557 | –0.55565 | 9.70935E–07 |
| *IRF6* | SNP_A-8363474 | Chr1 | 208027059 | 1.022487 | 3.23223E–13 |
| *IRF6* | SNP_A-2114785 | Chr1 | 208027646 | 0.8934 | 1.05353E–10 |
| *IRF8* | CN_745075 | Chr16 | 84508833 | –0.52976 | 7.32974E–10 |
| *IRF8* | SNP_A-2004975 | Chr16 | 84508855 | –0.59161 | 5.15274E–07 |
| *ITGA1* | CN_1119262 | Chr5 | 52234067 | –0.48328 | 1.55316E–10 |
| *ITGA1* | CN_1119263 | Chr5 | 52235265 | –0.45383 | 1.71591E–07 |
| *ITGA4* | CN_805064 | Chr2 | 182058204 | 0.652793 | 1.34383E–07 |
| *ITGA4* | SNP_A-8676720 | Chr2 | 182058390 | 0.589583 | 9.2113E–07 |
| *ITGA6* | SNP_A-8360957 | Chr2 | 173032084 | 0.8878 | 4.13638E–08 |
| *ITGA6* | SNP_A-1965850 | Chr2 | 173039791 | 0.765027 | 6.16319E–08 |
| *ITGA6* | CN_822217 | Chr2 | 173032136 | 0.424053 | 1.3619E–06 |
| *ITGAV* | CN_835618 | Chr2 | 187186529 | –0.49689 | 1.22133E–07 |
| *ITGAV* | CN_835619 | Chr2 | 187187529 | –0.38361 | 1.06234E–06 |
| *ITGB2* | SNP_A-8346062 | Chr21 | 45154168 | –0.61054 | 5.57605E–10 |
| *ITGB2* | CN_891571 | Chr21 | 45155014 | –0.4452 | 1.35083E–06 |
| *ITGB3* | SNP_A-4257129 | Chr17 | 42710352 | 0.741363 | 4.43328E–12 |
| *ITGB3* | SNP_A-1874351 | Chr17 | 42707934 | 0.74458 | 8.22743E–11 |
| *ITK* | SNP_A-4243674 | Chr5 | 156559935 | 1.249167 | 1.97998E–16 |
| *ITK* | SNP_A-1868301 | Chr5 | 156553911 | 0.598663 | 3.66826E–09 |
| *ITK* | CN_1098297 | Chr5 | 156553438 | 0.682463 | 2.32328E–07 |
| *ITK* | SNP_A-8306906 | Chr5 | 156559261 | 0.561227 | 2.9141E–07 |
| *ITK* | SNP_A-8295633 | Chr5 | 156559241 | 0.445387 | 1.12882E–06 |
| *JAK1* | CN_515467 | Chr1 | 65182873 | 1.337337 | 2.92187E–12 |
| *JAK1* | CN_515465 | Chr1 | 65182850 | 1.14613 | 6.16334E–11 |
| *JAK1* | CN_515404 | Chr1 | 65135018 | 0.678467 | 2.32898E–09 |
| *JAK1* | CN_515405 | Chr1 | 65135128 | 0.542823 | 4.63006E–09 |
| *JAK1* | CN_515469 | Chr1 | 65182783 | 0.706063 | 2.43376E–08 |
| *JAK1* | CN_515388 | Chr1 | 65129526 | 0.65733 | 3.32318E–08 |
| *JAK1* | SNP_A-4197685 | Chr1 | 65129664 | 0.413967 | 4.78691E–08 |
| *JAK1* | CN_515466 | Chr1 | 65182865 | 0.850007 | 1.70387E–07 |
| *JAK1* | CN_515389 | Chr1 | 65129549 | 0.714277 | 7.01715E–07 |
| *JAK2* | CN_1331586 | Chr9 | 5096698 | 1.122937 | 1.721E–10 |
| *JAK2* | CN_1331588 | Chr9 | 5096800 | 0.926307 | 1.50372E–09 |
| *JAK2* | CN_1331607 | Chr9 | 5105822 | 1.03309 | 2.10404E–09 |
| *JAK2* | CN_1329384 | Chr9 | 5027453 | 0.921187 | 4.14288E–09 |
| *JAK2* | CN_1329390 | Chr9 | 5027461 | 0.91835 | 5.82941E–09 |
| *JAK2* | CN_1331589 | Chr9 | 5096680 | 0.862753 | 6.1182E–09 |
| *JAK2* | CN_1331606 | Chr9 | 5105779 | 0.726923 | 7.35575E–09 |
| *JAK2* | CN_1329389 | Chr9 | 5027460 | 0.875383 | 1.01124E–08 |
| *JAK2* | CN_1329385 | Chr9 | 5027454 | 0.735113 | 3.35612E–08 |
| *JAK2* | CN_1329388 | Chr9 | 5027458 | 0.76774 | 4.58157E–08 |
| *JAK2* | CN_1329386 | Chr9 | 5027456 | 0.708693 | 1.03559E–07 |
| *JAK2* | CN_389854 | Chr9 | 5096796 | 0.73167 | 1.841E–07 |
| *JAK2* | CN_1329387 | Chr9 | 5027457 | 0.76501 | 3.19794E–07 |
| *JAK2* | CN_1329391 | Chr9 | 5027462 | 0.828813 | 4.28313E–07 |
| *JAK2* | CN_1331585 | Chr9 | 5096696 | 0.67959 | 1.07158E–06 |
| *KIR3DL2* | CN_768222 | Chr19 | 60060175 | –0.74643 | 2.03493E–14 |
| *KIR3DL2* | CN_768223 | Chr19 | 60065630 | –0.89113 | 4.40896E–08 |
| *KLRD1* | SNP_A-1945071 | Chr12 | 10349288 | –0.85459 | 4.43434E–10 |
| *KLRD1* | SNP_A-4301899 | Chr12 | 10349454 | –0.32255 | 1.10553E–06 |
| *KSR1* | CN_738940 | Chr17 | 22882653 | –0.78424 | 1.03124E–10 |
| *KSR1* | SNP_A-4297201 | Chr17 | 22883607 | –0.46942 | 9.36793E–07 |
| *LBP* | SNP_A-8533904 | Chr20 | 36427724 | 1.046117 | 6.3312E–12 |
| *LBP* | SNP_A-4259205 | Chr20 | 36426461 | 0.57654 | 2.64024E–07 |
| *LIFR* | SNP_A-1819232 | Chr5 | 38605614 | 0.444607 | 2.99487E–10 |
| *LIFR* | CN_1132373 | Chr5 | 38603745 | 0.615967 | 8.34845E–08 |
| *LILRB4* | SNP_A-4198657 | Chr19 | 59854767 | –0.66471 | 4.49658E–13 |
| *LILRB4* | SNP_A-4301584 | Chr19 | 59855149 | –0.74003 | 7.11375E–08 |
| *LIMS1* | SNP_A-4262139 | Chr2 | 108596821 | –1.2341 | 1.24464E–15 |
| *LIMS1* | CN_822971 | Chr2 | 108596934 | –0.83503 | 1.25963E–10 |
| *LIMS1* | CN_822970 | Chr2 | 108596263 | –0.40846 | 1.11449E–07 |
| *LRRC23* | SNP_A-8481444 | Chr12 | 6868632 | 0.97644 | 5.00375E–12 |
| *LRRC23* | SNP_A-8685056 | Chr12 | 6879469 | 0.987713 | 5.69655E–10 |
| *LYN* | SNP_A-8691666 | Chr8 | 57038845 | 1.08752 | 3.2103E–14 |
| *LYN* | SNP_A-1892133 | Chr8 | 57034656 | 0.856713 | 6.31277E–11 |
| *LYN* | SNP_A-4247835 | Chr8 | 57038773 | 0.511443 | 5.67433E–10 |
| *LYN* | CN_1266051 | Chr8 | 56984304 | 1.17878 | 6.54527E–10 |
| *LYN* | SNP_A-2084167 | Chr8 | 56987448 | 0.65872 | 6.93376E–09 |
| *LYN* | SNP_A-4218134 | Chr8 | 57034636 | 0.771077 | 8.95654E–09 |
| *MAP2K3* | SNP_A-8635525 | Chr17 | 21157832 | –0.79639 | 4.1114E–10 |
| *MAP2K3* | CN_764928 | Chr17 | 21157575 | –0.54083 | 1.16048E–06 |
| *MAP2K4* | SNP_A-8544946 | Chr17 | 11924957 | –0.64139 | 2.87285E–10 |
| *MAP2K4* | CN_749513 | Chr17 | 11872373 | –0.81756 | 3.0499E–10 |
| *MAP2K4* | CN_749528 | Chr17 | 11920990 | –0.62359 | 3.55649E–09 |
| *MAP2K4* | CN_749515 | Chr17 | 11880235 | –0.57122 | 3.97906E–08 |
| *MAP2K4* | CN_749529 | Chr17 | 11921909 | –0.47881 | 9.00939E–08 |
| *MAP2K4* | SNP_A-2146686 | Chr17 | 11925064 | –0.47801 | 3.31311E–07 |
| *MAP2K4* | SNP_A-8605871 | Chr17 | 11871706 | –0.56245 | 3.46479E–07 |
| *MAP2K6* | SNP_A-8400200 | Chr17 | 65034855 | 1.133493 | 3.96295E–13 |
| *MAP2K6* | SNP_A-2311053 | Chr17 | 64924561 | 0.784553 | 1.28929E–11 |
| *MAP2K6* | SNP_A-4283440 | Chr17 | 64924226 | 1.227347 | 1.36867E–11 |
| *MAP2K6* | SNP_A-2191548 | Chr17 | 64926642 | 0.894517 | 1.562E–11 |
| *MAP2K6* | SNP_A-8352055 | Chr17 | 64926630 | 0.840353 | 1.59668E–11 |
| *MAP2K6* | SNP_A-8292057 | Chr17 | 65033968 | 0.59013 | 4.14363E–10 |
| *MAP2K6* | SNP_A-2313584 | Chr17 | 64924711 | 0.549147 | 2.2168E–09 |
| *MAP3K3* | SNP_A-8304923 | Chr17 | 59123188 | –0.68353 | 7.1042E–12 |
| *MAP3K3* | CN_733099 | Chr17 | 59123213 | –0.61795 | 3.02902E–08 |
| *MAP3K5* | SNP_A-8671493 | Chr6 | 137011546 | –0.79264 | 6.68722E–12 |
| *MAP3K5* | CN_1159018 | Chr6 | 136937145 | –0.82168 | 1.16206E–09 |
| *MAP3K5* | SNP_A-1883415 | Chr6 | 136937159 | –0.54979 | 2.57555E–09 |
| *MAP3K5* | CN_1159049 | Chr6 | 137012769 | –0.60176 | 1.60362E–08 |
| *MAP3K5* | SNP_A-8393818 | Chr6 | 136981511 | –0.54420 | 4.52274E–08 |
| *MAP3K5* | CN_315320 | Chr6 | 136935606 | –0.5405 | 1.0135E–07 |
| *MAP3K5* | CN_1159036 | Chr6 | 136981787 | –0.60457 | 5.34173E–07 |
| *MAP3K8* | SNP_A-1805768 | Chr10 | 30771666 | 0.883003 | 1.64734E–10 |
| *MAP3K8* | CN_521697 | Chr10 | 30771830 | 0.5065 | 1.31618E–06 |
| *MAPKAPK2* | SNP_A-2212982 | Chr1 | 204937958 | 1.339203 | 3.10487E–17 |
| *MAPKAPK2* | SNP_A-8701207 | Chr1 | 204938407 | 1.170653 | 5.35953E–15 |
| *MARCO* | SNP_A-8575022 | Chr2 | 119456760 | –0.64421 | 5.83028E–13 |
| *MARCO* | SNP_A-1791973 | Chr2 | 119454287 | –0.63601 | 1.72315E–12 |
| *MARCO* | SNP_A-4241737 | Chr2 | 119454595 | –0.47514 | 8.42977E–08 |
| *MARCO* | SNP_A-4261192 | Chr2 | 119454657 | –0.53631 | 1.13315E–07 |
| *MASP1* | SNP_A-8380899 | Chr3 | 188452845 | –0.85996 | 9.17477E–17 |
| *MASP1* | SNP_A-2287818 | Chr3 | 188447962 | –0.93966 | 9.46459E–14 |
| *MASP1* | CN_994585 | Chr3 | 188447352 | –0.82132 | 2.40305E–11 |
| *MASP1* | SNP_A-2211042 | Chr3 | 188437018 | –0.53948 | 7.49317E–11 |
| *MASP1* | CN_994582 | Chr3 | 188436793 | –0.47861 | 5.6003E–08 |
| *MASP1* | SNP_A-1784736 | Chr3 | 188452328 | –0.49951 | 7.95149E–08 |
| *MDM2* | CN_624999 | Chr12 | 67522384 | 1.03213 | 7.88427E–11 |
| *MDM2* | CN_624996 | Chr12 | 67519797 | 0.76331 | 1.9708E–09 |
| *MDM2* | CN_625000 | Chr12 | 67522998 | 0.596347 | 4.69868E–08 |
| *MDM2* | CN_624995 | Chr12 | 67519783 | 0.753567 | 5.67924E–08 |
| *MED14* | SNP_A-2218704 | ChrX | 40430741 | 1.122657 | 1.459E–16 |
| *MED14* | SNP_A-8690576 | ChrX | 40429769 | 1.13729 | 1.94632E–15 |
| *MED14* | CN_962143 | ChrX | 40443852 | 0.995467 | 7.32966E–14 |
| *MED14* | CN_962139 | ChrX | 40429628 | 1.118813 | 3.80376E–13 |
| *MED14* | SNP_A-8494069 | ChrX | 40450393 | 0.962907 | 5.65611E–13 |
| *MED14* | SNP_A-8518826 | ChrX | 40425764 | 0.840813 | 5.04648E–12 |
| *MED14* | SNP_A-8464158 | ChrX | 40438785 | 1.301737 | 5.37542E–12 |
| *MED14* | CN_962138 | ChrX | 40428852 | 1.232537 | 1.0396E–11 |
| *MED14* | CN_962141 | ChrX | 40442463 | 0.90593 | 1.42719E–11 |
| *MED14* | CN_962142 | ChrX | 40442998 | 0.947517 | 6.10963E–11 |
| *MED14* | CN_962129 | ChrX | 40399089 | 0.945613 | 6.75507E–11 |
| *MED14* | CN_962137 | ChrX | 40426710 | 0.586363 | 8.24029E–11 |
| *MED14* | SNP_A-8616778 | ChrX | 40422682 | 0.53525 | 8.82102E–11 |
| *MED14* | CN_962145 | ChrX | 40450201 | 0.984267 | 1.05616E–09 |
| *MED14* | SNP_A-8592637 | ChrX | 40398419 | 0.491707 | 1.64768E–09 |
| *MED14* | CN_962136 | ChrX | 40424159 | 0.613497 | 1.63695E–07 |
| *MED14* | SNP_A-8301982 | ChrX | 40449974 | 0.516857 | 2.01988E–07 |
| *MED14* | CN_962144 | ChrX | 40450119 | 0.594743 | 2.38545E–07 |
| *MEF2A* | CN_721613 | Chr15 | 98068782 | 0.67713 | 1.95021E–09 |
| *MEF2A* | SNP_A-2004447 | Chr15 | 98068538 | 0.563587 | 2.22231E–08 |
| *MGST2* | SNP_A-8408111 | Chr4 | 140861894 | –0.72762 | 1.66808E–10 |
| *MGST2* | SNP_A-2046740 | Chr4 | 140851397 | –0.42102 | 9.50493E–08 |
| *MGST2* | SNP_A-8645829 | Chr4 | 140862772 | –0.49289 | 5.69592E–07 |
| *MGST3* | SNP_A-4285997 | Chr1 | 163892293 | 0.817637 | 2.61758E–12 |
| *MGST3* | SNP_A-8553172 | Chr1 | 163887226 | 1.130613 | 1.25185E–11 |
| *MGST3* | SNP_A-8379788 | Chr1 | 163886336 | 0.861153 | 1.80569E–11 |
| *MGST3* | SNP_A-8579420 | Chr1 | 163893837 | 0.606373 | 3.50595E–10 |
| *MGST3* | SNP_A-2199041 | Chr1 | 163892098 | 0.43691 | 4.918E–08 |
| *MMP2* | SNP_A-8478601 | Chr16 | 54081499 | –0.87964 | 1.38542E–12 |
| *MMP2* | SNP_A-4258044 | Chr16 | 54090771 | –0.61692 | 1.96281E–10 |
| *MMP2* | SNP_A-2183862 | Chr16 | 54081206 | –0.75238 | 4.55568E–09 |
| *MMP2* | CN_704962 | Chr16 | 54089725 | –0.49386 | 6.47554E–09 |
| *MS4A1* | SNP_A-2307552 | Chr11 | 59988901 | 0.963553 | 4.09746E–09 |
| *MS4A1* | SNP_A-2074432 | Chr11 | 59988562 | 0.580747 | 4.53531E–07 |
| *MSR1* | SNP_A-1876148 | Chr8 | 16082787 | –0.77671 | 8.14744E–12 |
| *MSR1* | SNP_A-4266818 | Chr8 | 16028961 | –0.74307 | 1.74883E–10 |
| *MSR1* | SNP_A-4259210 | Chr8 | 16083874 | –0.66329 | 3.69195E–10 |
| *MSR1* | SNP_A-8345258 | Chr8 | 16028994 | –0.47289 | 2.36681E–07 |
| *MYH10* | CN_733503 | Chr17 | 8334673 | –1.35313 | 3.40195E–17 |
| *MYH10* | CN_733540 | Chr17 | 8425452 | –0.6623 | 6.48872E–13 |
| *MYH10* | CN_733513 | Chr17 | 8353539 | –0.74407 | 1.71162E–11 |
| *MYH10* | CN_733547 | Chr17 | 8443267 | –0.95782 | 2.99561E–10 |
| *MYH10* | CN_733546 | Chr17 | 8442286 | –0.8049 | 4.28919E–10 |
| *MYH10* | SNP_A-8661302 | Chr17 | 8334625 | –1.14374 | 2.09208E–09 |
| *MYH10* | SNP_A-2052894 | Chr17 | 8425408 | –0.60196 | 3.00959E–09 |
| *MYH10* | CN_733514 | Chr17 | 8353916 | –0.59845 | 4.07971E–09 |
| *MYH4* | CN_740753 | Chr17 | 10297909 | –0.51619 | 5.15065E–07 |
| *MYH4* | SNP_A-2031468 | Chr17 | 10297541 | –0.40543 | 1.05529E–06 |
| *MYH9* | SNP_A-2103130 | Chr22 | 35062483 | –0.85587 | 3.12135E–12 |
| *MYH9* | SNP_A-8370383 | Chr22 | 35038570 | –0.59158 | 1.15979E–09 |
| *MYH9* | SNP_A-2069555 | Chr22 | 35034780 | –0.64207 | 1.95605E–09 |
| *MYH9* | CN_915610 | Chr22 | 35034752 | –0.68782 | 1.02289E–08 |
| *MYH9* | CN_915623 | Chr22 | 35062532 | –0.58104 | 1.27032E–07 |
| *MYH9* | SNP_A-8617356 | Chr22 | 35060893 | –0.49353 | 2.40301E–07 |
| *MYH9* | SNP_A-8610644 | Chr22 | 35037607 | –0.62782 | 2.87572E–07 |
| *NCAM1* | SNP_A-1932535 | Chr11 | 112570529 | –0.91896 | 4.76908E–16 |
| *NCAM1* | SNP_A-8649761 | Chr11 | 112641290 | –0.65878 | 1.56768E–11 |
| *NCAM1* | SNP_A-8688892 | Chr11 | 112570503 | –0.70803 | 6.1655E–11 |
| *NCAM1* | SNP_A-4248853 | Chr11 | 112504948 | –0.63201 | 8.08254E–11 |
| *NCAM1* | SNP_A-2019563 | Chr11 | 112491251 | –0.66944 | 1.90281E–10 |
| *NCAM1* | CN_553678 | Chr11 | 112634805 | –0.63526 | 9.23706E–10 |
| *NCAM1* | CN_553657 | Chr11 | 112606874 | –0.70063 | 1.92651E–09 |
| *NCAM1* | CN_551559 | Chr11 | 112413871 | –0.65461 | 2.26504E–09 |
| *NCAM1* | SNP_A-8338525 | Chr11 | 112491679 | –0.55883 | 7.28733E–09 |
| *NCAM1* | SNP_A-2019572 | Chr11 | 112491480 | –0.55729 | 8.65141E–09 |
| *NCAM1* | SNP_A-2019562 | Chr11 | 112491180 | –0.57125 | 1.2539E–08 |
| *NCAM1* | SNP_A-8652005 | Chr11 | 112505042 | –0.51958 | 1.54954E–08 |
| *NCAM1* | CN_551605 | Chr11 | 112532784 | –0.42443 | 5.16262E–08 |
| *NCAM1* | SNP_A-2262397 | Chr11 | 112606353 | –0.50857 | 6.03941E–08 |
| *NCAM1* | CN_551558 | Chr11 | 112411646 | –0.72866 | 6.36749E–08 |
| *NCAM1* | SNP_A-2194957 | Chr11 | 112630444 | –0.64099 | 1.58619E–07 |
| *NCAM1* | SNP_A-2050253 | Chr11 | 112629928 | –0.47327 | 2.22177E–07 |
| *NCAM1* | CN_553677 | Chr11 | 112630921 | –0.5291 | 2.23464E–07 |
| *NCAM1* | CN_553658 | Chr11 | 112609109 | –0.60288 | 8.6603E–07 |
| *NCAM1* | SNP_A-1900766 | Chr11 | 112532851 | –0.39555 | 9.39491E–07 |
| *NCAM1* | SNP_A-4259317 | Chr11 | 112640216 | –0.4926 | 1.09771E–06 |
| *NCOA2* | CN_373326 | Chr8 | 71268446 | 0.696277 | 2.47116E–10 |
| *NCOA2* | CN_1275446 | Chr8 | 71277679 | 0.928353 | 1.46567E–07 |
| *NFATC1* | CN_162724 | Chr18 | 75288253 | –0.8305 | 1.90285E–16 |
| *NFATC1* | SNP_A-8712992 | Chr18 | 75288153 | –1.02835 | 1.68513E–15 |
| *NFATC2* | CN_901198 | Chr20 | 49527334 | 1.09991 | 1.39698E–10 |
| *NFATC2* | SNP_A-8374999 | Chr20 | 49527151 | 0.914597 | 7.26794E–10 |
| *NFATC2* | CN_901187 | Chr20 | 49500135 | 0.381683 | 5.29167E–08 |
| *NFATC2* | CN_901188 | Chr20 | 49500347 | 0.981467 | 1.36355E–07 |
| *NFKB1* | CN_1042975 | Chr4 | 103703766 | –0.85815 | 3.7777E–11 |
| *NFKB1* | SNP_A-2193021 | Chr4 | 103700596 | –0.48159 | 1.70748E–09 |
| *NFKB1* | SNP_A-1978305 | Chr4 | 103725482 | –0.65908 | 1.11253E–08 |
| *NFKB1* | CN_1042992 | Chr4 | 103740165 | –0.53114 | 1.8759E–08 |
| *NFKB1* | CN_1042986 | Chr4 | 103725912 | –0.57329 | 4.61762E–07 |
| *NFKB1* | CN_1042991 | Chr4 | 103739444 | –0.50811 | 5.34943E–07 |
| *NLRP1* | CN_739513 | Chr17 | 5402432 | –1.01238 | 4.81928E–15 |
| *NLRP1* | SNP_A-1940088 | Chr17 | 5404556 | –0.42506 | 1.02202E–07 |
| *NLRP1* | CN_739523 | Chr17 | 5404414 | –0.68014 | 1.54939E–07 |
| *NLRP1* | SNP_A-1886965 | Chr17 | 5402238 | –0.43218 | 8.74725E–07 |
| *NLRP2* | SNP_A-1899719 | Chr19 | 60179962 | 1.51803 | 2.12592E–15 |
| *NLRP2* | CN_770331 | Chr19 | 60194502 | 1.25017 | 3.27051E–15 |
| *NLRP2* | SNP_A-2266907 | Chr19 | 60197020 | 1.024177 | 1.35344E–12 |
| *NLRP2* | SNP_A-8351390 | Chr19 | 60177979 | 1.213813 | 3.47233E–10 |
| *NLRP3* | SNP_A-2098659 | Chr1 | 245651266 | 1.364583 | 1.6866E–18 |
| *NLRP3* | CN_468963 | Chr1 | 245651587 | 0.619123 | 1.26324E–06 |
| *NOD2* | CN_709114 | Chr16 | 49317115 | –0.96976 | 6.65308E–09 |
| *NOD2* | SNP_A-1875178 | Chr16 | 49309288 | –0.54078 | 3.26929E–08 |
| *NOD2* | SNP_A-2266236 | Chr16 | 49317048 | –0.82191 | 4.30889E–08 |
| *NOD2* | SNP_A-2204401 | Chr16 | 49308899 | –0.44844 | 9.37319E–07 |
| *NOS2* | SNP_A-8408148 | Chr17 | 23140975 | 1.07746 | 1.99458E–16 |
| *NOS2* | AFFX-SNP_1102206 | Chr17 | 23148826 | 0.600567 | 3.43515E–10 |
| *NOS2* | SNP_A-1846050 | Chr17 | 23148826 | 0.65616 | 4.33805E–09 |
| *NOS2* | CN_741068 | Chr17 | 23140400 | 0.507337 | 7.22114E–09 |
| *NT5E* | CN_1213512 | Chr6 | 86250193 | –0.73664 | 2.23576E–09 |
| *NT5E* | SNP_A-1821739 | Chr6 | 86252017 | –0.34698 | 1.05714E–06 |
| *OAS2* | SNP_A-4287535 | Chr12 | 111925304 | –0.58338 | 6.18692E–10 |
| *OAS2* | CN_590900 | Chr12 | 111920855 | –0.64206 | 3.51815E–09 |
| *OAS2* | CN_590902 | Chr12 | 111922641 | –0.61396 | 5.2603E–09 |
| *OAS2* | SNP_A-8466176 | Chr12 | 111921206 | –0.62325 | 1.95158E–08 |
| *OAS2* | CN_590903 | Chr12 | 111926101 | –0.55017 | 1.19341E–06 |
| *OSMR* | CN_1132484 | Chr5 | 38928214 | –0.92531 | 1.54605E–16 |
| *OSMR* | SNP_A-8554138 | Chr5 | 38935698 | –0.64914 | 1.41514E–10 |
| *OSMR* | SNP_A-2126065 | Chr5 | 38967454 | –0.59838 | 4.00001E–10 |
| *OSMR* | SNP_A-2209868 | Chr5 | 38938129 | –0.83993 | 6.58826E–10 |
| *OSMR* | SNP_A-1914489 | Chr5 | 38965275 | 0.559273 | 3.82216E–07 |
| *OSMR* | CN_1132485 | Chr5 | 38939248 | 0.60875 | 3.99664E–07 |
| *PARP1* | SNP_A-2105844 | Chr1 | 224640025 | 0.983193 | 1.66589E–10 |
| *PARP1* | SNP_A-2238639 | Chr1 | 224643529 | 0.59001 | 5.0051E–08 |
| *PARP1* | SNP_A-2108341 | Chr1 | 224643929 | 0.50191 | 1.03876E–06 |
| *PAX5* | SNP_A-1996626 | Chr9 | 36866740 | –0.88873 | 8.52706E–14 |
| *PAX5* | SNP_A-2158412 | Chr9 | 36885654 | –0.67613 | 4.56304E–12 |
| *PAX5* | CN_1304446 | Chr9 | 36869722 | –0.62687 | 1.1187E–09 |
| *PAX5* | SNP_A-1915358 | Chr9 | 36918504 | –0.53348 | 4.06871E–09 |
| *PAX5* | SNP_A-1881034 | Chr9 | 36868774 | –0.60473 | 4.41625E–08 |
| *PAX5* | CN_1304474 | Chr9 | 36885661 | –0.51336 | 5.43003E–08 |
| *PAX5* | SNP_A-2206249 | Chr9 | 36912924 | –0.52027 | 1.1082E–07 |
| *PAX5* | SNP_A-8642207 | Chr9 | 36912489 | –0.52352 | 1.2788E–07 |
| *PAX5* | CN_1304497 | Chr9 | 36918487 | –0.43255 | 1.8774E–07 |
| *PAX5* | SNP_A-2264967 | Chr9 | 36965108 | –0.50386 | 3.69497E–07 |
| *PAX5* | SNP_A-8631895 | Chr9 | 36964991 | –0.31744 | 1.16489E–06 |
| *PDCD1LG2* | SNP_A-8454427 | Chr9 | 5531877 | 1.51986 | 4.88349E–16 |
| *PDCD1LG2* | SNP_A-8410584 | Chr9 | 5534405 | 0.83705 | 2.80659E–10 |
| *PDE1A* | SNP_A-8326709 | Chr2 | 183061583 | –0.49431 | 2.31987E–12 |
| *PDE1A* | SNP_A-1919399 | Chr2 | 182747666 | –0.60359 | 1.29322E–11 |
| *PDE1A* | SNP_A-8422139 | Chr2 | 182747179 | –0.51364 | 1.32289E–10 |
| *PDE1A* | SNP_A-4260877 | Chr2 | 183061827 | –0.43692 | 2.54887E–08 |
| *PDE1A* | CN_809363 | Chr2 | 182747515 | –0.36826 | 1.15475E–07 |
| *PDE1B* | SNP_A-8630284 | Chr12 | 53258566 | –0.6946 | 1.29888E–09 |
| *PDE1B* | SNP_A-8470868 | Chr12 | 53257614 | –0.47349 | 1.44386E–08 |
| *PDE1B* | SNP_A-8609235 | Chr12 | 53259110 | –0.50549 | 2.78595E–08 |
| *PDE1B* | CN_613867 | Chr12 | 53230545 | –0.39605 | 5.09686E–08 |
| *PDE1B* | SNP_A-2229150 | Chr12 | 53231332 | –0.36062 | 7.32727E–08 |
| *PDE1C* | SNP_A-8401367 | Chr7 | 32177958 | –0.84132 | 3.12467E–12 |
| *PDE1C* | SNP_A-2200622 | Chr7 | 32260779 | –0.72811 | 5.13458E–12 |
| *PDE1C* | SNP_A-8694710 | Chr7 | 32260169 | –0.56766 | 5.4717E–12 |
| *PDE1C* | SNP_A-2170643 | Chr7 | 32261546 | –0.51604 | 1.84793E–10 |
| *PDE1C* | SNP_A-2175751 | Chr7 | 32260519 | –0.532 | 2.6953E–10 |
| *PDE1C* | SNP_A-8405934 | Chr7 | 31779201 | –0.37792 | 3.80111E–10 |
| *PDE1C* | SNP_A-4235196 | Chr7 | 32036165 | –0.7474 | 1.7796E–09 |
| *PDE1C* | AFFX-SNP_8856868 | Chr7 | 32036165 | –0.64967 | 5.17133E–08 |
| *PDE1C* | SNP_A-2062780 | Chr7 | 32261700 | –0.36818 | 7.94379E–08 |
| *PDE1C* | SNP_A-2022567 | Chr7 | 31778788 | –0.50661 | 1.22917E–07 |
| *PDE2A* | SNP_A-8700666 | Chr11 | 72013482 | –1.0901 | 1.43698E–17 |
| *PDE2A* | CN_568433 | Chr11 | 71993407 | –0.63316 | 6.7341E–12 |
| *PDE2A* | CN_568441 | Chr11 | 72013558 | –0.92913 | 5.68906E–11 |
| *PDE2A* | SNP_A-8320029 | Chr11 | 71994132 | –0.58233 | 2.89306E–10 |
| *PDE2A* | CN_568459 | Chr11 | 72062719 | –0.73252 | 1.44223E–07 |
| *PDE3A* | SNP_A-2090095 | Chr12 | 20514796 | –0.63086 | 3.39547E–11 |
| *PDE3A* | SNP_A-1961051 | Chr12 | 20516066 | –0.63751 | 5.10476E–11 |
| *PDE3A* | CN_610995 | Chr12 | 20543086 | –0.61007 | 1.51121E–10 |
| *PDE3A* | CN_610987 | Chr12 | 20515473 | –0.75776 | 6.48217E–09 |
| *PDE3A* | SNP_A-8289823 | Chr12 | 20543378 | –0.53812 | 1.33588E–08 |
| *PDE3A* | CN_611006 | Chr12 | 20566071 | –0.53288 | 1.06422E–07 |
| *PDE3A* | CN_611007 | Chr12 | 20573288 | –0.53363 | 1.27966E–07 |
| *PDE3A* | SNP_A-1781724 | Chr12 | 20515544 | –0.43396 | 1.6702E–07 |
| *PDE3A* | CN_611005 | Chr12 | 20565228 | –0.36563 | 6.8166E–07 |
| *PDE3B* | CN_554177 | Chr11 | 14658651 | –0.48169 | 2.02023E–08 |
| *PDE3B* | CN_554176 | Chr11 | 14656025 | –0.6262 | 1.25872E–07 |
| *PDE4B* | SNP_A-8462113 | Chr1 | 66575668 | –0.82328 | 1.08574E–13 |
| *PDE4B* | SNP_A-8482298 | Chr1 | 66466583 | –0.63054 | 1.82396E–13 |
| *PDE4B* | SNP_A-2116334 | Chr1 | 66577858 | –0.67455 | 6.27466E–13 |
| *PDE4B* | SNP_A-2223776 | Chr1 | 66175012 | –0.72072 | 1.83906E–12 |
| *PDE4B* | SNP_A-2118706 | Chr1 | 66341686 | 0.803987 | 1.08616E–11 |
| *PDE4B* | SNP_A-1949676 | Chr1 | 66576136 | –0.49912 | 9.73846E–10 |
| *PDE4B* | SNP_A-4247253 | Chr1 | 66484864 | 0.665597 | 1.88318E–09 |
| *PDE4B* | SNP_A-8483943 | Chr1 | 66485033 | 0.647017 | 1.28999E–08 |
| *PDE4B* | CN_524158 | Chr1 | 66466509 | –0.63498 | 1.564E–08 |
| *PDE4B* | CN_522047 | Chr1 | 66269263 | 0.518123 | 1.99276E–08 |
| *PDE4B* | CN_522015 | Chr1 | 66175080 | –0.48146 | 9.29475E–08 |
| *PDE4B* | SNP_A-8480034 | Chr1 | 66348027 | 0.49788 | 2.07227E–07 |
| *PDE4B* | SNP_A-8455290 | Chr1 | 66272886 | 0.44339 | 5.0313E–07 |
| *PDE4D* | SNP_A-2231342 | Chr5 | 58625543 | –1.11487 | 4.29486E–15 |
| *PDE4D* | SNP_A-8367843 | Chr5 | 58698010 | 1.179987 | 5.44479E–14 |
| *PDE4D* | SNP_A-2240785 | Chr5 | 58514449 | –0.94233 | 8.19917E–13 |
| *PDE4D* | SNP_A-1861748 | Chr5 | 58514356 | –0.44673 | 5.10243E–12 |
| *PDE4D* | SNP_A-8411467 | Chr5 | 58692009 | 1.240977 | 5.51641E–12 |
| *PDE4D* | SNP_A-4210971 | Chr5 | 58697354 | 0.876677 | 1.36446E–11 |
| *PDE4D* | SNP_A-1895936 | Chr5 | 58681869 | –0.70544 | 1.77084E–11 |
| *PDE4D* | SNP_A-8455785 | Chr5 | 58625482 | –0.86299 | 9.7422E–11 |
| *PDE4D* | SNP_A-8705194 | Chr5 | 58645225 | 0.664963 | 2.0241E–10 |
| *PDE4D* | CN_1123893 | Chr5 | 59110568 | 1.152647 | 2.32263E–10 |
| *PDE4D* | SNP_A-8386153 | Chr5 | 58692085 | 0.96854 | 7.11612E–10 |
| *PDE4D* | CN_1119483 | Chr5 | 58514529 | –0.46633 | 1.55086E–09 |
| *PDE4D* | SNP_A-8475800 | Chr5 | 58721946 | –0.65665 | 8.85608E–09 |
| *PDE4D* | SNP_A-2024453 | Chr5 | 58682110 | –0.80521 | 1.52112E–08 |
| *PDE4D* | SNP_A-8676648 | Chr5 | 58443228 | –0.56888 | 1.68995E–08 |
| *PDE4D* | SNP_A-2076317 | Chr5 | 58681562 | –0.76164 | 2.05716E–08 |
| *PDE4D* | SNP_A-8635215 | Chr5 | 59109232 | 0.69571 | 5.3125E–08 |
| *PDE4D* | SNP_A-8630905 | Chr5 | 58645368 | 0.72206 | 8.55228E–08 |
| *PDE4D* | CN_1119533 | Chr5 | 58646783 | 0.659283 | 2.00936E–07 |
| *PDE4D* | SNP_A-4210969 | Chr5 | 58443782 | –0.36896 | 4.06343E–07 |
| *PDE4D* | CN_1121655 | Chr5 | 58721846 | –0.35987 | 1.22958E–06 |
| *PDGFRA* | CN_1065175 | Chr4 | 54847639 | –0.90278 | 1.05566E–10 |
| *PDGFRA* | CN_275828 | Chr4 | 54848421 | –0.98291 | 1.42746E–10 |
| *PDGFRA* | CN_1065122 | Chr4 | 54815314 | –0.72031 | 6.68927E–10 |
| *PDGFRA* | CN_1065146 | Chr4 | 54833917 | –0.62836 | 1.77337E–09 |
| *PDGFRA* | CN_1065153 | Chr4 | 54835536 | –0.61643 | 7.66102E–09 |
| *PDGFRA* | CN_1065151 | Chr4 | 54833839 | –0.52726 | 1.31339E–08 |
| *PDGFRA* | CN_1065155 | Chr4 | 54835616 | –0.66506 | 3.48358E–08 |
| *PDGFRA* | CN_1065174 | Chr4 | 54847613 | –0.57777 | 3.10085E–07 |
| *PDGFRA* | CN_1065181 | Chr4 | 54848508 | –0.51921 | 1.03609E–06 |
| *PDGFRA* | CN_1065124 | Chr4 | 54815339 | –0.46992 | 1.24331E–06 |
| *PDGFRB* | SNP_A-8608406 | Chr5 | 149489463 | –0.65875 | 6.00349E–08 |
| *PDGFRB* | CN_1093763 | Chr5 | 149489420 | –0.47552 | 1.49655E–07 |
| *PGLYRP4* | SNP_A-2267684 | Chr1 | 151581446 | 1.051277 | 6.02489E–14 |
| *PGLYRP4* | SNP_A-2308829 | Chr1 | 151575932 | 0.838613 | 1.63837E–10 |
| *PGLYRP4* | CN_435238 | Chr1 | 151575865 | 0.77083 | 4.09278E–10 |
| *PGLYRP4* | CN_435237 | Chr1 | 151574488 | 0.828973 | 1.24693E–09 |
| *PGLYRP4* | CN_435239 | Chr1 | 151581329 | 0.46443 | 8.54839E–08 |
| *PGLYRP4* | SNP_A-2205194 | Chr1 | 151581556 | 0.43032 | 1.09053E–07 |
| *PIAS1* | CN_696967 | Chr15 | 66186502 | 0.587327 | 2.77259E–08 |
| *PIAS1* | CN_696966 | Chr15 | 66184080 | 0.65468 | 4.33917E–08 |
| *PIAS2* | CN_779540 | Chr18 | 42687440 | –0.91265 | 3.95578E–11 |
| *PIAS2* | SNP_A-8594120 | Chr18 | 42687453 | –0.77388 | 2.97032E–09 |
| *PIK3AP1* | CN_536270 | Chr10 | 98444910 | –0.54147 | 2.3328E–12 |
| *PIK3AP1* | CN_536266 | Chr10 | 98437785 | –0.78906 | 5.9672E–11 |
| *PIK3AP1* | CN_536268 | Chr10 | 98440824 | –0.59897 | 3.6189E–10 |
| *PIK3AP1* | SNP_A-8482860 | Chr10 | 98370534 | –0.7783 | 9.77496E–09 |
| *PIK3AP1* | SNP_A-4221725 | Chr10 | 98370369 | –0.47431 | 5.46012E–08 |
| *PIK3AP1* | SNP_A-4244270 | Chr10 | 98438837 | –0.49443 | 3.73686E–07 |
| *PIK3AP1* | SNP_A-2289081 | Chr10 | 98444750 | –0.57698 | 7.15139E–07 |
| *PIK3CA* | CN_983546 | Chr3 | 180421778 | –0.82786 | 8.61311E–16 |
| *PIK3CA* | CN_981375 | Chr3 | 180354728 | –0.56254 | 1.79895E–09 |
| *PIK3CA* | CN_981437 | Chr3 | 180369316 | –0.61585 | 5.09762E–09 |
| *PIK3CA* | CN_983547 | Chr3 | 180421799 | –0.50242 | 1.13774E–08 |
| *PIK3CA* | CN_981436 | Chr3 | 180368999 | –0.56193 | 4.39381E–08 |
| *PIK3CA* | CN_981439 | Chr3 | 180369434 | –0.4203 | 5.48468E–07 |
| *PIK3CA* | CN_981438 | Chr3 | 180369327 | –0.41399 | 6.32753E–07 |
| *PIK3CA* | CN_981374 | Chr3 | 180354676 | –0.42004 | 6.63298E–07 |
| *PIK3CB* | CN_993483 | Chr3 | 139937753 | 0.894113 | 1.61225E–11 |
| *PIK3CB* | SNP_A-2034391 | Chr3 | 139937671 | 0.74679 | 3.47914E–11 |
| *PIK3R3* | SNP_A-1787544 | Chr1 | 46407736 | –0.50733 | 1.20138E–07 |
| *PIK3R3* | SNP_A-2021243 | Chr1 | 46406461 | –0.58647 | 5.30331E–07 |
| *PIK3R5* | SNP_A-2199520 | Chr17 | 8791185 | –0.83175 | 5.14733E–14 |
| *PIK3R5* | CN_735751 | Chr17 | 8792772 | –0.58106 | 5.83141E–10 |
| *PLA2G2A* | CN_470210 | Chr1 | 20176000 | –0.91 | 4.46565E–09 |
| *PLA2G2A* | CN_470213 | Chr1 | 20178308 | –0.67089 | 3.24776E–08 |
| *PLA2G2A* | SNP_A-8708201 | Chr1 | 20176194 | –0.3826 | 1.37E–06 |
| *PLCB1* | SNP_A-8638118 | Chr20 | 8109126 | 0.664003 | 3.01704E–11 |
| *PLCB1* | CN_877575 | Chr20 | 8109517 | 0.66177 | 2.75651E–07 |
| *PLCB4* | SNP_A-8589648 | Chr20 | 9256828 | –0.36481 | 7.97294E–10 |
| *PLCB4* | CN_884147 | Chr20 | 9180595 | –0.84818 | 1.5669E–09 |
| *PLCB4* | CN_884146 | Chr20 | 9180180 | –0.49078 | 1.98084E–08 |
| *PLCB4* | CN_884190 | Chr20 | 9255329 | –0.41909 | 2.12803E–07 |
| *PLCB4* | CN_884189 | Chr20 | 9251120 | –0.34307 | 1.43704E–06 |
| *PLCG1* | SNP_A-2254726 | Chr20 | 39203389 | –0.56432 | 4.78151E–10 |
| *PLCG1* | CN_874859 | Chr20 | 39206530 | –0.5811 | 6.1751E–10 |
| *PPARG* | SNP_A-1947193 | Chr3 | 12442613 | 1.092397 | 3.88537E–10 |
| *PPARG* | SNP_A-8636610 | Chr3 | 12426337 | –0.52643 | 1.12163E–09 |
| *PPARG* | CN_1008460 | Chr3 | 12427764 | –0.5399 | 1.75929E–08 |
| *PPARG* | CN_1010533 | Chr3 | 12442782 | 0.515483 | 1.96943E–08 |
| *PPARGC1A* | CN_1051383 | Chr4 | 23475013 | –0.60756 | 2.86652E–11 |
| *PPARGC1A* | SNP_A-1835294 | Chr4 | 23475114 | –0.54935 | 3.56175E–09 |
| *PPM1A* | CN_679980 | Chr14 | 59808023 | –0.82557 | 1.68526E–11 |
| *PPM1A* | CN_679981 | Chr14 | 59808452 | –0.55535 | 3.63005E–08 |
| *PPP1R7* | SNP_A-8349424 | Chr2 | 241767237 | 0.923333 | 3.61274E–13 |
| *PPP1R7* | SNP_A-8623189 | Chr2 | 241756908 | –0.79025 | 4.9056E–11 |
| *PPP1R7* | CN_854738 | Chr2 | 241740147 | 0.89105 | 1.73893E–09 |
| *PPP1R7* | SNP_A-8396914 | Chr2 | 241767359 | 0.554143 | 4.08641E–09 |
| *PPP1R7* | CN_854743 | Chr2 | 241756759 | –0.65046 | 2.67251E–08 |
| *PPP1R7* | SNP_A-8489744 | Chr2 | 241740255 | 0.654983 | 2.98488E–08 |
| *PPP1R7* | SNP_A-8541335 | Chr2 | 241768092 | 0.586913 | 5.09788E–07 |
| *PPP2CA* | SNP_A-2234670 | Chr5 | 133564957 | 0.647793 | 3.06466E–10 |
| *PPP2CA* | SNP_A-2046974 | Chr5 | 133565378 | 0.487917 | 2.11497E–08 |
| *PPP2R3A* | SNP_A-8618657 | Chr3 | 137196445 | 0.98102 | 1.35528E–12 |
| *PPP2R3A* | SNP_A-8445746 | Chr3 | 137196312 | 0.425907 | 7.34356E–08 |
| *PPP3CA* | CN_1034148 | Chr4 | 102369148 | –0.69084 | 4.01082E–11 |
| *PPP3CA* | SNP_A-2108277 | Chr4 | 102172345 | –0.71883 | 4.37448E–11 |
| *PPP3CA* | CN_1034131 | Chr4 | 102330514 | –0.69186 | 1.15865E–10 |
| *PPP3CA* | SNP_A-2122032 | Chr4 | 102354963 | –0.78908 | 3.05497E–10 |
| *PPP3CA* | SNP_A-1803406 | Chr4 | 102247778 | –0.76172 | 3.31865E–09 |
| *PPP3CA* | CN_1034076 | Chr4 | 102174966 | –0.57549 | 6.05038E–09 |
| *PPP3CA* | SNP_A-4291727 | Chr4 | 102332104 | –0.37174 | 1.85954E–08 |
| *PPP3CA* | CN_1034103 | Chr4 | 102247481 | –0.58686 | 8.41451E–08 |
| *PPP3CA* | SNP_A-1945405 | Chr4 | 102355196 | –0.55349 | 6.05188E–07 |
| *PPP3CA* | CN_1034150 | Chr4 | 102372705 | –0.72251 | 1.38019E–06 |
| *PPP3CB* | CN_542128 | Chr10 | 74867899 | –0.83305 | 1.0779E–11 |
| *PPP3CB* | SNP_A-1800394 | Chr10 | 74867057 | –0.43245 | 1.33472E–08 |
| *PPP3CC* | CN_1260862 | Chr8 | 22364081 | –0.62084 | 9.52266E–09 |
| *PPP3CC* | CN_1260900 | Chr8 | 22452777 | –0.77822 | 1.32495E–08 |
| *PPP3CC* | SNP_A-2139722 | Chr8 | 22364095 | –0.52728 | 3.45212E–08 |
| *PPP3CC* | SNP_A-8285812 | Chr8 | 22453493 | –0.49887 | 3.35046E–07 |
| *PRDX4* | SNP_A-2312294 | ChrX | 23601896 | 1.177107 | 2.00472E–13 |
| *PRDX4* | SNP_A-8600176 | ChrX | 23599308 | 0.713677 | 5.08023E–13 |
| *PRDX4* | SNP_A-8321246 | ChrX | 23599280 | 0.820253 | 5.41994E–13 |
| *PRDX4* | CN_936945 | ChrX | 23599608 | 0.92762 | 3.95408E–11 |
| *PRDX4* | SNP_A-8443876 | ChrX | 23602086 | 0.63104 | 8.93774E–09 |
| *PRDX4* | CN_936946 | ChrX | 23601916 | 0.622117 | 3.31222E–07 |
| *PRKACB* | SNP_A-4202513 | Chr1 | 84431717 | 0.793283 | 2.10287E–15 |
| *PRKACB* | CN_513741 | Chr1 | 84431734 | 0.996597 | 1.97276E–12 |
| *PRKACB* | SNP_A-2094409 | Chr1 | 84435529 | 0.53132 | 1.0617E–07 |
| *PRKCA* | SNP_A-1867375 | Chr17 | 61828086 | 1.119793 | 1.11141E–15 |
| *PRKCA* | SNP_A-8427406 | Chr17 | 62096890 | 1.02166 | 4.8815E–14 |
| *PRKCA* | SNP_A-2118344 | Chr17 | 61999089 | 1.118863 | 8.9609E–14 |
| *PRKCA* | SNP_A-8458316 | Chr17 | 62132085 | 0.904897 | 3.09708E–13 |
| *PRKCA* | SNP_A-2240932 | Chr17 | 61849811 | 0.641687 | 7.489E–13 |
| *PRKCA* | SNP_A-2072295 | Chr17 | 62218356 | 1.038493 | 7.7654E–13 |
| *PRKCA* | CN_750592 | Chr17 | 62168727 | 0.86915 | 2.12076E–12 |
| *PRKCA* | SNP_A-1883468 | Chr17 | 62132371 | 0.72191 | 1.73789E–11 |
| *PRKCA* | SNP_A-8502197 | Chr17 | 62137923 | 0.781423 | 1.20606E–10 |
| *PRKCA* | SNP_A-1952795 | Chr17 | 62219289 | 0.72445 | 2.21195E–10 |
| *PRKCA* | SNP_A-8403980 | Chr17 | 62038189 | 0.537403 | 4.83605E–10 |
| *PRKCA* | SNP_A-2253886 | Chr17 | 62168179 | 0.62655 | 3.70186E–09 |
| *PRKCA* | SNP_A-1844459 | Chr17 | 61848043 | 0.683167 | 6.85809E–09 |
| *PRKCA* | SNP_A-8471866 | Chr17 | 62135662 | 0.77701 | 7.00522E–09 |
| *PRKCA* | CN_748470 | Chr17 | 62000333 | 0.73095 | 1.46062E–08 |
| *PRKCA* | SNP_A-8403981 | Chr17 | 62037856 | 0.625603 | 1.66301E–08 |
| *PRKCA* | SNP_A-4251226 | Chr17 | 61828388 | 0.743453 | 3.58168E–08 |
| *PRKCA* | CN_750575 | Chr17 | 62132388 | 0.42938 | 2.028E–07 |
| *PRKCA* | SNP_A-1825738 | Chr17 | 62093747 | 0.312 | 6.53665E–07 |
| *PRKCB* | CN_697926 | Chr16 | 24051821 | –1.10717 | 2.91727E–12 |
| *PRKCB* | SNP_A-4242825 | Chr16 | 24052529 | –0.50768 | 6.49017E–11 |
| *PRKCB* | CN_697888 | Chr16 | 23959833 | –0.52352 | 3.55768E–10 |
| *PRKCB* | CN_730697 | Chr16 | 23849758 | –0.72633 | 9.89231E–09 |
| *PRKCB* | CN_730695 | Chr16 | 23847285 | –0.64218 | 1.45262E–07 |
| *PRKCB* | SNP_A-8676193 | Chr16 | 23957189 | –0.43997 | 3.05088E–07 |
| *PRKCE* | SNP_A-8705554 | Chr2 | 45947928 | –1.21639 | 1.82285E–18 |
| *PRKCE* | SNP_A-8581574 | Chr2 | 45848685 | –1.102 | 2.93524E–17 |
| *PRKCE* | SNP_A-1875937 | Chr2 | 45852620 | –0.86754 | 1.32752E–14 |
| *PRKCE* | SNP_A-8464919 | Chr2 | 45821800 | –0.43873 | 1.64408E–13 |
| *PRKCE* | SNP_A-8301976 | Chr2 | 45852579 | –0.86687 | 2.21257E–13 |
| *PRKCE* | SNP_A-8312164 | Chr2 | 45855664 | –0.36885 | 2.03344E–12 |
| *PRKCE* | SNP_A-1844125 | Chr2 | 46086885 | –0.7255 | 7.63263E–12 |
| *PRKCE* | CN_870884 | Chr2 | 45855929 | –0.4565 | 5.90714E–11 |
| *PRKCE* | SNP_A-2154461 | Chr2 | 45947977 | –0.96369 | 2.72008E–10 |
| *PRKCE* | SNP_A-2251120 | Chr2 | 46085876 | –0.72287 | 2.94246E–09 |
| *PRKCE* | SNP_A-4300528 | Chr2 | 45892651 | –0.60219 | 3.93594E–08 |
| *PRKCE* | CN_870873 | Chr2 | 45821795 | –0.29072 | 1.4625E–07 |
| *PRKCE* | CN_870880 | Chr2 | 45848953 | –0.75687 | 2.32017E–07 |
| *PRKCE* | SNP_A-8703923 | Chr2 | 45892305 | –0.40883 | 1.41452E–06 |
| *PRKCQ* | SNP_A-2039362 | Chr10 | 6541451 | 0.857127 | 1.6656E–10 |
| *PRKCQ* | SNP_A-4274400 | Chr10 | 6585110 | –0.5038 | 3.89471E–08 |
| *PRKCQ* | CN_524831 | Chr10 | 6600079 | –0.42491 | 6.54852E–08 |
| *PRKCQ* | SNP_A-4266974 | Chr10 | 6542103 | 0.681573 | 3.22105E–07 |
| *PRKCQ* | CN_526906 | Chr10 | 6610345 | –0.43488 | 4.25892E–07 |
| *PRKCQ* | SNP_A-2300959 | Chr10 | 6586077 | –0.52333 | 7.98509E–07 |
| *PRKCZ* | SNP_A-1788728 | Chr1 | 2095738 | –0.85886 | 4.9204E–16 |
| *PRKCZ* | SNP_A-1842509 | Chr1 | 2096298 | –0.68793 | 3.7694E–09 |
| *PSMA1* | CN_552146 | Chr11 | 14620169 | –0.5878 | 1.17298E–09 |
| *PSMA1* | CN_552139 | Chr11 | 14589089 | –0.45888 | 1.2519E–09 |
| *PSMA1* | CN_057379 | Chr11 | 14619022 | –0.40073 | 1.5123E–08 |
| *PSMA1* | SNP_A-2138300 | Chr11 | 14589146 | –0.76096 | 6.74342E–08 |
| *PSMB9* | SNP_A-1790157 | Chr6 | 32924936 | 1.181017 | 2.46953E–13 |
| *PSMB9* | SNP_A-4244883 | Chr6 | 32940378 | 0.768533 | 1.45427E–12 |
| *PSMB9* | SNP_A-1956059 | Chr6 | 32925108 | 1.003347 | 1.42458E–11 |
| *PSMB9* | SNP_A-8670485 | Chr6 | 32943236 | 0.64538 | 2.55593E–10 |
| *PTEN* | CN_555389 | Chr10 | 89713853 | –0.51559 | 5.35617E–09 |
| *PTEN* | CN_555390 | Chr10 | 89713866 | –0.70049 | 8.02196E–08 |
| *PTGFR* | SNP_A-2097216 | Chr1 | 78567620 | –0.54349 | 1.35912E–09 |
| *PTGFR* | CN_517991 | Chr1 | 78732446 | –0.59685 | 3.85967E–09 |
| *PTGFR* | SNP_A-2226034 | Chr1 | 78572002 | –0.48349 | 6.47831E–07 |
| *PTK2* | SNP_A-1816283 | Chr8 | 142069369 | 1.043823 | 3.29841E–10 |
| *PTK2* | CN_1267318 | Chr8 | 142068881 | 1.053753 | 1.18582E–09 |
| *PTK2* | CN_1265126 | Chr8 | 141813748 | 0.5991 | 1.94715E–09 |
| *PTK2* | CN_1265127 | Chr8 | 141814046 | 0.918093 | 2.44393E–09 |
| *PTK2B* | SNP_A-2137951 | Chr8 | 27363942 | –0.86292 | 7.98877E–12 |
| *PTK2B* | CN_1290158 | Chr8 | 27343896 | –0.74196 | 1.90226E–11 |
| *PTK2B* | SNP_A-2154531 | Chr8 | 27299633 | –0.48397 | 1.22307E–10 |
| *PTK2B* | CN_1290141 | Chr8 | 27299814 | –0.9403 | 8.50494E–10 |
| *PTK2B* | CN_1290140 | Chr8 | 27299736 | –0.62285 | 6.15857E–09 |
| *PTK2B* | SNP_A-2186008 | Chr8 | 27310997 | –0.48144 | 4.33281E–08 |
| *PTK2B* | SNP_A-2310192 | Chr8 | 27310553 | –0.36875 | 1.47351E–07 |
| *PTK2B* | SNP_A-8644929 | Chr8 | 27343950 | –0.36719 | 9.28587E–07 |
| *PTK2B* | SNP_A-2269048 | Chr8 | 27364455 | –0.4331 | 9.62084E–07 |
| *PTPN13* | SNP_A-1829064 | Chr4 | 87908475 | –0.94184 | 1.76384E–11 |
| *PTPN13* | CN_1098934 | Chr4 | 87844661 | –0.86594 | 4.16291E–10 |
| *PTPN13* | SNP_A-8538625 | Chr4 | 87908292 | –0.65553 | 1.58177E–09 |
| *PTPN13* | SNP_A-1952665 | Chr4 | 87908557 | –0.40735 | 2.02284E–07 |
| *PTPN13* | CN_1098935 | Chr4 | 87845098 | –0.48353 | 3.83724E–07 |
| *PTPN13* | SNP_A-1958156 | Chr4 | 87909434 | –0.4463 | 4.63235E–07 |
| *PTPN13* | CN_1098961 | Chr4 | 87908002 | –0.58417 | 6.96288E–07 |
| *PTPN13* | CN_1098933 | Chr4 | 87844501 | –0.59086 | 1.29396E–06 |
| *PTPRC* | SNP_A-8431022 | Chr1 | 196954570 | 0.436023 | 4.9083E–07 |
| *PTPRC* | SNP_A-1955333 | Chr1 | 196958987 | 0.648447 | 1.00771E–06 |
| *PTPRK* | CN_1178660 | Chr6 | 128478108 | –0.72474 | 1.81356E–14 |
| *PTPRK* | SNP_A-2083326 | Chr6 | 128362605 | –0.73021 | 9.24673E–12 |
| *PTPRK* | SNP_A-2159950 | Chr6 | 128607214 | –1.04769 | 1.51671E–10 |
| *PTPRK* | SNP_A-8704726 | Chr6 | 128607423 | –0.87225 | 7.59859E–10 |
| *PTPRK* | SNP_A-1921111 | Chr6 | 128610463 | –0.75396 | 2.83438E–09 |
| *PTPRK* | CN_1180848 | Chr6 | 128849769 | –0.55286 | 4.88721E–09 |
| *PTPRK* | SNP_A-4218314 | Chr6 | 128606026 | –0.62996 | 9.16021E–09 |
| *PTPRK* | CN_1178727 | Chr6 | 128663843 | –0.54421 | 9.25423E–09 |
| *PTPRK* | CN_1178610 | Chr6 | 128350190 | –0.74138 | 1.54403E–08 |
| *PTPRK* | CN_1180847 | Chr6 | 128843092 | –0.61993 | 2.21688E–08 |
| *PTPRK* | CN_1178661 | Chr6 | 128481507 | –0.44585 | 1.0416E–07 |
| *PTPRK* | CN_1178728 | Chr6 | 128665661 | –0.45738 | 1.25879E–07 |
| *PTPRK* | CN_1178611 | Chr6 | 128350196 | –0.60506 | 4.07414E–07 |
| *PTPRK* | SNP_A-8366646 | Chr6 | 128872727 | –0.4486 | 5.29343E–07 |
| *PTPRK* | SNP_A-4283512 | Chr6 | 128871996 | –0.52621 | 6.32135E–07 |
| *PTPRK* | CN_1178619 | Chr6 | 128362747 | –0.8579 | 7.44452E–07 |
| *PTPRU* | SNP_A-8676127 | Chr1 | 29504987 | 0.772577 | 4.05751E–09 |
| *PTPRU* | SNP_A-8324657 | Chr1 | 29505187 | 0.68641 | 5.31592E–08 |
| *RAC2* | SNP_A-8551586 | Chr22 | 35960808 | –0.66962 | 1.47821E–08 |
| *RAC2* | CN_920037 | Chr22 | 35967125 | –0.44986 | 1.10931E–07 |
| *RAC2* | SNP_A-1796283 | Chr22 | 35960734 | –0.4462 | 9.25053E–07 |
| *RAF1* | SNP_A-8617204 | Chr3 | 12624576 | 0.891433 | 1.17822E–10 |
| *RAF1* | CN_984602 | Chr3 | 12625712 | 0.706667 | 4.57825E–07 |
| *RAP1A* | CN_439802 | Chr1 | 112042712 | –0.66256 | 1.19559E–10 |
| *RAP1A* | SNP_A-4286704 | Chr1 | 112038561 | –0.40493 | 5.9233E–08 |
| *RAP1A* | CN_439801 | Chr1 | 112038004 | –0.34329 | 2.42644E–07 |
| *RASSF5* | SNP_A-2165732 | Chr1 | 204803880 | 0.99354 | 1.17384E–15 |
| *RASSF5* | SNP_A-4250653 | Chr1 | 204803899 | 0.548533 | 4.0817E–09 |
| *RASSF5* | CN_485619 | Chr1 | 204806224 | 0.559923 | 2.27391E–08 |
| *RFX4* | SNP_A-8710687 | Chr12 | 105577736 | –1.10091 | 3.43453E–12 |
| *RFX4* | CN_586431 | Chr12 | 105579844 | –0.83525 | 1.50906E–08 |
| *RHEB* | SNP_A-8338162 | Chr7 | 150805219 | 0.456043 | 8.56154E–10 |
| *RHEB* | SNP_A-8284974 | Chr7 | 150804982 | 0.518567 | 2.18615E–08 |
| *RIPK1* | SNP_A-8648027 | Chr6 | 3015437 | 0.611273 | 7.72297E–09 |
| *RIPK1* | SNP_A-1920574 | Chr6 | 3016608 | 0.661627 | 1.0046E–08 |
| *RPS6KA5* | SNP_A-4278399 | Chr14 | 90506789 | –0.80323 | 4.89868E–13 |
| *RPS6KA5* | SNP_A-8453267 | Chr14 | 90509132 | –0.76699 | 4.55582E–11 |
| *RPS6KA5* | SNP_A-2169653 | Chr14 | 90572871 | –0.90559 | 1.69384E–09 |
| *RPS6KA5* | SNP_A-2245903 | Chr14 | 90573122 | –0.7315 | 1.21037E–07 |
| *RPS6KA5* | CN_115606 | Chr14 | 90509067 | –0.44891 | 1.64618E–07 |
| *RPS6KA5* | CN_695792 | Chr14 | 90572832 | –0.41704 | 3.51421E–07 |
| *RPS6KA5* | CN_695791 | Chr14 | 90572315 | –0.48189 | 1.26177E–06 |
| *SELL* | SNP_A-8561103 | Chr1 | 167932764 | 1.320173 | 7.12306E–18 |
| *SELL* | SNP_A-4301173 | Chr1 | 167937712 | 0.418543 | 8.36959E–09 |
| *SELL* | CN_447094 | Chr1 | 167933993 | 0.824557 | 4.52747E–08 |
| *SELL* | SNP_A-4285385 | Chr1 | 167937741 | 0.32797 | 4.56389E–08 |
| *SELL* | SNP_A-8616909 | Chr1 | 167933979 | 0.686757 | 9.07026E–08 |
| *SH2D1A* | CN_916438 | ChrX | 123316921 | 0.95647 | 6.99103E–12 |
| *SH2D1A* | SNP_A-8404355 | ChrX | 123315919 | 0.883937 | 1.231E–11 |
| *SH2D1A* | CN_916437 | ChrX | 123314466 | 1.186213 | 2.31593E–11 |
| *SHB* | SNP_A-8282600 | Chr9 | 38030095 | –0.82221 | 6.3437E–15 |
| *SHB* | SNP_A-8649196 | Chr9 | 38030114 | –0.81655 | 5.32387E–13 |
| *SIRPB1* | CN_889680 | Chr20 | 1494811 | –0.62387 | 7.36577E–12 |
| *SIRPB1* | CN_207160 | Chr20 | 1508562 | 0.799257 | 1.30184E–10 |
| *SIRPB1* | CN_889679 | Chr20 | 1494762 | –0.84171 | 8.08515E–10 |
| *SIRPB1* | CN_891765 | Chr20 | 1508543 | 0.86535 | 1.38309E–09 |
| *SIRPG* | SNP_A-4234772 | Chr20 | 1558551 | –0.69273 | 5.77434E–14 |
| *SIRPG* | SNP_A-4289786 | Chr20 | 1558939 | –0.39056 | 9.76081E–10 |
| *SIRPG* | SNP_A-4295438 | Chr20 | 1558201 | –0.39396 | 1.49089E–08 |
| *SLAMF1* | SNP_A-8440933 | Chr1 | 158853211 | 1.312333 | 3.02934E–16 |
| *SLAMF1* | SNP_A-2110783 | Chr1 | 158854866 | 1.094657 | 2.92444E–14 |
| *SLAMF1* | CN_436639 | Chr1 | 158861274 | 0.60833 | 1.83E–09 |
| *SLAMF1* | SNP_A-2256542 | Chr1 | 158860862 | 0.480277 | 4.97138E–08 |
| *SLAMF1* | SNP_A-8520494 | Chr1 | 158852851 | 0.52499 | 4.53185E–07 |
| *SLAMF6* | SNP_A-4229977 | Chr1 | 158731535 | 0.956537 | 7.72868E–13 |
| *SLAMF6* | SNP_A-8691050 | Chr1 | 158732980 | 0.96367 | 9.75464E–12 |
| *SLAMF6* | SNP_A-2039761 | Chr1 | 158731640 | 0.632143 | 4.40939E–09 |
| *SLAMF6* | SNP_A-1968710 | Chr1 | 158723193 | 0.54582 | 4.51208E–08 |
| *SLAMF6* | SNP_A-8579236 | Chr1 | 158725234 | 0.524203 | 6.07981E–08 |
| *SOCS4* | SNP_A-1821152 | Chr14 | 54576510 | –0.58842 | 1.64381E–08 |
| *SOCS4* | CN_690654 | Chr14 | 54572523 | –0.51689 | 8.01568E–07 |
| *SOCS6* | CN_780050 | Chr18 | 66110471 | –0.6946 | 1.13883E–07 |
| *SOCS6* | SNP_A-2110677 | Chr18 | 66111294 | –0.35555 | 4.36177E–07 |
| *SOS2* | SNP_A-8305843 | Chr14 | 49759112 | 0.884277 | 2.75212E–13 |
| *SOS2* | CN_666773 | Chr14 | 49761339 | 0.676363 | 1.09793E–08 |
| *SOS2* | SNP_A-8292673 | Chr14 | 49738581 | 0.616103 | 6.3841E–08 |
| *SOS2* | CN_666771 | Chr14 | 49757820 | 0.545433 | 2.10229E–07 |
| *SOS2* | SNP_A-8470857 | Chr14 | 49738311 | 0.57403 | 6.19012E–07 |
| *SOS2* | SNP_A-2207167 | Chr14 | 49754083 | 0.48536 | 7.96267E–07 |
| *STAT1* | SNP_A-1966288 | Chr2 | 191577408 | –0.87894 | 3.01377E–20 |
| *STAT1* | SNP_A-1966287 | Chr2 | 191577187 | –0.79251 | 2.74413E–13 |
| *STAT1* | SNP_A-4237286 | Chr2 | 191569622 | –0.71135 | 2.3465E–07 |
| *STAT1* | CN_826915 | Chr2 | 191569802 | –0.38235 | 4.66973E–07 |
| *STAT3* | SNP_A-4242727 | Chr17 | 37783361 | 1.30656 | 4.21383E–13 |
| *STAT3* | CN_752233 | Chr17 | 37784260 | 0.826677 | 5.67388E–08 |
| *STAT3* | SNP_A-8406717 | Chr17 | 37764060 | 0.63792 | 9.21092E–08 |
| *STAT3* | SNP_A-2230022 | Chr17 | 37767727 | 0.571597 | 6.26701E–07 |
| *STAT4* | SNP_A-2006914 | Chr2 | 191662109 | –0.76903 | 4.59069E–15 |
| *STAT4* | SNP_A-8425513 | Chr2 | 191662292 | –0.5257 | 6.91861E–09 |
| *STAT5B* | CN_752182 | Chr17 | 37654134 | 0.877683 | 3.93727E–10 |
| *STAT5B* | CN_752180 | Chr17 | 37653475 | 0.578527 | 1.14405E–06 |
| *SYK* | SNP_A-1839764 | Chr9 | 92618320 | –0.9893 | 1.10361E–16 |
| *SYK* | CN_1333646 | Chr9 | 92619561 | –0.34735 | 7.21373E–08 |
| *SYK* | CN_1333662 | Chr9 | 92649184 | –0.62245 | 4.54404E–07 |
| *SYK* | CN_1333663 | Chr9 | 92649398 | –0.3788 | 5.55885E–07 |
| *SYNGAP1* | CN_323411 | Chr6 | 33515746 | 0.895427 | 4.56354E–08 |
| *SYNGAP1* | SNP_A-2222969 | Chr6 | 33516520 | 0.466653 | 2.40388E–07 |
| *TANK* | SNP_A-4208458 | Chr2 | 161729164 | 0.82641 | 1.02525E–11 |
| *TANK* | CN_828536 | Chr2 | 161760227 | 0.533147 | 1.76887E–08 |
| *TANK* | CN_181697 | Chr2 | 161760243 | 0.597807 | 2.69325E–08 |
| *TANK* | CN_828534 | Chr2 | 161760168 | 0.645753 | 3.27768E–07 |
| *TANK* | CN_828527 | Chr2 | 161729413 | 0.600063 | 1.21031E–06 |
| *TBXAS1* | SNP_A-2293098 | Chr7 | 139204991 | 0.613837 | 1.17272E–09 |
| *TBXAS1* | SNP_A-2206359 | Chr7 | 139205023 | 0.7598 | 1.00476E–07 |
| *TDP2* | SNP_A-4273713 | Chr6 | 24761525 | 1.057647 | 1.07084E–12 |
| *TDP2* | SNP_A-1797782 | Chr6 | 24761252 | 0.82653 | 7.23411E–12 |
| *TEC* | CN_1078293 | Chr4 | 47881699 | –0.72432 | 2.6456E–11 |
| *TEC* | SNP_A-4300646 | Chr4 | 47868898 | –0.59492 | 3.45979E–11 |
| *TEC* | CN_1078202 | Chr4 | 47834455 | –0.64739 | 1.94069E–10 |
| *TEC* | CN_1078292 | Chr4 | 47881682 | –0.66993 | 8.59249E–10 |
| *TEC* | CN_1078294 | Chr4 | 47881739 | –0.68207 | 4.5578E–09 |
| *TEC* | CN_1078291 | Chr4 | 47881855 | –0.61325 | 2.57797E–08 |
| *TEC* | SNP_A-4198749 | Chr4 | 47834509 | –0.3988 | 2.49537E–07 |
| *TEC* | CN_1080429 | Chr4 | 47923985 | 0.65616 | 6.13786E–07 |
| *TEC* | CN_1080430 | Chr4 | 47924002 | 0.623503 | 7.91151E–07 |
| *TEC* | SNP_A-2218046 | Chr4 | 47868704 | –0.65308 | 8.59661E–07 |
| *TFEB* | CN_1162528 | Chr6 | 41785046 | 0.865377 | 1.26302E–09 |
| *TFEB* | CN_324650 | Chr6 | 41784971 | 0.855633 | 1.80992E–07 |
| *TGFB2* | SNP_A-8716258 | Chr1 | 216648240 | 0.951753 | 3.06867E–13 |
| *TGFB2* | SNP_A-8535390 | Chr1 | 216646267 | 0.70145 | 1.76561E–07 |
| *TGFBR2* | SNP_A-4209390 | Chr3 | 30693946 | –1.1232 | 9.88075E–22 |
| *TGFBR2* | SNP_A-2196130 | Chr3 | 30682119 | –0.72459 | 1.46087E–13 |
| *TGFBR2* | SNP_A-4209391 | Chr3 | 30673100 | –1.13016 | 3.35506E–13 |
| *TGFBR2* | CN_982019 | Chr3 | 30694255 | –0.64557 | 3.61735E–11 |
| *TGFBR2* | CN_982007 | Chr3 | 30673423 | –0.63159 | 6.93849E–11 |
| *TGFBR2* | CN_982013 | Chr3 | 30682280 | –0.6979 | 7.88935E–10 |
| *TGFBR2* | SNP_A-8685724 | Chr3 | 30673396 | –0.73227 | 1.97503E–08 |
| *THEM4* | CN_450786 | Chr1 | 150136481 | 0.838537 | 2.6061E–12 |
| *THEM4* | CN_450787 | Chr1 | 150140661 | 0.605333 | 5.82658E–07 |
| *TLN2* | SNP_A-2142487 | Chr15 | 60919028 | 1.1416 | 2.72703E–16 |
| *TLN2* | SNP_A-8700448 | Chr15 | 60907209 | 0.775387 | 7.23681E–13 |
| *TLN2* | SNP_A-2124811 | Chr15 | 60869299 | –0.48299 | 9.60445E–10 |
| *TLN2* | CN_707756 | Chr15 | 60750741 | –0.68345 | 1.84808E–09 |
| *TLN2* | SNP_A-2212713 | Chr15 | 60905443 | 0.762803 | 4.67072E–09 |
| *TLN2* | CN_707820 | Chr15 | 60919363 | 0.54688 | 6.61089E–08 |
| *TLN2* | CN_707803 | Chr15 | 60868625 | –0.61573 | 7.67002E–08 |
| *TLN2* | CN_707757 | Chr15 | 60751162 | –0.45044 | 3.67958E–07 |
| *TLN2* | CN_707760 | Chr15 | 60757043 | –0.39858 | 5.28378E–07 |
| *TLN2* | CN_707759 | Chr15 | 60756534 | –0.38172 | 1.26914E–06 |
| *TLR5* | CN_472810 | Chr1 | 221367289 | 0.913033 | 2.86556E–15 |
| *TLR5* | SNP_A-1929451 | Chr1 | 221378563 | 0.820123 | 1.95608E–11 |
| *TLR5* | SNP_A-8511556 | Chr1 | 221378486 | 0.75533 | 2.02999E–11 |
| *TLR5* | SNP_A-8588426 | Chr1 | 221378230 | 0.723113 | 9.3479E–10 |
| *TLR5* | SNP_A-2189503 | Chr1 | 221378468 | 0.661873 | 1.87243E–09 |
| *TLR5* | SNP_A-1887576 | Chr1 | 221367336 | 0.42326 | 3.28613E–07 |
| *TLR7* | CN_936211 | ChrX | 12795690 | 1.195757 | 4.22243E–14 |
| *TLR7* | SNP_A-2092249 | ChrX | 12795499 | 1.161773 | 8.20203E–14 |
| *TLR7* | CN_936250 | ChrX | 12804837 | 0.711353 | 5.21409E–11 |
| *TLR7* | CN_938328 | ChrX | 12807209 | 0.734687 | 4.64687E–10 |
| *TLR7* | CN_938329 | ChrX | 12807640 | 0.444853 | 2.71406E–07 |
| *TLR7* | CN_938355 | ChrX | 12814715 | 0.470683 | 2.94689E–07 |
| *TLR7* | CN_936243 | ChrX | 12803339 | 0.564993 | 8.33584E–07 |
| *TLR7* | CN_938351 | ChrX | 12814366 | 0.442467 | 1.43213E–06 |
| *TLR8* | SNP_A-8688415 | ChrX | 12845033 | 0.690263 | 3.45819E–12 |
| *TLR8* | SNP_A-8531994 | ChrX | 12844894 | 0.514993 | 1.64487E–08 |
| *TNFAIP3* | SNP_A-1957616 | Chr6 | 138239517 | –0.85128 | 2.69832E–11 |
| *TNFAIP3* | CN_1165731 | Chr6 | 138240592 | –0.68414 | 3.89147E–08 |
| *TNFRSF11B* | SNP_A-4223042 | Chr8 | 120016597 | 0.614137 | 5.99838E–10 |
| *TNFRSF11B* | SNP_A-4203937 | Chr8 | 120016611 | 0.54835 | 8.08826E–10 |
| *TNFRSF19* | CN_660176 | Chr13 | 23078370 | –0.61249 | 9.8064E–10 |
| *TNFRSF19* | SNP_A-2131243 | Chr13 | 23087023 | 0.783473 | 3.23315E–09 |
| *TNFRSF19* | SNP_A-4285479 | Chr13 | 23078318 | –0.33528 | 5.35457E–08 |
| *TNFRSF19* | CN_660179 | Chr13 | 23088593 | 0.438037 | 1.11176E–06 |
| *TNFRSF21* | SNP_A-8689991 | Chr6 | 47385528 | 1.213547 | 1.49112E–15 |
| *TNFRSF21* | CN_1171274 | Chr6 | 47315260 | 0.94798 | 4.08637E–11 |
| *TNFRSF21* | SNP_A-8528028 | Chr6 | 47315040 | 0.49101 | 2.10023E–07 |
| *TNFSF11* | SNP_A-8485892 | Chr13 | 42069568 | –0.43375 | 4.07672E–09 |
| *TNFSF11* | SNP_A-8712018 | Chr13 | 42069666 | –0.48785 | 4.23491E–09 |
| *TNFSF11* | CN_651846 | Chr13 | 42070287 | –0.54777 | 6.63687E–07 |
| *TNFSF4* | SNP_A-1824873 | Chr1 | 171435020 | 0.503873 | 4.69954E–10 |
| *TNFSF4* | CN_438121 | Chr1 | 171434989 | 0.704177 | 2.16791E–07 |
| *TNFSF8* | SNP_A-1833863 | Chr9 | 116731379 | –0.67161 | 8.23679E–13 |
| *TNFSF8* | SNP_A-2090037 | Chr9 | 116707963 | –0.83987 | 1.13817E–10 |
| *TNFSF8* | SNP_A-4228037 | Chr9 | 116707264 | –0.67093 | 2.76165E–08 |
| *TNFSF8* | SNP_A-2077631 | Chr9 | 116731091 | –0.58799 | 1.62508E–07 |
| *TNIP1* | SNP_A-4278468 | Chr5 | 150428338 | –0.6061 | 3.63376E–14 |
| *TNIP1* | SNP_A-8307101 | Chr5 | 150415673 | –0.6657 | 7.31617E–14 |
| *TNIP1* | SNP_A-2112356 | Chr5 | 150428840 | –0.78774 | 1.00712E–10 |
| *TNIP1* | CN_1100247 | Chr5 | 150418211 | –0.58074 | 4.86612E–08 |
| *TRAF5* | CN_476998 | Chr1 | 209611310 | 0.820537 | 1.4079E–10 |
| *TRAF5* | SNP_A-4237216 | Chr1 | 209611717 | 0.713867 | 2.56503E–09 |
| *TREM1* | SNP_A-2101875 | Chr6 | 41352545 | –0.91710 | 3.38317E–13 |
| *TREM1* | CN_1160460 | Chr6 | 41353414 | –0.58784 | 2.86538E–08 |
| *TREM1* | SNP_A-1847549 | Chr6 | 41354451 | –0.47036 | 4.43445E–08 |
| *TRGV9* | SNP_A-1899805 | Chr7 | 38324937 | 1.52573 | 8.25861E–16 |
| *TRGV9* | SNP_A-8641150 | Chr7 | 38324752 | 0.555257 | 1.09789E–07 |
| *TSC1* | CN_1317354 | Chr9 | 134784987 | 0.631503 | 1.5847E–09 |
| *TSC1* | CN_383269 | Chr9 | 134784967 | 0.54735 | 2.60961E–08 |
| *TSC1* | CN_1319519 | Chr9 | 134800996 | –0.6143 | 5.29138E–08 |
| *TSC1* | CN_1319521 | Chr9 | 134801055 | –0.56396 | 3.04402E–07 |
| *TSC1* | CN_1319520 | Chr9 | 134801007 | –0.52185 | 1.01161E–06 |
| *VTCN1* | SNP_A-2208522 | Chr1 | 117516272 | –0.84103 | 1.40269E–15 |
| *VTCN1* | SNP_A-1963785 | Chr1 | 117516549 | –0.60966 | 1.94865E–14 |
| *VTCN1* | CN_437465 | Chr1 | 117516564 | –0.55195 | 1.53608E–07 |
| *YWHAQ* | CN_880738 | Chr2 | 9681065 | 0.804217 | 4.1342E–09 |
| *YWHAQ* | CN_880729 | Chr2 | 9679386 | 0.64994 | 1.06422E–06 |
| *ZEB1* | CN_491185 | Chr10 | 31653932 | 1.20137 | 3.63927E–10 |
| *ZEB1* | CN_491187 | Chr10 | 31657661 | 0.568293 | 1.96729E–07 |

*Probe ID in the Affymetrix Gene Chip Human Mapping 6.0 set.

† NCBI36/hg18.

‡ Paired Student's *t*-test.

Abbreviations: Chr., chromosome; FC, log(Fold changes), HCCs vs. adjacent non-tumor tissues.
