# Supplementary material for: Relatively Small Contribution of Methylation and Genomic Copy Number Aberration to the Aberrant Expression of Inflammation-Related Genes in HBV-Related Hepatocellular Carcinoma
Source: PLoS One. 2015 May 12;10(5):e0126836. doi: 10.1371/journal.pone.0126836 (PMC4429029; doi:10.1371/journal.pone.0126836)
Supplement: S6 Table — (DOC) [file pone.0126836.s008.doc]

**S6 Table. Inflammation-related Genes with DNA Methylation Changes Associated with Inverse Expression Changes in HCC**

| **Symbol** | **HUGO Gene Name*** | **Location** | **Correlation (r)†** | **Methylation**‡ | **Methylation Validation** | **Expression Changes**‡ | **Expression Changes Validation** |
| --- | --- | --- | --- | --- | --- | --- | --- |
| *BCL2* | B-cell CLL/lymphoma 2 | 18q21.3 | –0.347 | Hypermethylated | GSE54503 | Down-regulated | GSE25079 |
| *CCL20* | chemokine (C-C motif) ligand 20 | 2q36.3 | –0.379 | Hypomethylated | GSE54503, GSE37988 | Up-regulated | GSE14520, GSE25079 |
| *CR1* | complement component (3b/4b) receptor 1 (Knops blood group) | 1q32 | –0.309 | Hypermethylated | GSE54503 | Down-regulated | GSE14520, GSE25079 |
| *ESR1* | estrogen receptor 1 | 6q24-q27 | –0.421 | Hypermethylated | GSE54503 | Down-regulated | GSE14520, GSE25079 |
| *FYN* | FYN oncogene related to SRC, FGR, YES | 6q21 | –0.362 | Hypermethylated | GSE54503 | Down-regulated | GSE14520, GSE25079 |
| *LAG3* | lymphocyte-activation gene 3 | 12p13.3 | –0.297 | Hypermethylated | GSE54503 | Down-regulated | GSE14520, GSE25079 |
| *NRAS* | neuroblastoma RAS viral (v-ras) oncogene homolog | 1p13.2 | –0.542 | Hypomethylated | N/A | Up-regulated | GSE14520, GSE25079 |
| *PRKCB* | protein kinase C, beta | 16p12 | –0.356 | Hypermethylated | GSE54503 | Down-regulated | GSE14520, GSE25079 |
| *PTPN13* | protein tyrosine phosphatase, non-receptor type 13 (APO-1/CD95 (Fas)-associated phosphatase) | 4q21.3 | –0.556 | Hypermethylated | GSE54503 | Down-regulated | GSE14520, GSE25079 |
| *SOCS2* | suppressor of cytokine signaling 2 | 12q | –0.408 | Hypermethylated | GSE54503 | Down-regulated | GSE14520, GSE25079 |
| *SPTAN1* | spectrin, alpha, non-erythrocytic 1 | 9q34.11 | –0.400 | Hypomethylated | GSE54503 | Up-regulated | GSE14520, GSE25079 |

* From HGNC database, http://www.genenames.org/.

†Correlation of DNA methylation and expression.

‡Results in this study.

Abbreviations: N/A, Not available.
