# Supplementary material for: Relatively Small Contribution of Methylation and Genomic Copy Number Aberration to the Aberrant Expression of Inflammation-Related Genes in HBV-Related Hepatocellular Carcinoma
Source: PLoS One. 2015 May 12;10(5):e0126836. doi: 10.1371/journal.pone.0126836 (PMC4429029; doi:10.1371/journal.pone.0126836)
Supplement: S7 Table — (DOC) [file pone.0126836.s009.doc]

**S7 Table. Inflammation-related Genes with SCNAs Associated with Expression Changes in HCC**

| **Symbol** | **HUGO Gene Name*** | **Location** | **Correlation (r)**† | **SCNAs**‡ | **SCNAs Validation** | **Expression Changes**‡ | **Expression Changes Validation** |
| --- | --- | --- | --- | --- | --- | --- | --- |
| *ADCY1* | adenylate cyclase 1 (brain) | 7p13-p12 | 0.549 | Deletion | N/A | Down-regulated | GSE14520,GSE25079 |
| *ADCY5* | adenylate cyclase 5 | 3q21.1 | 0.680 | Deletion | N/A | Down-regulated | GSE25079 |
| *AKR1C3* | aldo-keto reductase family 1, member C3 | 10p15-p14 | 0.629 | Copy number gains | N/A | Up-regulated | GSE14520 |
| *ALCAM* | activated leukocyte cell adhesion molecule | 3q13.1 | 0.469 | Copy number gains | N/A | Up-regulated | GSE14520,GSE25079 |
| *ATF1* | activating transcription factor 1 | 12q13 | 0.410 | Copy number gains | GSE38323 | Up-regulated | GSE14520 |
| *B3GAT1* | beta-1,3-glucuronyltransferase 1 (glucuronosyltransferase P) | 11q25 | 0.574 | Deletion | N/A | Down-regulated | GSE14520,GSE25079 |
| *BCL2* | B-cell CLL/lymphoma 2 | 18q21.3 | 0.527 | Deletion | GSE38323 | Down-regulated | GSE25079 |
| *C8A* | complement component 8, alpha polypeptide | 1p32.2 | 0.709 | Deletion | GSE38323 | Down-regulated | GSE14520,GSE25079 |
| *C8B* | complement component 8, beta polypeptide | 1p32.2 | 0.551 | Deletion | GSE38323 | Down-regulated | GSE14520,GSE25079 |
| *CAMK4* | calcium/calmodulin-dependent protein kinase IV | 5q22.1 | 0.603 | Deletion | GSE38323 | Down-regulated | GSE25079 |
| *CAPN1* | calpain 1, (mu/I) large subunit | 11q13 | 0.463 | Copy number gains | N/A | Up-regulated | GSE14520,GSE25079 |
| *CREB1* | cAMP responsive element binding protein 1 | 2q34 | 0.632 | Copy number gains | N/A | Up-regulated | GSE14520,GSE25079 |
| *CSF3R* | colony stimulating factor 3 receptor (granulocyte) | 1p35-p34.3 | 0.488 | Deletion | GSE38323 | Down-regulated | GSE25079 |
| *CTTN* | cortactin | 11q13 | 0.381 | Copy number gains | N/A | Up-regulated | GSE14520,GSE25079 |
| *CXCL12* | chemokine (C-X-C motif) ligand 12 | 10q11.1 | 0.690 | Deletion | N/A | Down-regulated | GSE14520,GSE25079 |
| *CXCL14* | chemokine (C-X-C motif) ligand 14 | 5q31 | 0.785 | Deletion | GSE38323 | Down-regulated | GSE14520,GSE25079 |
| *DAP3* | death associated protein 3 | 1q22 | 0.611 | Copy number gains | N/A | Up-regulated | GSE14520,GSE25079 |
| *EDNRB* | endothelin receptor type B | 13q22 | 0.454 | Deletion | GSE38323 | Down-regulated | GSE14520,GSE25079 |
| *ENAH* | enabled homolog (Drosophila) | 1q32.2 | 0.785 | Copy number gains | N/A | Up-regulated | GSE14520,GSE25079 |
| *ESR1* | estrogen receptor 1 | 6q24-q27 | 0.819 | Deletion | GSE38323 | Down-regulated | GSE14520,GSE25079 |
| *FAF1* | Fas (TNFRSF6) associated factor 1 | 1p32.3 | 0.542 | Copy number gains | GSE38323 | Up-regulated | GSE14520,GSE25079 |
| *FLT3* | fms-related tyrosine kinase 3 | 13q12 | 0.376 | Deletion | GSE38323 | Down-regulated | GSE14520,GSE25079 |
| *FOXO1* | forkhead box O1 | 13q14.1 | 0.339 | Deletion | GSE38323 | Down-regulated | GSE14520,GSE25079 |
| *FYN* | FYN oncogene related to SRC, FGR, YES | 6q21 | 0.442 | Deletion | GSE38323 | Down-regulated | GSE14520,GSE25079 |
| *GRB2* | growth factor receptor-bound protein 2 | 17q24-q25 | 0.484 | Copy number gains | N/A | Up-regulated | GSE14520,GSE25079 |
| *IL16* | interleukin 16 | 15q26.3 | 0.530 | Deletion | N/A | Down-regulated | GSE25079 |
| *IL18R1* | interleukin 18 receptor 1 | 2q12 | 0.646 | Deletion | N/A | Down-regulated | GSE14520,GSE25079 |
| *IL1RL1* | interleukin 1 receptor-like 1 | 2q12 | 0.560 | Deletion | N/A | Down-regulated | GSE14520,GSE25079 |
| *IRF8* | interferon regulatory factor 8 | 16q24.1 | 0.481 | Deletion | N/A | Down-regulated | GSE14520,GSE25079 |
| *ITGA6* | integrin, alpha 6 | 2q31.1 | 0.601 | Copy number gains | GSE38323 | Up-regulated | GSE14520,GSE25079 |
| *MAP2K3* | mitogen-activated protein kinase kinase 3 | 17q11.2 | 0.503 | Deletion | N/A | Down-regulated | GSE14520 |
| *MARCO* | macrophage receptor with collagenous structure | 2q14.2 | 0.764 | Deletion | N/A | Down-regulated | GSE14520,GSE25079 |
| *MASP1* | mannan-binding lectin serine peptidase 1 (C4/C2 activating component of Ra-reactive factor) | 3q27-q28 | 0.508 | Deletion | N/A | Down-regulated | GSE14520,GSE25079 |
| *NCOA2* | nuclear receptor coactivator 2 | 8q13.3 | 0.617 | Copy number gains | N/A | Up-regulated | GSE14520,GSE25079 |
| *NLRP3* | NLR family, pyrin domain containing 3 | 1q44 | 0.513 | Copy number gains | N/A | Up-regulated | GSE25079 |
| *PARP1* | poly (ADP-ribose) polymerase 1 | 1q41-q42 | 0.735 | Copy number gains | N/A | Up-regulated | GSE14520,GSE25079 |
| *PDE1A* | phosphodiesterase 1A, calmodulin-dependent | 2q32.1 | 0.459 | Deletion | GSE38323 | Down-regulated | GSE25079 |
| *PDE2A* | phosphodiesterase 2A, cGMP-stimulated | 11q13.1-q14.1 | 0.638 | Deletion | N/A | Down-regulated | GSE14520,GSE25079 |
| *PDGFRA* | platelet-derived growth factor receptor, alpha polypeptide | 4q12 | 0.682 | Deletion | N/A | Down-regulated | GSE14520,GSE25079 |
| *PIAS1* | protein inhibitor of activated STAT, 1 | 15q | 0.479 | Copy number gains | N/A | Up-regulated | GSE14520 |
| *PIK3CB* | phosphatidylinositol-4,5-bisphosphate 3-kinase, catalytic subunit beta | 3q22.3 | 0.703 | Copy number gains | N/A | Up-regulated | GSE14520,GSE25079 |
| *PLCB1* | phospholipase C, beta 1 (phosphoinositide-specific) | 20p12 | 0.667 | Copy number gains | N/A | Up-regulated | GSE14520,GSE25079 |
| *PPP2CA* | protein phosphatase 2, catalytic subunit, alpha isozyme | 5q31.1 | 0.525 | Copy number gains | GSE38323 | Up-regulated | GSE14520 |
| *PRKCB* | protein kinase C, beta | 16p12 | 0.530 | Deletion | N/A | Down-regulated | GSE14520,GSE25079 |
| *PTK2* | protein tyrosine kinase 2 | 8q24.3 | 0.725 | Copy number gains | N/A | Up-regulated | GSE14520,GSE25079 |
| *PTPN13* | protein tyrosine phosphatase, non-receptor type 13 (APO-1/CD95 (Fas)-associated phosphatase) | 4q21.3 | 0.722 | Deletion | GSE38323 | Down-regulated | GSE14520,GSE25079 |
| *RAF1* | v-raf-1 murine leukemia viral oncogene homolog 1 | 3p25 | 0.549 | Copy number gains | GSE38323 | Up-regulated | GSE14520,GSE25079 |
| *RHEB* | Ras homolog enriched in brain | 7q36 | 0.648 | Copy number gains | GSE38323 | Up-regulated | GSE14520,GSE25079 |
| *STAT4* | signal transducer and activator of transcription 4 | 2q32.2-q32.3 | 0.551 | Deletion | GSE38323 | Down-regulated | GSE14520,GSE25079 |
| *TDP2* | tyrosyl-DNA phosphodiesterase 2 | 6p22.3-p22.1 | 0.726 | Copy number gains | N/A | Up-regulated | GSE14520 |
| *THEM4* | thioesterase superfamily member 4 | 1q21.3 | 0.616 | Copy number gains | N/A | Up-regulated | GSE25079 |
| *TNFSF8* | tumor necrosis factor (ligand) superfamily, member 8 | 9q33 | 0.458 | Deletion | N/A | Down-regulated | GSE14520 |
| *TSC1* | tuberous sclerosis 1 | 9q34 | 0.491 | Copy number gains | N/A | Up-regulated | GSE25079 |
| *VTCN1* | V-set domain containing T cell activation inhibitor 1 | 1p12 | 0.562 | Deletion | N/A | Down-regulated | GSE14520,GSE25079 |
| *YWHAQ* | tyrosine 3-monooxygenase/tryptophan 5-monooxygenase activation protein, theta | 2p25.2-p25.1 | 0.638 | Copy number gains | N/A | Up-regulated | GSE14520 |
| *ZEB1* | zinc finger E-box binding homeobox 1 | 10p11.22 | 0.520 | Copy number gains | N/A | Up-regulated | N/A |

* From HGNC database, http://www.genenames.org/.

†Correlation of SCNAs and expression.

‡Results in this study.

Abbreviations: N/A, Not available.
