# Supplementary material for: Relatively Small Contribution of Methylation and Genomic Copy Number Aberration to the Aberrant Expression of Inflammation-Related Genes in HBV-Related Hepatocellular Carcinoma
Source: PLoS One. 2015 May 12;10(5):e0126836. doi: 10.1371/journal.pone.0126836 (PMC4429029; doi:10.1371/journal.pone.0126836)
Supplement: S8 Table — (DOC) [file pone.0126836.s010.doc]

**S8 Table. 200 Aberrantly Expressed Inflammation-related Genes Validated in GSE14520 Dataset**

| **ID*** | **Symbol** | **P value**† | **FC** |
| --- | --- | --- | --- |
| 213245_at | *ADCY1* | 2.27E–31 | –1.38171 |
| 209160_at | *AKR1C3* | 1.04E–72 | 1.78422 |
| 201952_at | *ALCAM* | 1.16E–14 | 0.473583 |
| 201951_at | *ALCAM* | 1.47E–16 | 0.520223 |
| 204859_s_at | *APAF1* | 1.78E–29 | 0.492559 |
| 222103_at | *ATF1* | 7.75E–21 | 0.598223 |
| 205446_s_at | *ATF2* | 1.84E–19 | 0.352708 |
| 212984_at | *ATF2* | 2.84E–54 | 0.779342 |
| 219521_at | *B3GAT1* | 3.98E–40 | –0.70119 |
| 203728_at | *BAK1* | 1.48E–13 | 0.218017 |
| 204832_s_at | *BMPR1A* | 1.58E–20 | 0.345712 |
| 213578_at | *BMPR1A* | 2.61E–27 | 0.679356 |
| 218232_at | *C1QA* | 1.77E–17 | –0.90676 |
| 212067_s_at | *C1R* | 4.01E–69 | –1.80422 |
| 210168_at | *C6* | 5.72E–57 | –2.45405 |
| 202992_at | *C7* | 4.40E–81 | –3.28133 |
| 206305_s_at | *C8A* | 5.60E–69 | –2.53834 |
| 206979_at | *C8B* | 3.87E–45 | –1.84131 |
| 206727_at | *C9* | 1.90E–64 | –3.74962 |
| 200752_s_at | *CAPN1* | 8.08E–09 | 0.274717 |
| 208683_at | *CAPN2* | 1.89E–20 | 0.735642 |
| 200001_at | *CAPNS1* | 2.01E–20 | 0.559995 |
| 222201_s_at | *CASP8AP2* | 6.03E–29 | 0.687804 |
| 211922_s_at | *CAT* | 7.78E–37 | –1.40859 |
| 201432_at | *CAT* | 3.22E–48 | –1.18499 |
| 215573_at | *CAT* | 1.78E–24 | –0.57264 |
| 210072_at | *CCL19* | 9.14E–34 | –1.7892 |
| 205476_at | *CCL20* | 4.89E–60 | 3.123901 |
| 204606_at | *CCL21* | 2.43E–34 | –1.13954 |
| 205098_at | *CCR1* | 9.98E–08 | –0.41539 |
| 205099_s_at | *CCR1* | 1.92E–10 | –0.32255 |
| 201743_at | *CD14* | 4.40E–56 | –1.85241 |
| 207840_at | *CD160* | 6.36E–50 | –0.55126 |
| 205987_at | *CD1C* | 8.01E–17 | –0.35837 |
| 205789_at | *CD1D* | 1.40E–82 | –1.76813 |
| 208592_s_at | *CD1E* | 1.05E–06 | –0.17089 |
| 215784_at | *CD1E* | 1.64E–07 | –0.13677 |
| 207315_at | *CD226* | 1.10E–14 | –0.19711 |
| 220307_at | *CD244* | 1.96E–38 | –0.36011 |
| 210031_at | *CD247* | 1.69E–14 | –0.32722 |
| 209933_s_at | *CD300A* | 1.06E–35 | –0.81973 |
| 217078_s_at | *CD300A* | 9.64E–10 | –0.11476 |
| 206120_at | *CD33* | 4.42E–20 | –0.32835 |
| 209543_s_at | *CD34* | 2.91E–35 | 0.536769 |
| 203547_at | *CD4* | 1.00E–77 | –1.5963 |
| 208783_s_at | *CD46* | 1.04E–29 | 0.719402 |
| 211574_s_at | *CD46* | 3.57E–17 | 0.721168 |
| 207549_x_at | *CD46* | 6.98E–17 | 0.734118 |
| 203214_x_at | *CDK1* | 2.41E–50 | 1.140364 |
| 210559_s_at | *CDK1* | 3.95E–72 | 1.658584 |
| 203213_at | *CDK1* | 2.84E–96 | 2.472948 |
| 205382_s_at | *CFD* | 2.10E–18 | –0.49413 |
| 203854_at | *CFI* | 2.13E–49 | –1.41878 |
| 206380_s_at | *CFP* | 6.35E–96 | –1.4411 |
| 209666_s_at | *CHUK* | 3.39E–26 | 0.659973 |
| 206244_at | *CR1* | 1.20E–18 | –0.18564 |
| 204312_x_at | *CREB1* | 7.46E–11 | 0.224194 |
| 204314_s_at | *CREB1* | 4.63E–13 | 0.253468 |
| 204313_s_at | *CREB1* | 1.93E–18 | 0.462195 |
| 203104_at | *CSF1R* | 7.39E–23 | –0.65699 |
| 212075_s_at | *CSNK2A1* | 8.72E–19 | 0.605235 |
| 206075_s_at | *CSNK2A1* | 1.95E–29 | 0.613447 |
| 212072_s_at | *CSNK2A1* | 3.72E–43 | 0.853197 |
| 201390_s_at | *CSNK2B* | 2.35E–51 | 0.707206 |
| 201533_at | *CTNNB1* | 5.12E–24 | 0.480392 |
| 214074_s_at | *CTTN* | 5.78E–08 | 0.263228 |
| 201059_at | *CTTN* | 1.31E–20 | 0.559172 |
| 209687_at | *CXCL12* | 4.43E–97 | –3.17517 |
| 203666_at | *CXCL12* | 4.53E–94 | –2.43164 |
| 218002_s_at | *CXCL14* | 6.43E–153 | –3.46169 |
| 209774_x_at | *CXCL2* | 3.21E–63 | –2.68069 |
| 207008_at | *CXCR2* | 7.89E–14 | –0.2911 |
| 208822_s_at | *DAP3* | 8.19E–89 | 1.20577 |
| 216038_x_at | *DAXX* | 2.66E–13 | 0.23304 |
| 201763_s_at | *DAXX* | 3.73E–26 | 0.433104 |
| 219350_s_at | *DIABLO* | 1.07E–11 | 0.293622 |
| 219452_at | *DPEP2* | 3.82E–16 | –0.22094 |
| 201041_s_at | *DUSP1* | 5.72E–55 | –1.8486 |
| 201044_x_at | *DUSP1* | 4.21E–32 | –1.33432 |
| 204273_at | *EDNRB* | 2.32E–49 | –1.46099 |
| 204271_s_at | *EDNRB* | 1.39E–29 | –1.12105 |
| 206701_x_at | *EDNRB* | 1.12E–14 | –0.56752 |
| 217820_s_at | *ENAH* | 4.34E–84 | 1.95514 |
| 201809_s_at | *ENG* | 4.63E–33 | –0.96048 |
| 201808_s_at | *ENG* | 5.90E–09 | –0.13807 |
| 205225_at | *ESR1* | 3.64E–117 | –2.81401 |
| 211627_x_at | *ESR1* | 4.58E–30 | –0.48521 |
| 211234_x_at | *ESR1* | 4.59E–09 | –0.17222 |
| 211235_s_at | *ESR1* | 5.58E–08 | –0.15447 |
| 215552_s_at | *ESR1* | 8.96E–11 | –0.14872 |
| 218080_x_at | *FAF1* | 1.18E–29 | 0.539364 |
| 204781_s_at | *FAS* | 6.99E–41 | –0.99236 |
| 204780_s_at | *FAS* | 5.73E–19 | –0.74804 |
| 215719_x_at | *FAS* | 1.10E–14 | –0.54761 |
| 216252_x_at | *FAS* | 1.64E–11 | –0.39583 |
| 210865_at | *FASLG* | 1.79E–09 | –0.15566 |
| 210889_s_at | *FCGR2B* | 1.67E–50 | –1.38326 |
| 206674_at | *FLT3* | 3.51E–18 | –0.24046 |
| 209189_at | *FOS* | 1.84E–106 | –3.72919 |
| 204420_at | *FOSL1* | 4.91E–12 | –0.26758 |
| 202724_s_at | *FOXO1* | 1.74E–39 | –1.0703 |
| 202723_s_at | *FOXO1* | 5.36E–37 | –0.99939 |
| 210105_s_at | *FYN* | 3.33E–84 | –1.6537 |
| 216033_s_at | *FYN* | 1.16E–56 | –1.20484 |
| 212486_s_at | *FYN* | 8.34E–48 | –0.62126 |
| 215075_s_at | *GRB2* | 5.76E–10 | 0.330964 |
| 201209_at | *HDAC1* | 8.35E–32 | 0.616506 |
| 200943_at | *HMGN1* | 2.44E–57 | 0.868567 |
| 200944_s_at | *HMGN1* | 1.27E–56 | 0.968465 |
| 209709_s_at | *HMMR* | 5.71E–54 | 1.062628 |
| 207165_at | *HMMR* | 1.78E–81 | 1.959535 |
| 214328_s_at | *HSP90AA1* | 6.49E–43 | 0.607499 |
| 211969_at | *HSP90AA1* | 6.77E–32 | 0.611088 |
| 211968_s_at | *HSP90AA1* | 1.34E–28 | 0.678735 |
| 210211_s_at | *HSP90AA1* | 2.45E–37 | 0.787292 |
| 200064_at | *HSP90AB1* | 2.94E–76 | 1.18277 |
| 214359_s_at | *HSP90AB1* | 3.57E–37 | 1.31998 |
| 200599_s_at | *HSP90B1* | 7.02E–23 | 0.427355 |
| 200598_s_at | *HSP90B1* | 7.46E–08 | 0.438852 |
| 205824_at | *HSPB2* | 6.32E–08 | –0.20033 |
| 200807_s_at | *HSPD1* | 1.96E–36 | 0.529835 |
| 200806_s_at | *HSPD1* | 7.47E–19 | 0.744763 |
| 201422_at | *IFI30* | 1.70E–24 | 0.790682 |
| 202421_at | *IGSF3* | 9.84E–40 | 1.078213 |
| 204912_at | *IL10RA* | 7.11E–19 | –0.74976 |
| 206926_s_at | *IL11* | 4.43E–16 | –0.30668 |
| 206172_at | *IL13RA2* | 3.51E–09 | –0.5159 |
| 209827_s_at | *IL16* | 7.99E–22 | –0.38289 |
| 209828_s_at | *IL16* | 2.20E–06 | –0.12427 |
| 206618_at | *IL18R1* | 1.28E–26 | –0.37993 |
| 207072_at | *IL18RAP* | 3.04E–27 | –0.37668 |
| 205067_at | *IL1B* | 9.76E–19 | –0.39208 |
| 39402_at | *IL1B* | 4.91E–22 | –0.36617 |
| 210442_at | *IL1RL1* | 1.33E–10 | –0.17264 |
| 200052_s_at | *ILF2* | 3.79E–96 | 1.854289 |
| 201234_at | *ILK* | 1.19E–07 | 0.266845 |
| 201598_s_at | *INPPL1* | 1.99E–08 | 0.239458 |
| 208436_s_at | *IRF7* | 9.68E–13 | –0.48439 |
| 204057_at | *IRF8* | 4.40E–49 | –0.99686 |
| 201389_at | *ITGA5* | 4.29E–20 | 0.607071 |
| 215177_s_at | *ITGA6* | 5.78E–43 | 1.262961 |
| 201656_at | *ITGA6* | 1.75E–73 | 1.449377 |
| 216676_x_at | *KIR3DL3* | 5.67E–10 | –0.12629 |
| 214470_at | *KLRB1* | 1.33E–25 | –0.62389 |
| 205821_at | *KLRK1* | 5.43E–36 | –0.88487 |
| 206486_at | *LAG3* | 6.31E–11 | –0.20369 |
| 205270_s_at | *LCP2* | 4.42E–16 | –0.53291 |
| 205269_at | *LCP2* | 2.92E–12 | –0.4659 |
| 207409_at | *LECT2* | 2.24E–39 | –2.41058 |
| 205876_at | *LIFR* | 5.44E–40 | –0.43609 |
| 210660_at | *LILRA1* | 2.20E–24 | –0.267 |
| 215838_at | *LILRA5* | 3.29E–08 | –0.12962 |
| 207104_x_at | *LILRB1* | 9.02E–21 | –0.33447 |
| 211336_x_at | *LILRB1* | 5.48E–20 | –0.33313 |
| 207697_x_at | *LILRB2* | 1.79E–29 | –0.53266 |
| 206856_at | *LILRB5* | 7.79E–66 | –0.73234 |
| 212089_at | *LMNA* | 1.98E–23 | 0.569156 |
| 212086_x_at | *LMNA* | 1.44E–29 | 0.6007 |
| 203411_s_at | *LMNA* | 7.84E–65 | 1.135561 |
| 208771_s_at | *LTA4H* | 2.12E–29 | 0.553183 |
| 215498_s_at | *MAP2K3* | 3.86E–34 | –0.86519 |
| 207667_s_at | *MAP2K3* | 1.39E–41 | –0.75601 |
| 215499_at | *MAP2K3* | 4.72E–34 | –0.67281 |
| 211536_x_at | *MAP3K7* | 2.13E–10 | 0.287083 |
| 211537_x_at | *MAP3K7* | 3.12E–15 | 0.452208 |
| 206854_s_at | *MAP3K7* | 1.91E–21 | 0.526416 |
| 206853_s_at | *MAP3K7* | 2.10E–30 | 0.535438 |
| 208351_s_at | *MAPK1* | 9.47E–17 | 0.337026 |
| 212271_at | *MAPK1* | 1.68E–68 | 1.109027 |
| 202530_at | *MAPK14* | 4.04E–16 | 0.385389 |
| 210570_x_at | *MAPK9* | 3.17E–32 | 0.438733 |
| 203218_at | *MAPK9* | 4.11E–70 | 1.286038 |
| 212871_at | *MAPKAPK5* | 1.10E–47 | 0.68948 |
| 205819_at | *MARCO* | 3.12E–78 | –2.02027 |
| 210680_s_at | *MASP1* | 1.51E–42 | –0.95706 |
| 206449_s_at | *MASP1* | 4.55E–45 | –0.91806 |
| 213749_at | *MASP1* | 3.30E–12 | –0.35552 |
| 207041_at | *MASP2* | 2.17E–64 | –2.24874 |
| 210798_x_at | *MASP2* | 2.82E–33 | –1.44081 |
| 207256_at | *MBL2* | 1.01E–41 | –2.09869 |
| 203496_s_at | *MED1* | 4.09E–12 | 0.289524 |
| 203497_at | *MED1* | 1.90E–19 | 0.410092 |
| 208262_x_at | *MEFV* | 1.05E–10 | –0.16031 |
| 206247_at | *MICB* | 3.43E–28 | 0.62662 |
| 205732_s_at | *NCOA2* | 5.00E–19 | 0.491777 |
| 217095_x_at | *NCR1* | 4.20E–09 | –0.18893 |
| 207860_at | *NCR1* | 7.27E–08 | –0.11085 |
| 213028_at | *NFRKB* | 1.83E–12 | 0.304313 |
| 203964_at | *NMI* | 1.15E–11 | 0.432653 |
| 202340_x_at | *NR4A1* | 2.01E–41 | –1.09616 |
| 211143_x_at | *NR4A1* | 3.51E–30 | –0.51955 |
| 210226_at | *NR4A1* | 1.04E–12 | –0.18002 |
| 202647_s_at | *NRAS* | 2.55E–59 | 1.382627 |
| 209615_s_at | *PAK1* | 2.45E–11 | 0.202551 |
| 208644_at | *PARP1* | 3.73E–90 | 1.13063 |
| 204134_at | *PDE2A* | 1.66E–68 | –0.92832 |
| 203131_at | *PDGFRA* | 4.87E–59 | –2.65743 |
| 32029_at | *PDPK1* | 1.32E–26 | –0.31994 |
| 217864_s_at | *PIAS1* | 5.10E–11 | 0.270913 |
| 203035_s_at | *PIAS3* | 3.33E–31 | 0.469294 |
| 212688_at | *PIK3CB* | 7.13E–61 | 0.908671 |
| 213222_at | *PLCB1* | 1.36E–73 | 2.192049 |
| 204046_at | *PLCB2* | 4.28E–39 | –0.45364 |
| 210388_at | *PLCB2* | 4.30E–11 | –0.16214 |
| 200846_s_at | *PPP1CA* | 5.36E–11 | 0.385731 |
| 201408_at | *PPP1CB* | 3.26E–08 | 0.288707 |
| 201409_s_at | *PPP1CB* | 1.94E–18 | 0.3277 |
| 201407_s_at | *PPP1CB* | 1.93E–36 | 0.624637 |
| 200726_at | *PPP1CC* | 2.04E–43 | 0.798848 |
| 208652_at | *PPP2CA* | 5.92E–33 | 0.710241 |
| 200695_at | *PPP2R1A* | 1.59E–28 | 0.591361 |
| 208680_at | *PRDX1* | 2.76E–19 | 0.511733 |
| 213052_at | *PRKAR2A* | 1.73E–16 | 0.394161 |
| 203680_at | *PRKAR2B* | 1.82E–08 | –0.36758 |
| 209685_s_at | *PRKCB* | 9.67E–08 | –0.29369 |
| 211746_x_at | *PSMA1* | 3.90E–42 | 0.616649 |
| 201676_x_at | *PSMA1* | 1.98E–42 | 0.634534 |
| 210759_s_at | *PSMA1* | 4.41E–35 | 0.646862 |
| 208799_at | *PSMB5* | 1.81E–54 | 0.740013 |
| 215894_at | *PTGDR* | 2.65E–15 | –0.17469 |
| 215937_at | *PTGDR* | 6.02E–12 | –0.17253 |
| 200627_at | *PTGES3* | 6.11E–61 | 0.877817 |
| 206187_at | *PTGIR* | 7.57E–24 | –0.29931 |
| 208131_s_at | *PTGIS* | 1.13E–44 | –1.37317 |
| 211892_s_at | *PTGIS* | 1.10E–09 | –0.12402 |
| 207821_s_at | *PTK2* | 1.69E–27 | 0.702764 |
| 208820_at | *PTK2* | 1.23E–54 | 0.983161 |
| 212610_at | *PTPN11* | 1.11E–09 | 0.264816 |
| 209896_s_at | *PTPN11* | 6.57E–07 | 0.357815 |
| 209895_at | *PTPN11* | 1.35E–08 | 0.377733 |
| 202006_at | *PTPN12* | 7.77E–35 | 0.825613 |
| 204201_s_at | *PTPN13* | 1.74E–20 | –0.37039 |
| 201244_s_at | *RAF1* | 2.28E–17 | 0.457148 |
| 202677_at | *RASA1* | 2.73E–16 | 0.485097 |
| 210621_s_at | *RASA1* | 8.86E–17 | 0.561754 |
| 201453_x_at | *RHEB* | 6.61E–70 | 0.897317 |
| 213404_s_at | *RHEB* | 2.78E–51 | 1.084247 |
| 200059_s_at | *RHOA* | 1.68E–24 | 0.655919 |
| 209544_at | *RIPK2* | 3.86E–19 | 0.374868 |
| 209545_s_at | *RIPK2* | 1.55E–35 | 0.948949 |
| 214578_s_at | *ROCK1* | 6.47E–08 | 0.234512 |
| 213044_at | *ROCK1* | 5.75E–23 | 0.434135 |
| 202762_at | *ROCK2* | 8.18E–24 | 0.66158 |
| 211578_s_at | *RPS6KB1* | 2.41E–07 | 0.219597 |
| 205863_at | *S100A12* | 1.74E–21 | –0.25371 |
| 206995_x_at | *SCARF1* | 5.92E–37 | –0.51064 |
| 218711_s_at | *SDPR* | 1.00E–13 | –0.31733 |
| 206049_at | *SELP* | 8.29E–20 | –0.4552 |
| 200986_at | *SERPING1* | 1.89E–43 | –1.16021 |
| 201469_s_at | *SHC1* | 2.13E–24 | 0.685514 |
| 214853_s_at | *SHC1* | 6.29E–48 | 0.943011 |
| 219519_s_at | *SIGLEC1* | 9.35E–36 | –0.82661 |
| 44673_at | *SIGLEC1* | 7.55E–49 | –0.7655 |
| 207224_s_at | *SIGLEC7* | 4.92E–36 | –0.53398 |
| 216537_s_at | *SIGLEC7* | 1.88E–24 | –0.27994 |
| 217159_x_at | *SIGLEC7* | 6.50E–23 | –0.2612 |
| 203373_at | *SOCS2* | 1.77E–44 | –1.86072 |
| 203372_s_at | *SOCS2* | 2.55E–31 | –1.06669 |
| 206359_at | *SOCS3* | 1.50E–08 | –0.26077 |
| 206360_s_at | *SOCS3* | 1.08E–11 | –0.14496 |
| 209648_x_at | *SOCS5* | 6.78E–31 | 0.526653 |
| 208127_s_at | *SOCS5* | 5.94E–38 | 0.623767 |
| 209647_s_at | *SOCS5* | 1.37E–56 | 0.993594 |
| 212780_at | *SOS1* | 1.19E–14 | 0.329226 |
| 215235_at | *SPTAN1* | 3.38E–31 | 0.668851 |
| 208611_s_at | *SPTAN1* | 5.78E–33 | 0.696334 |
| 206118_at | *STAT4* | 1.13E–24 | –0.70099 |
| 218520_at | *TBK1* | 1.08E–12 | 0.337743 |
| 220684_at | *TBX21* | 1.02E–07 | –0.15581 |
| 336_at | *TBXA2R* | 3.75E–88 | –1.07546 |
| 207554_x_at | *TBXA2R* | 4.72E–46 | –0.48625 |
| 211590_x_at | *TBXA2R* | 2.28E–33 | –0.40165 |
| 208130_s_at | *TBXAS1* | 3.27E–09 | –0.18067 |
| 202266_at | *TDP2* | 4.73E–20 | 0.611349 |
| 208851_s_at | *THY1* | 5.73E–20 | 0.631368 |
| 208850_s_at | *THY1* | 1.07E–19 | 0.637842 |
| 213869_x_at | *THY1* | 8.94E–31 | 0.956942 |
| 221060_s_at | *TLR4* | 1.41E–12 | –0.21849 |
| 211163_s_at | *TNFRSF10C* | 2.04E–07 | –0.10712 |
| 207643_s_at | *TNFRSF1A* | 7.72E–16 | –0.4176 |
| 205611_at | *TNFSF12* | 2.90E–07 | –0.17267 |
| 207216_at | *TNFSF8* | 2.18E–08 | –0.12952 |
| 204413_at | *TRAF2* | 3.54E–18 | 0.270909 |
| 206828_at | *TXK* | 6.81E–14 | –0.15022 |
| 219768_at | *VTCN1* | 2.13E–08 | –0.27185 |
| 217717_s_at | *YWHAB* | 9.56E–11 | 0.393048 |
| 217718_s_at | *YWHAB* | 9.01E–47 | 0.577866 |
| 208743_s_at | *YWHAB* | 2.30E–52 | 1.004902 |
| 201020_at | *YWHAH* | 1.23E–50 | 1.154915 |
| 212426_s_at | *YWHAQ* | 2.21E–35 | 0.726787 |
| 200693_at | *YWHAQ* | 1.73E–29 | 0.751543 |
| 213699_s_at | *YWHAQ* | 1.89E–53 | 0.770258 |
| 200639_s_at | *YWHAZ* | 2.48E–57 | 1.027012 |
| 200641_s_at | *YWHAZ* | 6.92E–25 | 1.13586 |
| 200640_at | *YWHAZ* | 9.61E–51 | 1.218773 |
| 200638_s_at | *YWHAZ* | 1.16E–46 | 1.427792 |

*ID in GSE14520 dataset.

†Student's *t*-test. Bonferroni adjustment was used to correct for multiple comparisons, and *P*<2.24×10^-6^ was considered to be statistically significant in view of the 22,268 transcripts in GSE14520 dataset.

Abbreviations: FC, log_2_ (Fold changes), HCCs vs. adjacent non-tumor tissues.
