# Supplementary material for: Relatively Small Contribution of Methylation and Genomic Copy Number Aberration to the Aberrant Expression of Inflammation-Related Genes in HBV-Related Hepatocellular Carcinoma
Source: PLoS One. 2015 May 12;10(5):e0126836. doi: 10.1371/journal.pone.0126836 (PMC4429029; doi:10.1371/journal.pone.0126836)
Supplement: S9 Table — (DOC) [file pone.0126836.s011.doc]

**S9 Table. 204 Aberrantly Expressed Inflammation-related Genes Validated in GSE25097 Dataset**

| **ID*** | **Symbol** | **P Value**† | **FC** |
| --- | --- | --- | --- |
| 100135573_TGI_at | *ADCY1* | 4.08E–18 | –1.27515031 |
| 100146795_TGI_at | *ADCY5* | 2.46E–37 | –1.03841587 |
| 100142622_TGI_at | *ADCY5* | 8.91E–10 | –0.15608585 |
| 100151757_TGI_at | *ADCY6* | 3.07E–68 | 1.09993704 |
| 100131343_TGI_at | *ALCAM* | 6.02E–20 | 0.42864509 |
| 100132005_TGI_at | *APAF1* | 4.06E–09 | 0.18708101 |
| 100143587_TGI_at | *APAF1* | 4.69E–25 | 0.40435206 |
| 100125754_TGI_at | *ATF2* | 1.42E–14 | 0.21153834 |
| 100142633_TGI_at | *B3GAT1* | 5.28E–40 | –1.46776058 |
| 100122163_TGI_at | *B3GAT1* | 0.000000613 | –0.13689524 |
| 100130192_TGI_at | *BAK1* | 2.19E–26 | 0.7205226 |
| 100158107_TGI_at | *BCL2* | 9.93E–19 | –0.72130649 |
| 100126642_TGI_at | *BCL2* | 5.13E–13 | –0.40586968 |
| 100159021_TGI_at | *C1QA* | 9.21E–41 | –1.23323144 |
| 100140213_TGI_at | *C1QC* | 2.18E–33 | –1.07293219 |
| 100145579_TGI_at | *C1R* | 2.69E–49 | –0.75618491 |
| 100144074_TGI_at | *C6* | 3.52E–36 | –1.59829195 |
| 100138202_TGI_at | *C7* | 1.52E–95 | –3.94119093 |
| 100144076_TGI_at | *C7* | 5.46E–96 | –3.79940829 |
| 100142841_TGI_at | *C7* | 1.21E–63 | –2.69478192 |
| 100154096_TGI_at | *C8A* | 1.6E–38 | –0.91991375 |
| 100128767_TGI_at | *C8B* | 4.31E–29 | –0.99427921 |
| 100133037_TGI_at | *C9* | 3.88E–48 | –3.00389979 |
| 100160316_TGI_at | *CAMK4* | 2.59E–72 | –2.1335201 |
| 100162580_TGI_at | *CAMK4* | 3.52E–41 | –0.67687484 |
| 100136373_TGI_at | *CAPN1* | 3.23E–18 | 0.35351841 |
| 100129139_TGI_at | *CAPN2* | 0.000000374 | 0.25465166 |
| 100161865_TGI_at | *CAPNS1* | 6.01E–20 | 0.25888404 |
| 100147930_TGI_at | *CASP2* | 7.26E–19 | 0.29562369 |
| 100141497_TGI_at | *CASP2* | 2.07E–42 | 0.6058663 |
| 100160940_TGI_at | *CASP8AP2* | 2.35E–34 | 0.57948155 |
| 100146869_TGI_at | *CASP8AP2* | 3.52E–26 | 0.70860371 |
| 100157261_TGI_at | *CAT* | 9.06E–18 | –0.955996 |
| 100152409_TGI_at | *CAT* | 1.93E–23 | –0.5262007 |
| 100150876_TGI_at | *CCL19* | 3.86E–64 | –3.05678914 |
| 100158427_TGI_at | *CCL20* | 1.89E–09 | 1.13948818 |
| 100137678_TGI_at | *CCL21* | 2.21E–55 | –2.70754806 |
| 100133883_TGI_at | *CCR1* | 1.6E–40 | –1.31927387 |
| 100123330_TGI_at | *CD14* | 1.09E–68 | –1.39360129 |
| 100139900_TGI_at | *CD160* | 3.84E–27 | –0.70797102 |
| 100161022_TGI_at | *CD1C* | 2.57E–42 | –1.46698294 |
| 100145467_TGI_at | *CD1C* | 6.87E–14 | –0.44392709 |
| 100145982_TGI_at | *CD1D* | 1.2E–71 | –1.88067575 |
| 100143809_TGI_at | *CD207* | 2.37E–67 | –1.58988496 |
| 100130291_TGI_at | *CD226* | 2.47E–66 | –1.17535644 |
| 100131304_TGI_at | *CD244* | 1.89E–87 | –1.96922981 |
| 100153171_TGI_at | *CD247* | 3.28E–38 | –1.01120622 |
| 100161399_TGI_at | *CD300A* | 2.41E–58 | –1.4158939 |
| 100140443_TGI_at | *CD33* | 3.47E–29 | –1.02556822 |
| 100127008_TGI_at | *CD34* | 2.13E–61 | 1.49744227 |
| 100160421_TGI_at | *CD4* | 4.39E–63 | –1.44304698 |
| 100126371_TGI_at | *CD46* | 3.28E–20 | 0.26918722 |
| 100157986_TGI_at | *CD46* | 6.06E–16 | 0.41603572 |
| 100159289_TGI_at | *CDK1* | 1.49E–66 | 1.5053132 |
| 100127504_TGI_at | *CDK1* | 4.38E–121 | 2.70644511 |
| 100159629_TGI_at | *CFD* | 5.25E–33 | –0.6758992 |
| 100139068_TGI_at | *CFI* | 2.02E–53 | –0.98344807 |
| 100145758_TGI_at | *CFP* | 1.59E–165 | –3.63891539 |
| 100153680_TGI_at | *CR1* | 2.14E–49 | –1.87038605 |
| 100152465_TGI_at | *CREB1* | 2.63E–12 | 0.34787639 |
| 100131729_TGI_at | *CREB1* | 3.89E–19 | 0.38455451 |
| 100145076_TGI_at | *CSF1R* | 6.73E–35 | –1.10573608 |
| 100146516_TGI_at | *CSF1R* | 7.77E–23 | –0.68154062 |
| 100160674_TGI_at | *CSF3R* | 6.89E–25 | –0.82501491 |
| 100154559_TGI_at | *CSNK2A1* | 2.61E–14 | 0.26666424 |
| 100122830_TGI_at | *CSNK2B* | 4.79E–12 | 0.24554941 |
| 100126428_TGI_at | *CTNNB1* | 1.96E–23 | 0.21342185 |
| 100158290_TGI_at | *CTTN* | 3.01E–11 | 0.26904305 |
| 100155317_TGI_at | *CTTN* | 1.18E–37 | 0.43435136 |
| 100132265_TGI_at | *CXCL1* | 1.83E–20 | –1.63857737 |
| 100127959_TGI_at | *CXCL12* | 3.57E–97 | –2.97572506 |
| 100130092_TGI_at | *CXCL12* | 6.21E–95 | –2.93171844 |
| 100145996_TGI_at | *CXCL14* | 5.52E–212 | –4.72710295 |
| 100161301_TGI_at | *CXCL2* | 4.37E–62 | –1.82501062 |
| 100154933_TGI_at | *CXCR1* | 5.27E–38 | –1.06567127 |
| 100145807_TGI_at | *CXCR2* | 3.15E–36 | –1.59090809 |
| 100138238_TGI_at | *DAP3* | 1.49E–70 | 0.53156297 |
| 100139014_TGI_at | *DAXX* | 3.65E–42 | 0.5737604 |
| 100127748_TGI_at | *DFFA* | 8.66E–14 | 0.32435443 |
| 100154555_TGI_at | *DFFA* | 2.71E–26 | 0.52590614 |
| 100150454_TGI_at | *DPEP2* | 6.8E–34 | –0.97382414 |
| 100132466_TGI_at | *DUSP1* | 8.12E–50 | –0.96645022 |
| 100127708_TGI_at | *DUSP1* | 1.41E–15 | –0.26596554 |
| 100157638_TGI_at | *EDNRB* | 5.12E–67 | –1.83423568 |
| 100150224_TGI_at | *EDNRB* | 5.48E–89 | –1.6485111 |
| 100136502_TGI_at | *EIF2AK2* | 1.12E–13 | 0.26522582 |
| 100129230_TGI_at | *ENAH* | 1.14E–33 | 1.04345291 |
| 100142877_TGI_at | *ENAH* | 1.42E–50 | 1.16908007 |
| 100129652_TGI_at | *ENAH* | 2.69E–59 | 1.38033754 |
| 100148256_TGI_at | *ENG* | 6.91E–50 | –1.16200289 |
| 100128697_TGI_at | *ENG* | 7.59E–21 | –0.371508 |
| 100130436_TGI_at | *ESR1* | 6.12E–77 | –2.71522245 |
| 100149370_TGI_at | *ESR1* | 3.97E–12 | –0.16361045 |
| 100156815_TGI_at | *FAF1* | 2.67E–27 | 0.41262961 |
| 100128351_TGI_at | *FAS* | 1.16E–32 | –1.15368969 |
| 100138855_TGI_at | *FAS* | 8.35E–27 | –1.13579288 |
| 100152069_TGI_at | *FASLG* | 8.48E–31 | –0.8885487 |
| 100136439_TGI_at | *FLT3* | 3.17E–50 | –1.14983347 |
| 100123020_TGI_at | *FOS* | 9.32E–87 | –2.17225642 |
| 100144250_TGI_at | *FOSL1* | 5.95E–37 | –1.28912911 |
| 100132903_TGI_at | *FOXO1* | 3.24E–40 | –1.1722604 |
| 100143818_TGI_at | *FOXO1* | 5.33E–40 | –0.93699395 |
| 100130283_TGI_at | *FOXO1* | 4.05E–65 | –0.8930757 |
| 100137017_TGI_at | *FPR2* | 1.36E–56 | –2.31332987 |
| 100127703_TGI_at | *FYN* | 6.71E–58 | –1.19606583 |
| 100144846_TGI_at | *FYN* | 8.82E–64 | –1.07303552 |
| 100158755_TGI_at | *GRB2* | 9.08E–31 | 0.33555868 |
| 100140717_TGI_at | *GRB2* | 9.06E–11 | 0.40640019 |
| 100151540_TGI_at | *HDAC1* | 3.63E–32 | 0.38308551 |
| 100129151_TGI_at | *HMMR* | 3.43E–136 | 3.18016446 |
| 100148933_TGI_at | *HSPB2* | 5.53E–24 | –0.53245898 |
| 100140087_TGI_at | *IGSF3* | 3.49E–08 | 0.1355881 |
| 100154940_TGI_at | *IGSF3* | 1.93E–75 | 2.41886239 |
| 100151494_TGI_at | *IL10RA* | 2.89E–34 | –0.99018569 |
| 100161199_TGI_at | *IL13RA2* | 3.2E–80 | –3.43432515 |
| 100122424_TGI_at | *IL16* | 1.36E–33 | –0.97431483 |
| 100153960_TGI_at | *IL16* | 9.16E–40 | –0.59317076 |
| 100136977_TGI_at | *IL18R1* | 4.84E–93 | –2.06645783 |
| 100141328_TGI_at | *IL18RAP* | 4.2E–54 | –1.40124026 |
| 100126016_TGI_at | *IL1B* | 4.45E–46 | –1.80951859 |
| 100148210_TGI_at | *IL1RL1* | 3.84E–100 | –3.05088035 |
| 100148162_TGI_at | *IL1RL1* | 1.16E–83 | –2.30350472 |
| 100140509_TGI_at | *IL6* | 7.29E–35 | –1.88244844 |
| 100157113_TGI_at | *ILF2* | 1.01E–24 | 0.54573443 |
| 100145617_TGI_at | *INPPL1* | 4.61E–26 | 0.55392289 |
| 100155637_TGI_at | *IRF7* | 7.11E–29 | –0.82422227 |
| 100121850_TGI_at | *IRF8* | 7.69E–53 | –1.31075727 |
| 100135535_TGI_at | *ITGA2* | 3.18E–25 | 0.84843657 |
| 100149502_TGI_at | *ITGA5* | 5.3E–25 | 0.54865956 |
| 100130951_TGI_at | *ITGA6* | 2.34E–99 | 1.02179735 |
| 100130112_TGI_at | *ITGAD* | 3.58E–49 | –1.88608692 |
| 100122751_TGI_at | *KIR3DL3* | 1.99E–10 | –0.2326206 |
| 100122959_TGI_at | *KLRB1* | 4.8E–49 | –1.68908934 |
| 100142237_TGI_at | *KLRK1* | 5.3E–53 | –1.87085294 |
| 100147590_TGI_at | *LAG3* | 1.84E–50 | –1.04541177 |
| 100155799_TGI_at | *LCP2* | 6.2E–40 | –1.05751222 |
| 100150093_TGI_at | *LECT2* | 6.71E–22 | –1.32921358 |
| 100146716_TGI_at | *LIFR* | 1.49E–116 | –3.41680424 |
| 100142478_TGI_at | *LIFR* | 2.5E–147 | –2.94668562 |
| 100152174_TGI_at | *LIFR* | 7.71E–134 | –2.87125357 |
| 100121651_TGI_at | *LILRA1* | 2.06E–48 | –0.74123598 |
| 100125503_TGI_at | *LILRA5* | 1.7E–67 | –1.35257776 |
| 100155006_TGI_at | *LILRB1* | 1.65E–55 | –1.4795776 |
| 100155898_TGI_at | *LILRB2* | 2.08E–41 | –1.06905479 |
| 100158588_TGI_at | *LILRB5* | 4.7E–102 | –1.91954184 |
| 100137427_TGI_at | *LMNA* | 2.68E–17 | 0.3895784 |
| 100132630_TGI_at | *LMNA* | 1.15E–42 | 0.67159968 |
| 100139295_TGI_at | *LTA4H* | 1.01E–24 | 0.40803777 |
| 100125377_TGI_at | *MAL* | 6.81E–22 | –0.87982835 |
| 100157160_TGI_at | *MAP3K7* | 4.92E–12 | 0.23201211 |
| 100159769_TGI_at | *MAP3K7* | 1.86E–15 | 0.38940893 |
| 100131265_TGI_at | *MAPK1* | 1E–25 | 0.32194249 |
| 100143930_TGI_at | *MAPK1* | 1.79E–27 | 0.63722955 |
| 100153577_TGI_at | *MAPK1* | 1.13E–43 | 0.69077103 |
| 100125092_TGI_at | *MAPK14* | 1.11E–22 | 0.3183158 |
| 100144224_TGI_at | *MAPK3* | 6.32E–33 | 0.54965588 |
| 100157523_TGI_at | *MAPK9* | 2.24E–14 | 0.27526993 |
| 100134582_TGI_at | *MAPK9* | 3.22E–38 | 0.63919013 |
| 100136295_TGI_at | *MAPKAPK5* | 2.24E–17 | 0.23888727 |
| 100124563_TGI_at | *MARCO* | 2.34E–180 | –5.11955392 |
| 100156012_TGI_at | *MASP1* | 6.54E–123 | –2.09762967 |
| 100158033_TGI_at | *MASP1* | 4.61E–25 | –1.18948095 |
| 100122471_TGI_at | *MASP1* | 1.89E–24 | –0.83494412 |
| 100159675_TGI_at | *MASP2* | 1.21E–44 | –2.21935599 |
| 100131988_TGI_at | *MASP2* | 2.03E–22 | –1.21890857 |
| 100138059_TGI_at | *MAVS* | 5.62E–44 | 0.78320302 |
| 100154805_TGI_at | *MAVS* | 1.23E–44 | 0.90876546 |
| 100121792_TGI_at | *MAVS* | 7.14E–58 | 1.09248304 |
| 100130781_TGI_at | *MBL2* | 5.93E–38 | –1.57710984 |
| 100131485_TGI_at | *MED1* | 1.71E–16 | 0.30254933 |
| 100131086_TGI_at | *MED1* | 1.63E–29 | 0.49225795 |
| 100128963_TGI_at | *MEF2D* | 1.33E–13 | 0.32556128 |
| 100137745_TGI_at | *MEF2D* | 4.92E–41 | 0.74412922 |
| 100155945_TGI_at | *MEFV* | 2.33E–36 | –0.6604992 |
| 100161520_TGI_at | *MICB* | 5.64E–21 | 0.73986031 |
| 100154288_TGI_at | *NCOA2* | 5.68E–08 | 0.47882409 |
| 100160726_TGI_at | *NFIL3* | 3.77E–49 | –0.823141 |
| 100127622_TGI_at | *NFRKB* | 9.37E–20 | 0.32214213 |
| 100148170_TGI_at | *NFRKB* | 1.16E–41 | 0.4662169 |
| 100143806_TGI_at | *NLRC4* | 3.66E–44 | –0.96712418 |
| 100137352_TGI_at | *NLRP3* | 6.05E–26 | –0.67707183 |
| 100150559_TGI_at | *NR4A1* | 5.26E–49 | –1.43656401 |
| 100149159_TGI_at | *NRAS* | 3.52E–34 | 0.42640192 |
| 100145889_TGI_at | *NRAS* | 2E–28 | 0.62511936 |
| 100149649_TGI_at | *PAK1* | 5.5E–16 | 0.51106753 |
| 100138490_TGI_at | *PAK1* | 4.97E–23 | 0.57453764 |
| 100161390_TGI_at | *PARP1* | 1.11E–23 | 0.3802835 |
| 100145644_TGI_at | *PARP1* | 4.44E–24 | 0.67332366 |
| 100127502_TGI_at | *PDE1A* | 3.47E–45 | –1.57267332 |
| 100154543_TGI_at | *PDE1A* | 9.75E–26 | –1.1745309 |
| 100147156_TGI_at | *PDE1A* | 2.64E–21 | –0.83796001 |
| 100144527_TGI_at | *PDE2A* | 7.24E–82 | –1.16016281 |
| 100152051_TGI_at | *PDGFRA* | 1.07E–68 | –3.24795813 |
| 100160502_TGI_at | *PDGFRA* | 4.59E–41 | –1.8113469 |
| 100132209_TGI_at | *PDPK1* | 3.49E–26 | 0.45034449 |
| 100128294_TGI_at | *PDPK1* | 5.58E–25 | 0.48977591 |
| 100145146_TGI_at | *PGLYRP2* | 2.35E–11 | –0.83386353 |
| 100145838_TGI_at | *PIK3CA* | 2.16E–11 | 0.32496263 |
| 100140555_TGI_at | *PIK3CB* | 3.6E–16 | 0.22741089 |
| 100150856_TGI_at | *PIK3CB* | 6.79E–14 | 0.378554 |
| 100146093_TGI_at | *PLCB1* | 9.38E–41 | 0.79465067 |
| 100150289_TGI_at | *PLCB1* | 2.51E–38 | 1.1239266 |
| 100162486_TGI_at | *PLCB1* | 6.1E–43 | 1.38465406 |
| 100128098_TGI_at | *PLCB1* | 8.3E–46 | 1.46240006 |
| 100122823_TGI_at | *PLCB2* | 2.26E–35 | –0.61228233 |
| 100141576_TGI_at | *PPP1CC* | 2.79E–51 | 0.38858922 |
| 100136039_TGI_at | *PPP2R1A* | 1.34E–17 | 0.31187492 |
| 100137794_TGI_at | *PRKAR2A* | 1.32E–61 | 0.52620824 |
| 100148166_TGI_at | *PRKAR2A* | 4.91E–33 | 0.72043642 |
| 100125255_TGI_at | *PRKAR2B* | 1.06E–117 | –2.74686352 |
| 100129911_TGI_at | *PRKCB* | 2.07E–41 | –1.32726415 |
| 100149531_TGI_at | *PRKCB* | 2.42E–33 | –0.58175848 |
| 100152894_TGI_at | *PRKCB* | 4.95E–25 | –0.56336465 |
| 100157582_TGI_at | *PRKCB* | 1.76E–34 | –0.545834 |
| 100122144_TGI_at | *PSMA1* | 2.55E–13 | 0.16515192 |
| 100151268_TGI_at | *PSMB5* | 2.44E–18 | 0.31564807 |
| 100133016_TGI_at | *PTGDR* | 1.65E–30 | –1.12681131 |
| 100160363_TGI_at | *PTGIR* | 3.25E–70 | –1.73836465 |
| 100143106_TGI_at | *PTGIS* | 2.49E–56 | –2.76298593 |
| 100154148_TGI_at | *PTK2* | 1.1E–50 | 0.47422921 |
| 100128190_TGI_at | *PTPN13* | 1.45E–90 | –2.22276017 |
| 100136859_TGI_at | *PTPN13* | 1.99E–35 | –0.61763999 |
| 100153468_TGI_at | *RAF1* | 1.91E–12 | 0.39360525 |
| 100135278_TGI_at | *RASA1* | 6.04E–08 | 0.1849287 |
| 100150077_TGI_at | *RHEB* | 7.1E–39 | 0.62469928 |
| 100138300_TGI_at | *RHOA* | 2.69E–22 | 0.14904314 |
| 100124154_TGI_at | *RIPK2* | 4.62E–08 | 0.32948826 |
| 100159613_TGI_at | *ROCK2* | 1.62E–13 | 0.30506387 |
| 100142098_TGI_at | *RPS6KB1* | 2.11E–08 | 0.20856841 |
| 100136388_TGI_at | *S100A12* | 1.57E–35 | –1.80857387 |
| 100141181_TGI_at | *S100A8* | 3E–30 | –1.7424117 |
| 100138458_TGI_at | *SCARF1* | 2.54E–31 | –0.61233884 |
| 100151817_TGI_at | *SDPR* | 6.77E–45 | –1.46261115 |
| 100139289_TGI_at | *SELP* | 2.1E–68 | –2.25702249 |
| 100156679_TGI_at | *SEMA4D* | 4.09E–28 | –0.99286075 |
| 100135219_TGI_at | *SERPING1* | 5.37E–30 | –0.38554385 |
| 100157444_TGI_at | *SH2D1B* | 2.17E–26 | –0.70043336 |
| 100125508_TGI_at | *SHC1* | 1.62E–51 | 0.54171817 |
| 100125136_TGI_at | *SHC1* | 3.72E–25 | 0.55194977 |
| 100123369_TGI_at | *SIGLEC1* | 3.51E–59 | –1.54598141 |
| 100141684_TGI_at | *SIGLEC7* | 2.08E–72 | –1.70323684 |
| 100149346_TGI_at | *SOCS2* | 6.61E–78 | –2.13035101 |
| 100143469_TGI_at | *SOCS3* | 1.96E–49 | –1.39216773 |
| 100154644_TGI_at | *SOCS4* | 2.9E–11 | 0.28276416 |
| 100137092_TGI_at | *SOCS5* | 1.22E–23 | 0.40481582 |
| 100162163_TGI_at | *SOCS5* | 7.01E–46 | 0.7969193 |
| 100131422_TGI_at | *SOS1* | 7.17E–26 | 0.49430914 |
| 100143052_TGI_at | *SOS1* | 1.19E–40 | 0.49807314 |
| 100138604_TGI_at | *SPTAN1* | 1.24E–31 | 0.41983683 |
| 100122405_TGI_at | *STAT4* | 1.84E–21 | –0.88123596 |
| 100159453_TGI_at | *TBX21* | 4.68E–29 | –0.87093467 |
| 100160987_TGI_at | *TBXA2R* | 1.85E–107 | –2.36507986 |
| 100132994_TGI_at | *TBXAS1* | 5.85E–36 | –1.47661414 |
| 100145541_TGI_at | *TBXAS1* | 5.4E–29 | –1.2306796 |
| 100153982_TGI_at | *TGFBR1* | 4.3E–11 | 0.31091463 |
| 100122843_TGI_at | *TGFBR1* | 2.32E–16 | 0.46103349 |
| 100140535_TGI_at | *THEM4* | 1.57E–23 | 0.68704924 |
| 100155966_TGI_at | *THY1* | 6.08E–45 | 1.59064424 |
| 100125837_TGI_at | *TICAM2* | 0.000000268 | 0.28766052 |
| 100137302_TGI_at | *TLR4* | 4.45E–73 | –1.48746839 |
| 100139578_TGI_at | *TLR4* | 2.09E–56 | –1.11004092 |
| 100128797_TGI_at | *TLR4* | 5.56E–56 | –0.96166407 |
| 100153099_TGI_at | *TMEM189* | 1.2E–17 | 0.34374541 |
| 100161061_TGI_at | *TNFRSF10C* | 9.17E–23 | –0.39540932 |
| 100145006_TGI_at | *TNFRSF10C* | 1.42E–19 | –0.33054077 |
| 100130857_TGI_at | *TNFRSF10D* | 5.17E–43 | –1.01012772 |
| 100142204_TGI_at | *TNFSF12* | 2.34E–14 | –0.47873217 |
| 100132298_TGI_at | *TRAF2* | 7.38E–36 | 0.61928207 |
| 100154782_TGI_at | *TSC1* | 1.24E–28 | 0.65871557 |
| 100137837_TGI_at | *TXK* | 4.34E–37 | –0.97156357 |
| 100159536_TGI_at | *VTCN1* | 3.05E–65 | –2.07553747 |
| 100162324_TGI_at | *VTCN1* | 1.28E–24 | –0.55378835 |
| 100147121_TGI_at | *YWHAB* | 0.000000874 | 0.2599037 |
| 100124465_TGI_at | *YWHAG* | 8.45E–12 | 0.11605447 |
| 100148978_TGI_at | *YWHAH* | 3.77E–08 | 0.17675571 |
| 100134155_TGI_at | *YWHAZ* | 1.1E–09 | 0.23927195 |

*Transcript ID in GSE25097 dataset.

†Student's *t*-test. Bonferroni adjustment was used to correct for multiple comparisons, and *P*<1.33×10^-6^ was considered to be statistically significant in view of the 37,582 transcripts in GSE25097 dataset.

Abbreviations: FC, log_2_ (Fold changes), HCCs vs. adjacent non-tumor tissues.
