# Supplementary material for: Relatively Small Contribution of Methylation and Genomic Copy Number Aberration to the Aberrant Expression of Inflammation-Related Genes in HBV-Related Hepatocellular Carcinoma
Source: PLoS One. 2015 May 12;10(5):e0126836. doi: 10.1371/journal.pone.0126836 (PMC4429029; doi:10.1371/journal.pone.0126836)
Supplement: S10 Table — (DOC) [file pone.0126836.s012.doc]

**S10 Table. 110 Aberrantly Methylated Inflammation-related Genes Validated in GSE54503 Dataset**

| **ID*** | **Symbol** | **P Value**† | **FC** |
| --- | --- | --- | --- |
| cg09579061 | *ADORA1* | 6.52E–14 | –0.2 |
| cg08644365 | *ADORA1* | 1.72E–10 | –0.134 |
| cg05160197 | *ADORA1* | 1.24E–15 | –0.13 |
| cg14544831 | *BCL2* | 1.47E–10 | 0.152 |
| cg24740531 | *BCL2* | 6.91E–12 | 0.213 |
| cg14889643 | *BCL2* | 1.69E–14 | 0.226 |
| cg17602451 | *BCL2* | 7.22E–17 | 0.23 |
| cg21602520 | *BCL2* | 1.34E–21 | 0.243 |
| cg09229893 | *BMP4* | 5.52E–08 | 0.116 |
| cg00883329 | *BMP4* | 3.67E–13 | 0.187 |
| cg20297199 | *BMP4* | 8.29E–21 | 0.283 |
| cg14310034 | *BMP4* | 1.38E–29 | 0.341 |
| cg08162372 | *BMP4* | 4.77E–29 | 0.426 |
| cg15494458 | *BPI* | 7.62E–21 | –0.221 |
| cg18493415 | *BPI* | 3.67E–15 | –0.199 |
| cg08254089 | *BPI* | 1.03E–17 | –0.181 |
| cg24229276 | *BPI* | 2.17E–15 | –0.137 |
| cg08355317 | *BTK* | 4.85E–11 | –0.156 |
| cg15847896 | *BTK* | 4.58E–08 | –0.141 |
| cg13168221 | *C1QA* | 4.06E–20 | –0.255 |
| cg24677002 | *C1QA* | 1.12E–10 | –0.215 |
| cg07109332 | *C1QA* | 3.53E–13 | –0.162 |
| cg20676350 | *C1QA* | 1.87E–17 | –0.158 |
| cg08710757 | *C1QA* | 4.62E–09 | –0.143 |
| cg20556988 | *CCL1* | 1.21E–15 | –0.255 |
| cg17118262 | *CCL1* | 9.39E–14 | –0.23 |
| cg22186223 | *CCL1* | 3.37E–16 | –0.213 |
| cg12627751 | *CCL1* | 1.25E–10 | –0.169 |
| cg01387036 | *CCL1* | 2.75E–13 | –0.168 |
| cg00036723 | *CCL1* | 6.88E–20 | –0.146 |
| cg26101277 | *CCL1* | 7.95E–15 | –0.139 |
| cg05281206 | *CCL13* | 4.71E–22 | –0.287 |
| cg02706575 | *CCL13* | 2.25E–15 | –0.232 |
| cg24615251 | *CCL13* | 7.81E–14 | –0.223 |
| cg10042846 | *CCL13* | 5.55E–17 | –0.205 |
| cg21042139 | *CCL13* | 2.35E–12 | –0.162 |
| cg01566155 | *CCL13* | 6.47E–14 | –0.138 |
| cg21643045 | *CCL20* | 1.8E–25 | –0.263 |
| cg09425228 | *CCL20* | 3.68E–10 | –0.136 |
| cg08575688 | *CCL20* | 1.55E–14 | –0.123 |
| cg01520122 | *CCL7* | 5.35E–22 | –0.311 |
| cg08124722 | *CCL7* | 2.14E–22 | –0.303 |
| cg02936263 | *CCL7* | 3.64E–21 | –0.289 |
| cg11313065 | *CCR2* | 1.73E–18 | –0.264 |
| cg06280059 | *CCR2* | 8.53E–17 | –0.211 |
| cg04110105 | *CCR2* | 4.18E–17 | –0.164 |
| cg05266321 | *CCR2* | 8.29E–08 | –0.158 |
| cg03928384 | *CCR2* | 1.26E–08 | –0.142 |
| cg07743747 | *CCR2* | 4.69E–12 | –0.141 |
| cg09487078 | *CCR6* | 5.66E–20 | –0.192 |
| cg14391016 | *CCR6* | 9.12E–14 | –0.192 |
| cg05540913 | *CCR6* | 4.12E–11 | –0.173 |
| cg03533561 | *CCR6* | 1.46E–12 | –0.168 |
| cg02335141 | *CCR6* | 1.01E–08 | –0.149 |
| cg27303699 | *CD1A* | 8.38E–28 | –0.374 |
| cg03528325 | *CD1A* | 2.21E–32 | –0.324 |
| cg19355763 | *CD1A* | 1.29E–20 | –0.302 |
| cg07950803 | *CD1A* | 1.66E–25 | –0.27 |
| cg26946836 | *CD207* | 1.41E–14 | –0.246 |
| cg08989942 | *CD207* | 5.72E–21 | –0.243 |
| cg03976366 | *CD207* | 1.82E–16 | –0.209 |
| cg03895348 | *CD207* | 2.02E–19 | –0.207 |
| cg21108412 | *CD247* | 3.28E–12 | –0.195 |
| cg19762811 | *CD247* | 3.14E–15 | –0.192 |
| cg00583147 | *CD247* | 8.68E–14 | –0.184 |
| cg24929556 | *CD247* | 4.15E–11 | –0.152 |
| cg23114435 | *CD247* | 4.34E–13 | –0.148 |
| cg06531517 | *CD247* | 1.52E–09 | –0.131 |
| cg09179987 | *CD247* | 2.99E–08 | –0.122 |
| cg22356347 | *CD247* | 1.81E–10 | –0.12 |
| cg01833122 | *CD247* | 2.98E–08 | –0.0831 |
| cg24809544 | *CD33* | 9.91E–24 | –0.265 |
| cg10129493 | *CD33* | 3.9E–23 | –0.23 |
| cg11581627 | *CD33* | 1.99E–29 | –0.2 |
| cg11122968 | *CD33* | 2.61E–21 | –0.2 |
| cg06861672 | *CD33* | 1.46E–20 | –0.183 |
| cg27642618 | *CD33* | 1.19E–19 | –0.147 |
| cg24462702 | *CD40LG* | 2.55E–18 | –0.312 |
| cg11944101 | *CD40LG* | 8.94E–20 | –0.278 |
| cg00292305 | *CD40LG* | 1.24E–21 | –0.261 |
| cg23907260 | *CD40LG* | 8.89E–18 | –0.255 |
| cg14232368 | *CD40LG* | 1E–21 | –0.254 |
| cg02992767 | *CD40LG* | 6.42E–21 | –0.244 |
| cg21302055 | *CD40LG* | 8.29E–17 | –0.244 |
| cg09226411 | *CD40LG* | 6.33E–22 | –0.231 |
| cg07919695 | *CD40LG* | 1.69E–16 | –0.227 |
| cg27207932 | *CD40LG* | 8.27E–10 | –0.177 |
| cg00102714 | *CD46* | 4.58E–09 | 0.0462 |
| cg13794529 | *CD46* | 9.09E–21 | 0.0872 |
| cg02152968 | *CD55* | 2.88E–11 | 0.141 |
| cg00797651 | *CD55* | 2.53E–14 | 0.181 |
| cg05163496 | *CD8A* | 3.91E–11 | 0.109 |
| cg19410791 | *CD8A* | 1.84E–09 | 0.118 |
| cg07152196 | *CD8A* | 7.84E–14 | 0.122 |
| cg02170525 | *CD8A* | 6.53E–12 | 0.137 |
| cg12653796 | *CD8A* | 1.37E–15 | 0.143 |
| cg00916536 | *CD8A* | 2.22E–12 | 0.151 |
| cg06804210 | *CD8A* | 8.08E–11 | 0.154 |
| cg13803976 | *CD8A* | 2.94E–14 | 0.166 |
| cg13946520 | *CD8A* | 3.61E–10 | 0.166 |
| cg17108819 | *CD8A* | 1.19E–14 | 0.172 |
| cg27247697 | *CD8A* | 3.37E–16 | 0.185 |
| cg27502457 | *CD8A* | 3.16E–19 | 0.206 |
| cg26057751 | *CD8A* | 3.16E–19 | 0.21 |
| cg12606911 | *CD8A* | 3.25E–22 | 0.323 |
| cg17826043 | *CEACAM3* | 7.17E–19 | –0.282 |
| cg11310087 | *CEACAM3* | 8.2E–14 | –0.19 |
| cg13322449 | *CEACAM3* | 1.15E–10 | –0.149 |
| cg23181133 | *CEACAM3* | 6.03E–13 | –0.129 |
| cg19399653 | *CEACAM8* | 1.71E–17 | –0.189 |
| cg11654011 | *CEACAM8* | 4.67E–10 | –0.154 |
| cg14779825 | *CEACAM8* | 5.18E–16 | –0.149 |
| cg06475327 | *CEACAM8* | 2.12E–08 | –0.114 |
| cg27389562 | *CEACAM8* | 3.1E–14 | –0.0818 |
| cg08551633 | *CEACAM8* | 1.98E–12 | –0.0748 |
| cg05825950 | *CR1* | 8.25E–09 | 0.0642 |
| cg05922028 | *CR1* | 2.56E–09 | 0.104 |
| cg17147317 | *CR1* | 3.4E–09 | 0.12 |
| cg18147366 | *CR1* | 1.25E–11 | 0.127 |
| cg00175709 | *CR1* | 5.02E–13 | 0.147 |
| cg14726637 | *CR1* | 9.55E–12 | 0.149 |
| cg02588107 | *CR1* | 3.28E–14 | 0.167 |
| cg23510026 | *CXCL14* | 1.97E–11 | –0.194 |
| cg17008288 | *CXCL14* | 4.46E–11 | –0.194 |
| cg22891674 | *CXCL14* | 3.13E–11 | –0.163 |
| cg26525592 | *CXCL14* | 4.95E–10 | –0.151 |
| cg13048967 | *CXCR1* | 6.62E–22 | –0.3 |
| cg09294937 | *CXCR1* | 6.21E–21 | –0.264 |
| cg14702787 | *CXCR1* | 1.07E–16 | –0.252 |
| cg06683602 | *CXCR1* | 8.23E–15 | –0.245 |
| cg15768138 | *CXCR1* | 5.01E–20 | –0.241 |
| cg15908708 | *CXCR1* | 1.16E–15 | –0.209 |
| cg13519373 | *CXCR1* | 6.93E–15 | –0.208 |
| cg00832199 | *CXCR1* | 9.72E–18 | –0.202 |
| cg09905973 | *CXCR1* | 5.35E–15 | –0.166 |
| cg18956547 | *CXCR1* | 8.07E–10 | –0.142 |
| cg21004129 | *CXCR1* | 5.08E–09 | –0.139 |
| cg14652717 | *CXCR2* | 3.47E–21 | –0.304 |
| cg03464560 | *CXCR2* | 2.31E–15 | –0.248 |
| cg02937802 | *CXCR2* | 5.82E–16 | –0.242 |
| cg14150666 | *CXCR2* | 1.25E–17 | –0.235 |
| cg17081998 | *CXCR2* | 1.1E–16 | –0.228 |
| cg06547715 | *CXCR2* | 5.18E–19 | –0.213 |
| cg10819992 | *CXCR2* | 1.25E–15 | –0.184 |
| cg25941354 | *CXCR2* | 7.76E–18 | –0.17 |
| cg10591797 | *CXCR2* | 2.91E–10 | –0.145 |
| cg15657330 | *CXCR2* | 1.13E–14 | –0.142 |
| cg13739417 | *CXCR2* | 1.06E–09 | –0.139 |
| cg19225688 | *CXCR2* | 7.58E–09 | –0.106 |
| cg06038701 | *CYSLTR2* | 6.63E–14 | –0.195 |
| cg06322064 | *CYSLTR2* | 5.47E–10 | –0.167 |
| cg16299590 | *CYSLTR2* | 4.75E–09 | –0.154 |
| cg18236297 | *CYSLTR2* | 5.28E–09 | –0.128 |
| cg02506353 | *DAP* | 4.24E–09 | 0.0553 |
| cg19633004 | *DAP* | 2.97E–09 | 0.074 |
| cg14662675 | *DAP* | 1.02E–12 | 0.1 |
| cg14739039 | *DAP* | 0.00000011 | 0.105 |
| cg07817783 | *DAP* | 5.9E–10 | 0.175 |
| cg11027354 | *DAP* | 6.16E–10 | 0.197 |
| cg10808936 | *DARC* | 1.79E–21 | –0.268 |
| cg23018689 | *DARC* | 2.13E–16 | –0.195 |
| cg20003179 | *DARC* | 1E–11 | –0.165 |
| cg23507131 | *DARC* | 2.23E–14 | –0.15 |
| cg07202479 | *DARC* | 7.37E–15 | –0.141 |
| cg19260329 | *DARC* | 9.65E–10 | –0.139 |
| cg18552413 | *DARC* | 1.06E–09 | –0.125 |
| cg27232482 | *DARC* | 1.62E–16 | –0.118 |
| cg19384032 | *DUSP4* | 6.61E–08 | 0.11 |
| cg05976753 | *DUSP4* | 4.75E–10 | 0.135 |
| cg13983578 | *DUSP4* | 0.000000112 | 0.141 |
| cg27130302 | *DUSP4* | 4.81E–08 | 0.142 |
| cg19687358 | *DUSP4* | 1.18E–10 | 0.151 |
| cg05418877 | *DUSP4* | 2.88E–11 | 0.163 |
| cg18070061 | *DUSP4* | 2.52E–12 | 0.213 |
| cg25565730 | *ESR1* | 9.08E–08 | 0.123 |
| cg23009221 | *ESR1* | 1.31E–08 | 0.141 |
| cg24900983 | *ESR1* | 3.11E–08 | 0.144 |
| cg05171584 | *ESR1* | 4.96E–11 | 0.158 |
| cg21950534 | *ESR1* | 8.89E–08 | 0.162 |
| cg21614759 | *ESR1* | 1.01E–08 | 0.172 |
| cg23165623 | *ESR1* | 0.000000023 | 0.172 |
| cg22839866 | *ESR1* | 4.31E–11 | 0.179 |
| cg11813455 | *ESR1* | 7.09E–10 | 0.182 |
| cg27316393 | *ESR1* | 2.36E–09 | 0.187 |
| cg19411146 | *ESR1* | 1.74E–08 | 0.191 |
| cg20627916 | *ESR1* | 1.05E–10 | 0.193 |
| cg07671949 | *ESR1* | 5.01E–10 | 0.196 |
| cg23164938 | *ESR1* | 9E–10 | 0.216 |
| cg23467008 | *ESR1* | 2.38E–14 | 0.239 |
| cg13612689 | *ESR1* | 2.17E–16 | 0.244 |
| cg04611395 | *FKBP4* | 5.06E–16 | 0.199 |
| cg03310242 | *FKBP4* | 5.63E–21 | 0.324 |
| cg12306057 | *FPR2* | 3.42E–29 | –0.314 |
| cg14203721 | *FPR2* | 4.18E–28 | –0.298 |
| cg16726204 | *FPR2* | 5.31E–26 | –0.298 |
| cg05280088 | *FPR2* | 2.67E–23 | –0.292 |
| cg02309029 | *FPR2* | 1.55E–24 | –0.251 |
| cg09499256 | *FPR2* | 2.35E–24 | –0.192 |
| cg04554195 | *FPR2* | 2.21E–20 | –0.162 |
| cg03195665 | *FPR2* | 2.01E–17 | –0.0842 |
| cg09512548 | *FYN* | 1.28E–09 | 0.115 |
| cg14683699 | *FYN* | 4.67E–09 | 0.142 |
| cg02115050 | *FYN* | 2.96E–10 | 0.171 |
| cg26846592 | *FYN* | 7.95E–13 | 0.185 |
| cg17100322 | *FYN* | 4.27E–11 | 0.189 |
| cg15615586 | *HLA-DQB2* | 1.24E–24 | –0.329 |
| cg21566383 | *HLA-DQB2* | 5.62E–24 | –0.305 |
| cg21038932 | *HLA-DQB2* | 1.18E–22 | –0.3 |
| cg21810004 | *HLA-DQB2* | 9.97E–18 | –0.295 |
| cg04345908 | *HLA-DQB2* | 3.04E–21 | –0.29 |
| cg16899306 | *HLA-DQB2* | 1.91E–20 | –0.278 |
| cg04322111 | *HLA-DQB2* | 1.27E–14 | –0.272 |
| cg17043222 | *HLA-DQB2* | 6.04E–22 | –0.268 |
| cg21332305 | *HLA-DQB2* | 1.09E–15 | –0.262 |
| cg23743845 | *HLA-DQB2* | 5.62E–19 | –0.258 |
| cg10298215 | *HLA-DQB2* | 1.42E–19 | –0.255 |
| cg11505404 | *HLA-DQB2* | 4.15E–25 | –0.254 |
| cg21013323 | *HLA-DQB2* | 1.15E–14 | –0.253 |
| cg12296550 | *HLA-DQB2* | 3.36E–18 | –0.245 |
| cg23831898 | *HLA-DQB2* | 5.76E–17 | –0.24 |
| cg16315262 | *HLA-DQB2* | 1.67E–18 | –0.239 |
| cg25327122 | *HLA-DQB2* | 3.25E–12 | –0.237 |
| cg24298476 | *HLA-DQB2* | 8.25E–14 | –0.233 |
| cg24892091 | *HLA-DQB2* | 2.33E–19 | –0.23 |
| cg08976995 | *HLA-DQB2* | 1.6E–15 | –0.221 |
| cg23418102 | *HLA-DQB2* | 3.47E–13 | –0.22 |
| cg20985082 | *HLA-DQB2* | 2.57E–14 | –0.213 |
| cg11530659 | *HLA-DQB2* | 1.54E–16 | –0.21 |
| cg22407511 | *HLA-DQB2* | 3.77E–17 | –0.207 |
| cg04090745 | *HLA-DQB2* | 1.4E–16 | –0.206 |
| cg10218546 | *HLA-DQB2* | 1.61E–12 | –0.204 |
| cg02964065 | *HLA-DQB2* | 3.34E–09 | –0.199 |
| cg17637045 | *HLA-DQB2* | 1.85E–11 | –0.198 |
| cg04418355 | *HLA-DQB2* | 1.11E–12 | –0.197 |
| cg17360552 | *HLA-DQB2* | 3.04E–12 | –0.197 |
| cg23785275 | *HLA-DQB2* | 6.56E–12 | –0.195 |
| cg24307229 | *HLA-DQB2* | 1.02E–10 | –0.179 |
| cg06423300 | *HLA-DQB2* | 6.6E–13 | –0.17 |
| cg05764570 | *HLA-DQB2* | 6.2E–11 | –0.169 |
| cg07739876 | *HLA-DQB2* | 2.66E–13 | –0.167 |
| cg20645912 | *HLA-DQB2* | 8.31E–12 | –0.163 |
| cg03926206 | *HLA-DQB2* | 7.85E–10 | –0.156 |
| cg17475271 | *HLA-DQB2* | 1.3E–14 | –0.138 |
| cg06499030 | *HLA-DQB2* | 2.01E–20 | –0.129 |
| cg19939773 | *HLA-DQB2* | 7.87E–15 | –0.105 |
| cg06457736 | *HRH1* | 1.06E–32 | –0.386 |
| cg23247337 | *HRH1* | 1.78E–20 | –0.282 |
| cg27530352 | *HRH1* | 8.58E–20 | –0.275 |
| cg08831077 | *HRH1* | 2.11E–22 | –0.266 |
| cg13593758 | *HRH1* | 8.64E–18 | –0.257 |
| cg12179658 | *HRH1* | 6.98E–24 | –0.239 |
| cg14837082 | *HRH1* | 1.4E–15 | –0.235 |
| cg02960016 | *HRH1* | 1.04E–16 | –0.228 |
| cg12974545 | *HRH1* | 5.95E–16 | –0.223 |
| cg02150536 | *HRH1* | 1.56E–14 | –0.223 |
| cg26864526 | *HRH1* | 4.71E–15 | –0.208 |
| cg12477452 | *HRH1* | 4.71E–13 | –0.208 |
| cg27230009 | *HRH1* | 1.88E–15 | –0.207 |
| cg16929739 | *HRH1* | 3.34E–14 | –0.204 |
| cg03737442 | *HRH1* | 2.84E–12 | –0.204 |
| cg05551003 | *HRH1* | 5.19E–21 | –0.2 |
| cg01331196 | *HRH1* | 3.99E–14 | –0.197 |
| cg11702866 | *HRH1* | 1.77E–14 | –0.194 |
| cg17660833 | *HRH1* | 8.41E–13 | –0.191 |
| cg14458903 | *HRH1* | 6.41E–12 | –0.191 |
| cg25677394 | *HRH1* | 5.65E–15 | –0.185 |
| cg24357890 | *HRH1* | 1.76E–12 | –0.177 |
| cg18070504 | *HRH1* | 1.1E–12 | –0.176 |
| cg13256912 | *HRH1* | 1.69E–14 | –0.17 |
| cg21566860 | *HRH1* | 8.27E–11 | –0.151 |
| cg24607283 | *HRH1* | 2.15E–12 | –0.145 |
| cg15469802 | *HRH1* | 3.1E–10 | –0.141 |
| cg27092248 | *HRH1* | 4.43E–08 | –0.117 |
| cg08039854 | *HRH1* | 8.09E–08 | –0.112 |
| cg09666573 | *HRH1* | 1.09E–09 | –0.0932 |
| cg24397241 | *HRH1* | 1.54E–09 | –0.0929 |
| cg14773365 | *HRH2* | 7.47E–25 | –0.308 |
| cg12449682 | *HRH2* | 1.61E–18 | –0.289 |
| cg06477056 | *HRH2* | 1.3E–22 | –0.283 |
| cg01296705 | *HRH2* | 1.39E–21 | –0.267 |
| cg25599573 | *HRH2* | 4.46E–18 | –0.265 |
| cg06857674 | *HRH2* | 1.04E–23 | –0.253 |
| cg05011238 | *HRH2* | 8.57E–27 | –0.252 |
| cg02389195 | *HRH2* | 3.8E–18 | –0.25 |
| cg03657040 | *HRH2* | 6.91E–19 | –0.245 |
| cg25568981 | *HRH2* | 2.41E–13 | –0.239 |
| cg15207662 | *HRH2* | 1.26E–18 | –0.236 |
| cg17200850 | *HRH2* | 9.18E–14 | –0.233 |
| cg05919907 | *HRH2* | 1.38E–25 | –0.229 |
| cg14345676 | *HRH2* | 2.92E–14 | –0.226 |
| cg20277670 | *HRH2* | 1.87E–20 | –0.223 |
| cg22578632 | *HRH2* | 8.21E–15 | –0.22 |
| cg02765962 | *HRH2* | 3.17E–14 | –0.21 |
| cg01281175 | *HRH2* | 1E–11 | –0.193 |
| cg17578539 | *HRH2* | 6.71E–16 | –0.188 |
| cg18371750 | *HRH2* | 8.62E–12 | –0.174 |
| cg22806527 | *HRH2* | 3.57E–08 | –0.142 |
| cg17405853 | *HRH2* | 2.72E–08 | –0.141 |
| cg01940810 | *IFNG* | 1.06E–18 | –0.256 |
| cg00848007 | *IFNG* | 2.33E–18 | –0.241 |
| cg01281450 | *IFNG* | 2.82E–20 | –0.225 |
| cg05224770 | *IFNG* | 1.08E–16 | –0.211 |
| cg20890145 | *IFNG* | 1.95E–15 | –0.206 |
| cg12640631 | *IFNG* | 6.28E–13 | –0.196 |
| cg26227465 | *IFNG* | 3.08E–14 | –0.179 |
| cg11242303 | *IL12RB2* | 1.17E–09 | 0.114 |
| cg03975876 | *IL12RB2* | 2.43E–10 | 0.122 |
| cg15991072 | *IL12RB2* | 2.56E–12 | 0.16 |
| cg00993824 | *IL12RB2* | 6.43E–14 | 0.177 |
| cg04311496 | *IL12RB2* | 7.13E–14 | 0.177 |
| cg27416067 | *IL12RB2* | 2.79E–21 | 0.264 |
| cg20803547 | *IL12RB2* | 5.54E–20 | 0.32 |
| cg18139692 | *IL13RA2* | 6.55E–18 | –0.273 |
| cg00488364 | *IL13RA2* | 6.8E–16 | –0.229 |
| cg03244736 | *IL13RA2* | 8.13E–09 | –0.167 |
| cg02998206 | *IL13RA2* | 8.65E–08 | –0.138 |
| cg00423014 | *IL13RA2* | 1.17E–10 | –0.0748 |
| cg11924517 | *IL17A* | 2.13E–19 | –0.258 |
| cg27168844 | *IL17A* | 1.15E–18 | –0.243 |
| cg10492152 | *IL17A* | 4.37E–15 | –0.235 |
| cg09582880 | *IL17A* | 4.32E–12 | –0.208 |
| cg07376932 | *IL17A* | 3.74E–12 | –0.174 |
| cg05884768 | *IL17A* | 4.07E–08 | –0.13 |
| cg27361520 | *IL18RAP* | 1.08E–24 | –0.323 |
| cg13897122 | *IL18RAP* | 5.79E–19 | –0.252 |
| cg21282997 | *IL18RAP* | 2.04E–28 | –0.239 |
| cg11335172 | *IL18RAP* | 4.94E–13 | –0.197 |
| cg26675329 | *IL18RAP* | 1.86E–14 | –0.195 |
| cg16386158 | *IL1RL1* | 1.88E–18 | –0.286 |
| cg12076546 | *IL1RL1* | 2.03E–16 | –0.271 |
| cg17738684 | *IL1RL1* | 5.07E–17 | –0.247 |
| cg11916609 | *IL1RL1* | 9.38E–19 | –0.243 |
| cg20060108 | *IL1RL1* | 7.69E–19 | –0.238 |
| cg01254707 | *IL1RL1* | 1.18E–13 | –0.202 |
| cg02983090 | *IL21R* | 2.25E–21 | –0.224 |
| cg10416668 | *IL21R* | 6.75E–21 | –0.206 |
| cg08282819 | *IL21R* | 1.48E–21 | –0.201 |
| cg02656594 | *IL21R* | 1.3E–14 | –0.188 |
| cg00050618 | *IL21R* | 4.38E–11 | –0.174 |
| cg10454258 | *IL21R* | 3.51E–17 | –0.171 |
| cg05814654 | *IL21R* | 2.25E–14 | –0.145 |
| cg26505691 | *IL21R* | 1.29E–09 | –0.143 |
| cg03145200 | *IL21R* | 5.73E–09 | –0.138 |
| cg04059988 | *IL21R* | 4.49E–12 | –0.0457 |
| cg14588682 | *IL22* | 4.39E–24 | –0.327 |
| cg11520493 | *IL22* | 2.34E–22 | –0.298 |
| cg26333641 | *IL22* | 2.43E–19 | –0.295 |
| cg27518047 | *IL22* | 6.38E–19 | –0.262 |
| cg27018240 | *IL22* | 8.42E–15 | –0.24 |
| cg13195526 | *IL22* | 1.28E–24 | –0.203 |
| cg03694077 | *IL22* | 6.07E–11 | –0.17 |
| cg02543402 | *IL22* | 2.29E–10 | –0.168 |
| cg13851647 | *IL22* | 1.19E–08 | –0.15 |
| cg14366598 | *IL25* | 4.74E–16 | –0.213 |
| cg16025120 | *IL25* | 6.55E–16 | –0.211 |
| cg23619217 | *IL25* | 2.96E–16 | –0.206 |
| cg07258507 | *IL25* | 3.9E–15 | –0.158 |
| cg10140158 | *IL6* | 5.08E–23 | –0.279 |
| cg07998387 | *IL6* | 1.1E–16 | –0.244 |
| cg13104385 | *IL6* | 5.69E–12 | –0.176 |
| cg17067544 | *IL6* | 4.19E–18 | –0.175 |
| cg05265849 | *IL6* | 4.32E–12 | –0.17 |
| cg10732560 | *INS* | 3.69E–16 | –0.265 |
| cg16711450 | *INS* | 3.65E–14 | –0.202 |
| cg23390871 | *INS* | 8.34E–15 | –0.193 |
| cg11322849 | *INS* | 5.32E–09 | –0.172 |
| cg25601886 | *INS* | 7.74E–14 | –0.164 |
| cg25969082 | *INS* | 1.05E–08 | –0.136 |
| cg18098639 | *INS* | 4.01E–09 | –0.132 |
| cg03366382 | *INS* | 5.46E–08 | –0.132 |
| cg09864961 | *INS* | 3.75E–09 | –0.125 |
| cg06294416 | *IRAK3* | 7.26E–16 | –0.204 |
| cg01194336 | *IRAK3* | 1.64E–12 | –0.168 |
| cg08660562 | *IRAK3* | 2.3E–11 | –0.168 |
| cg19894085 | *IRAK3* | 2.29E–08 | –0.145 |
| cg05996573 | *IRAK3* | 7.83E–08 | –0.0895 |
| cg17128892 | *IRF2* | 3.49E–09 | 0.13 |
| cg25140370 | *IRF4* | 5.34E–09 | 0.106 |
| cg26433102 | *IRF4* | 1.32E–18 | 0.195 |
| cg12612118 | *IRF4* | 1.18E–17 | 0.213 |
| cg12741420 | *IRF4* | 3.8E–21 | 0.222 |
| cg05766140 | *IRF4* | 2.66E–19 | 0.244 |
| cg17228900 | *IRF4* | 3.75E–18 | 0.253 |
| cg10379890 | *IRF4* | 3.43E–20 | 0.254 |
| cg01108118 | *IRF4* | 5.25E–20 | 0.269 |
| cg12684209 | *IRF4* | 1.94E–23 | 0.276 |
| cg21277995 | *IRF4* | 1.64E–18 | 0.286 |
| cg06223767 | *IRF4* | 1.65E–20 | 0.319 |
| cg06392169 | *IRF4* | 2.7E–27 | 0.376 |
| cg11947981 | *ITGA4* | 2.47E–10 | 0.126 |
| cg21995919 | *ITGA4* | 5.7E–09 | 0.147 |
| cg06952671 | *ITGA4* | 1.35E–09 | 0.162 |
| cg25024074 | *ITGA4* | 2.61E–12 | 0.181 |
| cg24997944 | *KCNH8* | 3.62E–08 | 0.104 |
| cg23011597 | *KCNH8* | 4.19E–09 | 0.133 |
| cg01398050 | *KCNH8* | 4.62E–11 | 0.156 |
| cg18500192 | *KCNH8* | 5.77E–10 | 0.161 |
| cg24668089 | *KCNH8* | 1.46E–11 | 0.163 |
| cg06494497 | *KIR2DS4* | 7.41E–30 | –0.324 |
| cg24865007 | *KIR2DS4* | 3.66E–20 | –0.29 |
| cg27162757 | *KIR2DS4* | 1.04E–22 | –0.268 |
| cg22556816 | *KIR2DS4* | 3.02E–29 | –0.231 |
| cg10195200 | *KIR2DS4* | 1.87E–11 | –0.104 |
| cg04586348 | *KIR3DL2* | 1.62E–31 | –0.307 |
| cg19554939 | *KIR3DL2* | 6.52E–34 | –0.287 |
| cg05720980 | *KIR3DL2* | 5.27E–20 | –0.241 |
| cg08510934 | *KIR3DL2* | 2.23E–16 | –0.21 |
| cg20176989 | *KIR3DL2* | 2.22E–08 | –0.171 |
| cg12304663 | *KIR3DL2* | 8.18E–14 | –0.161 |
| cg10680488 | *KIR3DL2* | 6.71E–16 | –0.132 |
| cg01532713 | *KIR3DL2* | 9.11E–10 | –0.102 |
| cg20652042 | *LAG3* | 6.22E–13 | 0.0669 |
| cg10191002 | *LAG3* | 7.32E–12 | 0.109 |
| cg19421125 | *LAG3* | 2.15E–14 | 0.155 |
| cg02695343 | *LAG3* | 3.67E–19 | 0.174 |
| cg16352928 | *LAG3* | 1.17E–19 | 0.185 |
| cg19872463 | *LAG3* | 2.04E–15 | 0.187 |
| cg17213699 | *LAG3* | 2.69E–16 | 0.192 |
| cg10500147 | *LAG3* | 4.62E–17 | 0.206 |
| cg21878746 | *LAIR1* | 2.21E–28 | –0.269 |
| cg10132694 | *LAIR1* | 2.32E–28 | –0.259 |
| cg01515802 | *LAIR1* | 3.55E–29 | –0.258 |
| cg20117742 | *LAIR1* | 1.39E–30 | –0.225 |
| cg21892720 | *LAIR1* | 8.55E–15 | –0.203 |
| cg22211917 | *LAIR1* | 3.36E–12 | –0.196 |
| cg23672828 | *LAIR1* | 9.3E–13 | –0.18 |
| cg24147838 | *LAIR1* | 3.37E–14 | –0.175 |
| cg22545168 | *LAIR1* | 1.9E–18 | –0.163 |
| cg25573710 | *LAIR1* | 8.33E–08 | –0.145 |
| cg17713010 | *LAIR1* | 4.41E–18 | –0.101 |
| cg21748244 | *LCP2* | 4E–24 | –0.307 |
| cg26104475 | *LCP2* | 4.17E–24 | –0.299 |
| cg26518932 | *LCP2* | 5.16E–21 | –0.277 |
| cg02676052 | *LCP2* | 1.5E–22 | –0.276 |
| cg04822621 | *LCP2* | 4.74E–21 | –0.202 |
| cg08670465 | *LCP2* | 1.02E–16 | –0.168 |
| cg11528914 | *LCP2* | 1.36E–08 | –0.16 |
| cg08748308 | *LCP2* | 5.03E–12 | –0.145 |
| cg05265596 | *LCP2* | 1.17E–08 | –0.136 |
| cg09451413 | *LCP2* | 6.03E–08 | –0.126 |
| cg17752270 | *LCP2* | 4.78E–12 | –0.0977 |
| cg04167833 | *LCP2* | 4.51E–14 | –0.0854 |
| cg14071925 | *LILRA1* | 1.07E–20 | –0.291 |
| cg25806685 | *LILRA1* | 1.43E–29 | –0.286 |
| cg22029476 | *LILRA1* | 4.14E–25 | –0.278 |
| cg25384595 | *LILRA1* | 2.19E–28 | –0.185 |
| cg15701210 | *LILRA1* | 2.14E–23 | –0.154 |
| cg23184503 | *LILRA1* | 1.21E–16 | –0.134 |
| cg14575739 | *LILRA2* | 6.85E–23 | –0.235 |
| cg07280593 | *LILRA2* | 1.37E–27 | –0.231 |
| cg19486673 | *LILRA2* | 2.69E–25 | –0.179 |
| cg16893868 | *LILRA2* | 1.04E–14 | –0.16 |
| cg15659599 | *LILRA2* | 3.33E–17 | –0.127 |
| cg19216475 | *LILRA2* | 2.46E–22 | –0.111 |
| cg03320607 | *LILRA3* | 2.03E–23 | –0.286 |
| cg15460135 | *LILRA3* | 8.55E–21 | –0.286 |
| cg20038038 | *LILRA3* | 1.3E–30 | –0.204 |
| cg00705255 | *LILRA3* | 1.24E–15 | –0.101 |
| cg24755177 | *LILRA4* | 1.67E–30 | –0.398 |
| cg23899409 | *LILRA4* | 2.82E–18 | –0.259 |
| cg01067405 | *LILRA4* | 2.58E–23 | –0.251 |
| cg03575041 | *LILRA4* | 1.19E–21 | –0.232 |
| cg01204985 | *LILRA4* | 9.35E–16 | –0.224 |
| cg26453295 | *LILRA4* | 2.92E–17 | –0.217 |
| cg19802477 | *LILRA4* | 6.63E–26 | –0.196 |
| cg20542190 | *LILRA4* | 2.36E–21 | –0.156 |
| cg02434051 | *LILRA4* | 3.95E–08 | –0.066 |
| cg05340438 | *LILRA5* | 2.05E–24 | –0.322 |
| cg21293500 | *LILRA5* | 7.11E–22 | –0.281 |
| cg00727947 | *LILRA5* | 2.8E–19 | –0.245 |
| cg15691140 | *LILRA5* | 1.39E–14 | –0.139 |
| cg26642960 | *LILRA6* | 1.13E–23 | –0.291 |
| cg19835973 | *LILRA6* | 5.05E–20 | –0.232 |
| cg22686939 | *LILRA6* | 3.99E–08 | –0.184 |
| cg10429096 | *LILRA6* | 1.08E–15 | –0.167 |
| cg13400332 | *LILRA6* | 5.98E–19 | –0.15 |
| cg00001510 | *LILRA6* | 3.92E–13 | –0.0995 |
| cg04753936 | *LILRB1* | 5.33E–26 | –0.379 |
| cg08386867 | *LILRB1* | 2.31E–26 | –0.316 |
| cg04730930 | *LILRB1* | 2.85E–31 | –0.287 |
| cg24154699 | *LILRB1* | 6.23E–23 | –0.209 |
| cg13762704 | *LILRB1* | 8.79E–30 | –0.196 |
| cg05365532 | *LILRB1* | 1.05E–23 | –0.161 |
| cg02340056 | *LILRB1* | 5.45E–11 | –0.13 |
| cg26778001 | *LILRB1* | 8.77E–15 | –0.113 |
| cg01720520 | *LILRB1* | 2.36E–11 | –0.0847 |
| cg26649140 | *LILRB1* | 0.000000111 | –0.0639 |
| cg02630143 | *LILRB1* | 6.99E–09 | –0.0585 |
| cg19279346 | *LILRB2* | 6.64E–25 | –0.307 |
| cg21955916 | *LILRB2* | 3.97E–24 | –0.268 |
| cg05248470 | *LILRB2* | 1.71E–21 | –0.222 |
| cg24260917 | *LILRB2* | 1.3E–20 | –0.191 |
| cg12485793 | *LILRB2* | 4.11E–12 | –0.0948 |
| cg04742671 | *LILRB2* | 2.78E–19 | –0.0663 |
| cg23679434 | *LILRB3* | 1.25E–24 | –0.232 |
| cg15816012 | *LILRB3* | 1.8E–12 | –0.143 |
| cg17295720 | *LILRB3* | 6.62E–11 | –0.0996 |
| cg15627251 | *LILRB3* | 3.21E–15 | –0.0977 |
| cg22299782 | *LILRB3* | 2.65E–14 | –0.0818 |
| cg02421734 | *LILRB4* | 1.83E–29 | –0.278 |
| cg05329879 | *LILRB4* | 1.65E–19 | –0.237 |
| cg05922591 | *LILRB4* | 1.09E–16 | –0.234 |
| cg09552641 | *LILRB4* | 2.06E–26 | –0.231 |
| cg00113020 | *LILRB4* | 1.5E–14 | –0.231 |
| cg16661609 | *LILRB4* | 7.1E–15 | –0.227 |
| cg25975071 | *LILRB4* | 1.88E–19 | –0.21 |
| cg22458693 | *LILRB4* | 4.72E–20 | –0.202 |
| cg12161905 | *LILRB4* | 5.9E–21 | –0.192 |
| cg24140775 | *LILRB4* | 5.57E–17 | –0.191 |
| cg04680738 | *LILRB4* | 8.64E–14 | –0.115 |
| cg13535489 | *LILRB5* | 1.41E–29 | –0.28 |
| cg14802684 | *LILRB5* | 1.91E–21 | –0.246 |
| cg15584497 | *LILRB5* | 8.01E–18 | –0.224 |
| cg08684473 | *LILRB5* | 1.65E–21 | –0.164 |
| cg20649991 | *LILRB5* | 1.56E–23 | –0.163 |
| cg03688987 | *LILRB5* | 7.02E–14 | –0.135 |
| cg12197557 | *LILRB5* | 1.12E–13 | –0.0902 |
| cg19845720 | *LILRP2* | 1.1E–26 | –0.333 |
| cg16012769 | *LILRP2* | 9.92E–27 | –0.323 |
| cg19273454 | *LILRP2* | 1.24E–19 | –0.251 |
| cg17759564 | *LILRP2* | 7.4E–20 | –0.243 |
| cg05135107 | *LILRP2* | 3.77E–18 | –0.185 |
| cg03372852 | *LILRP2* | 1.31E–08 | –0.0916 |
| cg17580616 | *MADD* | 9.64E–10 | 0.047 |
| cg03393444 | *MADD* | 2.28E–09 | 0.0884 |
| cg16826777 | *MAP3K14* | 1.29E–10 | 0.0666 |
| cg19601328 | *MAP3K14* | 3.69E–10 | 0.111 |
| cg22657351 | *MARCO* | 3.95E–20 | –0.264 |
| cg02431964 | *MARCO* | 3.46E–14 | –0.25 |
| cg07164606 | *MARCO* | 4.9E–15 | –0.248 |
| cg11009736 | *MARCO* | 4.03E–19 | –0.245 |
| cg09601514 | *MARCO* | 4.79E–17 | –0.214 |
| cg19284751 | *MARCO* | 1.12E–13 | –0.213 |
| cg13995101 | *MEF2C* | 5.59E–08 | 0.0829 |
| cg07742134 | *MEF2C* | 2.37E–10 | 0.0937 |
| cg08223748 | *MEF2C* | 4.54E–12 | 0.132 |
| cg16225168 | *MEF2C* | 3.72E–09 | 0.17 |
| cg13690989 | *MEF2C* | 3.66E–11 | 0.175 |
| cg25611476 | *MEF2C* | 2.22E–13 | 0.202 |
| cg04656101 | *MMP9* | 2.71E–08 | –0.119 |
| cg21451869 | *MMP9* | 3.99E–18 | –0.113 |
| cg02310296 | *MMP9* | 5.32E–15 | –0.11 |
| cg10505873 | *MMP9* | 1.13E–22 | –0.105 |
| cg23322851 | *MSR1* | 2.86E–18 | –0.252 |
| cg16536964 | *MSR1* | 8.83E–24 | –0.245 |
| cg16303562 | *MSR1* | 5.87E–16 | –0.224 |
| cg10836855 | *MSR1* | 5.19E–14 | –0.213 |
| cg21431387 | *MSR1* | 2.14E–14 | –0.21 |
| cg06623274 | *MSR1* | 1.17E–15 | –0.19 |
| cg07347534 | *MSR1* | 1.88E–11 | –0.166 |
| cg03055449 | *MUC1* | 7.42E–08 | 0.0968 |
| cg02367916 | *NCR1* | 2.25E–16 | –0.236 |
| cg11356156 | *NCR1* | 6.35E–16 | –0.207 |
| cg13518366 | *NCR1* | 1.86E–14 | –0.19 |
| cg18832619 | *NCR1* | 1.15E–18 | –0.177 |
| cg17804112 | *NCR1* | 9.14E–09 | –0.138 |
| cg12952132 | *NCR1* | 2.92E–08 | –0.0695 |
| cg16313382 | *NFATC1* | 6.91E–23 | –0.409 |
| cg02714192 | *NFATC1* | 3.49E–20 | –0.403 |
| cg20513080 | *NFATC1* | 6.27E–19 | –0.336 |
| cg05264214 | *NFATC1* | 2.28E–19 | –0.335 |
| cg07740306 | *NFATC1* | 1.42E–18 | –0.333 |
| cg20542619 | *NFATC1* | 2.09E–16 | –0.32 |
| cg17938607 | *NFATC1* | 3.61E–20 | –0.315 |
| cg02736255 | *NFATC1* | 1.73E–22 | –0.31 |
| cg03432598 | *NFATC1* | 8.79E–22 | –0.307 |
| cg05859578 | *NFATC1* | 4.53E–20 | –0.306 |
| cg16246200 | *NFATC1* | 1.26E–22 | –0.305 |
| cg25595641 | *NFATC1* | 2.61E–18 | –0.304 |
| cg18108009 | *NFATC1* | 4.29E–21 | –0.302 |
| cg18935516 | *NFATC1* | 2.58E–21 | –0.298 |
| cg27100149 | *NFATC1* | 3.02E–19 | –0.297 |
| cg14663983 | *NFATC1* | 4.86E–20 | –0.296 |
| cg02675550 | *NFATC1* | 3.81E–19 | –0.296 |
| cg00445548 | *NFATC1* | 2.08E–22 | –0.291 |
| cg04516518 | *NFATC1* | 5.74E–21 | –0.289 |
| cg00110171 | *NFATC1* | 6.05E–16 | –0.288 |
| cg06856570 | *NFATC1* | 2.55E–15 | –0.287 |
| cg05944967 | *NFATC1* | 7.39E–18 | –0.286 |
| cg17102627 | *NFATC1* | 6.92E–21 | –0.285 |
| cg15702661 | *NFATC1* | 3.79E–25 | –0.282 |
| cg02113385 | *NFATC1* | 3.31E–25 | –0.281 |
| cg15798890 | *NFATC1* | 1.87E–21 | –0.28 |
| cg18512446 | *NFATC1* | 1.63E–17 | –0.279 |
| cg02889418 | *NFATC1* | 1.91E–13 | –0.279 |
| cg12424293 | *NFATC1* | 1.06E–17 | –0.278 |
| cg05718035 | *NFATC1* | 1.48E–19 | –0.277 |
| cg25332265 | *NFATC1* | 2.18E–21 | –0.276 |
| cg22532194 | *NFATC1* | 1.64E–19 | –0.275 |
| cg15532942 | *NFATC1* | 1.54E–19 | –0.274 |
| cg07759042 | *NFATC1* | 2.61E–18 | –0.274 |
| cg08157446 | *NFATC1* | 7.56E–20 | –0.269 |
| cg03285823 | *NFATC1* | 2.85E–19 | –0.269 |
| cg27019645 | *NFATC1* | 6.99E–17 | –0.268 |
| cg12352896 | *NFATC1* | 7.96E–15 | –0.266 |
| cg07342752 | *NFATC1* | 4.06E–16 | –0.265 |
| cg18773522 | *NFATC1* | 2E–21 | –0.264 |
| cg02743589 | *NFATC1* | 4.15E–18 | –0.264 |
| cg06351295 | *NFATC1* | 2.5E–19 | –0.262 |
| cg02763290 | *NFATC1* | 1.82E–17 | –0.261 |
| cg17977133 | *NFATC1* | 5.43E–19 | –0.26 |
| cg05712931 | *NFATC1* | 8.01E–19 | –0.256 |
| cg02875487 | *NFATC1* | 1.7E–17 | –0.254 |
| cg05707833 | *NFATC1* | 1.62E–20 | –0.25 |
| cg03239925 | *NFATC1* | 3.53E–19 | –0.248 |
| cg27308982 | *NFATC1* | 1.08E–16 | –0.247 |
| cg27334938 | *NFATC1* | 7.79E–19 | –0.246 |
| cg07912144 | *NFATC1* | 1.1E–12 | –0.246 |
| cg11844358 | *NFATC1* | 3.17E–15 | –0.245 |
| cg18475143 | *NFATC1* | 7.55E–14 | –0.242 |
| cg14715383 | *NFATC1* | 1.92E–16 | –0.239 |
| cg07277828 | *NFATC1* | 6.51E–19 | –0.238 |
| cg26244832 | *NFATC1* | 1.3E–15 | –0.236 |
| cg23240477 | *NFATC1* | 8.38E–17 | –0.234 |
| cg13731636 | *NFATC1* | 1.76E–16 | –0.234 |
| cg24538512 | *NFATC1* | 3.32E–13 | –0.234 |
| cg26550337 | *NFATC1* | 2.37E–20 | –0.233 |
| cg16421653 | *NFATC1* | 1.69E–17 | –0.231 |
| cg17057218 | *NFATC1* | 1.34E–15 | –0.231 |
| cg05532093 | *NFATC1* | 5.6E–19 | –0.23 |
| cg13691622 | *NFATC1* | 2.37E–17 | –0.23 |
| cg13456321 | *NFATC1* | 2.53E–14 | –0.23 |
| cg06937978 | *NFATC1* | 2.86E–18 | –0.228 |
| cg24163668 | *NFATC1* | 7.89E–18 | –0.227 |
| cg24488344 | *NFATC1* | 3.75E–13 | –0.226 |
| cg16960291 | *NFATC1* | 7.97E–12 | –0.226 |
| cg26100137 | *NFATC1* | 5.98E–16 | –0.224 |
| cg16536399 | *NFATC1* | 2.35E–14 | –0.224 |
| cg12524531 | *NFATC1* | 2.77E–16 | –0.223 |
| cg15704408 | *NFATC1* | 4.08E–19 | –0.217 |
| cg21242663 | *NFATC1* | 9.54E–15 | –0.216 |
| cg18590092 | *NFATC1* | 6.26E–14 | –0.211 |
| cg12761626 | *NFATC1* | 8.4E–19 | –0.208 |
| cg21806238 | *NFATC1* | 3.68E–15 | –0.208 |
| cg06432200 | *NFATC1* | 1.21E–13 | –0.208 |
| cg13880617 | *NFATC1* | 1.29E–17 | –0.206 |
| cg06084244 | *NFATC1* | 1.84E–19 | –0.203 |
| cg13580107 | *NFATC1* | 1.92E–14 | –0.203 |
| cg26915704 | *NFATC1* | 4.06E–13 | –0.202 |
| cg06493806 | *NFATC1* | 9.59E–08 | –0.201 |
| cg05302701 | *NFATC1* | 3E–14 | –0.199 |
| cg16253249 | *NFATC1* | 2.69E–16 | –0.198 |
| cg11977716 | *NFATC1* | 4.72E–09 | –0.197 |
| cg18974966 | *NFATC1* | 1.25E–17 | –0.196 |
| cg02870946 | *NFATC1* | 5.7E–14 | –0.195 |
| cg11321921 | *NFATC1* | 1.87E–13 | –0.194 |
| cg16179938 | *NFATC1* | 4.56E–09 | –0.192 |
| cg00431336 | *NFATC1* | 2.23E–16 | –0.189 |
| cg16182267 | *NFATC1* | 6.9E–14 | –0.185 |
| cg07637243 | *NFATC1* | 6.22E–13 | –0.182 |
| cg13207250 | *NFATC1* | 2.37E–13 | –0.181 |
| cg16308790 | *NFATC1* | 5.49E–11 | –0.181 |
| cg00356361 | *NFATC1* | 2.28E–14 | –0.18 |
| cg15908877 | *NFATC1* | 8.73E–12 | –0.179 |
| cg00636427 | *NFATC1* | 4.2E–10 | –0.179 |
| cg22279865 | *NFATC1* | 2.02E–11 | –0.177 |
| cg00151768 | *NFATC1* | 6.25E–08 | –0.171 |
| cg18092363 | *NFATC1* | 1.3E–12 | –0.168 |
| cg16997486 | *NFATC1* | 2.9E–11 | –0.168 |
| cg18486850 | *NFATC1* | 1.85E–09 | –0.167 |
| cg26475087 | *NFATC1* | 1.9E–12 | –0.163 |
| cg21192698 | *NFATC1* | 7.78E–10 | –0.162 |
| cg23322122 | *NFATC1* | 3.11E–11 | –0.161 |
| cg16071091 | *NFATC1* | 6.34E–09 | –0.161 |
| cg00140494 | *NFATC1* | 2.4E–11 | –0.16 |
| cg01277438 | *NFATC1* | 1.15E–12 | –0.157 |
| cg01514831 | *NFATC1* | 2.58E–10 | –0.156 |
| cg03586564 | *NFATC1* | 4.41E–08 | –0.155 |
| cg15260951 | *NFATC1* | 0.000000032 | –0.154 |
| cg25546405 | *NFATC1* | 5.11E–08 | –0.131 |
| cg15517343 | *NFATC1* | 5.99E–08 | –0.13 |
| cg12798257 | *NFATC1* | 2.96E–09 | –0.129 |
| cg05753993 | *NFATC1* | 1.37E–10 | –0.126 |
| cg15138382 | *NFATC1* | 5.09E–10 | –0.125 |
| cg23439917 | *NFATC1* | 1.25E–09 | –0.121 |
| cg26910047 | *NFATC1* | 5.62E–09 | –0.121 |
| cg07952877 | *NFATC1* | 4.02E–08 | –0.115 |
| cg25235326 | *NFATC1* | 4.58E–13 | –0.108 |
| cg10357989 | *NFATC1* | 3.54E–08 | –0.107 |
| cg12759909 | *NFATC1* | 7.27E–18 | –0.102 |
| cg18793688 | *NLRP3* | 7.25E–25 | –0.381 |
| cg07164722 | *NLRP3* | 1.89E–30 | –0.354 |
| cg14413862 | *NLRP3* | 1.52E–25 | –0.342 |
| cg21991396 | *NLRP3* | 8.03E–27 | –0.335 |
| cg24639969 | *NLRP3* | 3.08E–31 | –0.319 |
| cg06710101 | *NLRP3* | 5E–29 | –0.313 |
| cg18183941 | *NLRP3* | 4.62E–16 | –0.251 |
| cg12280471 | *NLRP3* | 2.34E–26 | –0.227 |
| cg03466998 | *NLRP3* | 7.59E–19 | –0.213 |
| cg21919599 | *NLRP3* | 3.27E–27 | –0.208 |
| cg03505654 | *NLRP3* | 4.84E–20 | –0.183 |
| cg21806273 | *NLRP3* | 1.03E–19 | –0.169 |
| cg07313373 | *NLRP3* | 4.93E–26 | –0.163 |
| cg26112639 | *NLRP3* | 4.67E–13 | –0.131 |
| cg21824010 | *NLRP3* | 2.73E–12 | –0.124 |
| cg08690999 | *NR2F1* | 1.31E–08 | 0.0981 |
| cg05553502 | *NR2F1* | 1.08E–10 | 0.114 |
| cg15171839 | *NR2F1* | 3.65E–11 | 0.131 |
| cg03714110 | *NR2F1* | 6.18E–14 | 0.166 |
| cg06101180 | *NR4A2* | 3.34E–08 | 0.0677 |
| cg27074041 | *NR4A2* | 1.09E–09 | 0.0731 |
| cg21226516 | *NR4A2* | 1.31E–09 | 0.0739 |
| cg00194126 | *NR4A2* | 5.97E–09 | 0.119 |
| cg11358945 | *NR4A2* | 4.62E–14 | 0.138 |
| cg11638472 | *PAG1* | 2.87E–16 | –0.199 |
| cg00476430 | *PAG1* | 4.52E–08 | –0.0721 |
| ch.8.1702176R | *PAG1* | 5.07E–11 | –0.0408 |
| cg13866253 | *PAK1* | 2.58E–10 | 0.0652 |
| cg26996201 | *PAK1* | 1.17E–20 | 0.164 |
| cg13039082 | *PAK1* | 8.78E–21 | 0.223 |
| cg17202086 | *PAK1* | 8.89E–27 | 0.256 |
| cg12269002 | *PAK1* | 1.99E–23 | 0.277 |
| cg18309286 | *PAK1* | 3E–26 | 0.349 |
| cg07990736 | *PAX5* | 9.06E–08 | 0.0986 |
| cg18928153 | *PAX5* | 3.14E–08 | 0.108 |
| cg13910860 | *PAX5* | 6.24E–08 | 0.109 |
| cg06671450 | *PAX5* | 6.75E–12 | 0.12 |
| cg06394247 | *PAX5* | 6.43E–09 | 0.125 |
| cg23916167 | *PAX5* | 2.52E–09 | 0.131 |
| cg00890208 | *PAX5* | 2.87E–11 | 0.137 |
| cg13683040 | *PAX5* | 1.2E–11 | 0.153 |
| cg02034102 | *PAX5* | 4.66E–14 | 0.161 |
| cg14094646 | *PAX5* | 2.71E–13 | 0.163 |
| cg13482308 | *PAX5* | 5.24E–10 | 0.168 |
| cg14267095 | *PAX5* | 7.63E–10 | 0.169 |
| cg00464519 | *PAX5* | 1.47E–13 | 0.173 |
| cg14344261 | *PAX5* | 3E–10 | 0.18 |
| cg14585700 | *PAX5* | 1.23E–11 | 0.185 |
| cg14317609 | *PAX5* | 4.73E–13 | 0.197 |
| cg14306956 | *PAX5* | 3.74E–14 | 0.241 |
| cg07524873 | *PDE2A* | 9.55E–19 | –0.269 |
| cg04538289 | *PDE2A* | 8.12E–16 | –0.222 |
| cg18776207 | *PDE2A* | 2.09E–21 | –0.213 |
| cg25933104 | *PDE2A* | 7.53E–17 | –0.213 |
| cg19988577 | *PDE2A* | 3.96E–14 | –0.195 |
| cg10051714 | *PDE2A* | 3.4E–10 | –0.186 |
| cg19417024 | *PDE2A* | 5.14E–12 | –0.183 |
| cg18574813 | *PDE2A* | 6.11E–14 | –0.18 |
| cg03962783 | *PDE2A* | 8.16E–14 | –0.18 |
| cg20749932 | *PDE2A* | 5.04E–14 | –0.175 |
| cg14378727 | *PDE2A* | 0.000000113 | –0.175 |
| cg00804634 | *PDE2A* | 1.27E–10 | –0.173 |
| cg22646210 | *PDE2A* | 1.51E–13 | –0.164 |
| cg19245310 | *PDE2A* | 6.6E–14 | –0.161 |
| cg03712260 | *PDE2A* | 2.45E–09 | –0.142 |
| cg03673138 | *PDE2A* | 2.48E–11 | –0.139 |
| cg22123156 | *PDE2A* | 1.79E–16 | –0.125 |
| cg04808985 | *PDE2A* | 1.57E–13 | –0.122 |
| cg19341656 | *PDE2A* | 2.89E–17 | –0.119 |
| cg24987622 | *PDE2A* | 5.16E–08 | –0.108 |
| cg14790396 | *PDE2A* | 1.18E–08 | –0.0709 |
| cg14182841 | *PDE2A* | 4.71E–13 | –0.047 |
| cg17801983 | *PDE4C* | 1.69E–11 | 0.0991 |
| cg05606136 | *PDE4C* | 1.64E–09 | 0.107 |
| cg13899108 | *PDE4C* | 6.06E–18 | 0.143 |
| cg26218977 | *PDE4C* | 1.46E–21 | 0.15 |
| cg15215348 | *PDE4C* | 2.83E–14 | 0.213 |
| cg06907708 | *PDE4C* | 5.97E–16 | 0.236 |
| cg14444710 | *PDPK1* | 2.69E–17 | –0.231 |
| cg12428378 | *PGLYRP3* | 6.61E–26 | –0.33 |
| cg08244156 | *PGLYRP3* | 9.36E–22 | –0.296 |
| cg09448880 | *PGLYRP3* | 1.85E–19 | –0.283 |
| cg04450606 | *PGLYRP3* | 2.37E–17 | –0.255 |
| cg06275635 | *PGLYRP3* | 5.78E–15 | –0.24 |
| cg22702025 | *PGLYRP3* | 3.24E–15 | –0.211 |
| cg03222483 | *PGLYRP3* | 4.5E–12 | –0.183 |
| cg21691717 | *PGLYRP4* | 4.86E–24 | –0.33 |
| cg01093088 | *PGLYRP4* | 1.19E–24 | –0.278 |
| cg01798266 | *PGLYRP4* | 1.32E–24 | –0.265 |
| cg03277515 | *PGLYRP4* | 1.08E–18 | –0.253 |
| cg06954535 | *PGLYRP4* | 6.34E–19 | –0.235 |
| cg21400802 | *PGLYRP4* | 1.77E–15 | –0.23 |
| cg02956542 | *PGLYRP4* | 6.15E–19 | –0.158 |
| cg07335619 | *PIK3R5* | 1.54E–23 | –0.268 |
| cg08003321 | *PIK3R5* | 8.49E–19 | –0.222 |
| cg07835141 | *PIK3R5* | 1.57E–17 | –0.213 |
| cg25342409 | *PIK3R5* | 3.22E–16 | –0.2 |
| cg09528351 | *PIK3R5* | 4.21E–16 | –0.2 |
| cg13453139 | *PIK3R5* | 2.56E–14 | –0.199 |
| cg15818905 | *PIK3R5* | 2.85E–11 | –0.196 |
| cg16672810 | *PIK3R5* | 7.96E–18 | –0.186 |
| cg09302474 | *PIK3R5* | 9.2E–11 | –0.159 |
| cg24251850 | *PIK3R5* | 3.3E–15 | –0.153 |
| cg04907571 | *PIK3R5* | 3.86E–11 | –0.15 |
| cg05244974 | *PIK3R5* | 3.91E–16 | –0.142 |
| cg01565508 | *PIK3R5* | 2.78E–10 | –0.14 |
| cg03366884 | *PIK3R5* | 1.75E–08 | –0.126 |
| cg25591996 | *PIK3R5* | 6.28E–08 | –0.108 |
| cg03100801 | *PLCB4* | 6.5E–18 | –0.282 |
| cg20484832 | *PLCB4* | 1.92E–18 | –0.269 |
| cg24736099 | *PLCB4* | 3.98E–19 | –0.257 |
| cg08576827 | *PLCB4* | 1.67E–19 | –0.225 |
| cg09143713 | *PLCB4* | 2.18E–15 | –0.225 |
| cg18870258 | *PLCB4* | 1.36E–14 | –0.225 |
| cg19416290 | *PLCB4* | 2.71E–14 | –0.196 |
| cg22989419 | *PLCB4* | 1.62E–17 | –0.104 |
| cg23194766 | *PPP2R3A* | 3.55E–12 | 0.13 |
| cg04907151 | *PPP2R3A* | 7.55E–16 | 0.183 |
| cg07038400 | *PPP2R3A* | 7.68E–16 | 0.189 |
| cg08406370 | *PRKCB* | 1.7E–10 | 0.162 |
| cg21370856 | *PRKCB* | 6.63E–13 | 0.165 |
| cg03156893 | *PRKCB* | 4.23E–13 | 0.179 |
| cg05436658 | *PRKCB* | 2.08E–16 | 0.196 |
| cg04279973 | *PRKCB* | 1.25E–15 | 0.196 |
| cg03217795 | *PRKCB* | 5.83E–20 | 0.297 |
| cg03306374 | *PRKCB* | 7.38E–17 | 0.31 |
| cg26597539 | *PTGER4* | 7.66E–08 | 0.12 |
| cg16104076 | *PTGER4* | 8.27E–11 | 0.142 |
| cg04092800 | *PTGER4* | 1.07E–17 | 0.213 |
| cg27071460 | *PTGER4* | 2.12E–23 | 0.282 |
| cg05971966 | *PTGER4* | 5.11E–26 | 0.35 |
| cg03045831 | *PTPN13* | 8.34E–09 | 0.0766 |
| cg19429466 | *PTPN13* | 2.56E–10 | 0.102 |
| cg12647643 | *PTPN13* | 1.56E–09 | 0.123 |
| cg04397429 | *PTPN13* | 4.82E–12 | 0.134 |
| cg11117633 | *PTPN13* | 2.48E–10 | 0.136 |
| cg13781843 | *PTPN13* | 1.79E–10 | 0.139 |
| cg01243968 | *PTPN13* | 8.12E–13 | 0.148 |
| cg15543566 | *PTPN13* | 1.13E–11 | 0.183 |
| cg18337337 | *PTPN13* | 9.12E–15 | 0.197 |
| cg05756492 | *RASSF5* | 8.88E–09 | –0.0962 |
| cg10952918 | *RASSF5* | 4.63E–18 | –0.0337 |
| cg24825966 | *RFX4* | 7.27E–10 | 0.116 |
| cg25566352 | *RFX4* | 2.44E–08 | 0.124 |
| cg04508964 | *RFX4* | 4.05E–10 | 0.156 |
| cg18061259 | *RFX4* | 7.74E–11 | 0.191 |
| cg20070090 | *S100A8* | 1.7E–25 | –0.291 |
| cg20335425 | *S100A8* | 9.43E–32 | –0.287 |
| cg20256009 | *S100A8* | 2.44E–27 | –0.229 |
| cg01431057 | *S100A8* | 1.1E–26 | –0.224 |
| cg09174555 | *S100A8* | 1.55E–30 | –0.188 |
| cg24898863 | *S100A8* | 6.98E–19 | –0.144 |
| cg18778827 | *SIGLEC10* | 8.22E–18 | –0.214 |
| cg03582327 | *SIGLEC10* | 4.05E–16 | –0.202 |
| cg19503700 | *SIGLEC10* | 5E–14 | –0.194 |
| cg00481739 | *SIGLEC10* | 1.2E–15 | –0.182 |
| cg03934257 | *SIGLEC10* | 7.27E–13 | –0.16 |
| cg08844849 | *SIGLEC10* | 9.56E–11 | –0.148 |
| cg17669009 | *SIGLEC10* | 5.29E–08 | –0.062 |
| cg27347523 | *SIGLEC5* | 2.89E–11 | –0.227 |
| cg03264071 | *SIGLEC5* | 2.04E–20 | –0.222 |
| cg03541909 | *SIGLEC5* | 3.06E–29 | –0.208 |
| cg09488502 | *SIGLEC5* | 8.21E–09 | –0.175 |
| cg03965138 | *SIGLEC5* | 7.73E–08 | –0.0768 |
| cg24205048 | *SIGLEC5* | 1.64E–09 | –0.0742 |
| cg01193293 | *SIGLEC7* | 8.56E–26 | –0.271 |
| cg00833257 | *SIGLEC7* | 2.29E–28 | –0.255 |
| cg14133643 | *SIGLEC7* | 1.85E–24 | –0.229 |
| cg17923401 | *SIGLEC7* | 2.45E–14 | –0.223 |
| cg23458892 | *SIGLEC7* | 4.67E–21 | –0.211 |
| cg06367655 | *SIGLEC7* | 3.01E–20 | –0.194 |
| cg22911650 | *SIGLEC7* | 2.22E–23 | –0.189 |
| cg00447110 | *SIGLEC7* | 9.05E–12 | –0.133 |
| cg04103198 | *SIRPB1* | 2.09E–19 | –0.298 |
| cg26616780 | *SIRPB1* | 1.91E–14 | –0.241 |
| cg27365103 | *SIRPB1* | 9.28E–18 | –0.222 |
| cg02198625 | *SIRPB1* | 3.45E–13 | –0.186 |
| cg09577651 | *SIRPB1* | 3.26E–12 | –0.13 |
| cg23295754 | *SIRPG* | 7.94E–20 | –0.28 |
| cg11061975 | *SIRPG* | 1.94E–20 | –0.27 |
| cg10131232 | *SIRPG* | 2.87E–21 | –0.263 |
| cg13408605 | *SIRPG* | 1.3E–23 | –0.261 |
| cg03745414 | *SIRPG* | 5.72E–09 | –0.119 |
| cg11738543 | *SOCS2* | 8.87E–10 | 0.154 |
| cg23412850 | *SOCS2* | 7.19E–11 | 0.172 |
| cg04797323 | *SOCS2* | 3.86E–12 | 0.23 |
| cg13508904 | *SPTAN1* | 0.000000117 | –0.0882 |
| cg14055502 | *SYK* | 1.69E–18 | –0.248 |
| cg14054883 | *SYK* | 5.24E–16 | –0.245 |
| cg14424519 | *SYK* | 4.79E–17 | –0.242 |
| cg13782919 | *SYK* | 4.38E–15 | –0.203 |
| cg14005120 | *SYK* | 2.24E–13 | –0.195 |
| cg13897882 | *SYK* | 1.04E–09 | –0.186 |
| cg05266212 | *TBX21* | 7.83E–08 | –0.106 |
| cg04451353 | *TREM1* | 2.56E–21 | –0.299 |
| cg18505453 | *TREM1* | 4.44E–14 | –0.238 |
| cg09310966 | *TREM1* | 1.28E–15 | –0.237 |
| cg21328082 | *TREM1* | 1.14E–21 | –0.197 |
| cg03843170 | *TREM1* | 3.4E–12 | –0.185 |
| cg06196379 | *TREM1* | 4.2E–17 | –0.184 |
| cg10981439 | *TREM1* | 1.2E–16 | –0.175 |
| cg04456284 | *TREM2* | 3.25E–21 | –0.273 |
| cg20095587 | *TREM2* | 7.2E–18 | –0.268 |
| cg25748868 | *TREM2* | 8.8E–16 | –0.241 |
| cg00718409 | *TREM2* | 1.75E–17 | –0.238 |
| cg10725937 | *TREM2* | 1.23E–14 | –0.211 |
| cg02539671 | *TREM2* | 4.72E–11 | –0.175 |
| cg01980222 | *TREM2* | 1.49E–12 | –0.154 |

*Methylation probe ID in GSE54503 dataset.

†Student's *t*-test. Bonferroni adjustment was used to correct for multiple comparisons, and *P*<1.37×10^-7^ was considered to be statistically significant in view of the 365,600 methylation probes in GSE54503 dataset.

Abbreviations: FC, log_2_ (Fold changes), HCCs vs. adjacent non-tumor tissues.
