# Supplementary material for: Relatively Small Contribution of Methylation and Genomic Copy Number Aberration to the Aberrant Expression of Inflammation-Related Genes in HBV-Related Hepatocellular Carcinoma
Source: PLoS One. 2015 May 12;10(5):e0126836. doi: 10.1371/journal.pone.0126836 (PMC4429029; doi:10.1371/journal.pone.0126836)
Supplement: S11 Table — (DOC) [file pone.0126836.s013.doc]

**S11 Table. 47 Aberrantly Methylated Inflammation-related Genes Validated in GSE37988 Dataset**

| **ID*** | **Symbol** | **P Value**† | **FC** |
| --- | --- | --- | --- |
| cg13626881 | *ADORA1* | 1.48E–07 | –0.36905 |
| cg14310034 | *BMP4* | 1.58E–21 | 1.862329 |
| cg15494458 | *BPI* | 3.13E–10 | –1.18037 |
| cg08254089 | *BPI* | 1.22E–07 | –0.64857 |
| cg00108454 | *C1QA* | 1.84E–07 | –0.46386 |
| cg17118262 | *CCL1* | 4.17E–08 | –0.84531 |
| cg20556988 | *CCL1* | 4.49E–08 | –0.62053 |
| cg02706575 | *CCL13* | 1.26E–07 | –0.72631 |
| cg24615251 | *CCL13* | 5.63E–08 | –0.46069 |
| cg21643045 | *CCL20* | 9.44E–19 | –0.88872 |
| cg09425228 | *CCL20* | 3.11E–08 | –0.58082 |
| cg08124722 | *CCL7* | 8.25E–10 | –0.61119 |
| cg02936263 | *CCL7* | 1.56E–08 | –0.57911 |
| cg13615963 | *CCR6* | 2.31E–13 | –0.85078 |
| cg07950803 | *CD1A* | 8.9E–11 | –1.0429 |
| cg10129493 | *CD33* | 1.02E–09 | –0.89374 |
| cg11122968 | *CD33* | 6.74E–08 | –0.58733 |
| cg11944101 | *CD40LG* | 1.52E–07 | –0.65161 |
| cg02992767 | *CD40LG* | 8.11E–07 | –0.61815 |
| cg23181133 | *CEACAM3* | 4.02E–09 | –0.72453 |
| cg23507131 | *DARC* | 7.07E–07 | –0.49171 |
| cg16899306 | *HLA-DQB2* | 2.86E–10 | –0.9349 |
| cg04345908 | *HLA-DQB2* | 8.01E–09 | –0.42364 |
| cg14837082 | *HRH1* | 1.17E–06 | –0.6006 |
| cg20277670 | *HRH2* | 1.65E–08 | –0.37761 |
| cg27351998 | *IFNA5* | 1.34E–08 | –0.64107 |
| cg26227465 | *IFNG* | 1.1E–06 | –0.62103 |
| cg00488364 | *IL13RA2* | 3.8E–07 | –0.60427 |
| cg21282997 | *IL18RAP* | 3.18E–08 | –0.78257 |
| cg18156583 | *IL18RAP* | 7.58E–07 | –0.66046 |
| cg11916609 | *IL1RL1* | 8.49E–07 | –0.64708 |
| cg02656594 | *IL21R* | 9.69E–07 | –0.83847 |
| cg26333641 | *IL22* | 7.1E–07 | –0.62806 |
| cg13993218 | *INS* | 2.53E–12 | –0.92791 |
| cg25336198 | *INS* | 1.54E–10 | –0.86562 |
| cg00613255 | *INS* | 1.16E–09 | –0.70511 |
| cg03366382 | *INS* | 2.31E–08 | –0.34327 |
| cg12741420 | *IRF4* | 1.02E–07 | 1.377803 |
| cg25384595 | *LILRA1* | 7.3E–11 | –0.91233 |
| cg19486673 | *LILRA2* | 1.99E–10 | –1.06735 |
| cg00705255 | *LILRA3* | 1.77E–10 | –0.90989 |
| cg13733733 | *LILRA3* | 1.84E–08 | –0.60811 |
| cg20542190 | *LILRA4* | 1.78E–11 | –0.89897 |
| cg01204985 | *LILRA4* | 1.72E–09 | –0.66385 |
| cg06392096 | *LILRA5* | 4.37E–13 | –0.6051 |
| cg00727947 | *LILRA5* | 1.47E–07 | –0.46098 |
| cg19279346 | *LILRB2* | 6.94E–09 | –0.89951 |
| cg05248470 | *LILRB2* | 4.88E–08 | –0.74619 |
| cg22456522 | *LILRB3* | 2.97E–08 | –0.82574 |
| cg05922591 | *LILRB4* | 1.93E–08 | –0.5304 |
| cg08684473 | *LILRB5* | 2.41E–11 | –0.83076 |
| cg20649991 | *LILRB5* | 3.39E–13 | –0.70476 |
| cg19601328 | *MAP3K14* | 2.12E–09 | 0.295091 |
| cg11009736 | *MARCO* | 8.35E–10 | –1.06043 |
| cg16303562 | *MSR1* | 6.74E–11 | –0.74775 |
| cg01668126 | *MSR1* | 1.75E–10 | –0.67974 |
| cg13899108 | *PDE4C* | 2.68E–11 | 0.333947 |
| cg14444710 | *PDPK1* | 1.59E–10 | –0.54706 |
| cg09448880 | *PGLYRP3* | 1.25E–07 | –0.7319 |
| cg24736099 | *PLCB4* | 9.36E–09 | –0.42782 |
| cg24898863 | *S100A8* | 1.7E–12 | –1.02495 |
| cg20070090 | *S100A8* | 3.28E–10 | –0.82831 |
| cg01193293 | *SIGLEC7* | 6.19E–10 | –1.09146 |
| cg23458892 | *SIGLEC7* | 2.57E–07 | –0.53945 |
| cg09577651 | *SIRPB1* | 7.32E–07 | –0.61323 |
| cg06196379 | *TREM1* | 7.99E–10 | –0.96542 |
| cg01980222 | *TREM2* | 1.17E–07 | –0.65677 |
| cg20095587 | *TREM2* | 6.26E–08 | –0.6341 |

*Methylation probe ID in GSE37988 dataset.

†Student's *t*-test. Bonferroni adjustment was used to correct for multiple comparisons, and *P*<1.8×10^-6^ was considered to be statistically significant in view of the 27,578 methylation probes in GSE37988 dataset.

Abbreviations: FC, log_2_ (Fold changes), HCCs vs. adjacent non-tumor tissues.
