# Supplementary material for: Relatively Small Contribution of Methylation and Genomic Copy Number Aberration to the Aberrant Expression of Inflammation-Related Genes in HBV-Related Hepatocellular Carcinoma
Source: PLoS One. 2015 May 12;10(5):e0126836. doi: 10.1371/journal.pone.0126836 (PMC4429029; doi:10.1371/journal.pone.0126836)
Supplement: S12 Table — (DOC) [file pone.0126836.s014.doc]

**S12 Table. Networks Constructed by the Inflammation-related Genes with DNA Methylation Changes Associated with Inverse Expression Changes in HCC**

| **No.** | **Network** | **GO processes** | **Total nodes** | **Seed nodes** | **Pathways** | **gScore*** |
| --- | --- | --- | --- | --- | --- | --- |
| 1 | PKC-beta, Bcl-2, FAP-1, ESR, PKC | response to alcohol (42.0%; 7.183e-20), response to organic cyclic compound (54.0%; 1.810e-19), immune response-regulating cell surface receptor signaling pathway (36.0%; 7.994e-18), response to steroid hormone stimulus (42.0%; 1.052e-17), immune response-regulating signaling pathway (38.0%; 2.658e-17) | 50 | 9 | 0 | 76.97 |
| 2 | PKC-beta, PKC-beta2, PKC, ESR1 (nuclear), MARCKS | platelet activation (63.2%; 3.309e-18), protein phosphorylation (78.9%; 3.381e-18), phosphorylation (78.9%; 6.404e-16), regulation of sequence-specific DNA binding transcription factor activity (63.2%; 1.429e-15), negative regulation of insulin receptor signaling pathway (36.8%; 2.593e-15) | 19 | 4 | 0 | 55.49 |
| 3 | Fyn, PKC-beta2, ESR1 (nuclear), cPKC (conventional), PKC-beta | response to organic cyclic compound (60.0%; 2.475e-23), positive regulation of cellular process (86.0%; 3.534e-22), response to oxygen-containing compound (66.0%; 7.929e-22), response to endogenous stimulus (66.0%; 1.129e-21), response to growth factor stimulus (48.0%; 4.974e-21) | 50 | 6 | 0 | 51.27 |
| 4 | PKC, PKC-beta2, ESR1 (membrane), c-Fos, RARbeta | cellular response to calcium ion (28.0%; 2.251e-25), response to steroid hormone stimulus (52.0%; 1.205e-24), response to progesterone stimulus (30.0%; 1.610e-24), response to ketone (38.0%; 8.565e-24), response to lipid (60.0%; 1.116e-23) | 50 | 4 | 0 | 34.14 |
| 5 | ESR1 (nuclear), N-Ras, FAP-1, PAK2, PSMC5 | nucleosome assembly (20.8%; 4.987e-13), DNA packaging (22.9%; 1.340e-12), chromatin assembly (20.8%; 1.413e-12), nucleosome organization (20.8%; 4.102e-12), protein-DNA complex assembly (20.8%; 4.102e-12) | 50 | 3 | 0 | 25.58 |
| 6 | ESR1 (nuclear), MCM7, HOTAIR, FLJ20366, Link-GEFII | intein-mediated protein splicing (8.9%; 2.301e-11), protein splicing (8.9%; 2.301e-11), prostate epithelial cord elongation (8.9%; 8.013e-10), prostate gland morphogenetic growth (8.9%; 1.600e-09), regulation of reproductive process (15.6%; 1.451e-08) | 50 | 2 | 0 | 17.01 |
| 7 | ESR1 (nuclear), MSX-2, eEF1A1, LAMB1, tRNA | neuronal-glial interaction involved in cerebral cortex radial glia guided migration (23.3%; 6.743e-20), substrate adhesion-dependent cell spreading (30.0%; 9.279e-18), regulation of embryonic development (36.7%; 4.262e-17), extracellular matrix organization (46.7%; 2.212e-16), extracellular structure organization (46.7%; 2.288e-16) | 50 | 1 | 0 | 9.72 |
| 8 | CCL20, c-Jun, p22-phox, Aha1, GABA-A receptor gamma-2 subunit | gamma-aminobutyric acid signaling pathway (66.0%; 2.214e-76), chloride transport (70.0%; 1.372e-68), inorganic anion transport (70.0%; 2.629e-64), synaptic transmission, GABAergic (44.0%; 4.087e-51), anion transport (70.0%; 5.574e-44) | 50 | 1 | 0 | 8.92 |

*Evaluation of saturation with genes and Canonical pathways in network.
