# Supplementary material for: Relatively Small Contribution of Methylation and Genomic Copy Number Aberration to the Aberrant Expression of Inflammation-Related Genes in HBV-Related Hepatocellular Carcinoma
Source: PLoS One. 2015 May 12;10(5):e0126836. doi: 10.1371/journal.pone.0126836 (PMC4429029; doi:10.1371/journal.pone.0126836)
Supplement: S13 Table — (DOC) [file pone.0126836.s015.doc]

**S13 Table. Networks Constructed by the Inflammation-related Genes with SCNAs Associated with Expression Changes in HCC**

| **No** | **Network** | **GO processes** | **Total nodes** | **Seed nodes** | **Pathways** | **gScore*** |
| --- | --- | --- | --- | --- | --- | --- |
| 1 | FLT3, GRB2, TCF8, Beta-catenin, Fyn | immune response-regulating signaling pathway (63.3%; 1.730e-36), Fc receptor signaling pathway (55.1%; 4.303e-36), innate immune response (69.4%; 1.721e-33), regulation of immune system process (77.6%; 2.041e-33), regulation of immune response (69.4%; 6.429e-33) | 52 | 10 | 187 | 271.81 |
| 2 | PP2A catalytic, ESR1 (nuclear), cPKC (conventional), PDGF receptor, PDGF-R-alpha | intracellular signal transduction (77.6%; 2.212e-30), response to growth factor stimulus (61.2%; 2.861e-30), enzyme linked receptor protein signaling pathway (65.3%; 3.008e-30), fibroblast growth factor receptor signaling pathway (42.9%; 1.506e-29), cellular response to growth factor stimulus (59.2%; 2.041e-29) | 50 | 14 | 0 | 53.39 |
| 3 | PARP-1, Calpain 1(mu), Beta-catenin, cPKC (conventional), PI3K cat class IA (p110-beta) | response to growth factor stimulus (63.3%; 6.829e-32), positive regulation of cellular process (98.0%; 3.138e-31), cellular response to growth factor stimulus (61.2%; 4.980e-31), cellular response to organic substance (81.6%; 1.263e-29), intracellular signal transduction (75.5%; 7.151e-29) | 50 | 14 | 0 | 53.39 |
| 4 | PKC-beta, PKC-beta2, cPKC (conventional), FAP-1, c-Raf-1 | neurotrophin TRK receptor signaling pathway (42.9%; 1.400e-24), neurotrophin signaling pathway (42.9%; 1.900e-24), immune response-regulating cell surface receptor signaling pathway (44.9%; 4.541e-24), fibroblast growth factor receptor signaling pathway (36.7%; 5.519e-24), cellular response to endogenous stimulus (61.2%; 5.703e-24) | 50 | 13 | 0 | 49.56 |
| 5 | SDF-1, c-Raf-1, 14-3-3, FAF1, PIAS1 | regulation of signal transduction (76.0%; 1.245e-23), regulation of signaling (78.0%; 8.241e-23), regulation of cell communication (78.0%; 8.980e-23), positive regulation of cellular metabolic process (74.0%; 1.462e-22), response to growth factor stimulus (50.0%; 2.027e-22) | 50 | 13 | 0 | 49.56 |
| 6 | ESR1 (nuclear), IRF8, PIAS1, AKR1C3, PDGF-R-alpha | positive regulation of cellular metabolic process (76.6%; 7.215e-23), positive regulation of metabolic process (76.6%; 6.159e-22), positive regulation of macromolecule metabolic process (72.3%; 1.568e-20), regulation of primary metabolic process (93.6%; 2.464e-20), regulation of cellular metabolic process (93.6%; 3.068e-20) | 50 | 11 | 5 | 49.48 |
| 7 | c-Raf-1, PKC, PP2A cat (alpha), Beta-catenin, ESR1 (membrane) | immune response-regulating cell surface receptor signaling pathway (53.2%; 1.314e-29), cellular response to fibroblast growth factor stimulus (44.7%; 6.255e-29), response to fibroblast growth factor stimulus (44.7%; 9.136e-29), fibroblast growth factor receptor signaling pathway (42.6%; 4.138e-28), immune response-regulating signaling pathway (53.2%; 5.907e-27) | 50 | 11 | 4 | 47.33 |
| 8 | Cortactin, FAK1, Fyn, ST2(L), PIAS1 | fibroblast growth factor receptor signaling pathway (41.7%; 7.031e-28), Fc receptor signaling pathway (45.8%; 3.067e-27), cellular response to fibroblast growth factor stimulus (41.7%; 7.829e-27), response to fibroblast growth factor stimulus (41.7%; 1.121e-26), neurotrophin TRK receptor signaling pathway (45.8%; 1.651e-26) | 50 | 12 | 0 | 45.73 |
| 9 | Beta-catenin, RHEB2, PKC-beta2, c-Raf-1, 14-3-3 | positive regulation of cellular process (91.8%; 6.893e-26), negative regulation of cellular process (87.8%; 2.857e-24), regulation of signal transduction (77.6%; 3.430e-24), positive regulation of biological process (91.8%; 1.462e-23), apoptotic process (61.2%; 4.564e-23) | 50 | 12 | 0 | 45.73 |
| 10 | GDNF, ITGA6, CXCL14, CD166, CD30L (TNFSF8) | signal transduction (89.4%; 4.107e-18), signaling (91.5%; 9.349e-18), single organism signaling (91.5%; 9.349e-18), cAMP metabolic process (21.3%; 1.519e-17), cell communication (91.5%; 2.730e-17) | 50 | 11 | 0 | 42.33 |
| 11 | Beta-catenin, FAK1, PI3K cat class IA (p110-beta), GRB2, PKC-beta2 | intracellular signal transduction (83.0%; 2.805e-33), transmembrane receptor protein tyrosine kinase signaling pathway (63.8%; 3.234e-32), neurotrophin TRK receptor signaling pathway (53.2%; 4.040e-32), neurotrophin signaling pathway (53.2%; 5.840e-32), enzyme linked receptor protein signaling pathway (66.0%; 1.520e-29) | 50 | 11 | 0 | 41.89 |
| 12 | CREB1, GRB2, BCL2L13, MENA, C8alpha | regulation of response to stimulus (71.4%; 2.769e-16), complement activation, alternative pathway (12.2%; 4.862e-13), cytolysis (14.3%; 8.952e-13), regulation of complement activation (14.3%; 1.821e-12), regulation of protein activation cascade (14.3%; 2.272e-12) | 50 | 10 | 0 | 38.86 |
| 13 | PLC-beta1, FKHR, MEK3(MAP2K3), IL-16, 14-3-3 theta | intracellular signal transduction (71.4%; 1.849e-22), immune response-regulating signaling pathway (47.6%; 1.517e-20), innate immune response (50.0%; 6.015e-17), positive regulation of biological process (85.7%; 7.543e-17), regulation of immune system process (57.1%; 9.643e-17) | 50 | 9 | 0 | 34.95 |
| 14 | FKHR, PIAS1, PKC, c-Raf-1, CaMK IV | innate immune response (63.8%; 5.177e-28), positive regulation of cellular process (95.7%; 5.620e-28), positive regulation of cellular metabolic process (83.0%; 4.347e-27), intracellular signal transduction (74.5%; 5.056e-27), cellular response to growth factor stimulus (57.4%; 5.613e-27) | 50 | 9 | 0 | 34.23 |
| 15 | FKHR, 14-3-3 theta, Calpain 1(mu), ESR, IRF8 | cellular response to growth factor stimulus (70.8%; 2.338e-38), intracellular signal transduction (87.5%; 9.358e-38), response to growth factor stimulus (70.8%; 1.734e-37), regulation of cell death (83.3%; 1.418e-34), regulation of apoptotic process (81.2%; 8.834e-34) | 50 | 9 | 0 | 34.23 |
| 16 | ESR1 (nuclear), EDNRB, PDGF-R-alpha, IRF8, NCOA2 (GRIP1/TIF2) | elevation of cytosolic calcium ion concentration (48.0%; 1.592e-33), cytosolic calcium ion homeostasis (48.0%; 1.346e-31), G-protein coupled receptor signaling pathway (76.0%; 2.781e-30), cellular divalent inorganic cation homeostasis (50.0%; 1.986e-28), divalent inorganic cation homeostasis (50.0%; 7.084e-28) | 50 | 8 | 0 | 30.4 |
| 17 | EDNRB, Bcl-2, PKC, Calpain 1(mu), CREB1 | cellular response to organic substance (81.6%; 1.263e-29), intracellular signal transduction (73.5%; 2.077e-27), cellular response to chemical stimulus (81.6%; 2.478e-26), positive regulation of molecular function (69.4%; 1.677e-25), response to organic substance (83.7%; 8.237e-25) | 50 | 8 | 0 | 30.4 |
| 18 | ESR, STAT4, FAF1, FAP-1, TTRAP | response to DNA damage stimulus (54.3%; 1.032e-23), G1/S transition of mitotic cell cycle (39.1%; 2.438e-23), negative regulation of mitotic cell cycle phase transition (37.0%; 3.716e-22), negative regulation of cell cycle phase transition (37.0%; 5.087e-22), negative regulation of cell cycle process (39.1%; 1.117e-21) | 52 | 8 | 0 | 30.09 |
| 19 | CREB1, CaMK IV, c-Raf-1, Bcl-2, Ca('2+) cytosol | translation (62.5%; 1.292e-31), cellular protein metabolic process (90.0%; 1.369e-23), protein metabolic process (90.0%; 2.113e-20), cellular macromolecule metabolic process (95.0%; 3.245e-15), macromolecule metabolic process (95.0%; 1.693e-13) | 50 | 4 | 9 | 26.32 |
| 20 | CREB1, LPP, C17orf96, NIFIE14, MRPL43 | cAMP catabolic process (23.3%; 1.331e-22), cyclic nucleotide catabolic process (23.3%; 4.644e-22), cGMP catabolic process (16.3%; 4.791e-18), cAMP metabolic process (23.3%; 5.665e-18), cyclic nucleotide metabolic process (23.3%; 1.805e-15) | 50 | 6 | 0 | 22.73 |
| 21 | ATF-1, CaMK IV, PI3K cat class IA, EDNRB, PKC-delta | intracellular signal transduction (59.6%; 7.672e-18), signaling (91.5%; 9.349e-18), single organism signaling (91.5%; 9.349e-18), cell communication (91.5%; 2.730e-17), signal transduction (87.2%; 7.299e-17) | 50 | 6 | 0 | 22.73 |
| 22 | CREB1, Hamartin, B3GA1, ZNF23, G-protein beta-4 | transferrin transport (13.3%; 1.094e-09), ATP hydrolysis coupled proton transport (13.3%; 1.094e-09), energy coupled proton transmembrane transport, against electrochemical gradient (13.3%; 1.245e-09), ferric iron transport (13.3%; 1.598e-09), trivalent inorganic cation transport (13.3%; 1.598e-09) | 50 | 5 | 0 | 19.3 |
| 23 | ESR1 (nuclear), BCL2L15, PRKCBP1, PSMD7, alpha-2/beta-1 integrin | regulation of cellular amino acid metabolic process (39.6%; 5.012e-36), negative regulation of ubiquitin-protein ligase activity involved in mitotic cell cycle (39.6%; 1.449e-34), signal transduction involved in mitotic DNA integrity checkpoint (39.6%; 2.514e-34), signal transduction involved in mitotic cell cycle checkpoint (39.6%; 2.514e-34), DNA damage response, signal transduction by p53 class mediator resulting in cell cycle arrest (39.6%; 2.514e-34) | 50 | 4 | 0 | 15.07 |
| 24 | CREB1, Substance P receptor, PDE7A, GABA-A receptor alpha-1 subunit, eIF3S10 | G-protein coupled receptor signaling pathway (80.0%; 2.111e-33), gamma-aminobutyric acid signaling pathway (32.0%; 1.324e-29), chloride transport (34.0%; 4.216e-26), cell surface receptor signaling pathway (88.0%; 1.515e-25), synaptic transmission (54.0%; 1.490e-24) | 50 | 4 | 0 | 15.07 |
| 25 | CREB1, CTTNBP2NL, GluR3, ARPC4, MRPL40 | transferrin transport (35.4%; 5.307e-34), ferric iron transport (35.4%; 1.839e-33), trivalent inorganic cation transport (35.4%; 1.839e-33), phagosome maturation (35.4%; 9.059e-32), ATP hydrolysis coupled proton transport (33.3%; 1.743e-31) | 50 | 3 | 0 | 11.74 |
| 26 | CREB1, FAM167A, MRPL4, MLF2, G-protein gamma 13 | formation of translation preinitiation complex (18.8%; 1.152e-19), G-protein coupled receptor signaling pathway (56.2%; 1.079e-16), phospholipase C-activating G-protein coupled receptor signaling pathway (22.9%; 1.128e-15), neuropeptide signaling pathway (22.9%; 2.396e-14), regulation of translational initiation (18.8%; 3.269e-13) | 50 | 3 | 0 | 11.24 |
| 27 | CREB1, Rab-34, Sgsm1, LAMR1, OBFC2B | retrograde axon cargo transport (10.6%; 2.726e-11), microtubule-based movement (19.1%; 9.941e-10), organelle organization (51.1%; 1.061e-09), localization (66.0%; 5.368e-09), transport (59.6%; 7.055e-09) | 50 | 2 | 0 | 7.65 |

*Evaluation of saturation with genes and Canonical pathways in network.
